# A general ^11^C-labeling approach enabled by fluoride-mediated desilylation of organosilanes

**DOI:** 10.1038/s41467-020-15556-7

**Published:** 2020-04-08

**Authors:** Wenchao Qu, Bao Hu, John W. Babich, Nicole Waterhouse, Marybeth Dooley, Shashikanth Ponnala, Julie Urgiles

**Affiliations:** 1 Citigroup Biomedical Imaging Center, Weill Cornell Medicine, New York, NY USA; 2 Division of Radiopharmaceutical Sciences, Department of Radiology, Weill Cornell Medicine, New York, NY USA; 3 Molecular Imaging Innovations Institute (MI3), Weill Cornell Medicine, New York, NY USA; 4 Present Address: Department of Psychiatry, Stony Brook University, Stony Brook, NY USA

**Keywords:** Medicinal chemistry, Nuclear chemistry, Synthetic chemistry methodology

## Abstract

Carbon-11 (^11^C) is one of the most ideal positron emitters for labeling bioactive molecules for molecular imaging studies. The lack of convenient and fast incorporation methods to introduce ^11^C into organic molecules often hampers the use of this radioisotope. Here, a fluoride-mediated desilylation (FMDS) ^11^C-labeling approach is reported. This method relies on thermodynamically favored Si-F bond formation to generate a carbanion, therefore enabling the highly efficient and speedy incorporation of [^11^C]CO_2_ and [^11^C]CH_3_I into molecules with diversified structures. It provides facile and rapid access to ^11^C-labeled compounds with carbon-11 attached at various hybridized carbons as well as oxygen, sulfur and nitrogen atoms with broad functional group tolerance. The exemplified syntheses of several biologically and clinically important radiotracers illustrates the potentials of this methodology.

## Abstract (web summary)

Convenient and fast methods to introduce ^11^C into organic molecules are of great help for molecular imaging studies. Here, the authors developed an efficient incorporation of [^11^C]CO_2_ and [^11^C]CH_3_I into molecules via a fluoride-mediated desilylation process.

## Introduction

Positron emission tomography (PET) is a clinical and research imaging modality for the non-invasive investigation of biochemical and molecular events in living organisms using radioactive positron emitting tracers. In the past two decades, the application of PET to the study of various diseases (oncological, neurological and cardiovascular) has positioned this modality as one of the most powerful translational imaging tools available. Among several short-lived positron-emitting radionuclides used in PET imaging, carbon-11 (^11^C, *t*_1/2_ = 20.4 min, *E*_β+_ = 1.98 MeV) stands out as unique^1–3^. The ubiquitous presence of carbon in organic molecules makes ^11^C an attractive and important positron-emitting radionuclide for labeling a vast array of molecules of biological interest. Importantly, ^11^C-labeled molecules possess the same chemical and biological properties as the non-radioactive ^12^C-molecule of interest; thus making ^11^C extraordinarily useful for the exploration of molecules with well-characterized biological and pharmacological properties (i.e., metabolism, drug pharmacokinetics, receptor binding affinity, enzyme substrate affinity, etc.). In addition, the short half-life of ^11^C enables the possibility of performing multiple imaging studies in the same subject on a single day, which is especially beneficial for clinical researches. This unique utility of ^11^C demands that there be rapid and robust approaches available to incorporate ^11^C into various organic molecules in an efficient manner.

Carbon-11 labeled PET tracer syntheses generally start with the cyclotron production of [^11^C]CO_2_ or [^11^C]CH_4_, which are produced by the proton bombardment of N_2_ gas (doped with O_2_ or H_2_) using the ^14^N(p,α)^11^C nuclear reaction. The most commonly produced radioactive intermediate [^11^C]CO_2_, can be either used directly as a primary radiosynthon or be rapidly converted to more reactive secondary radiosynthons ([^11^C]CH_3_I, [^11^C]CH_3_OTf, [^11^C]HCN, [^11^C]HCHO, [^11^C]CO, [^11^C]COCl_2_, etc.), enabling a variety of radiosynthetic strategies. After postreaction purification, typically using chromatographic and/or solid phase extraction (SPE) techniques, the desired ^11^C-labeled tracer is isolated and formulated for use in preclinical or clinical studies. The main challenge for a successful synthesis of ^11^C-labeled PET tracers is a rapid, robust, and practical radiolabeling method that yields a desired tracer dose of pharmaceutically acceptable quality, i.e. high radiochemical & chemical purity, and typically high specific activity (*A*_s_) or molar activity (*A*_m_). In addition, such a method should be easily adapted for automation to minimize radiation exposure to the operator, and the labeling precursors should be either commercially available or readily synthesized^1–3^.

[^11^C]CO_2_ has been directly used for PET tracer synthesis for decades. It is now routinely produced for the synthesis of carbon-11 labeled fatty acids such as [^11^C]acetic acid and [^11^C]palmitic acid via the Grignard reaction in many PET facilities worldwide. However, because of its inertness, radiolabeling reactions using [^11^C]CO_2_ generally require the use of strong organometallic reagents, such as Grignard and organolithium reagents, which are sensitive to moisture and atmospheric ^12^CO_2_ and less tolerant to the presence of various functional groups. These drawbacks impede the broader application of [^11^C]CO_2_ for synthesizing highly functionalized molecules. To expand the utility of [^11^C]CO_2_ for the synthesis of PET tracers with diverse structures, including multiple functional groups, researchers have recently reported on both the incorporation of [^11^C]CO_2_ into molecules under milder conditions, and the introduction of ^11^C to specific positions that are not easily achieved using other secondary radiosynthons^4–10^. Despite such developments, the method of directly incorporating [^11^C]CO_2_ for synthesis of ^11^C-labeled radiotracers remains sorely lacking in the field of PET chemistry. Hence, there is a heretofore unmet need to develop radiolabeling methodologies that can directly and promptly introduce [^11^C]CO_2_ to molecules with diverse structure and/or containing multiple functional groups under mild reaction conditions.

Herein, we report our recent development of a fluoride-mediated desilylation (FMDS) ^11^C-labeling approach derived from a fluoride desilylation promoted nucleophilic reaction^11^. Carbon-11 labeled carboxylic acids containing various functional groups with ^11^C attached at different hybridized carbons (sp, sp^2^, and sp^3^) are synthesized by in situ generation of various nucleophiles via fluoride agents and organosilanes, followed by a quick ^11^C-carboxylation reaction. Moreover, this method is also readily extended to label organic molecules with [^11^C]CH_3_I as the radiolabeling synthon.

## Results

### Synthesis of [^11^C]acetoacetic acid via FMDS ^11^C-carboxylation

**Fig. 1:**
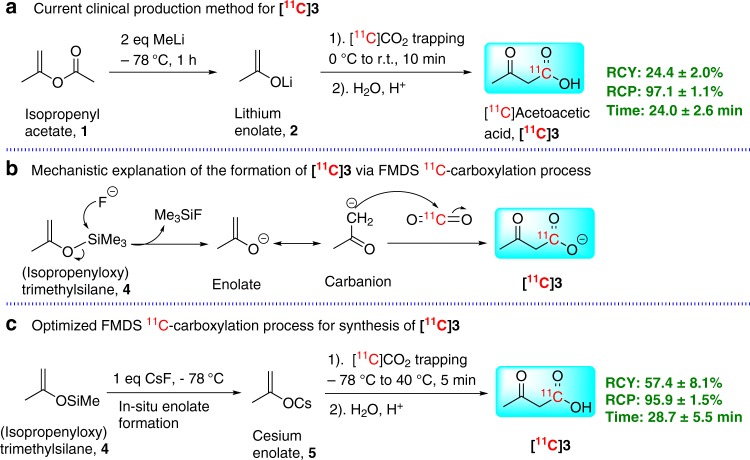
Methods for synthesizing [^11^C]3. Two cGMP-compliant synthesis methods for preparing [^**11**^**C**]**3.** **a** organolithium method; **c** FMDS ^11^C-carboxylation method, and mechanistic explanation of formation of [^**11**^**C**]**3** via FMDS ^11^C-carboxylation approach (**b**).

To support the PET imaging study of the metabolic process of ketone bodies in brain tumors, we recently developed a production method for [^11^C]acetoacetic acid (**[**^**11**^**C]3**) following a literature method^12,13^, in which isopropenyl acetate, **1**, was first reacted with methyl lithium to form lithium enolate, **2**. Next, the enolate, **2**, was used for reacting with [^11^C]CO_2_ to form the desired product **[**^**11**^**C]3** (Fig. 1a). One of the difficulties encountered in the production process was that the chemical impurities were occasionally detected in the final product and proved difficult to remove using standard purification methods. To assure robust production of a high quality **[**^**11**^**C]3** tracer, we sought to develop a different approach to [^11^C]CO_2_ incorporation under milder conditions, such conditions would avoid the use of a harsh organometallic reagent, which not only generated the desired enolate but also caused some side reaction(s). After an extensive literature search, we found that a fluoride ion desilylation enolate generation method reported by Noyori in 1983 could be amenable for this purpose^14^.

The exploration of a FMDS ^11^C-carboxylation strategy for synthesis of **[**^**11**^**C]3** began by using (isopropenyloxy)trimethylsilane, **4**, as the organosilane reagent and tris(dimethylamino)sulfonium difluorotrimethylsilicate (TASF) as the fluoride ion source^14,15^. Binary solvent systems (tetrahydrofuran (THF) and dichloromethane (DCM)) were employed for the reaction due to the low solubility of TASF in THF^16^. The initial experiment provided us **[**^**11**^**C]3** with a 5% radiochemical yield (RCY, based on HPLC analysis of the crude product). The formation of **[**^**11**^**C]3** via FMDS ^11^C-carboxylation approach could be understood from the mechanistic scheme in Fig. 1b. The driving force of the enolate anion formation under such mild conditions comes from the strength of the silicon-fluorine bond (139 kcal/mol), which makes enolate generation via fluoride-mediated desilylation as a thermodynamically favored process^14^. After several preliminary experiments, the RCY (based on HPLC analysis of the crude product) of **[**^**11**^**C]3** quickly improved to over 50%. However, purification with either ion-exchange or semi-prep HPLC methods failed to provide a product with acceptable chemical purity. This was due to the large amount of TASF reagent and water incompatible solvent DCM used for reactions. At this stage, cesium fluoride (CsF) was tested as a replacement for TASF to overcome these problems^17,18^. The modification of experimental conditions (such as using THF and dimethylformamide (DMF) (3/1, V/V) binary solvents, trapping [^11^C]CO_2_ at low temperature, pre-drying the CsF reagent using azeotropic evaporation, adjusting the quantity of the ion exchange resin as well as introducing an Al–N cartridge at the end of the purification process for removal of extra fluoride ion) dramatically improved the overall reaction yield and the chemical purity of the final product. An optimized synthetic protocol (Fig. 1c) was developed that was suitable for production under cGMP compliant conditions. The product **[**^**11**^**C]3** was synthesized with high radiochemical purity (RCP, 95.9 ± 1.5%) and high RCY (57.4 ± 8.1%, *n* = 3; decay corrected, DC), in a similar time frame (from end of [^11^C]CO_2_ collection to end of product collection, 28.7 ± 5.5 min) compared with the results of the organolithium reagent based method (RCP, 97.1 ± 1.1%; RCY, 24.4 ± 2.0%; 24.0 ± 2.6 min, *n* = 3). It is noticeable that both production processes developed by us needed longer production time comparing with the automated production reported by Sébastien Tremblay, et al in 2007 (RCY, 34 ± 5%; 18 min, *n* = 20)^12^. The development of automated synthesis process in the future, hopefully, could help us to shorten the whole production time. An important feature of this updated method is that both reagents used (enol silyl ether, **4**, and CsF), unlike organometallic reagents, are not sensitive to atmospheric ^12^CO_2_. In addition, CsF can be easily dried using an azeotropic distillation method which means greater ease in handling of reagents and less stringent operating conditions for production of this clinically useful radiotracer.

### Exploration of synthesis of ^11^C-carboxylates and derivatives

The success in developing an updated method for synthesizing **[**^**11**^**C]3** triggered our interest in exploring carbon nucleophiles, generated in situ via FMDS approach, for ^11^C-carboxylation. Although FMDS method had been used to generate a variety of nucleophiles in many organic reactions (such as alkylation, allylation, alkynylation, arylation, vinylation, and cyanation), and had been broadly used to synthesize complex molecules^11^, there are only a handful of reports of directly using organosilanes for carboxylation reaction without involving any transition metal catalysts^19–29^. While organometallic reagent catalyzed carboxylation has already drawn extensive attention to radiochemistry research^30–32^, to the best of our knowledge, we have not found any reports of the use of the FMDS methodology for direct ^11^C-carboxylation. It is comprehensible that the high cost and necessity of stoichiometric amounts of organosilane reagents made this method less attractive in synthetic organic chemistry research when compared to other organometallic reagent catalyzed carboxylation methodologies^33,34^. For ^11^C-labeled radiotracer production, however, the primary costs come from cyclotron bombardment for generation of [^11^C]CO_2_, highly complex equipment-dependent automated synthesis, and elaborate quality control processes. The quantities of chemicals required for ^11^C-labeling reactions are at the micromole and milliliter level, hence the cost of reagents is in a minor consideration. Based upon these prerequisites, adopting a FMDS strategy for ^11^C-carboxylation provides many potential advantages, such as: (1) The simplicity of the whole reaction system, since only four reagents (organosilane, fluoride source, solvent, [^11^C]CO_2_) are involved in the labeling reactions and there is no organometallic catalyst, ligand(s), base, etc. needed; (2) Less precaution is needed for preparation compared with organometallic reagent based ^11^C-carboxylation methods since most organosilanes and fluoride source (such as CsF) are not sensitive to atmospheric ^12^CO_2_. This difference implies another advantage, i.e. the ^11^C-labeled PET tracers with high molar activity could be obtained under less stringent conditions; (3) The mildness of the FMDS ^11^C-labeling strategy could help us to synthesize ^11^C-carboxylic acids and their derivatives attached to compounds with diversified functional groups thereby dramatically broadening the scope of ^11^C-labeling via direct use of [^11^C]CO_2_ as a radiosynthon.

**Fig. 2:**
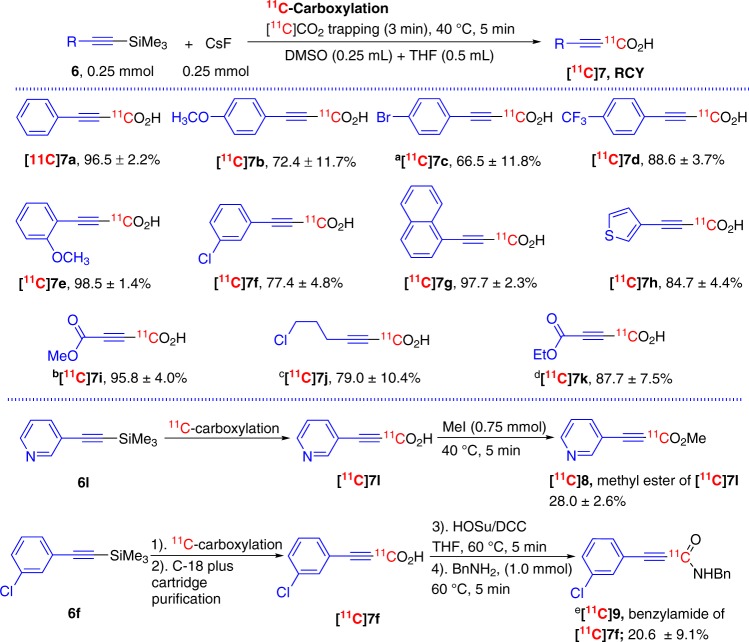
FMDS ^11^C-carboxylation with sp-hybridized carbon attached trimethylsilanes. Unless otherwise indicated, the reported values are radiochemical yields (RCY, *n* = 3) of determined by radio-HPLC analysis of the crude product and the product identities were determined by co-injection with corresponding carbon-12 standards. ^a^THF (0.5 mL) + DMF (0.25 mL) were used; ^b^Reaction was maintained at r.t., 2 min; ^c^DMSO was used as solvent; ^d^Precursor (0.029 mmol, 5 mg) and DMF (0.3 mL) were used for reactions.

We first explored ^11^C-carboxylation using alkynyltrimethylsilanes, **6**, and CsF for sp-hybridized carbanion generation^35^. After preliminary tests, it was found that a combination of THF and dimethyl sulfoxide (DMSO) worked better for these ^11^C-carboxylation reactions, which stoichiometric reagent ([^11^C]CO_2_ and [^12^C]CO_2_ together) at nano- or subnano-moles level, than DMSO alone (Fig. 2)^27^. The selected reaction conditions not only trapped [^11^C]CO_2_ efficiently (usually >90% [^11^C]CO_2_ radioactivity was retained in the reaction mixture), but also transferred it into 3-substituted propiolic-[1-^11^C]acid upon heating the reaction mixture at 40 °C for 5 min with excellent radiochemical yield (RCY, **[**^**11**^**C]7a-b, e-f**, ranging from 72.4 to 98.5%; all radiochemical yields displayed for the rest of work were determined by multiplying the radiochemical purity as determined by HPLC times isolated radioactivity divided by the starting [^11^C]CO_2_ (decay corrected), unless stated otherwise) despite the difference in functional groups (either electron withdrawing or donating) and their positions in the phenyl ring (*ortho*, *para*, or *meta*). The replacement of DMSO with DMF was less impactful for the ^11^C-carboxylation (3-(4-bromophenyl)propiolic-[1-^11^C]acid, **[**^**11**^**C]7c**, yield = 66.5 ± 11.8%). In addition, the ^11^C-carboxylation conditions were also adaptable with naphthalenyl (**6g**), 3-thienyl (**6h**), methyl, and ethyl esters (**6i** and **6k**), and chloropropyl groups (**6j**) attached ethynyltrimethylsilanes and all reagents provided the corresponding 3-substituted propiolic-[1-^11^C]acids, **[**^**11**^**C]7g-k**, with excellent yields as well. Decreasing the amount and concentration of precursor (**6k**, 0.05 mmol and 0.167 M vs **6i**, 0.25 mmol and 0.33 M) only slightly decreased the incorporation of [^11^C]CO_2_ into the desired 3-substituted propiolic-[1-^11^C]acid (yields of **[**^**11**^**C]7i** and **[**^**11**^**C]7k**, 95.8 ± 4.0% vs 87.7 ± 7.5%).

To further demonstrate the versatility of this ^11^C-carboxylation method, we explored the amenability of a quick conversion of ^11^C-carboxylic acids into their ester and amide derivatives (Fig. 2)^5^. The desired product, 3-pyridyl attached methyl [1-^11^C]propiolate, **[**^**11**^**C]9**, was formed with a 28.0 ± 2.6% yield without any optimization of reaction conditions by the ^11^C-carboxylation reaction of alkynyltrimethylsilanes, **7l**, followed with the methyl esterification by adding methyl iodide (0.75 mmol) to the same reaction vial and heating at 40 °C for 5 min. The transformation of ^11^C-carboxylic acid into carboxamide was also exemplified by smoothly converting [^11^C]carboxylic acid, **[**^**11**^**C]7f**, to its benzylamide derivative, **[**^**11**^**C]9**^5^. As an intermediate, **[**^**11**^**C]7f** was first purified using solid phase extraction (SPE) technique with a C18 plus cartridge. The radioactivity was then eluted from the cartridge using THF into a second reaction vial, and reacted with *N*-hydroxysuccinimide (HOSu) and dicyclohexylcarbodiimide (DCC) at 60 °C for 5 min. The desired benzylamide derivative, **[**^**11**^**C]9**, was formed by adding benzylamine (1.0 mmol) and heating the reaction mixture at 60 °C for 5 min with an overall yield of 20.6%.

**Fig. 3:**
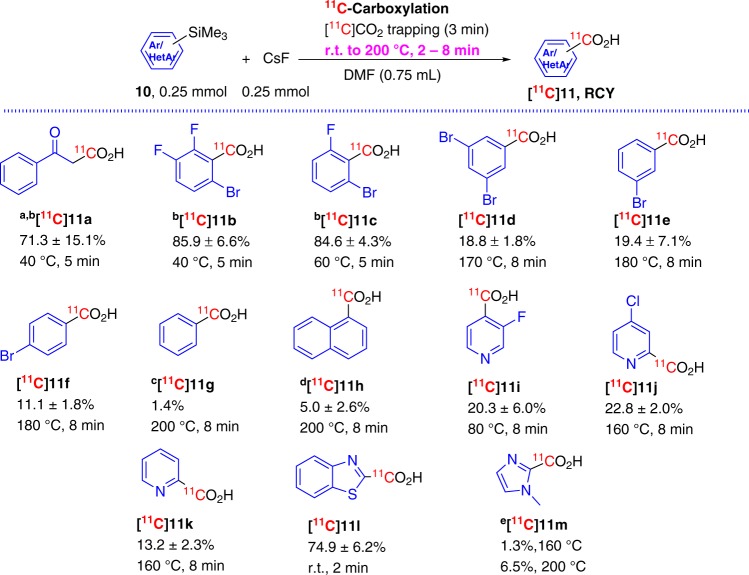
FMDS ^11^C-carboxylation with sp^2^-hybridized carbon attached trimethylsilanes. ^a^Solvent DMF (0.25 mL) + THF (0.5 mL), precursor is sp^2^-hybridized carbon attached silyl enol ether; ^b^Precursors are highly moisture sensitive; ^c^There was no detected product at 150 and 160 °C; ^d^Precursor 10 mg (0.05 mmol) in DMF (0.3 mL) (*n* = 4 for RCC); ^e^*tert*-Butyldimethylsilyl attached 1-methylimidazole was used as precursor; there was no product detected at 120 °C, 8 min for all reactions.

Upon successful addition of the ^11^C-carboxylate moiety at sp-hybridized alkynyl carbons, we immediately turned our focus to different organosilane substrates, specifically trimethylsilyl (TMS) groups attached at the sp^2^-hybridized carbon, for synthesizing ^11^C-carboxylic acids using FMDS ^11^C-carboxylation approach (Fig. 3). Unlike the synthesis of various ^11^C-propiolates, in which the reaction parameters required minor variation, the reaction temperature for the synthesis of different aryl/heteroaryl ^11^C-carboxylic acids, **[**^**11**^**C]11**, had to be modified significantly. Acetophenone enol trimethylsilyl ether, **10a**, similar to silyl enol ether **4**, displayed high reactivity and the *β*-carbonyl [^11^C]carboxylic acid, **[**^**11**^**C]11a**, was obtained with a 71.3 ± 15.1% yield under similar reaction conditions as propiolic-[1-^11^C]acids, with only a change of solvent to DMF. The di- and tri-halide substituted trimethylsilylbenzene, **10b** and **10c**, displayed high propensity to convert to the corresponding [^11^C]benzoic acids, **[**^**11**^**C]11b** and **[**^**11**^**C]11c** with excellent conversion yields. The substrate reactivity dropped significantly when the halide groups were moved from *ortho-* to *meta-* positions and when one fluorine was changed to bromine (**[**^**11**^**C]11c** vs **[**^**11**^**C]11d**, 60 °C, 5 min, 84.6 ± 4.3% vs 170 °C, 8 min, 18.8 ± 1.8%)^24^. The removal of one bromide group from *meta-* position further decreased the reactivity of the substrate and the reaction temperature had to be increased to 180 °C to maintain comparable reaction yields (**[**^**11**^**C]11e**, 19.4 ± 7.1%). The change of the bromide group from *meta-* to *para-* position further reduced reaction yields and only 11.1 ± 1.8% of the desired 4-bromo-[1-^11^C]benzoic acid, **[**^**11**^**C]11f**, was obtained under the same reaction conditions.

When trimethylsilylbenzene, **10g**, was tested, only 1.4% of desired [^11^C]benzoic acid, **[**^**11**^**C]11g**, was observed at extreme conditions (200 °C, 8 min) and no product was detected at lower reaction temperature (150 and 160 °C). The other indolent substrate, (1-naphthyl)trimethylsilane, **10h**, showed slightly better reactivity under the same conditions and **[**^**11**^**C]11h** was obtained with a 5 ± 2.6% yield at lower reactant concentration (0.167 M vs 0.33 M). A similar trend in reactivity was found when pyridyltrimethylsilanes were tested as substrates for ^11^C-carboxylation (Fig. 3). With the more electron-withdrawing group, fluoro, attached at the *ortho-* position, the substrate **10i** clearly showed higher reactivity (80 °C, 8 min, 20.3 ± 6.0% **[**^**11**^**C]11i**) than substrate **10j**, which has a less electron negative chloride atom substituted at *meta-* position of the TMS group (160 °C, 8 min, 22.8 ± 2.0% **[**^**11**^**C]11j**). Without the chlorine substitution, 2-trimethylsilylpyridine, **10k**, clearly showed weaker reactivity and the product **[**^**11**^**C]11k** was obtained in lower yields under the same reaction conditions as **[**^**11**^**C]11j** (160 °C, 8 min, 13.2 ± 2.3%). The other two heteroaryl trimethylsilanes also presented dramatically different reactivity: 2-trimethylsilylbenzothioazole, **10l**, displayed a high propensity to form **[**^**11**^**C]11l** (r.t., 2 min, 74.9 ± 6.2%). While 2-(*tert*-butyldimethylsilyl)-1-methyl-1*H*-imidazole, **10m**, exhibited low reactivity with only a 6.5% of the desired **[**^**11**^**C]11m** even when the reaction mixture was heated to 200 °C for 8 min (only 1.3% **[**^**11**^**C]11m** was detected with reaction mixture was heated to 160 °C for 8 min).

**Fig. 4:**
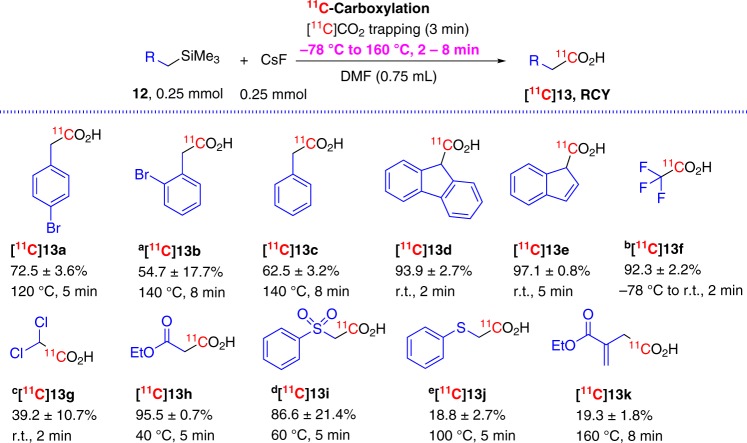
FMDS ^11^C-carboxylation with sp^3^-hybridized carbon attached trimethylsilanes. ^a^Precursor (0.05 mmol, 12 mg), DMF (0.3 mL); ^b^Reaction vial was maintained at r.t. for 2 min after [^11^C]CO_2_ collection was done at −78 °C, THF as solvent; ^c^Solvent DMF (0.25 mL) + THF (0.5 mL); ^d^Precursor is highly moisture sensitive; ^e^RCY based on *n* = 4.

Following the exploratory synthesis of various aryl and heteroaryl ^11^C-carboxylic acids, we further extended our investigation to employ FMDS strategy for ^11^C-carboxylation using organosilanes with the TMS group attached at the sp^3^-hybridized carbon (Fig. 4). Three benzylsilanes showed similar reactivity with or without bromine substituted in the aromatic ring (**12a** and **12b** vs **12c**) and these reactions (120–140 °C, 5–8 min) gave the desired [^11^C]phenyl acetic acids (**[**^**11**^**C]13a-c**) with good yields (54–72%)^28^. Remarkably, two other benzyltrimethylsilane type substrates, (9-trimethylsilyl)fluorine **12d**, and bis(1*H*-inden-1-yl)dimethylsilane **12e**, gave corresponding ^11^C-carboxylic acids, **[**^**11**^**C]13d**-**e**, with excellent yields even at ambient temperature. A large variation in RCY was seen for methyltrimethylsilanes bearing a variety of substitutions (**12f-k**, Fig. 4). Trifluoromethyl trimethylsilane **12f** displayed extremely high reactivity and ^11^C-carboxylation had to be performed using extreme conditions, i.e., the collection of [^11^C]CO_2_ was processed at −78 °C using THF as the solvent. After [^11^C]CO_2_ trapping, the reaction vessel was maintained at room temperature (r.t.) for 2 min to afford over 90% conversion of [^11^C]CO_2_ to the desired [^11^C]trifluoroacetic acid, **[**^**11**^**C]13f**. The extended reaction time (from 2 to 5 min) at r.t. was detrimental to the reaction with a reduced yield of 19%. When dichloromethyl trimethylsilane, **12g**, was tested, both the collection of [^11^C]CO_2_ and the reaction were maintained at r.t. and the reaction solvent was switched to a dual solvent system (THF/DMF, 2/1, v/v) to afford [^11^C]dichloroacetic acid, **[**^**11**^**C]13g**, with a yield of 39.2 ± 10.7% . The substrate Ethyl 3-(trimethylsilyl)acetate, **12h**, needed to be maintained at 40 °C for 5 min to provide monoethyl [1-^11^C]malonate, **[**^**11**^**C]13h**, with an excellent yield (95.5 ± 0.7%). Interestingly, phenylsulfone and phenylthio substituted methyl trimethylsilanes, **12i** and **12j**, showed quite different reactivity toward ^11^C-carboxylation. The former gave desired product, **[**^**11**^**C]13i**, with a 86.6 ± 21.4% yield at 60 °C for 5 min. The latter, however, displayed moderate reactivity and provided desired phenylthio substituted [1-^11^C]acetic acid, **[**^**11**^**C]13j**, with only a 18.8 ± 2.7% yield even when heated at 100 °C for 5 min. It was found that the allyltrimethylsilane derivative, **12k**, was less reactive, as the reaction mixture had to be heated to 160 °C for 8 min to produce the corresponding ^11^C-carboxylic acid, **[**^**11**^**C]13k**, with a 19.3 ± 1.8% yield.

The different reactivity of sp-, sp^2^-, and sp^3^-carbon attached organosilanes used in FMDS ^11^C-carboxylation reactions can be explained by the p*K*_a_ value of the conjugate acid of the fluoride-desilylation generated anionic nucleophile^11^. The carbanions generated by FMDS process can be assigned to three groups based upon the p*K*_a_ values of their conjugate acids: Group 1 (p*K*_a_ ~20–35) usually form stabilized anions and display high reactivity. Trimethylsilanes (**4**, **6**, **10a-c**, **10l**, **12d-i**) all belong to this category. Group 2 (p*K*_a_ ~35–45) includes carbanions attaching one weakly anion stabilizing group such as allyl, benzyl, and heterocyclic benzyl analogs, and phenylthiomethyl. Aryl groups bearing one or more electron-withdrawing substituents formed carbanions also can be included in Group 2. Trimethylsilane substrates (**10d-f**, **10i-k**, **12a-c**, and **12j-k**) could be categorized in this group. Last, group 3 contains very weakly stabilized anions (p*K*_a_ values of their conjugate acids is usually >45). The examples in our study, such as **10g-h** and **10m**, can be assigned to this category and usually gave minimal (<10% RCY or non-detectable) desired ^11^C-carboxylic acids.

### Extension of FMDS ^11^C-labeling approach for ^11^C-methylation

Bolstered by the success of using FMDS ^11^C-carboxylation strategy to synthesize various ^11^C-carboxylic acids and their derivatives, we next attempted the translation of this radiolabeling approach for ^11^C-methylation. Since methyl iodide (CH_3_I) and methyl triflate (CH_3_OTf) are much better electrophiles compared with the chemically inert CO_2_, we assumed that FMDS ^11^C-carboxylation approach should be easily adaptable for ^11^C-methylation and the following results supported our speculation. We started exploration of ^11^C-methylation using FMDS approach, again, from the protocol reported by Noyori in 1983^14^. After screening various experimental conditions (Table 1), it was found that the combination of [^11^C]CH_3_I/CsF/DMF (Table 1, entry 4) was the optimal choice compared with the more reactive [^11^C] CH_3_OTf (Table 1, entry 3) or the more labile fluoride source TASF (Table 1, entry 1 and 2)^36^. The silyl enol ether, **4**, converted to the desired [4-^11^C]butanone, **[**^**11**^**C]14**, with a 43.7 ± 16.4% yield while maintaining the reaction at r.t. for 2 min after the collection of [^11^C]CH_3_I to the reaction vial (Table 1, entry 4).

**Table 1:** FMDS ^11^C-methylation reaction conditions screening.


Entry	Fluoride source	[^11^C]Methylation agent and solvent(s)	RCY (%)
**1**	TASF	[^11^C]CH_3_OTf, THF/DCM (0.5 mL/0.25 mL)	12.7%
**2**	TASF	[^11^C]CH_3_I, THF/DCM (0.5 mL/0.25 mL)	40.7%
**3**	CsF	[^11^C]CH_3_OTf, DMF (0.75 mL)	26.6%
**4**	CsF	[^11^C]CH_3_I, DMF (0.75 mL)	**43.7 ± 16.4%,** ***n* = 3**

**Fig. 5:**
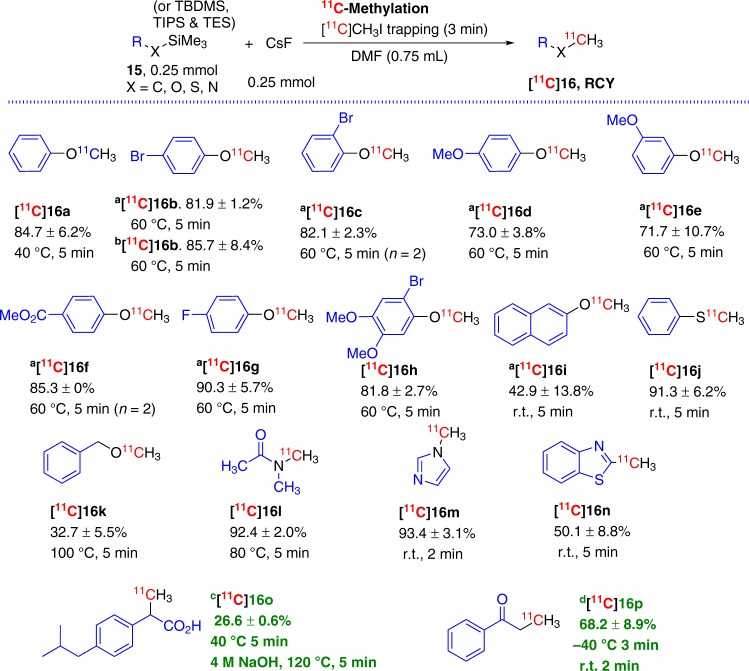
FMDS ^11^C-methylation. ^a^*tert*-Butyldimethylsilyl (TBDMS) group attached compounds as labeling precursors; ^b^Triisopropylsilyl (TIPS) group attached compound as labeling precursor. ^c^Triethylsilyl (TES) group attached ethyl ester as labeling precursor; [^11^C]CH_3_I was collected at r.t., 3 min, then 40 °C, 2 min; Next saponification: 4 M NaOH, 120 °C, 5 min; products were purified by semi-prep HPLC. ^d^TASF was used as fluoride source, THF (0.5 mL)/DCM (0.25 mL) was used as solvent, [^11^C]CH_3_I was collected at −40 °C, 3 min, reaction mixture was left at r.t., 2 min, products were purified by semi-prep HPLC; starting radioactivity of [^11^C]CH_3_I: 7.3 ± 2.67 GBq; total synthesis time: 37.7 ± 1.2 min; radiochemical purity (RCP) > 99% and *A*_m_: 29.7 ± 10.9 GBq/µmol (end of bombardment, EOB).

With the initial CsF/DMF/[^11^C]CH_3_I conditions defined, we next evaluated the scope of FMDS ^11^C-methylation using a variety of organosilane reagents (Fig. 5). All phenoxysilane substrates that were tested gave good to excellent ^11^C-methylation yields under mild reaction conditions despite the variation of electron -donating (**15d-e**), -neutral (**15a**) or -withdrawing (**15b**, **15b′,** **15c**, **15f-g**) groups attached at the phenyl ring. The change of *tert*-butyldimethylsilyl (TBDMS) to triisopropylsilyl (TIPS) (**15b** vs **15b′**) had minimal impact upon the labeling yields (81.9 ± 1.2% **[**^**11**^**C]16b** vs 85.7 ± 8.4% **[**^**11**^**C]16b**). Even the precursor, **15h**, with a phenyl ring that possessed three functional groups afforded excellent reaction yields (81.8 ± 2.7% **[**^**11**^**C]16h**). The naphthyl group containing compound *tert*-butyldimethyl(naphthalen-2-yloxy)silane, **15i**, was labile under the reaction conditions. The reactions formed large amount of radioactive by-products even with reactions at r.t. for 5 min and only gave a moderate yield (42.9 ± 13.8% **[**^**11**^**C]16i**). The trimethyl(phenylthio)silane, **15j**, on the contrary, provided excellent yields (91.3 ± 6.2% **[**^**11**^**C]16j**) under the same reaction conditions. Benzyloxytrimethylsilane, **15k**, displayed moderate reactivity and the radiolabeling yield only reached 32.7 ± 5.5% (**[**^**11**^**C]16k**) when the reaction mixture was heated at 100 °C for 5 min. Both *N*-trimethylsilyl substituted substrates gave excellent labeling yields (92.4 ± 2.0% **[**^**11**^**C]16l** and 93.4 ± 3.1% **[**^**11**^**C]16m**) despite quite different reaction conditions (80 °C vs r.t.). The substrate **15n** (which is the same as **10m**) displayed excellent reactivity and labeling reactions were performed at r.t. for 5 min to afford 2-(methyl-^11^C)benzo[d]thiazole **[**^**11**^**C]16n** with a 50.1 ± 8.8% yield. Using the semi-prep HPLC for purification, [3-^11^C]ibuprofen, **[**^**11**^**C]16o**, was obtained with a 26.6 ± 0.6% yield (based upon the product separated from semi-prep HPLC) from a triethylsilyl (TES) group attached compound **15o** after ^11^C-methylation at 40 °C for 5 min and saponification by 4 M NaOH. Similar to the synthesis process of [4-^11^C]butanone **[**^**11**^**C]14**, Acetophenone enol trimethylsilyl ether, **15p** (same as **10a**), was employed as the precursor and TASF as the fluoride reagent, the desired product [3-^11^C]propiophenone, **[**^**11**^**C]16p**, was synthesized with a 68.2 ± 8.9% yield (based upon the product separated from semi-prep HPLC).

The significance of our FMDS ^11^C-labeling method was not only demonstrated by the successful synthesis of thirty seven different ^11^C-carboxylic acids, but also exemplified by the in situ quick and smooth conversion of the ^11^C-carboxylic acids to their methyl ester and benzylamide derivatives. Additionally, we have readily extended this method to the ^11^C-methylation process. The robustness of FMDS ^11^C-methylation approach was illustrated by fast and facile synthesis of seventeen different ^11^C-methylated compounds with diversified structures by selectively attaching [^11^C]CH_3_- group to the specific position (oxygen, sulfur, nitrogen and carbon atoms) of these molecules.

### Exploratory synthesis of three radiotracers

**Fig. 6:**
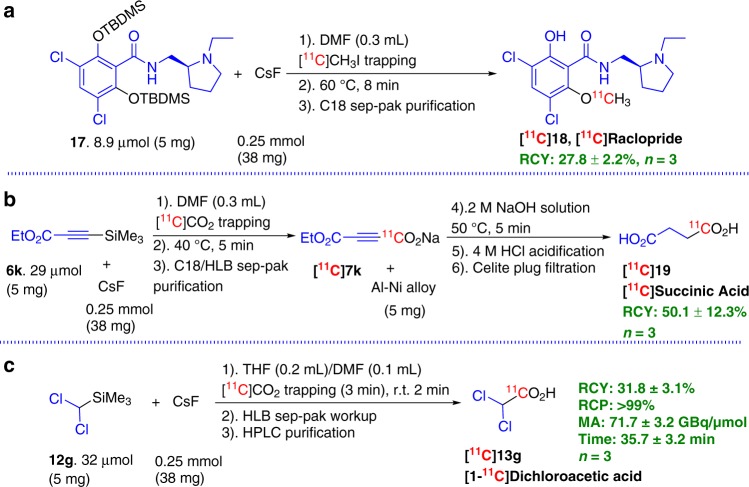
Practical synthesis of three radiotracers via FMDS ^11^C-labeling approach. **a** [^11^C]raclopride synthesis via ^11^C-methylation of dual TBDMS attached precursor **17**; **b** [^11^C]succinic acid synthesis via ^11^C-carboxylation of alkylsilane **6k** and Al-Ni alloy reduction of intermediate [^**11**^**C**]**7**; **c** [^11^C]dichloroacetic acid synthesis via ^11^C-carboxylation of alkylsilane **12g**.

As a final demonstration of the strength of FMDS ^11^C-labeling method and its feasibility for practical radiotracer production, in addition to the aforementioned cGMP-compliant production of [^11^C]AcAc (**[**^**11**^**C]3**), we further explored the practical synthesis of three ^11^C-labeled organic molecules, ([^11^C]raclopride **[**^**11**^**C]18**, [^11^C]succinic acid **[**^**11**^**C]19**, and [^11^C]dichloroacetic acid **[**^**11**^**C]13g**), which are of biological and clinical interest but synthetically challenging molecules. To facilitate the regular production and the purification process of these tracers, a lower amount of organosilane substrate (5 mg) was used for the synthesis of these radiotracers. Raclopride is a dopamine D_2_/D_3_ receptor antagonist and its carbon-11 labeled isotopologue, [^11^C]raclopride (**[**^**11**^**C]18**), is commonly produced as a PET tracer in many PET radiochemistry facilities (Fig. 6a) for evaluating the density or occupancy of the D_2_-dopamine receptor. Although the [^11^C]ethyl iodide based *N*-ethylation method had been reported before for synthesizing this tracer at its early development stage^37^, the phenoxy group ^11^C-methylation method using [^11^C]CH_3_I/[^11^C]CH_3_OTf as the radiosynthon has become the regular synthesis method for routine production of this clinically important tracer^38,39^, although the ^11^C-carbonylation method was also intensively investigated^40^. In this research, FMDS ^11^C-methylation approach was successfully implemented to synthesize this tracer. Using dual TBDMS attached precursor **17**, the ^11^C-labeling and in situ deprotection of TBDMS group produced [^11^C]raclopride, **[**^**11**^**C]18**, with 27.8 ± 2.2% yield after purification through the solid phase extraction (SPE) method, which is lower than reference reported methods^37–40^. Further optimizations in the future, such as changing TBDMS group to more labile TMS or TES groups and therefore decreasing ^11^C-methylation reaction temperature, should help us to improve the production yield.

Succinic acid is an endogenous dicarboxylic acid which had been identified as an oncometabolite^41^. Carbon-11 labeled succinic acid may have the potential to map the metabolic process of cancer cells and help cancer diagnosis, staging and re-staging^41,42^. Carbon-11 labeled dicarboxylic acids were synthesized by nucleophilic ^11^C-cyanation and followed by basic hydrolysis^43^. However, this method requires an additional expensive and complex automated [^11^C]HCN production module. In this study, the 4-ethoxy-4-oxobut-2-ynoic-[1-^11^C]acid, **[**^**11**^**C]7k**, was first synthesized by FMDS ^11^C-carboxylation method (described in Fig. 2). After two quick and efficient SPE purifications to remove unreacted [^11^C]CO_2_, CsF and solvent, etc., the ^11^C-intermediate, **[**^**11**^**C]7k**, was eluted to the second reaction vial and mixed with Al-Ni alloy in basic solution for alkyne reduction (50 °C, 5 min)^44^. The desired [^11^C]succinic acid, **[**^**11**^**C]19**, was obtained with a 50.1 ± 12.3% yield (Fig. 6b) following acidification and filtration through a celite plug.

Dichloroacetic acid (DCAA) is a small halogenated acetic acid analog, it can affect cancer cell metabolism and antagonize its growth by inhibiting mitochondrial pyruvate dehydrogenase kinases. DCAA has been investigated clinically for the treatment of various cancers (including brain, colon, breast, colorectal, and skin cancers). Its therapeutic applications and intriguing pharmacological properties have attracted a lot of attention from medical researchers^45,46^. The combination of PET imaging technique and ^11^C-labeled DCAA, potentially, could facilitate researchers’ understanding of in vivo metabolic processes and pharmacokinetics of this low-price, low toxicity and promising cancer drug candidate. Employing FMDS ^11^C-carboxylation protocol for synthesis of **[**^**11**^**C]13g** (Fig. 4) followed by a quick SPE workup and semi-prep HPLC purification process, with only 5 min cyclotron beam time (producing 11.8 ± 4.2 GBq starting [^11^C]CO_2_ radioactivity), we obtained 1.1 GBq of the desired [^11^C]DCAA, **[**^**11**^**C]13g**, (RCY, 31.8 ± 3.1%) with over 99% radiochemical purity in a 35.7 ± 3.2 min synthesis process (Fig. 6c). The product had *A*_m_ of 71.7 ± 18.1 GBq/µmol (EOB), which should be satisfactory for oncological imaging studies in humans.

## Discussion

The development of a robust FMDS ^11^C-labeling strategy, although still in its early stages, opens up the potential for the synthesis of ^11^C-labeled organic carboxylic acids and their derivatives directly using [^11^C]CO_2_. The simplicity, high degree of reproducibility, and broad scope of the transformation of this approach, as showcased by the ^11^C-labeling of various biologically interesting organic molecules, makes it a promising approach in radiotracer chemistry. In addition, the expansion of FMDS ^11^C-labeling method using [^11^C]CH_3_I as a radiosynthon is a beneficial supplement to current [^11^C]CH_3_I/[^11^C]CH_3_OTf based radiochemistry. The further extension and application of FMDS ^11^C-labeling method to solve several long-standing problems in ^11^C-radiotracer chemistry will be reported in due course.

## Methods

### General procedures of [^11^C]CO_2_ production

[^11^C]CO_2_ was generated by bombarding *N*_2_ gas (360 psi 99.9999% pure *N*_2_ doped with 0.5% O_2_) via the ^14^*N*(p,α)^11^C nuclear reaction using a EBCO TR-19/9 cyclotron. General bombardment conditions: 2–40 min beam time with 25 µA current (3.7–44.4 GBq; 100–1200 mCi). After the bombardment, target gas containing radioactivity was released and delivered to a home-made automated [^11^C]CO_2_ purification box for controlled trap and release of [^11^C]CO_2_, where the [^11^C]CO_2_ was first trapped by a molecular sieve (MS) furnace at room temperature (200 mg molecular sieve 13X, 100/120 mesh, SUPELCO). Next, the furnace is heated to 190 °C and [^11^C]CO_2_ was released and delivered to the reaction vial using helium flow (10 mL/min). Once the radioactivity collected in the reaction vial plateaued, the delivery was stopped and [^11^C]CO_2_ production and collection was done. It took 3–4 min from end of bombardment (EOB) to end of the collection of [^11^C]CO_2_ in the reaction vial.

### General procedures of FMDS ^11^C-carboxylation reaction

Once the [^11^C]CO_2_ was ready, the [^11^C]CO_2_ delivery line with a 4-inch needle was inserted into a reaction vial containing the anhydrous fluoride reagent and solvent reaction mixture. The vial was equipped with outlet line (an ascarite trap was attached at the end for trapping escaped [^11^C]CO_2_). After confirmation of a stable helium flow (10 mL/min), the organosilane precursor was immediately added. The reaction vial was placed in a dose calibrator for measuring the collected radioactivity. Once the increase of radioactivity in the reaction vial plateaued, the [^11^C]CO_2_ delivery line and outlet line were removed immediately. The total activity trapped in the reaction vial was checked again, and recorded as starting radioactivity **A**_**0**_. When dimethylformamide (DMF), dimethylacetamide (DMA) and dimethyl sulfoxide (DMSO), as well as its mixture with tetrahydrofuran (THF) (1/1, v/v) were used as solvents for reactions, the trapping of radioactivity [^11^C]CO_2_ was generally efficient and the escaped [^11^C]CO_2_ collected by the ascarite trap at the outlet of the reaction vial was usually less than 10%. When only THF or THF/dichloromethane (DCM) (3/1, v/v) was used as the reaction solvent, a cooling bath was used (−70–0 °C) to keep reaction vial cool and escaped radioactivity was also minimal (< 10%). After maintaining the reaction mixture at the desired temperature with stirring for a specific time frame (normally 2–10 min), the reaction was quenched by addition of an acidic solution (1 mL, CH_3_CN/H_2_O/formic acid, 90/9/1; or 0.1 M HCl aqueous solution). The unreacted [^11^C]CO_2_ was purged from the reaction vial with a gentle stream of helium (2–5 psi) and trapped in a second ascarite trap. When the radioactivity collected in this ascarite trap became constant (**A**_**leak**_), the remaining radioactivity in the reaction vial was again measured and recorded as **A**_**left**_. After that, a small portion of solution was removed from reaction vial (0.1–0.2 mL) and diluted in a sample vial pre-loaded with an acidic solution (1 mL, CH_3_CN/1% formic acid, 90/10). Next, an analytical sample, which was a mixture of an aliquot of sample solution (usually 10 µL) and a product standard solution (usually 10 µL, 1 mg/mL solution), was injected into HPLC for analysis. The percentage of the radio-peak in the radio-chromatogram coincident with product reference UV peak was regarded as radiochemical purity (RCP). The radiochemical yield (RCY) was calculated by the equation [(RCP × **A**_**left**_)/**A**_**0**_] × 100%. The **A**_**0**_ and **A**_**left**_ were decay corrected values. If the reaction mixture was submitted for the purification process (solid phase extraction, anion/cation resins exchange method, semi-prep HPLC, or the combination of two of these methods), the total amount of radioactivity of purified product was recorded as **A**_**prod**_. The radiochemical yield (RCY, decay corrected) was calculated as (**A**_**prod**_/**A**_**0**_) × 100%. Total synthesis times were calculated from time point of finished collection of [^11^C]CO_2_ to the end of radiotracer purification process.

### [^11^C]CH_3_I/[^11^C]CH_3_OTf production

[^11^C]CO_2_ was generated by bombarding *N*_2_ gas (360 psi 99.9999% pure *N*_2_ doped with 0.5% O_2_) via the ^14^*N*(p,α)^11^C nuclear reaction using a EBCO TR-19/9 cyclotron. General bombardment conditions for [^11^C]CH_3_I/ [^11^C]CH_3_OTf production: 5–40 min beam time with 25 µA current. After the bombardment, target gas containing radioactivity was released and delivered to a GE TRACERlab FXC automatic synthesizer to convert [^11^C]CO_2_ to [^11^C]CH_3_I or [^11^C]CH_3_OTf. It took 16–18 min from end of bombardment (EOB) to finish the collection of [^11^C]CH_3_I or [^11^C]CH_3_OTf radioactivity in the reaction vial.

### General procedures of FMDS ^11^C-methylation reaction

FMDS ^11^C-methylation experimental process was the same as described for FMDS ^11^C-carboxylation reactions except for that there was no unreacted [^11^C]CO_2_ exclusion process since unreacted [^11^C]CH_3_I or by-product [^11^C]CH_3_OH dissolved well in the reaction mixture and there was no leakage of radioactivity detected during the sampling process after the ^11^C-methylation reaction. When [^11^C]CH_3_I or [^11^C]CH_3_OTf was ready, the delivery line with a 4-inch needle was inserted into a reaction vial containing the anhydrous fluoride reagent and solvent reaction mixture equipped with outlet line. The organosilane precursor was immediately added. The reaction vial was placed in a dose calibrator for measuring the collected radioactivity. Once the increase of radioactivity in the reaction vial plateaued, the [^11^C]CH_3_I or [^11^C]CH_3_OTf delivery line and airflow outlet line were removed. The total activity trapped in the reaction vial was checked again as starting radioactivity **A**_**0**_. After keeping the reaction mixture stirring under desired temperature for a specific time (generally 2–10 min), the reaction mixture was measured again to have total radioactivity A_0_′ (The value of **A**_**0**_**′** was supposed to equal as **A**_**0**_ after decay correction since unreacted [^11^C]CH_3_I or by-product [^11^C]CH_3_OH dissolved well in the reaction mixture. However, it was found that the value of **A**_**0**_**′** was slightly less than the value of **A**_**0**_ after decay correction in some experiments. It is most likely because of the small leakage of [^11^C]CH_3_I or by-product [^11^C]CH_3_OH from reaction vial.) A small portion of reaction mixture (~0.1 mL) was removed and diluted with an acidic solution (CH_3_CN/1% formic acid, 90/10) in a septa cap sealed glass vial; the radioactivity of this sample was counted and recorded. Next, an analytical sample, which was a mixture of an aliquot of sample solution (usually 10 µL) and a product standard solution (usually 10 µL, 1mg/mL solution), was analyzed by analytical HPLC. The percentage of radio-peak in radio-chromatogram coincident with the product reference UV peak was regarded as radiochemical purity (RCP) and also as radiochemical yield (RCY, decay corrected) if **A**_**0**_**′** = **A**_**0**_, or RCY = RCP × (**A**_**0**_**′/A**_**0**_) at the case of **A**_**0**_**′** < **A**_**0**_. If the reaction mixture was submitted for the purification process (solid phase extraction, semi-prep HPLC, anion/cation resins exchange method, or the combination of two of these methods), the total amount of radioactivity of purified product was recorded as **A**_**prod**_. The radiochemical yield (RCY, decay corrected) was calculated as (**A**_**prod**_/**A**_**0**_) × 100%. Total synthesis times were calculated from EOB to the end of radioactive product collection after the purification.

### Molar activity (*A*_m_)

*A*_m_ values, decay corrected back to EOB and recorded in GBq/µmol, were determined from the carbon-11 activity in the HPLC product peak and the mass of compound.

## Supplementary information

Supplementary Information

## Data Availability

Complete experimental procedures and compound characterization data are available in the Supplementary Information, or from the corresponding author upon request.
