# Supplementary material for: A general 11C-labeling approach enabled by fluoride-mediated desilylation of organosilanes
Source: Nat Commun. 2020 Apr 8;11:1736. doi: 10.1038/s41467-020-15556-7 (PMC7142131; doi:10.1038/s41467-020-15556-7)
Supplement: Supplementary file 1 — Supplementary Information [file 41467_2020_15556_MOESM1_ESM.pdf]

Supplementary Information for

A general ^{11}C-labeling approach enabled by fluoride-mediated desilylation of organosilanes

Qu and Hu, et al.

Supplementary Methods

General Chemistry Experimental Information

All materials were obtained from commercial sources and used as received unless otherwise noted. All reactions described below were carried out in dried glassware. Purifications were performed using silica chromatography on VWR® High Purity Silica Gel 60 Å or flash chromatography using a CombiFlash Rf+ (Teledyne Isco). Mass determinations were performed by LC-MS analysis using a Waters ACQUITY UPLC@ coupled to a Waters SQ Deterctor 2. High resolution mass data were acquired by an Orbitrap Q Exactive (Thermo Fisher Scientific) under positive mode. Resolution was 140,000. NMR analyses were performed using a Bruker Avance III 500 MHz spectrometer in the NMR laboratory at the Weill Cornell Medicine. All silyl precursors and standards used for ^{11}C-labeling were purchased from commercial sources, or synthesized based on reported procedures (see below).

Synthesis of carboxylation and methylation standards

3-(2-Methoxyphenyl)-2-propynoic acid (^{12}C standard **7e**)

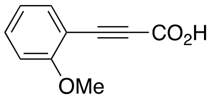

The title compound was prepared according to literature method (*1*):

To a dried and CO_2 (balloon) infused Schlenk-flask, equipped with a magnetic stirrer and a septum, CsF (30 mg, 0.20 mmol) and DMSO (1.0 mL) was added. ((2-Methoxyphenyl)ethynyl)trimethylsilane (41 mg, 0.20 mmol) was added to the same flask and the reaction was stirred at room temperature (r.t.) for overnight. The reaction mixture was diluted with water (15 mL) and extracted with CH_2Cl_2 (15 mL) twice. The aqueous layer was acidified (pH > 1) with aqueous HCl (4 M) at 0 ºC and then extracted with ethyl acetate (20 mL x 3). The combined organic layers were dried over Na_2SO_4. The solvent was removed under vacuum to afford the title product as a white solid (26 mg, 75%). The practical yield of product (67.3%) was calculated based upon the integration values of proton NMR (The NMR spectra showed that the final product contained a small amount of solvent DMSO). ^{1}H NMR (500 MHz, $CDCl_3$): δ = 7.54 (d, *J* = 7.5 Hz, 1H), 7.43 (t, *J* = 7.5 Hz, 1H), 6.96 (t, *J* = 7.5 Hz, 1H), 6.92 (d, *J* = 8.5 Hz, 1H), 3.91 (s, 3H). ^{13}C NMR (125 MHz, $CDCl_3$): δ 161.8, 157.5, 135.2, 132.7, 120.6, 110.9, 108.6, 85.4, 84.2, 55.9, 39.8. ESI(-) = 131.0 (M-CO2H)$^-$; Calculated mass: 176.0. ^{1}H and ^{13}C NMR data matched those previously reported results (*1*).

6-Chlorohex-2-ynoic acid (^{12}C standard **7j**)

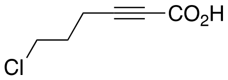

The title compound was prepared following the above procedure using CsF (38 mg, 0.25 mmol), (5-chloropent-1-yn-1-yl)trimethylsilane (44 mg, 0.25 mmol) and DMSO (0.50 mL). Product was obtained as a colorless liquid (24 mg, 65% yield). ^{1}H NMR (500 MHz, $CDCl_3$): δ = 8.63 (brs, 1H), 3.65 (t, *J* = 7.0 Hz, 2H), 2.54 (t, *J* = 7.0 Hz, 2H), 2.03 (A_2B_2, t, 2 H). ^{13}C NMR (125 MHz, $CDCl_3$): δ 155.6, 87.4, 74.2, 43.3, 30.3, 16.1. ESI(+) = 147.0 $(M+H)^+$. Calculated mass: 146.0. ^{1}H and ^{13}C NMR data matched with previously reported results (*2*).

<u>4-Methoxy-4-oxobut-2-ynoic acid (^{12}C standard **7i**)</u>

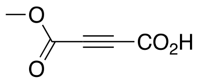

The title compound was prepared according to literature method (*3, 4*):

To a dried Schlenk-flask under nitrogen, equipped with a magnetic stirrer and a septum, methyl propiolate (0.84 mL, 10 mmol) was added in 25 mL dry THF and cooled to -78 ºC. *n*-BuLi (2.5 M in hexane, 4.0 mL, 10 mmol) was added dropwise via a syringe. After being maintained at -78 ºC for 30 min, CO_2 was infused into reaction mixture by purging the flask with CO_2 (balloon) three times. The reaction mixture was slowly warmed up to r.t. and maintained at r.t. for overnight. The solvent was removed in *vacuo*. Water (30 mL) was added and the mixture was extracted with hexane (30 mL x 2). The aqueous layer was cooled with an ice water bath and acidified with aqueous HCl (2 M, 20 mL), and then extracted with ethyl acetate (20 mL x 3). The combined organic layers were washed with water and brine, dried over Na_2SO_4 and filtered. The solvent was removed under vacuum. The residue was purified via flash chromatography (DCM : MeOH = 90 : 10) to provide the product (0.48 g, 37%) as a slightly brown liquid. Because the product owes low boiling point and the vacuum drying method could not entirely remove solvent (MeOH and acetone) from product. We did see the contaminated MeOH and acetone in NMR spectra in final product and the practical yield of product was 35.1% (calculated based upon the integration values of proton NMR). ^{1}H NMR (500 MHz, $CDCl_3$): δ = 7.14 (brs, 1H), 3.87 (s, 3H). ^{13}C NMR (125 MHz, $CDCl_3$): δ = 154.1, 152.1, 76.1, 74.3, 53.6. ESI (+) = 129.0 $(M+H)^+$; Calculated mass: 128.0. ^{1}H and ^{13}C NMR data matched those previously reported results (*3, 4*).

<u>4-Ethoxy-4-oxobut-2-ynoic acid (or monoethyl acetylenedicarboxylate) (^{12}C standard **7k**)</u>

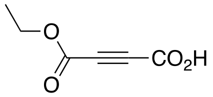

The title compound was prepared according to literature method (*5*):

To a dried and CO_2 (balloon) infused Schlenk-flask, equipped with a magnetic stirrer and a septum, Ag_2CO_3 (28 mg, 0.10 mmol), Cs_2CO_3 (0.49 g, 1.50 mmol), ethyl propiolate (98 mg, 1.0 mmol) and DMF (5 mL) were added, respectively. The reaction mixture was stirred at 50 ºC for overnight. It was diluted with water (10 mL) and extracted with CH_2Cl_2 (3 x 10 mL). The aqueous layer was cooled with an ice water bath and it was acidified (pH

>1) with concentrated HCl, and then extracted with ethyl acetate (3 x 10 mL). The combined organic layers were dried over Na_2SO_4. The solvent was removed under vacuum to afford the title product (95 mg, 67%) as a yellow liquid. Because the product has low boiling point and the vacuum drying method could not entirely remove reaction solvent DMF from product. We did see the contaminated DMF in NMR spectra in final product and the practical yield of product was 24% (calculated based upon the integration values of proton NMR). ^{1}H NMR (500 MHz, $CDCl_3$): δ = 10.33 (brs, 1H), 4.30 (q, *J* = 7.0 Hz, 2H), 1.34 (t, *J* = 7.0 Hz, 3H). ^{13}C NMR (125 MHz, $CDCl_3$): δ 153.4, 152.0, 75.3, 75.0, 63.0, 13.9. ESI (+) = 143.0 $(M+H)^+$; ESI (-) = 141.1 $(M-H)^-$; Calculated mass: 142.0. ^{1}H and ^{13}C NMR data matched those previously reported results (*5*).

N-Benzyl-3-(3-chlorophenyl)propiolamide (^{12}C standard **9**)

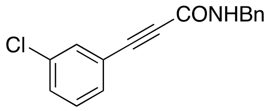

The title compound was prepared according to literature method (*6*):

To a two-neck round-bottom flask (15 mL) equipped with a magnetic stirrer and a septum, 3-(3-chlorophenyl)propiolic acid (12 mg, 66 µmol), N-hydroxysuccinimide (HOSu, 9 mg, 80 µmol, 1.2 eq.) and THF (2.0 mL), were added, respectively. Dicyclohexylcarbodiimide (DCC, 17 mg, 80 µmol, 1.2 eq.) was added and the reaction mixture was stirred at r.t. for 30 min. Benzylamine (9 mg, 80 µmol, 1.2 eq., pre-dissolved in 0.5 mL THF) was added in one portion and the reaction was stirred at r.t. for overnight. The precipitate was filtered off, the organic filtrate was concentrated in *vacuo* and the residue was submitted to purification by flash chromatography (EtOAc : hexane = 1 : 4) to give the desired amide product as a white solid (13 mg, 73%). ^{1}H NMR (500 MHz, $CDCl_3$): δ 7.50 (s, 0.55H), 7.48 (s, 0.35H), 7.45-7.25 (m, 8H)(it is scaled as 9H on the spectrum because $CDCl_3$ residue overlaps in this area), 6.27 (brs, 0.89H), 6.13 (brs, 0.10H), 4.69 (d, *J* = 6.5 Hz, 0.16H), 1.33 (t, *J* = 6.0 Hz, 1.85H). ^{13}C NMR (125 MHz, $CDCl_3$): δ 156.0, 153.0, 137.4, 137.3, 134.6, 132.4, 130.9, 130.8, 130.6, 130.1, 130.0, 129.2, 129.1, 128.2, 128.1, 127.4, 122.1, 121.9, 83.8, 83.5, 47.6, 44.2. ESI (+) = 270.1 $(M+H)^+$. Calculated mass: 269.1.

Preparation of silyl precursors for radiolabeling

(4-Bromophenoxy)(*tert*-butyl)dimethylsilane (**15b**)

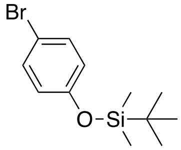

The title compound was prepared according to literature method (*6*):

To a two-neck round-bottom flask (100 mL) equipped with a magnetic stirrer and a septum, 4-bromophenol (1.73 g, 10 mmol), CH_2Cl_2 (25 mL), Et_3N (1.70 mL, 1.21 g, 12 mmol) and TBDMSCl (1.66 g, 11 mmol), were added

respectively. The reaction mixture was stirred vigorously at r.t. for overnight. After the reaction was quenched with water (20 mL), the reaction mixture was extracted with CH_2Cl_2 (20 mL). The separated organic phase was washed with water (20 mL x 2) and dried with Na_2SO_4. After evaporation in *vacuo*, the oily residue was purified by flash chromatography with hexane to give a colorless oil as the desired product (2.28 g, 79.4% yield). ^{1}H NMR (500 MHz, $CDCl_3$): δ 7.33 (d, J = 8.5 Hz, 2H), 6.73 (d, J = 8.5 Hz, 2H), 0.99 (s, 9H), 0.20 (s, 6H). ^{13}C NMR (125 MHz, $CDCl_3$): δ 154.9, 132.3, 121.9, 113.6, 25.6, 18.2, -4.5. ^{1}H and ^{13}C NMR data matched those previously reported results (*7*).

(2-Bromophenoxy)(*tert*-butyl)dimethylsilane (**15c**)

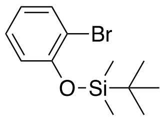

The title compound was prepared according to literature method (*8*):

To a two-neck round-bottom flask (100 mL) equipped with a magnetic stirrer and a septum, 2-bromophenol (1.73 g, 10 mmol), CH_2Cl_2 (25 mL), imidazole (0.82 g, 12 mmol) and TBDMSCl (1.81 g, 12 mmol), were added respectively. After being maintained at r.t. with vigorous stirring for overnight, the reaction was quenched with water (20 mL) and the reaction mixture was extracted with CH_2Cl_2 (20 mL). The organic phase was separated, washed with water (20 mL x 2) and dried with Na_2SO_4. After evaporation in *vacuo*, the oily residue was purified by flash chromatography with hexane to give a colorless oil as the desired product (2.58 g, 90.0% yield). ^{1}H NMR (500 MHz, $CDCl_3$): δ = 7.53 (dd, *J* = 8.0, 1.3 Hz, 1 H), 7.18 (td, *J* = 7.5, 1.4 Hz 1H), 6.89 (d, *J* = 8.0 Hz 1H), 6.83 (d, *J* = 7.5 Hz 1H), 1.07 (s, 9 H), 0.27 (s, 6 H). ^{13}C NMR (125 MHz, $CDCl_3$): δ = 152.6, 133.4, 128.2, 122.4, 120.3, 115.4, 25.8, 18.4, -4.2. ^{1}H and ^{13}C NMR data matched those previously reported results.

tert-Butyl(4-methoxyphenoxy)dimethylsilane (**15d**)

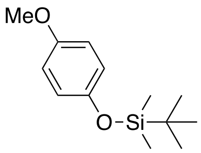

The title compound was prepared following the above procedure using 4-methoxyphenol (1.24 g, 10 mmol), imidazole (0.82 g, 12 mmol), TBDMSCl (1.81 g, 12 mmol), and CH_2Cl_2 (25 mL). The product was obtained as a colorless liquid (2.29 g, 96% yield). ^{1}H NMR (500 MHz, $CDCl_3$): δ 6.78 (s, 4H), 3.77 (s, 3H), 0.99 (s, 9H), 0.18 (s, 6H). ^{13}C NMR (125 MHz, $CDCl_3$) δ 154.1, 149.4, 120.6, 114.5, 55.6, 25.7, 18.2, -4.5. ^{1}H and ^{13}C NMR data matched those previously reported results (*9*).

tert-Butyl(3-methoxyphenoxy)dimethylsilane (**15e**)

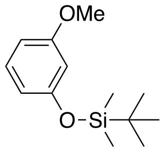

The title compound was prepared following the above procedure using 3-methoxyphenol (1.24 g, 10 mmol), imidazole (0.82 g, 12 mmol), TBDMSCl (1.81 g, 12 mmol), and CH_2Cl_2 (25 mL). The product was obtained as a yellow liquid (2.14 g, 93% yield). ^{1}H NMR (500 MHz, $CDCl_3$): δ 7.15 (t, *J* = 8.0 Hz, 1H), 6.53 (d, *J* = 8.0 Hz, 1H), 6.46 (d, *J* = 8.0 Hz, 1H), 6.42 (s, 1H), 3.79 (s, 3H), 1.00 (s, 9H), 0.22 (s, 6H). ^{13}C NMR (125 MHz, $CDCl_3$): δ 160.7, 156.9, 129.7, 112.6, 106.8, 106.3, 55.2, 25.7, 18.2, -4.4. ^{1}H and ^{13}C NMR data matched those previously reported results (*10*).

Methyl 4-((*tert*-butyldimethylsilyl)oxy)benzoate (**15f**)

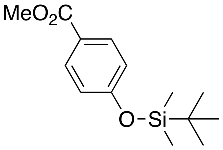

The title compound was prepared following the above procedure using methyl 4-hydroxybenzoate (1.52 g, 10 mmol), imidazole (0.82 g, 12 mmol), TBDMSCl (1.81 g, 12 mmol), and CH_2Cl_2 (25 mL). The product was obtained as a colorless liquid (2.34 g, 88% yield). ^{1}H NMR (500 MHz, $CDCl_3$): δ 7.94 (d, *J* = 8.5 Hz, 2H), 6.86 (d, *J* = 8.5 Hz, 2H), 3.88 (s, 3H), 0.99 (s, 9H), 0.23 (s, 6H); ^{13}C NMR (125 MHz, $CDCl_3$): δ 166.9, 160.0, 131.5, 123.2, 119.8, 51.8, 25.6, 18.2, -4.4. ^{1}H and ^{13}C NMR data matched those previously reported results (*11*).

tert-Butyl(4-fluorophenoxy)dimethylsilane (**15g**)

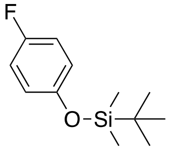

The title compound was prepared following the above procedure using 4-fluorophenol (1.12 g, 10 mmol), imidazole (0.82 g, 12 mmol), TBDMSCl (1.81 g, 12 mmol), and CH_2Cl_2 (25 mL). The product was obtained as a yellowish liquid (2.06 g, 91% yield). ^{1}H NMR (500 MHz, $CDCl_3$): δ 6.98-6.88 (m, 2H), 6.82-6.74 (m, 2H), 1.0 (s, 9H), 0.19 (s, 6H), ^{13}C NMR (125 MHz, $CDCl_3$): δ 158.5, 156.7, 151.6, 151.6, 120.9, 120.8, 115.8, 115.6, 25.7, 18.2, -4.5. ^{1}H NMR data matched those previously reported results (*12*).

tert-Butyldimethyl(naphthalen-2-yloxy)silane (**15i**)

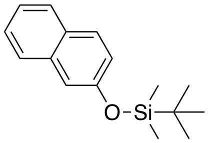

The title compound was prepared following the above procedure using naphthalen-2-ol (1.44 g, 10 mmol), imidazole (0.82 g, 12 mmol), TBDMSCl (1.81 g, 12 mmol), and CH_2Cl_2 (25 mL). The product was obtained as a colorless liquid (2.52 g, 97.5% yield). ^{1}H NMR (500 MHz, $CDCl_3$): δ 7.80 (d, *J* = 8.0 Hz, 1H), 7.76 (d, *J* = 9.0 Hz, 1H), 7.73 (d, *J* = 7.5 Hz, 1H), 7.46 (d, *J* = 8.0 Hz, 1H), 7.37 (d, *J* = 8.0 Hz, 1H), 7.27 (s, 1H), 7.12 (dd, *J* = 8.5, 1.4 Hz, 1H), 1.07 (s, 9H), 0.29 (s, 6H). ^{13}C NMR (125 MHz, $CDCl_3$): δ 153.5, 134.7, 129.3, 129.3, 127.7, 126.7, 126.1, 123.8, 122.1, 114.9, 25.8, 18.3, -4.3. ^{1}H and ^{13}C NMR data matched previously reported results (*13*).

(S)-2,6-Bis((*tert*-butyldimethylsilyl)oxy)-3,5-dichloro-N-((1-ethylpyrrolidin-2-yl)methyl)benzamide (**17**)

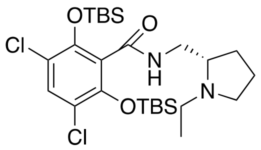

To a two-neck round-bottom flask (25 mL) equipped with a magnetic stirrer and a septum, and cooled with an ice-water bath, were added desmethylraclopride (67 mg, 0.20 mmol), DMF (3.5 mL), CH_2Cl_2 (2.0 mL), imidazole (41 mg, 0.60 mmol) and TBDMSCl (90 mg, 0.60 mmol), consecutively. After maintained at 0 ^{o}C for 3 h with vigorous stir, the reaction was quenched with saturated $NaHCO_3$ (10 mL) and EtOAc (10 mL). Following the separation of the two phases, the aqueous phase was extracted with EtOAc (10 mL x 2). The organic phases were combined, washed with saturated $NaHCO_3$ (20 mL) and dried with Na_2SO_4. The solvent was removed in *vacuo* and the oily residue was purified by flash chromatography on silica (EtOAc : hexane = 1 : 5) to give a white solid (100 mg, 89% yield). ^{1}H NMR (500 MHz, $CDCl_3$): δ 7.34 (s, 1H), 6.08 (s, 1H), 3.73-3.59 (m, 1H), 3.24-3.09 (m, 2H), 2.89-2.72 (m, 1H), 2.62-2.52 (m, 1H), 2.24-2.08 (m, 1H), 1.96-1.82 (m, 1H), 1.78-1.52 (m, 3H), 1.07 (t, *J* = 7.0 Hz, 3H), 1.01 (s, 18H), 0.23 (s, 6H), 0.22 (s, 6H); ^{13}C NMR (125 MHz, $CDCl_3$): δ 164.2, 148.3, 130.6, 125.5, 118.7, 62.1, 53.3, 48.1, 41.6, 28.6, 26.2, 22.4, 18.8, 13.8, -3.3, -3.4. HRMS (ESI) for $C_{26}H_{46}Cl_2N_2O_3Si_2$ $[M]^+$ requires 561.2497; found 561.2484. This compound was not previously reported.

Ethyl 2-(4-isobutylphenyl)acetate

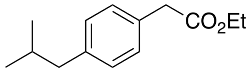

The title compound was prepared according to literature method (*14*):

To a 250-mL round-bottom flask, anhydrous ethanol (80 mL) was added. It was cooled with an ice water bath, and thionyl chloride (1.83 mL, 25 mmol) was slowly added. After 15 min, 2-(4-isobutylphenyl)acetic acid (1.92 g, 10 mmol) was added. The reaction mixture was stirred at r.t. for 3 h and the solution was concentrated to get the title product ethyl 2-(4-isobutylphenyl)acetate (2.20 g, quant.) as a colorless liquid. (The NMR spectra indicated that the final product contains a small amount of EtOH). ^{1}H NMR (500 MHz, $CDCl_3$) δ 7.19 (d, *J* = 7.5 Hz, 2H), 7.10 (d, *J* = 7.5 Hz, 2H), 4.15 (q, *J* = 7.0 Hz, 2H), 3.59 (s, 2H), 2.46 (d, *J* = 7.0 Hz, 2H), 1.91-1.80 (m, 1H), 1.26 (t, *J* = 7.0 Hz, 3H), 0.90 (d, *J* = 6.5 Hz, 6H); ^{13}C NMR (125 MHz, $CDCl_3$): δ 171.9, 140.5, 131.4, 129.3, 128.9,

60.8, 45.1, 41.1, 30.2, 22.4, 14.2. ESI (+) = 221.3 $(M+H)^+$. Calculated mass: 220.2. ^1H NMR data matched those previously reported results (*15*).

Ethyl 2-diazo-2-(4-isobutylphenyl)acetate

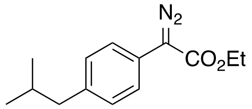

The title compound was prepared according to literature method (*16*):

To a mixture of ethyl 2-(4-isobutylphenyl)acetate (1.10 g, 5 mmol), and tosyl azide (1.48 g, 7.5 mmol) in anhydrous MeCN (10 mL), 1,8-diazabicyclo[5.4.0]undec-7-ene (DBU) (1.12 mL, 1.14 g, 7.5 mmol) was added. The reaction mixture was stirred at r.t. for overnight, making sure to keep it out from direct exposure of light. Upon complete consumption of the starting material ester, the reaction mixture was concentrated under reduced pressure. The residue was purified by flash chromatography (hexane : EtOAc = 9 : 1) to give a reddish oil as the desired α- ethyl 2-diazo-2-(4-isobutylphenyl)acetate (0.72 g, 59%). ^1H NMR (500 MHz, $CDCl_3$) δ 7.39 (d, J = 7.5 Hz, 2H), 7.17 (d, J = 7.5 Hz, 2H), 4.33 (q, J = 7.0 Hz, 2H), 2.46 (d, J = 7.0 Hz, 2H), 1.92-1.80 (m, 1H), 1.35 (t, J = 7.0 Hz, 3H), 0.91 (d, J = 6.5 Hz, 6H); ^{13}C NMR (125 MHz, $CDCl_3$) δ 165.5, 139.5, 129.7, 124.0, 122.5, 60.9, 51.9, 44.9, 30.2, 22.3, 14.5; ESI (+) = 219.2 $(M-N_2+H)^+$. Calculated mass: 246.1. Known compound, no reported data (Z. Wang, A. G. Herraiz, A. M. del Hoyo, M. G. Suero, *Nature* **2018**, *554*, 86-91.)

Ethyl 2-(4-*iso*butylphenyl)-2-(triethylsilyl)acetate (**15o**)

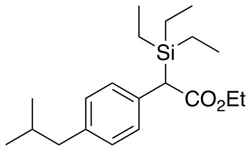

The title compound was prepared according to literature methods (*17*):

To an oven-dried glass tube, under argon atmosphere, $Cu(OTf)_2$ (18 mg, 0.05 mmol, 5 mol%), CH_2Cl_2 (7.0 mL) and triethyl silane (0.32 mL, 2.0 mmol) were added consecutively, and the mixture was cooled with an ice water bath. Ethyl α-diazoacetate (0.25 g, 1.0 mmol, diluted with 13 mL CH_2Cl_2), as prepared above, was added dropwise over 2 h using a syringe pump. The mixture was further maintained at r.t. with stirring for 1 h; the solvent was then removed under vacuum. The residue was purified by flash chromatography (hexane : EtOAc = 97 : 3) to afford a colorless oil as the product (0.20 g, 60% yield). ^1H NMR (500 MHz, $CDCl_3$) δ 7.25 (d, J = 10.0 Hz, 2H), 7.04 (d, J = 8.5 Hz, 2H), 4.20-4.08 (m, 2H), 3.49 (s, 1H), 2.43 (d, J = 7.0 Hz, 2H), 1.90-1.79 (m, 1H), 1.28 (t, J = 7.0 Hz, 3H), 0.94-0.83 (m ,15H), 0.65-0.53 (m, 6H); ^{13}C NMR (125 MHz, $CDCl_3$) δ 173.4, 138.8, 133.7, 128.8, 128.2, 60.1, 45.0, 42.4, 30.2, 22.4, 22.3, 14.3, 7.08, 2.7; ESI (+) = 335.3 $(M+H)^+$. Calculated mass: 334.23. HRMS (ESI) for $C_{20}H_{34}O_2Si$ $[M]^+$ requires 335.2401; found 335.2384. This compound was not previously reported.

General Information for Radiochemistry Experiments

The reagents and solvents used for the synthesis process were generally purchased from Sigma-Aldrich (MO, USA), Thermo Fisher Scientific and TCI America with a minimum of ACS reagent grade, or from ABX *Advanced Biochemical Compounds* and used without further purification. Solid phase extraction (SPE) cartridges were obtained from Waters (Waters Corporation, MA, USA). Reaction vials were purchased from Biotage (Biotage® Microwave reaction Kit, 2-5 mL or 0.5 – 2 mL). High and low level radioactivity measurements were performed using a Capintec CRC-15 DUAL PET radioisotope dose calibrator (Capintec Inc., NJ, USA). Semi-prep radio-HPLC purification and analytical radio-HPLC analysis was performed using either a VARIAN ProStar HPLC system (Agilent Technologies) equipped with dual Model 210 pumps and a ProStar UV-vis 325 or 1260 DAD VL dual ultraviolet-visible light (UV-vis) detector, or RefractoMax 520 RI detector; Or Agilent 1200 series equipped with Interface35900E multichannel interference. The radioactivity signal was measured with Eckert & Ziegler Flow-Count radio-HPLC detecting system equipped with Bioscan B-FC-3400 PIN diode or a NaI PMT detector. The HPLC chromatographic data were collected using a ProStar data acquisition system, Galaxie Chromatography Data system or OpenLab system. The desired products were identified by comparing the retention time of peaks from radio-HPLC chromatography with the UV peak coming from co-injected product standard; the retention time had been previously verified using identical conditions.

[^{11}C]CO_2 production

[^{11}C]CO_2 was generated by bombarding N_2 gas (360 psi 99.9999% pure N_2 doped with 0.5% O_2) via the ^{14}N(p,α)^{11}C nuclear reaction using a EBCO TR-19/9 cyclotron. General bombardment conditions: 2 – 40 min beam time with 25 µA current (3.7 – 44.4 GBq; 100 – 1200 mCi). After the bombardment, target gas containing radioactivity was released and delivered to a home-made automated [^{11}C]CO_2 purification box for controlled trap and release of [^{11}C]CO_2, where the [^{11}C]CO_2 was first trapped by a molecular sieve (MS) furnace at room temperature (200 mg Molecular Sieve 13X, 100/120 mesh, SUPELCO). Next, the furnace was heated to 190 ^{o}C and [^{11}C]CO_2 was released and delivered to the reaction vial using helium flow (10 mL/min). Once the radioactivity collected in the reaction vial plateaued, the delivery was stopped and [^{11}C]CO_2 production and collection was done. It took 3 – 4 min from end of bombardment (EOB) to finish the collection of [^{11}C]CO_2 in the reaction vial.

General procedures of ^{11}C-carboxylation reaction, determination of radiochemical purities (RCP) and radiochemical yields (RCY)

When the [^{11}C]CO_2 was ready, to a reaction vial equipped with outlet line (an ascarite trap was attached at the end for trapping escaped [^{11}C]CO_2) the [^{11}C]CO_2 delivery line with a 4 inch needle was inserted into the anhydrous fluoride reagent and solvent reaction mixture. After confirmation of a stable helium flow (10 mL/min), the organosilane precursor was immediately added. The reaction vial was placed in a dose calibrator for measuring the collected radioactivity. Once the increase of radioactivity in the reaction vial plateaued, the [^{11}C]CO_2 delivery line and airflow outlet line were removed immediately. The total activity trapped in the reaction vial was checked again, and recorded as starting radioactivity $\mathbf{A_0}$. When dimethylformamide (DMF), dimethylacetamide (DMA) and dimethyl sulfoxide (DMSO), as well as its mixture with tetrahydrofuran (THF) (1/1, v/v) were used as solvents for reactions, the trapping of radioactivity [^{11}C]CO_2 was generally efficient and the escaped [^{11}C]CO_2 collected by an ascarite trap attached to the reaction vial was usually less than 10%. When only THF or THF/dichloromethane (DCM) (3/1, v/v) was used as the reaction solvent, a cooling bath was used (-70 – 0 ^{o}C) to keep reaction vial cool and leaked radioactivity was also minimal (< 10%). After maintaining the reaction mixture at the desired temperature with stirring for a certain time frame (normally 2 – 10 minutes), the reaction was quenched by addition of an acidic solution (1 mL, CH_3CN/H_2O/formic acid, 90/9/1; or 0.1 M HCl aqueous solution). The unreacted [^{11}C]CO_2 was excluded from the reaction vial by purging with a gentle stream of helium (2 – 5 psi) and it was trapped in a second ascarite trap. When the radioactivity collected in this ascarite trap became constant ($\mathbf{A_{leak}}$), the remaining radioactivity in the reaction vial was again measured and recorded as $\mathbf{A_{left}}$. After that, a small portion of solution was removed from reaction vial (0.1 – 0.2 mL) and diluted in a sample vial pre-loaded with an acidic solution (1 mL, CH_3CN/1% formic acid, 90/10). Next, an analytical sample, which was a mixture of an aliquot of sample solution (usually 10 µL) and a product standard solution (usually 10 µL, 1 mg/mL solution), was injected into HPLC for analysis. The percentage of the radio-peak in radio-chromatogram coincident with product reference UV peak was regarded as radiochemical purity (RCP). The radiochemical yield (RCY) was calculated by the equation $\frac{RCP \times Aleft}{A0} \times 100\%$. The $\mathbf{A_0}$ and $\mathbf{A_{left}}$ were decay corrected values. If the reaction mixture was submitted for the purification process (solid phase extraction, anion/cation resins exchange method, semi-prep HPLC, or the combination of two of these methods), the total amount of radioactivity of purified product was recorded as $\mathbf{A_{prod}}$. The radiochemical yield (RCY, decay corrected) was calculated as $\frac{Aprod}{A0} \times 100\%$. **Molar activity values ($\mathbf{A_m}$)**, decay corrected back to EOB and recorded in GBq/µmol, were determined from the carbon-11 activity in the HPLC product peak and the mass of compound. Total synthesis times were calculated from time point of finished collection of [^{11}C]CO_2 to the end of radiotracer purification process.

^{11}C-Carboxylation experimental results

[1-^{11}C]Acetoactic acid ([^{11}C]3 or [^{11}C]AcAc) production – lithium reagent based method (*18, 19*)

Pre-labeling work:

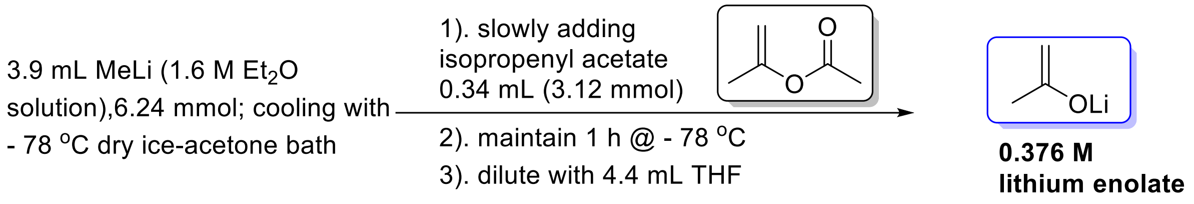

1). Citrate buffer preparation: weigh citric acid (304 mg), citric acid trisodium salt (179 mg) and NaCl (113 mg) and dissolve them with 25 mL injectable water to give citrate buffer (pH = 3.5).

2). Ion-exchange column conditioning: weigh DOWEX 50WX8-100 resin (400 mg), pour it into a chromatography flex-column (0.7 × 4 cm) and wash it with sterile H_2O (10 mL). Similarly, weigh AG 1X-8 resin (150 mg), pour it into a chromatography flex-column (0.7 × 4 cm) and wash it with NaOH (6 mL, 1 M), rinse with sterile H_2O until a neutral pH was reached.

3). To a pre-dried R.B. flask (50 mL), cooled with acetone-dry ice bath, MeLi (3.9 mL 1.6 M) was added. After that, isopropenyl acetate (3.12 mmol, 0.34 mL) was added dropwise into flask, with caution. Once this was finished, the reaction solution was maintained at -78 °C for 1 h and was then diluted with THF (4.4 mL) to give fresh prepared lithium enolate reagent (0.376 M).

Radiolabeling and purification:

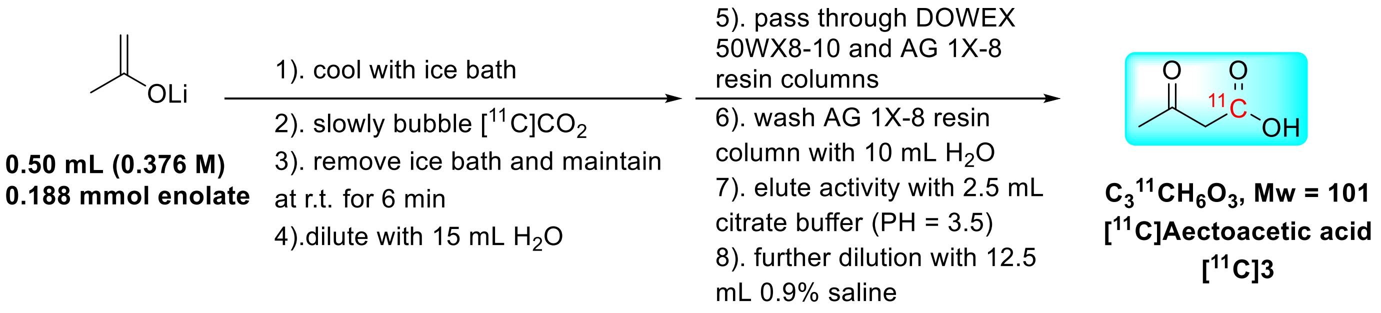

1) To a pre-dried reaction vial cooled with ice-water bath, enolate reagent (0.5 mL, 0.376 M) was added. When [^{11}C]CO_2 was ready, it was delivered by bubbling into the enolate solution using helium flow (10 mL/min). When the radioactivity in reaction vial plateaued, the reaction vial was removed from the ice-water bath and maintained at r.t. for 6 min while stirring.

2) The reaction solution was diluted with H_2O (15 mL) and the formed mixture was passed through DOWEX and AG 1X-8 resin columns consecutively. The column containing AG 1X-8 resin was further washed with H_2O (10 mL) and the radioactivity trapped in it was eluted with citrate buffer (2.5 mL) and the acidic crude product solution was collected in a plastic tube (15 mL).

3) Unreacted [^{11}C]CO_2 dissolved in crude product solution was excluded from citrate elute by flushing this solution with a stream of N_2 (5 psi) until no radioactivity increase was found in the ascarite trap (usually ≤ 3 min) attached at the end of an air-flow line. The radioactivity left in this plastic tube was measured and regarded as the final product. If necessary, this solution was passed through a 0.22 µm Millex GV filter for sterilization and the collected solution in the sterile vial was the final drug product (FDP).

Supplementary Table 1. Summarization of **[^{11}C]3** synthesis experimental results

**Exp. No.**	**Starting [^{11}C]CO_2 (GBq)**	**Prod [^{11}C]3 (GBq)**	**Total Synthesis Time (min)**	**RCY**	**RCP**
**01**	33.3	3.06	27	23.0%	97.9%
**02**	35.5	3.95	22	23.5%	97.6%
**03**	38.3	4.67	23	26.6%	95.8%
**Avg ± SD**	35.7 ± 2.5	3.9 ± 0.8	24.0 ± 2.6	24.4 ± 2.0%	97.1 ± 1.1%

Supplementary Figure 1. A **[^{11}C]3** final product analytical HPLC profile (co-injected with acetoacetic acid standard). HPLC conditions: Bio-Rad HPX-87H column, 300 × 7.8 mm; 0.005 M H_2SO_4 as mobile phase, 0.6 mL/min; UV Wavelength: 205 nm.

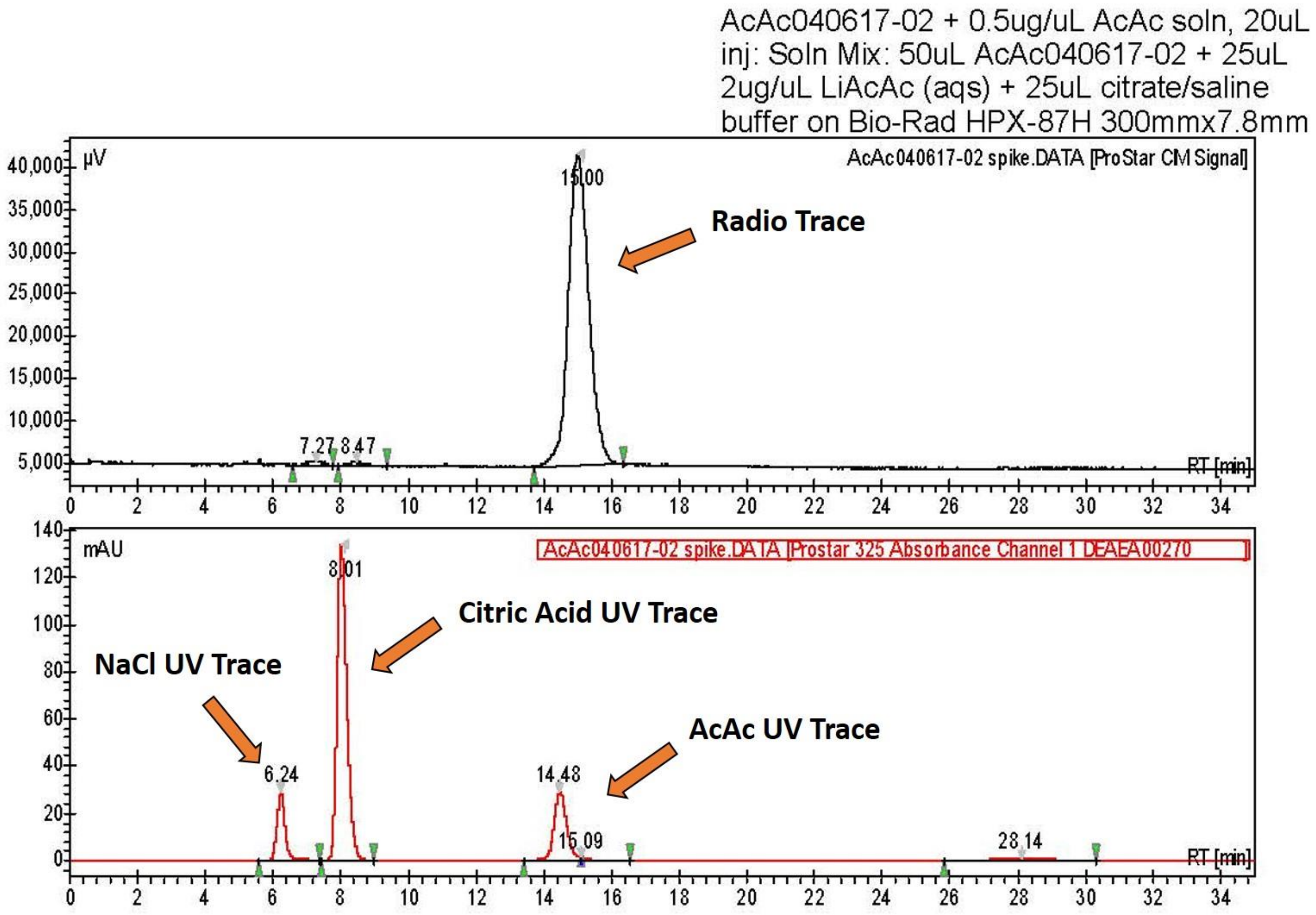

[1-^{11}C]Acetoactic acid ([^{11}C]3) synthesis – FMDS ^{11}C-carboxylation approach

Reaction parameters investigated in preliminary research:

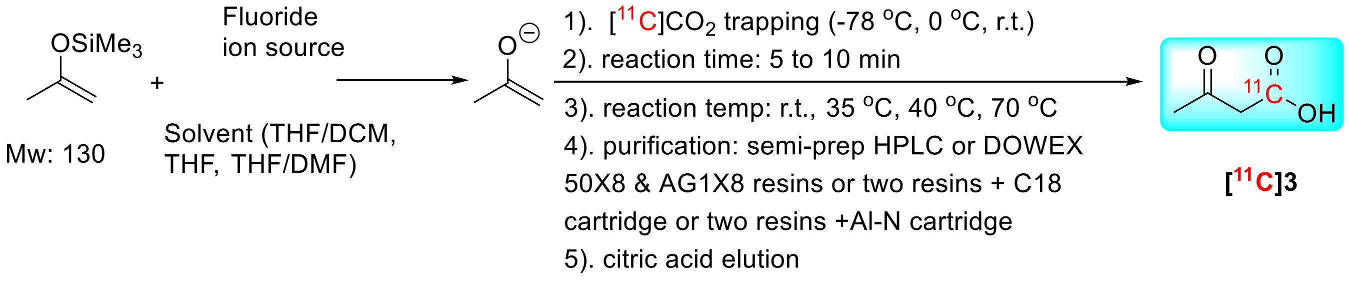

Optimized synthetic protocol for synthesis of [^{11}C]3:

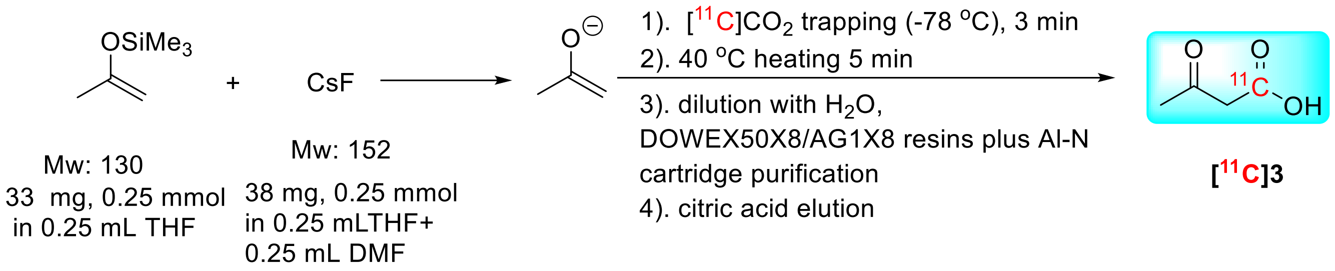

General procedure of pre-labeling work:

1). CsF stock solution: A stock solution was prepared by dissolving CsF(1.52 g, 10 mmol) in H_2O (12 mL) and stored in a FALCON® plastic tube (15 mL).

2). Azeotropic drying of CsF: The CsF stock solution (0.3 mL, 0.25 mmol) and CH_3CN (1 mL) was added to a microwave glass vial (Biotage, 2 – 5 mL, RV01) and the solvent was evaporated by heating at 120 °C under a mild argon stream. After most of the solvent was evaporated, CsF reagent was further dried azeotropically with CH_3CN (2 × 1 mL). Next, RV01 was heated at 140 °C under vacuum for 0.5 h to remove trace amount of H_2O from CsF. Once the drying process was complete, RV01 was cooled to room temperature under the argon atmosphere, solvent (0.25 mL DMF + 0.25 mL THF) was added and the glass vial was subjected to vigorously vortex (15 s), and it was ready for radiolabelling reaction.

3). To an oven dried glass vial (RV02), were added (isopropenyloxy)trimethylsilane (65 mg, 0.5 mmol) and THF (0.5 mL) as precursor stock solution.

4). Citrate buffer preparation: weigh citric acid (304 mg), citric acid trisodium salt (179 mg) and NaCl (113 mg) and dissolve them with 25 mL injectable water to give citrate buffer (pH = 3.5).

5). Ion-exchange column conditioning: weigh DOWEX 50WX8-100 resin (800 mg), pour it into a chromatographic flex-column (0.7 × 4 cm) and wash it with sterile H_2O (10 mL). Similarly, weigh AG 1X-8 resin (300 mg), pour it into a chromatographic flex-column (0.7 × 4 cm) and wash it with NaOH (6 mL, 1 M), rinse with sterile H_2O until a neutral PH was reached.

6). Pre-activate an Al-N Plus cartridge (1.71 g): the cartridge was activated by passing through EtOH (5 mL), 10 mL H_2O (10 mL) and air (60 mL × 2) consecutively.

Radiolabeling and purification:

1) To a reaction vial (RV01) pre-cooled with a dry ice-acetone bath, (isopropenyloxy)trimethylsilane stock solution (0.25 mL, 0.25 mmol) was added. Next, $[^{11}C]CO_2$ was delivered into this vessel using helium flow (10 mL/min). When the radioactivity in RV01 plateaued, the dry ice-acetone bath was removed and the reaction mixture was heated at 40 ^oC for 5 min with stirring.
2) The reaction mixture was diluted with H_2O (15 mL) and was passed through DOWEX and AG 1X-8 resin columns consecutively. The column containing AG 1X-8 resin was further washed with a portion of H_2O (10 mL) and the radioactivity trapped in it was eluted with citrate buffer (8 mL). After being passed through an Al-N plus cartridge, the acidic crude product solution was collected in a plastic tube (15 mL).
3) Unreacted $[^{11}C]CO_2$ dissolved in crude product solution was excluded from citrate elute by flushing this solution with a stream of N_2 (5 psi) until no radioactivity increase was found in the ascarite trap (usually ≤ 3 min) attached at the end of an air-flow line. The radioactivity left in this plastic tube was measured and regarded as the final product. If necessary, this solution was passed through a 0.22 µm Millex GV filter for sterilization and the collected solution in the sterile vial was the final drug product (FDP).

Supplementary Table 2. Experimental results of **$[^{11}C]$3** synthesis via FMDS ^{11}C-carboxylation approach

**Exp. No.**	**Starting $[^{11}C]CO_2$ (GBq)**	**Prod $[^{11}C]$3 (GBq)**	**Total Synthesis Time (min)**	**RCY**	**RCP**
**01**	7.77	1.80	29	62%	97.3%
**02***	16.3	2.46	34	48%	96.0%
**03**	11.8	3.35	23	62%	94.3%
**Avg ± SD**	11.9 ± 4.3	2.5 ± 0.8	28.7 ± 5.5	57.4 ± 8.1%	95.9 ± 1.5%

*(used half amount start materials for reaction, i.e. 0.125 mmol trimethylsilyl enol ether and 0.125 mmol CsF)

Supplementary Figure 2. A **[11C]3** final product analytical HPLC profile (co-injected with acetoacetic acid standard). HPLC conditions: Bio-Rad HPX-87H column, 300 × 7.8 mm; 0.005 M H_2SO_4 as mobile phase, 0.6 mL/min; UV Wavelength: 205 nm.

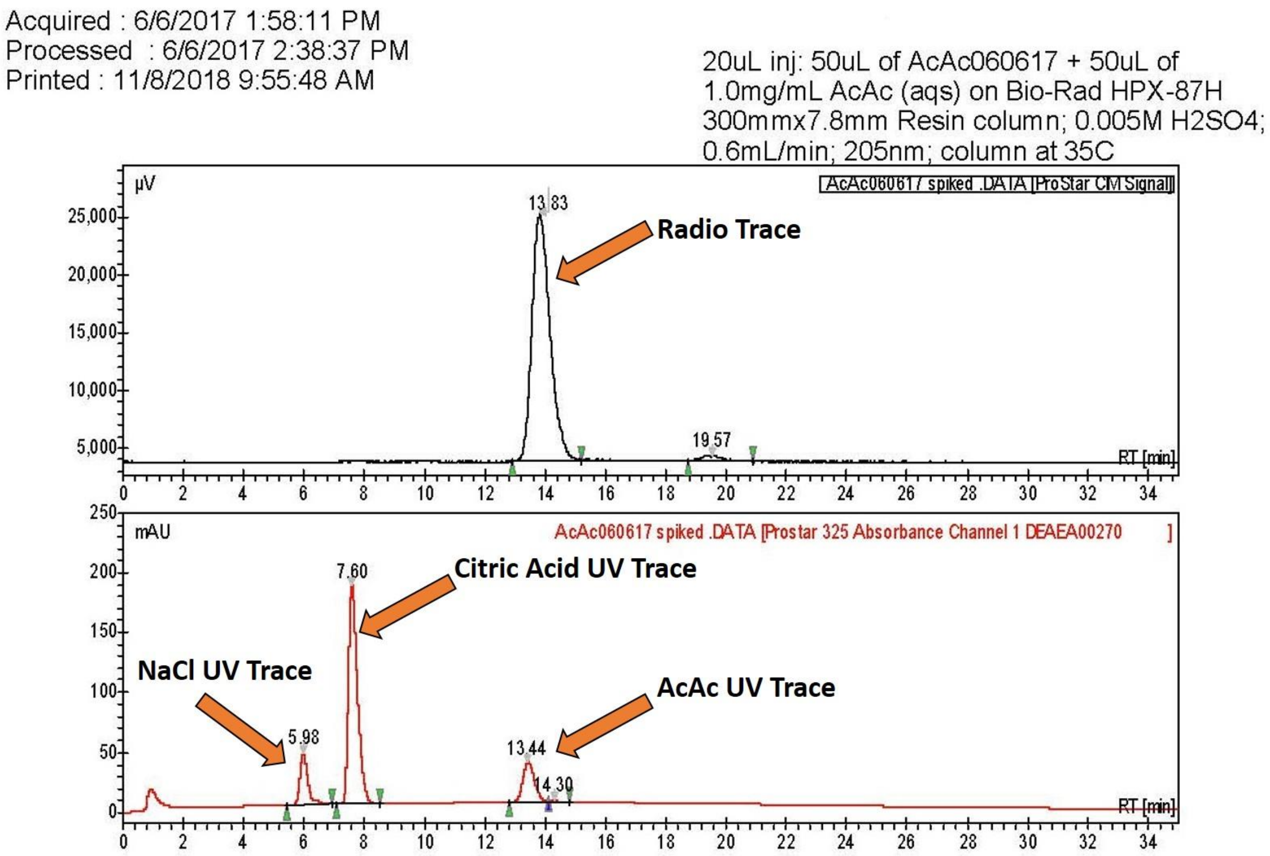

FMDS ^{11}C-carboxylation with sp-hybridized carbon attached trimethylsilanes

For FMDS ^{11}C-carboxylation, a typical experimental procedure is exemplified with the synthesis of [^{11}C]PPA, [^{11}C]7a:

3-Phenylpropiolic-1-[^{11}C]acid, ([^{11}C]7a) synthesis

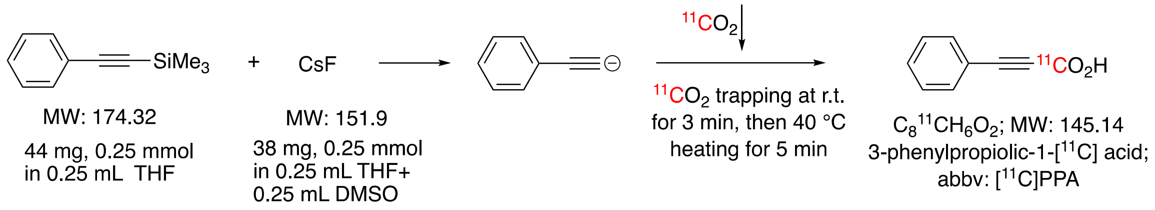

Pre-labeling work:

Following the procedures of pre-labeling work steps 1-3 described in the synthesis of **[^{11}C]3** by FMDS ^{11}C-carboxylation approach, with solvent changed to THF and DMSO (0.25 mL + 0.25 mL).

Radiolabeling:

1). When [^{11}C]CO_2 was ready, it was released into RV01 at r.t.. Meanwhile, trimethyl(phenylethynyl)silane **6a** (44mg, 0.25 mmol) stock solution prepared in RV02 (THF, 0.25 mL) was quickly transferred into RV01. When the radioactivity collected in RV01 plateaued, delivery and vent needles were removed and the radioactivity in RV01 was measured, and the reaction mixture was maintained at 40 ^{o}C for 5 min with vigorous stirring.

2). The reaction was quenched by adding an acidic solution (1.0 mL, CH_3CN/H_2O/formic acid, 90/10/1) into RV01, attached with 2nd ascarite trap. A stream of N_2 (5 psi) was flushed through the reaction mixture until the read-out of radioactivity [^{11}C]CO_2 trapped in the 2nd ascarite trap stop increasing (usually ≤ 3 min), count the left radioactivity in the RV01 as the crude product.

3). Sample solution: remove crude product solution from RV01 (~ 0.1 mL) and dilute it with an acidic solvent (CH_3CN/H_2O/formic acid, 90/10/1, 1 mL).

4). HPLC analysis used sample preparation: transfer an aliquot of above diluted crude product sample solution and an aliquot of carbon-12 standard solution into an Eppendorf safe-lock tube (1.5 mL) and vortex (10 s) for well mixing. After that, it was ready for analytical HPLC (A-HPLC) analysis.

5). HPLC analysis conditions: stationary phase: Phenomenex Luna(2) C18 150 × 4.6 mm; Mobile phase: phase A (0.1% formic acid), phase B (90% CH_3CN/10% H_2O); UV: 254 nm; Flow rate: 1 mL/min; Gradient/isocratic method: 40%B/60%A (0-12 min), rise 40%B to 100%B (12-13 min), 100%B (13-21 min). It is expected to wash all radioactive and UV active impurities from column after detection of desired product. In the rest of research, the crude product analytical sample preparation and the HPLC analysis process for obtaining RCP and RCY values were similar as above described method unless otherwise mentioned.

The detailed results are listed in the table below:

Supplementary Table 3. Summarization of [^{11}C]PPA, **[^{11}C]7a**, synthesis experimental results

No.	RCP and RCY
Exp 01	>99.0% & 95.8%
Exp 02	>99.0% & >99.0%
Exp 03	>99.0% & 94.7%
Exp 04	>99.0% & 94.2%
Avg ± SD of RCP and RCY	>99.0 ± 0.0% & 96.5 ± 2.2%

Supplementary Figure 3. A [^{11}C]PPA, **[^{11}C]7a**, crude product sample analytical HPLC profile

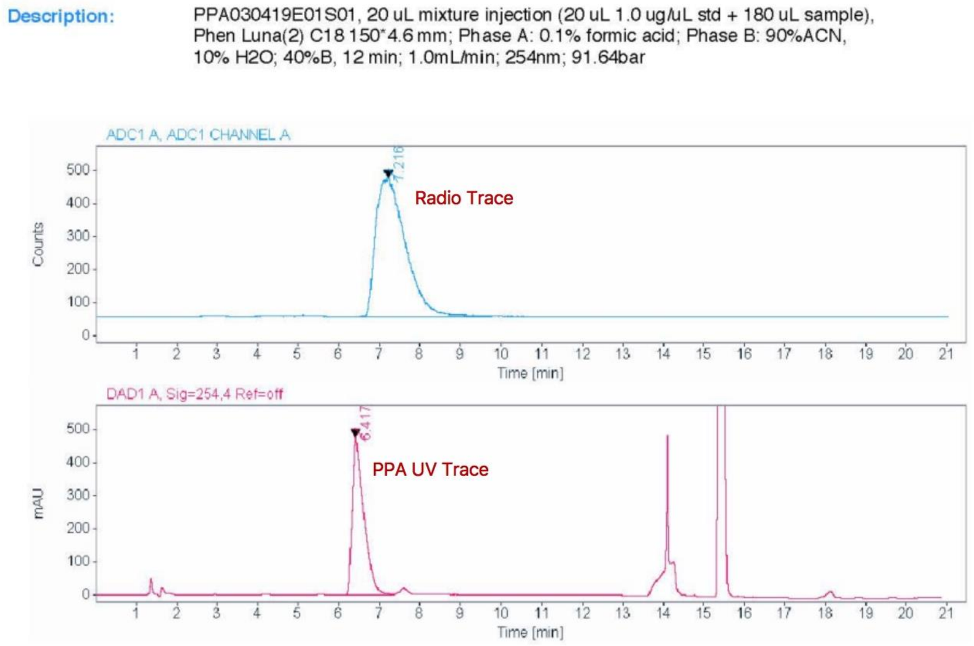

3-(4-Methoxyphenyl)propiolic-1-[^{11}C]acid, ([^{11}C]7b) synthesis

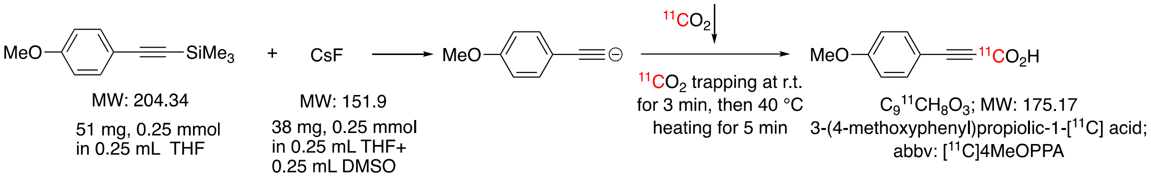

Following the procedures of pre-labeling work and ^{11}C-carboxylation reaction described above, [^{11}C]4MeOPPA, **[^{11}C]7b**, was synthesized (see above scheme for details) and the results are listed in the table below:

Supplementary Table 4. Summarization of **[^{11}C]7b** synthesis experimental results

No.	RCP and RCY
Exp 01	76.3% & 68.8%
Exp 02	86.2% & 62.9%
Exp 03	94.2% & 85.4%
Avg ± SD of RCP and RCY	85.6% ± 9.0% & 72.4% ± 11.7%

Supplementary Figure 4. A [^{11}C]4MeOPPA, **[^{11}C]7b**, crude product sample analytical HPLC profile

HPLC analysis conditions: stationary phase: Phenomenex Luna(2) C18 150 × 4.6 mm; Mobile phase: phase A (0.1% formic acid), phase B (90% CH_3CN/10% H_2O); UV: 254 nm; Flow rate: 1 mL/min; Gradient/isocratic method: 45%B/55%A (0-10 min), rise 45%B to 100%B (10-11 min), 100%B (11-26 min), drop 100%B to 45%B (26-27 min), 45%B (27-30 min).

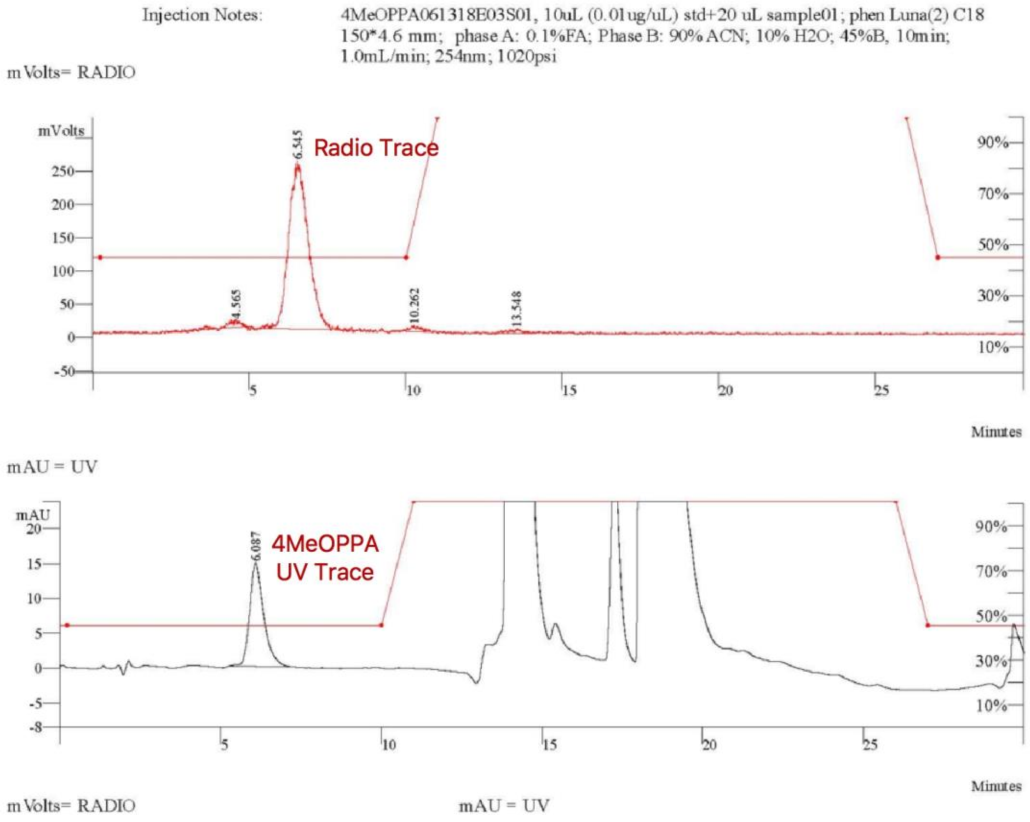

3-(4-Bromophenyl)propiolic-1-[^{11}C]acid, ([^{11}C]7c) synthesis

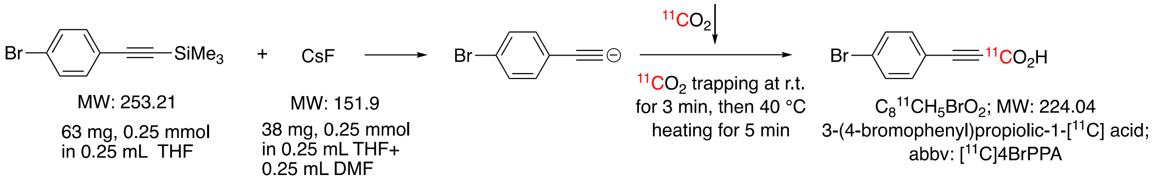

Following the procedures of pre-labeling work and ^{11}C-carboxylation reaction described above, [^{11}C]4BrPPA, **[^{11}C]7c**, was synthesized (see above scheme for details) and the results are listed in the table below:

Supplementary Table 5. Summarization of **[^{11}C]7c** synthesis experimental results

No.	RCP and RCY
Exp 01	98.4% & 80.0%
Exp 02	95.6% & 58.1%
Exp 03	>99.0% & 61.4%
Avg ± SD of RCP and RCY	97.7 ± 1.8% & 66.5 ± 11.8%

Supplementary Figure 5. A [^{11}C]4BrPPA, **[^{11}C]7c**, crude product sample analytical HPLC profile

HPLC analysis conditions: Stationary phase: Phenomenex Luna(2) C18 150 × 4.6 mm; mobile phase: phase A (0.1% formic acid), phase B (90% CH_3CN/10% H_2O); UV: 254 nm; Flow rate: 1 mL/min; Gradient/isocratic method: 55%B/45%A (0-10 min), rise 55%B to 100%B (10-11 min), 100%B (11-26 min), drop 100%B to 55%B (26-27 min), 55%B (27-30 min).

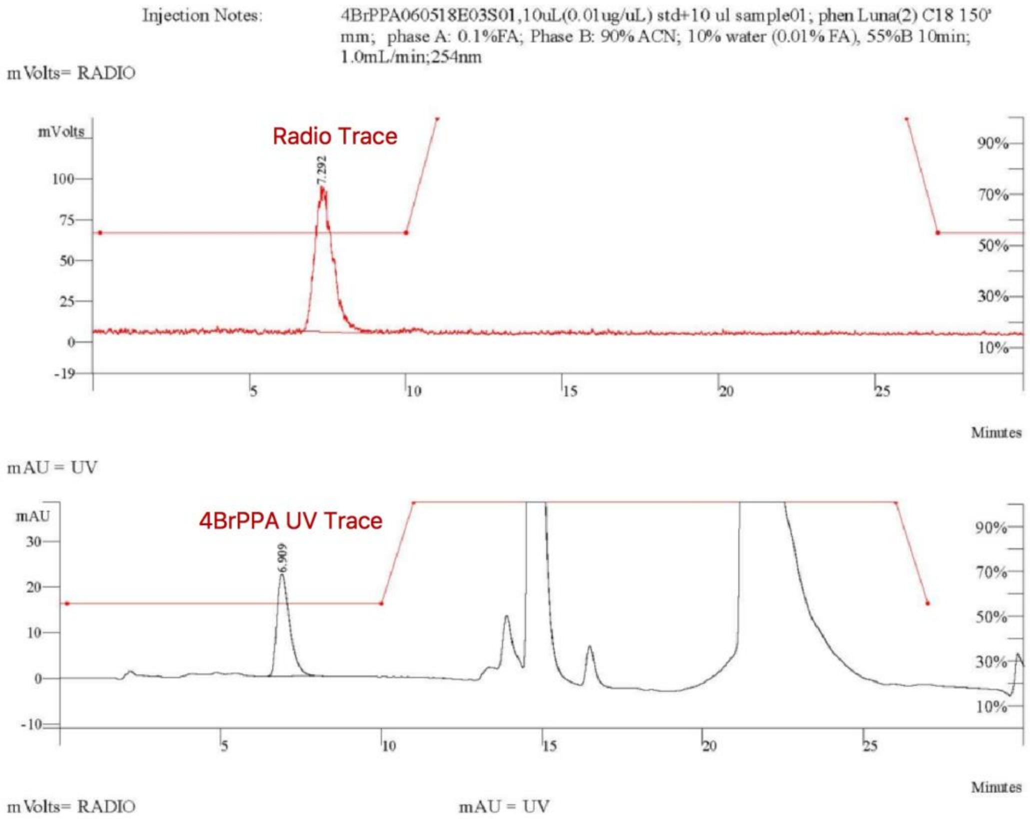

3-(4-Trifluoromethylphenyl)propiolic-1-[^{11}C]acid, ([^{11}C]7d) synthesis

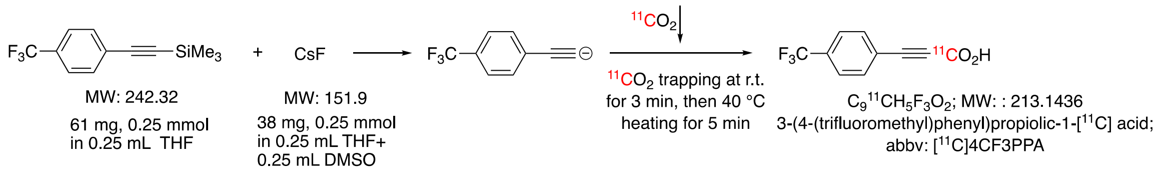

Following the procedures of pre-labeling work and ^{11}C-carboxylation reaction described above, [^{11}C]4CF3PPA, **[^{11}C]7d**, was synthesized (see above scheme for details) and the results are listed in the table below:

Supplementary Table 6. Summarization of **[^{11}C]7d** synthesis experimental results

No.	RCP and RCY
Exp 01	85.5% & 84.6%
Exp 02	90.9% & 89.2%
Exp 03	91.9% & 91.9%
Avg ± SD of RCP and RCY	89.4 ± 3.4% & 88.6 ± 3.7%

Supplementary Figure 6. A [^{11}C]4CF3PPA, **[^{11}C]7d,** crude product sample analytical HPLC profile

HPLC analysis conditions: stationary phase: Phenomenex Luna(2) C18 150 × 4.6 mm; Mobile phase: phase A (0.1% formic acid), phase B (90% CH_3CN/10% H_2O); UV: 254 nm; Flow rate: 1 mL/min; Gradient/isocratic method: 55%B/45%A (0-10 min), rise 55%B to 100%B (10-11 min), 100%B (11-26 min), drop 100%B to 55%B (26-27 min), 55%B (27-30 min).

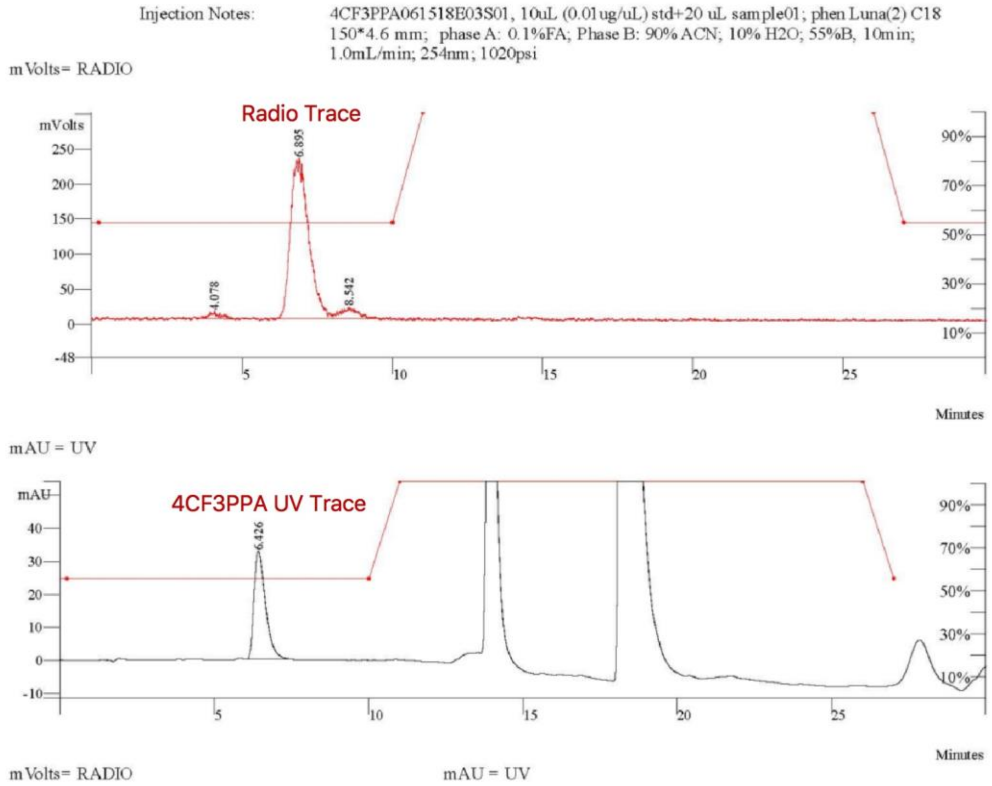

3-(2-Methoxyphenyl)propiolic-1-[^{11}C]acid, ([^{11}C]7e) synthesis

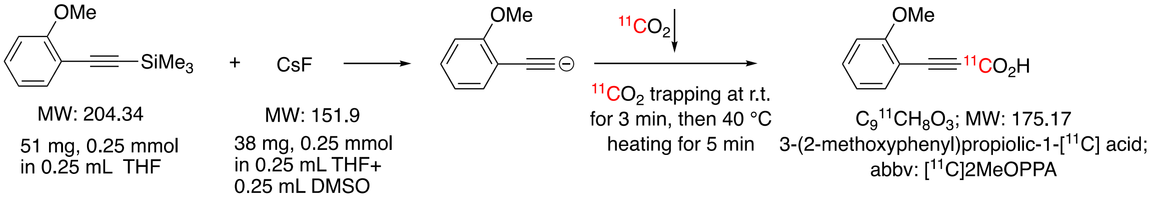

Following the procedures of pre-labeling work and ^{11}C-carboxylation reaction described above, [^{11}C]2MeOPPA, **[^{11}C]7e**, was synthesized (see above scheme for details) and the results are listed in the table below:

Supplementary Table 7. Summarization of **[^{11}C]7e** synthesis experimental results

No.	RCP and RCY
Exp 01	98.7% & 98.7%
Exp 02	99.7% & 99.7%
Exp 03	>99.0% & 97.0%
Avg ± SD of RCP and RCY	99.1 ± 0.5% & 98.5 ± 1.4%

Supplementary Figure 7. A [^{11}C]2MeOPPA, **[^{11}C]7e**, crude product sample analytical HPLC profile

HPLC analysis conditions: stationary phase: Phenomenex Luna(2) C18 150 × 4.6 mm; Mobile phase: phase A (0.1% formic acid), phase B (90% CH_3CN/10% H_2O); UV: 254 nm; Flow rate: 1 mL/min; Gradient/isocratic method: 45%B/55%A (0-10 min), rise 45%B to 100%B (10-11 min), 100%B (11-26 min), drop 100%B to 45%B (26-27 min), 45%B (27-30 min).

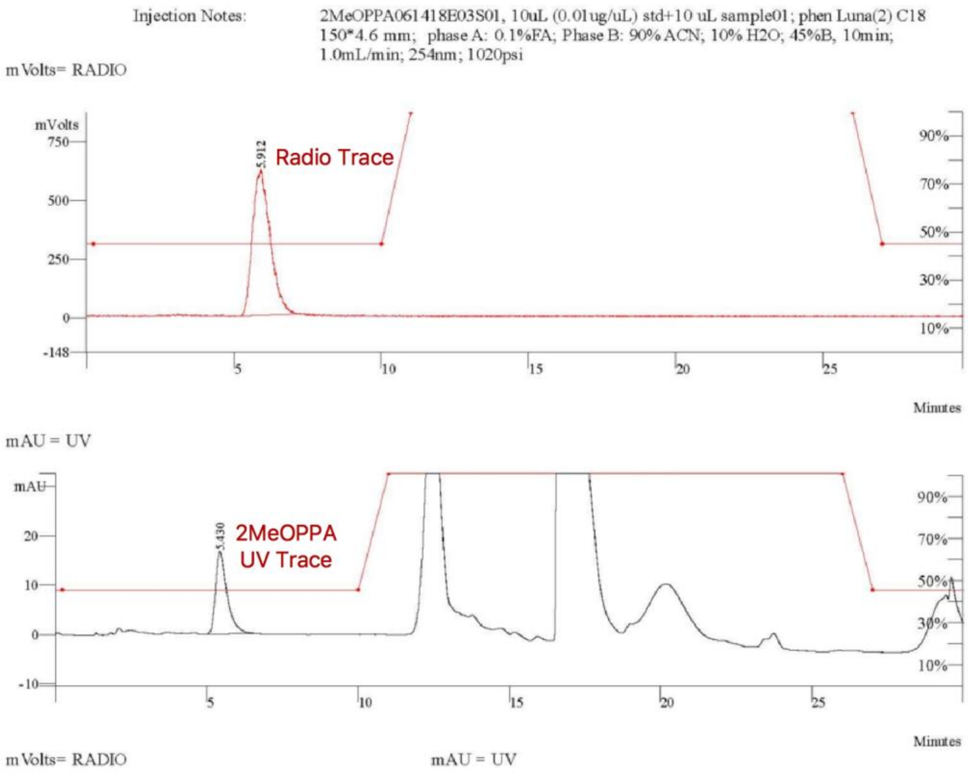

3-(3-Chlorophenyl)propiolic-1-[^{11}C]acid, ([^{11}C]7f) synthesis

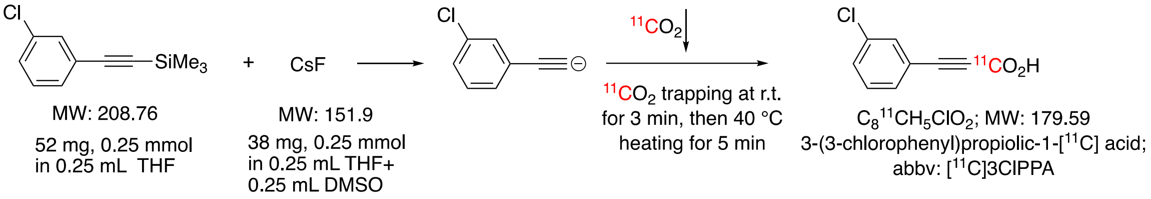

Following the procedures of pre-labeling work and ^{11}C-carboxylation reaction described above, [^{11}C]3ClPPA, **[^{11}C]7f,** was synthesized (see above scheme for details) and the results are listed in the table below:

Supplementary Table 8. Summarization of **[^{11}C]7f** synthesis experimental results

No.	RCP and RCY
Exp 01	85.3% & 79.7%
Exp 02	80.7% & 80.7%
Exp 03	71.9% & 71.9%
Avg ± SD of RCP and RCY	79.3 ± 6.8% & 77.4 ± 4.8%

Supplementary Figure 8. A [^{11}C]3ClPPA, **[^{11}C]7f,** crude product sample analytical HPLC profile

HPLC analysis conditions: stationary phase: Phenomenex Luna(2) C18 150 × 4.6 mm; Mobile phase: phase A (0.1% formic acid), phase B (90% CH_3CN/10% H_2O); UV: 254 nm; Flow rate: 1 mL/min; Gradient/isocratic method: 55%B/45%A (0-10 min), rise 55%B to 100%B (10-11 min), 100%B (11-26 min), drop 100%B to 55%B (26-27 min), 55%B (27-30 min).

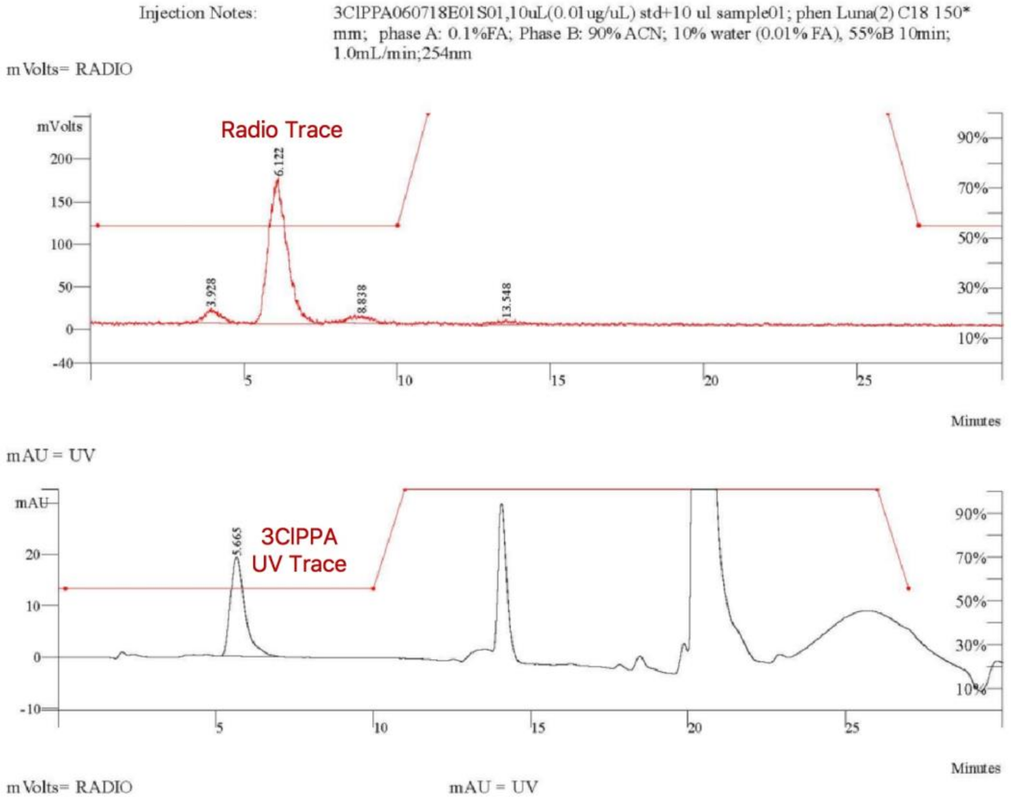

3-(Naphthalen-2-yl)propiolic-1-[^{11}C]acid, ([^{11}C]7g) synthesis

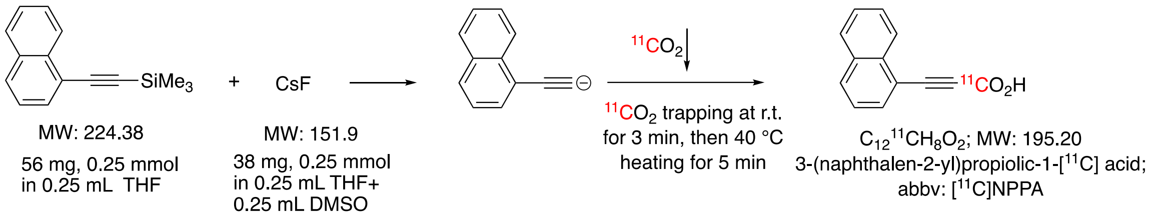

Following the procedures of pre-labeling work and ^{11}C-carboxylation reaction described above, [^{11}C]NPPA, **[^{11}C]7g**, was synthesized (see above scheme for details) and the detailed results are listed in the table below:

Supplementary Table 9. Summarization of **[^{11}C]7g** synthesis experimental results

No.	RCP and RCY
Exp 01	>99.0% & >99.0%
Exp 02	>99.0% & 95.0%
Exp 03	>99.0% & >99.0%
Avg ± SD of RCP and RCY	>99.0 ± 0.0% & 97.7 ± 2.3%

Supplementary Figure 9. A [^{11}C]NPPA, **[^{11}C]7g**, crude product sample analytical HPLC profile

HPLC analysis conditions: stationary phase: Phenomenex Luna(2) C18 150 × 4.6 mm; Mobile phase: phase A (0.1% formic acid), phase B (90% CH_3CN/10% H_2O); UV: 254 nm; Flow rate: 1 mL/min; Gradient/isocratic method: 55%B/45%A (0-10 min), rise 55%B to 100%B (10-11 min), 100%B (11-26 min), drop 100%B to 55%B (26-27 min), 55%B (27-30 min).

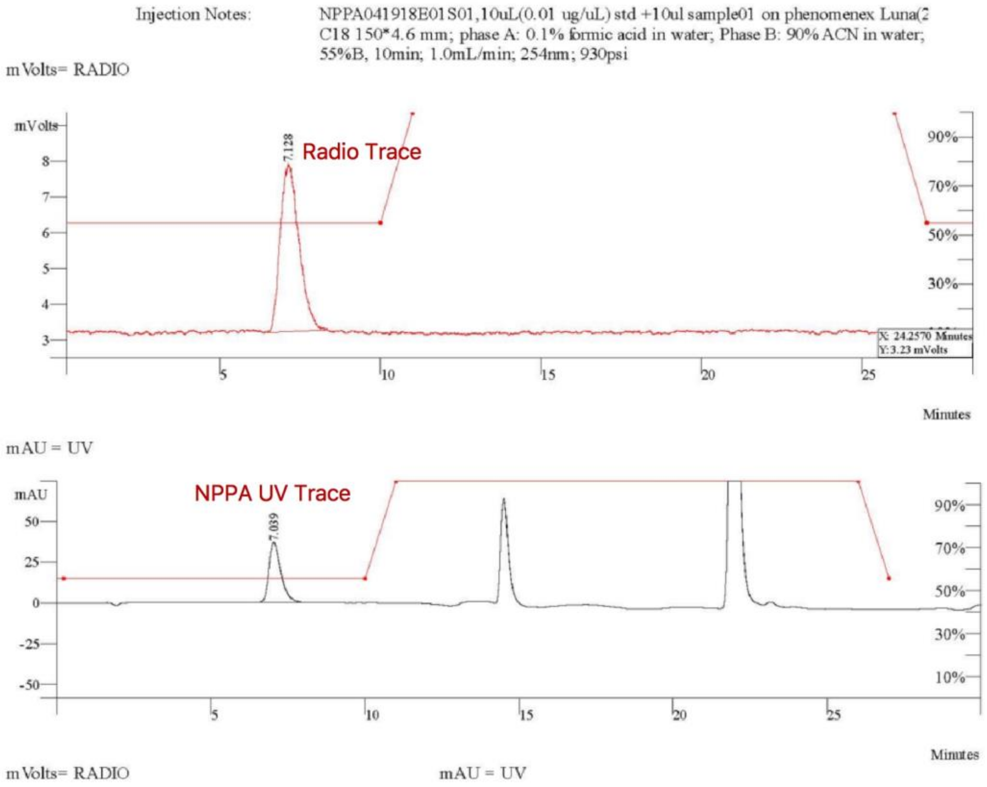

3-(Thiophen-2-yl)propiolic-1-[^{11}C]acid, ([^{11}C]7h) synthesis

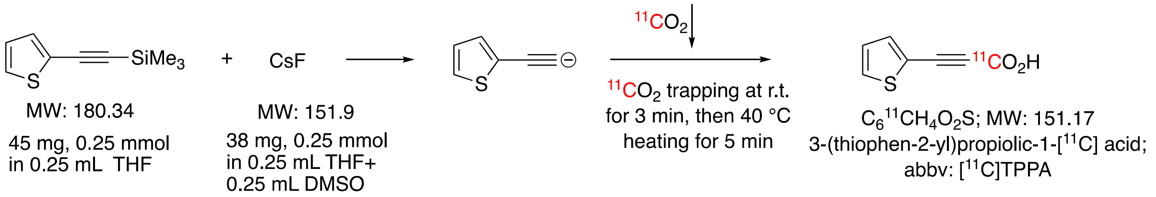

Following the procedures of pre-labeling work and ^{11}C-carboxylation reaction described above, [^{11}C]TPPA, **[^{11}C]7h**, was synthesized (see above scheme for details) and the detailed results are listed in the table below:

Supplementary Table 10. Summarization of **[^{11}C]7h** synthesis experimental results

No.	RCP and RCY
Exp 01	89.5% & 86.0%
Exp 02	81.2% & 79.8%
Exp 03	88.9% & 88.2%
Avg ± SD of RCP and RCY	86.5 ± 4.6% & 84.7 ± 4.4%

Supplementary Figure 10. A [^{11}C]TPPA, **[^{11}C]7h,** crude product sample analytical HPLC profile

HPLC analysis conditions: stationary phase: Phenomenex Luna(2) C18 150 × 4.6 mm; Mobile phase: phase A (0.1% formic acid), phase B (90% CH_3CN/10% H_2O); UV: 254 nm; Flow rate: 1 mL/min; Gradient/isocratic method: 60%B/40%A (0-10 min), rise 60%B to 100%B (10-11 min), 100%B (11-26 min), drop 100%B to 60%B (26-27 min), 60%B (27-30 min).

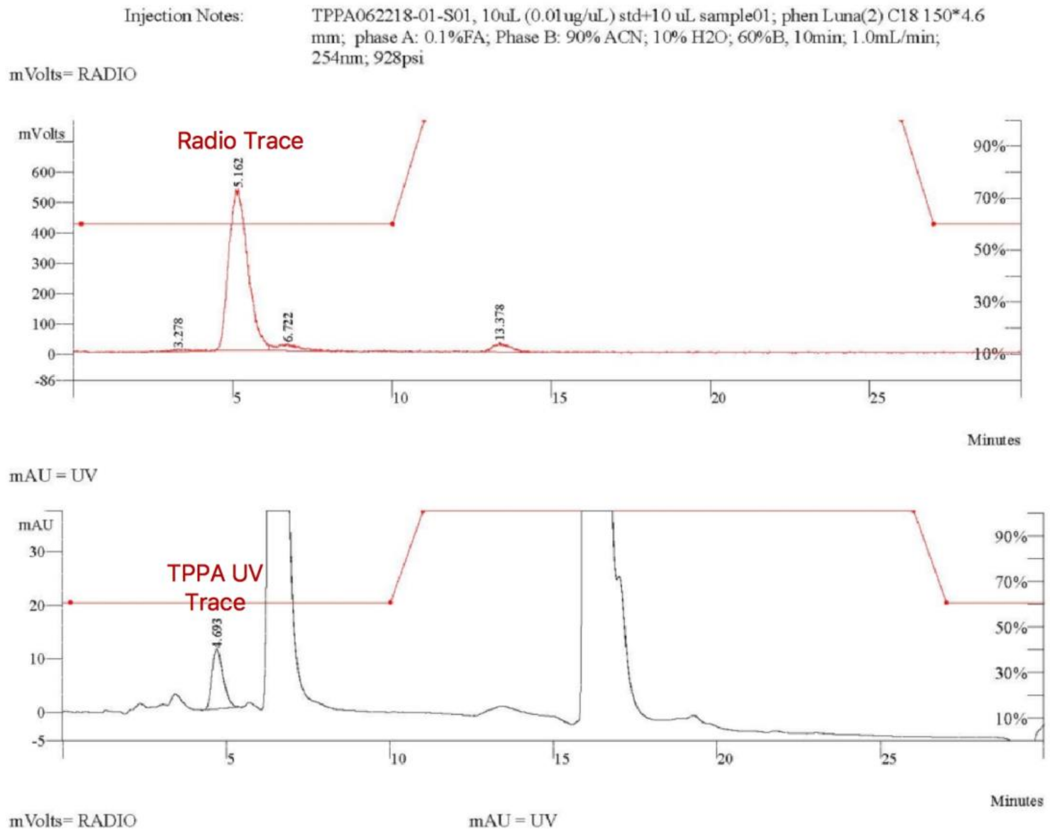

3-(Methoxycarbonyl)propiolic-1-[^{11}C]acid, ([^{11}C]7i) synthesis

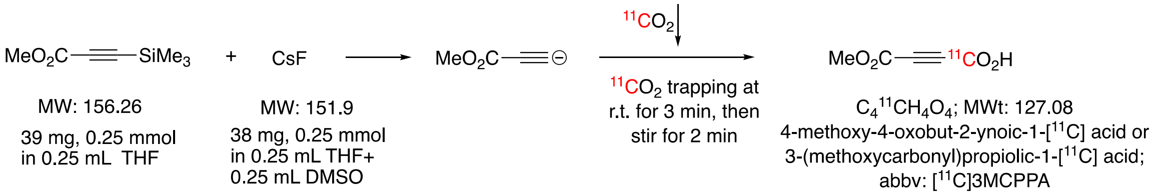

Following the procedures of pre-labeling work and ^{11}C-carboxylation reaction described above, [^{11}C]3MCPPA, **[^{11}C]7i**, was synthesized (see above scheme for details) and the detailed results are listed in the table below:

Supplementary Table 11. Summarization of **[^{11}C]7i** synthesis experimental results

No.	RCP and RCY
Exp 01	97.8% & 91.2%
Exp 02	98.0% & 98.0%
Exp 03	98.1% & 98.1%
Avg ± SD of RCP and RCY	98.0 ± 0.2% & 95.8 ± 4.0%

Supplementary Figure 11. A [^{11}C]3MCPPA, **[^{11}C]7i,** crude product sample analytical HPLC profile

HPLC analysis conditions: stationary phase: Phenomenex Luna(2) C18 150 × 4.6 mm; Mobile phase: phase A (0.1% formic acid), phase B (90% CH_3CN/10% H_2O); UV: 220 nm; Flow rate: 1 mL/min; Gradient/isocratic method: 3%B/97%A (0-15 min), rise 3%B to 100%B (15-16 min), 100%B (16-31 min), drop 100%B to 3%B (31-32 min), 3%B (32-35 min).

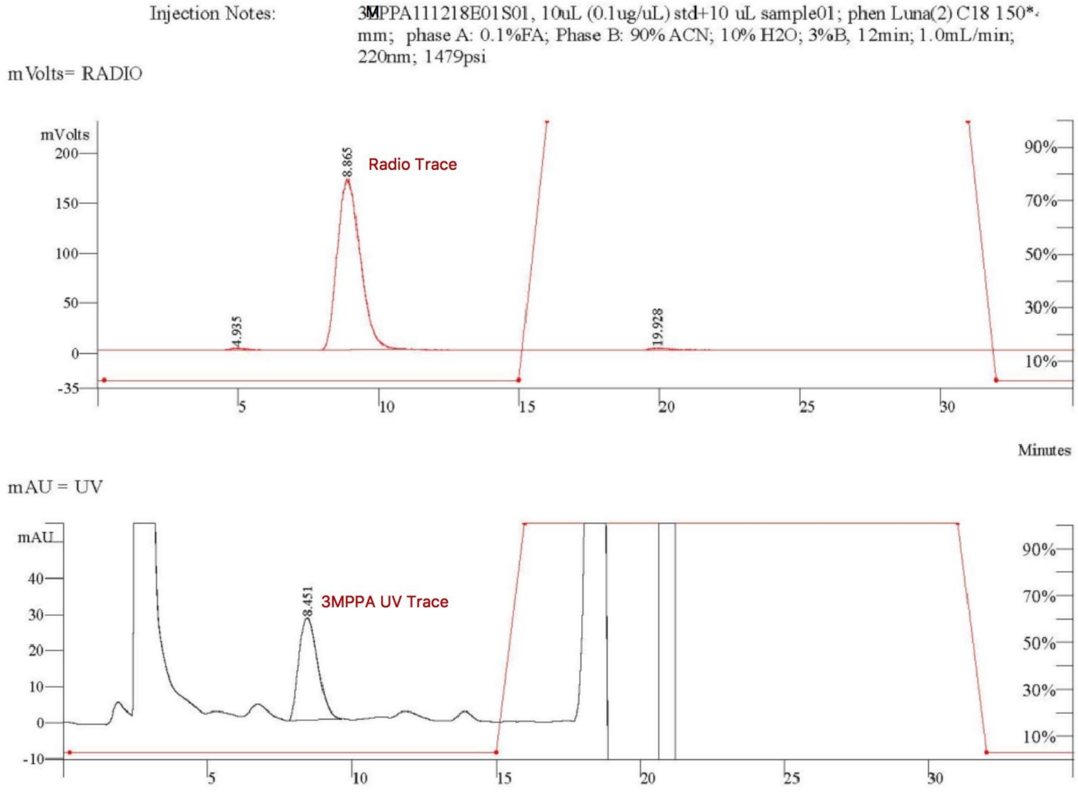

<u>6-Chlorohex-2-ynoic-1-[^{11}C]acid, ([^{11}C]7j) synthesis</u>

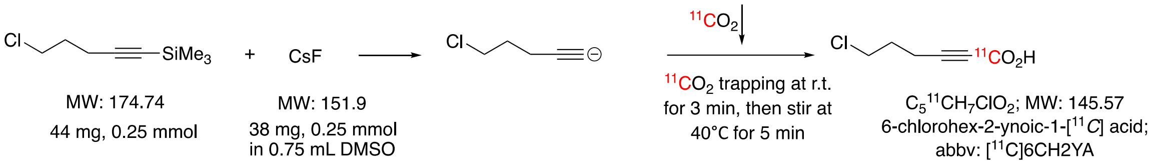

Following <u>the procedures of pre-labeling work</u> and <u>^{11}C-carboxylation reaction</u> described above, [^{11}C]6CH2YA, **[^{11}C]7j**, was synthesized (see above scheme for details) and the detailed results are listed in the table below:

Supplementary Table 12. Summarization of **[^{11}C]7j** synthesis experimental results

No.	RCP and RCY
Exp 01	68.1% & 62.1%
Exp 02	88.8% & 88.1%
Exp 03	80.0% & 80.0%
Avg ± SD of RCP and RCY	79.0 ± 10.4% & 76.7 ± 10.4%

Supplementary Figure 12. A [^{11}C]6CH2YA, **[^{11}C]7j,** crude product sample analytical HPLC profile

HPLC analysis conditions: stationary phase: Phenomenex Luna(2) C18 150 × 4.6 mm; Mobile phase: phase A (0.1% formic acid), phase B (90% CH_3CN/10% H_2O); UV: 215 nm; Flow rate: 1.2 mL/min; Gradient/isocratic method: 25%B/75%A (0-12 min), rise 25%B to 100%B (12-13 min), 100%B (13-20 min).

Description: 6CH2YA021519E02S01, 20 uL of mixture (20 uL 1.0 ug/uL std +180 uL Sample01), Phen Luna(2) C18 150*4.6 mm; Phase A: 0.1% formic acid; Phase B: 90%ACN, 10% H2O; 25%B, 12 min; 1.2mL/min; 215nm; 117.8bar

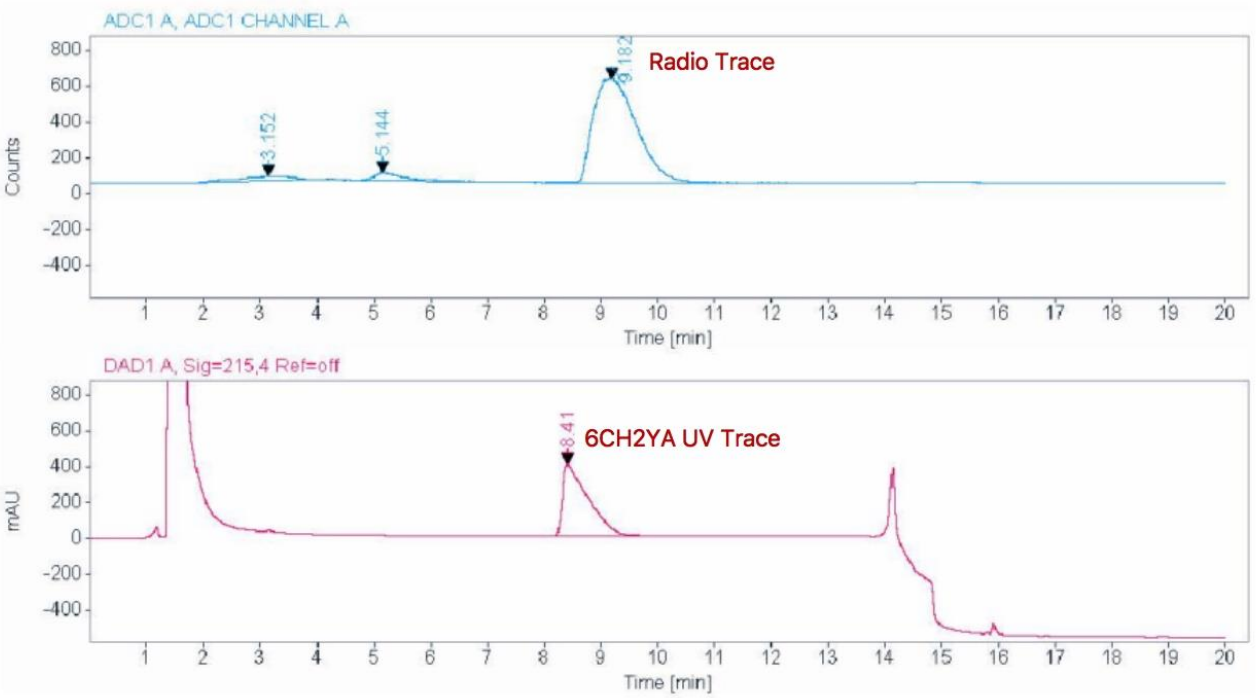

3-(Ethoxycarbonyl)propiolic-1-[^{11}C]acid, ([^{11}C]7k) synthesis

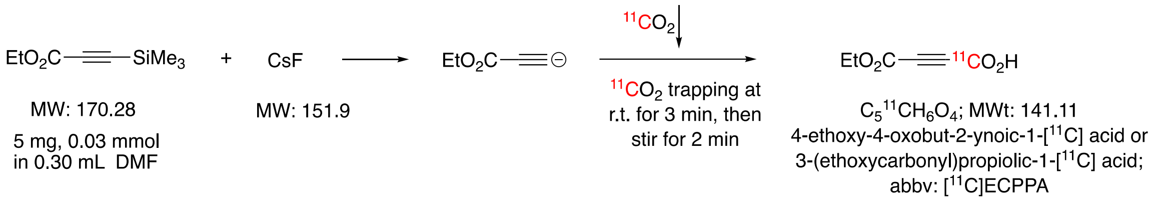

Following the procedures of pre-labeling work and [^{11}C] carboxylation reaction described above, [^{11}C]ECPPA, **[^{11}C]7k**, was synthesized (see above scheme for details) and the detailed results are listed in the table below:

Supplementary Table 13. Summarization of **[^{11}C]7k** synthesis experimental results

No.	RCP and RCY
Exp 01	>99% & 79.2%
Exp 02	>99% & 93.3%
Exp 03	>99% & 90.6%
Avg ± SD of RCP and RCY	>99 ± 0.0% & 87.7 ± 7.5%

Supplementary Figure 13. An [^{11}C]ECPPA, **[^{11}C]7k,** crude product sample analytical HPLC profile

HPLC analysis conditions: stationary phase: Phenomenex Luna(2) C18 150 × 4.6 mm; Mobile phase: phase A (0.1% formic acid), phase B (90% CH_3CN/10% H_2O); UV: 215 nm; Flow rate: 1.5 mL/min; Gradient/isocratic method: 10%B/90%A (0-14 min), rise 10%B to 100%B (14-15 min), 100%B (15-23 min).

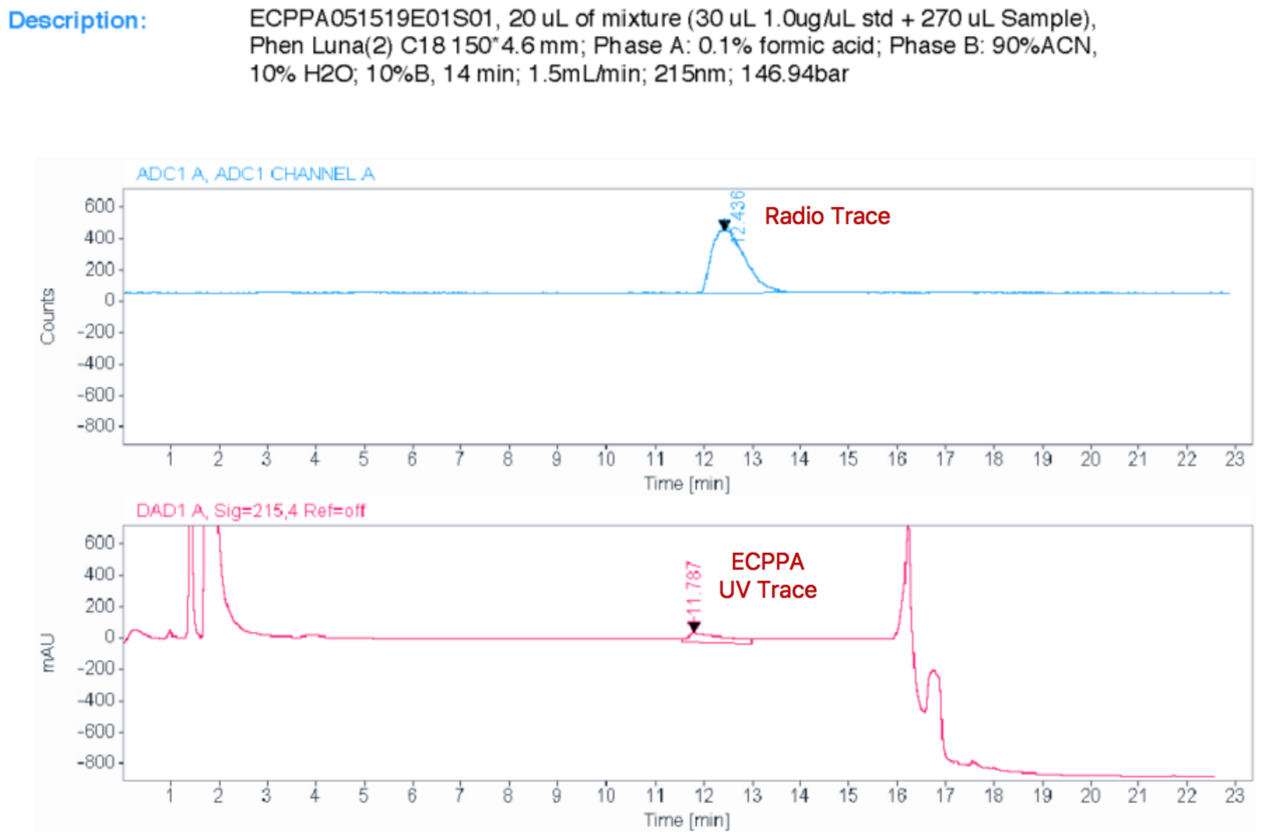

Methyl 3-(pyridin-2-yl)propiolate-1-[11C], ([11C]8) synthesis

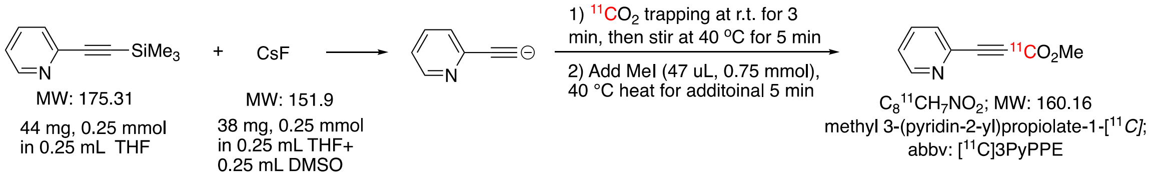

Following the procedures of pre-labeling work and 11C-carboxylation reaction described above, methyl 3-(pyridin-2-yl)(1-[11C])propiolate ([11C]3PyPPE), **[11C]8**, was synthesized (see above scheme for details) and the detailed results are listed in the table below:

Supplementary Table 14. Summarization of **[11C]8** synthesis experimental results

No.	RCP and RCY
Exp 01	25.5% & 25.0%
Exp 02	29.5% & 29.0%
Exp 03	30.0% & 30.0%
Avg ± SD of RCP and RCY	28.3 ± 2.5% & 28.0 ± 2.6%

Supplementary Figure 14. A [11C]3PyPPE, **[11C]8,** crude product sample analytical HPLC profile

HPLC analysis conditions: stationary phase: Phenomenex Luna(2) C18 150 × 4.6 mm; Mobile phase: phase A (0.1% formic acid), phase B (90% CH_3CN/10% H_2O); UV: 254 nm: Flow rate: 1.0 mL/min; Gradient/isocratic method: 35%B/65%A (0-12 min), rise 35%B to 100%B (12-13 min), 100%B (13-25 min).

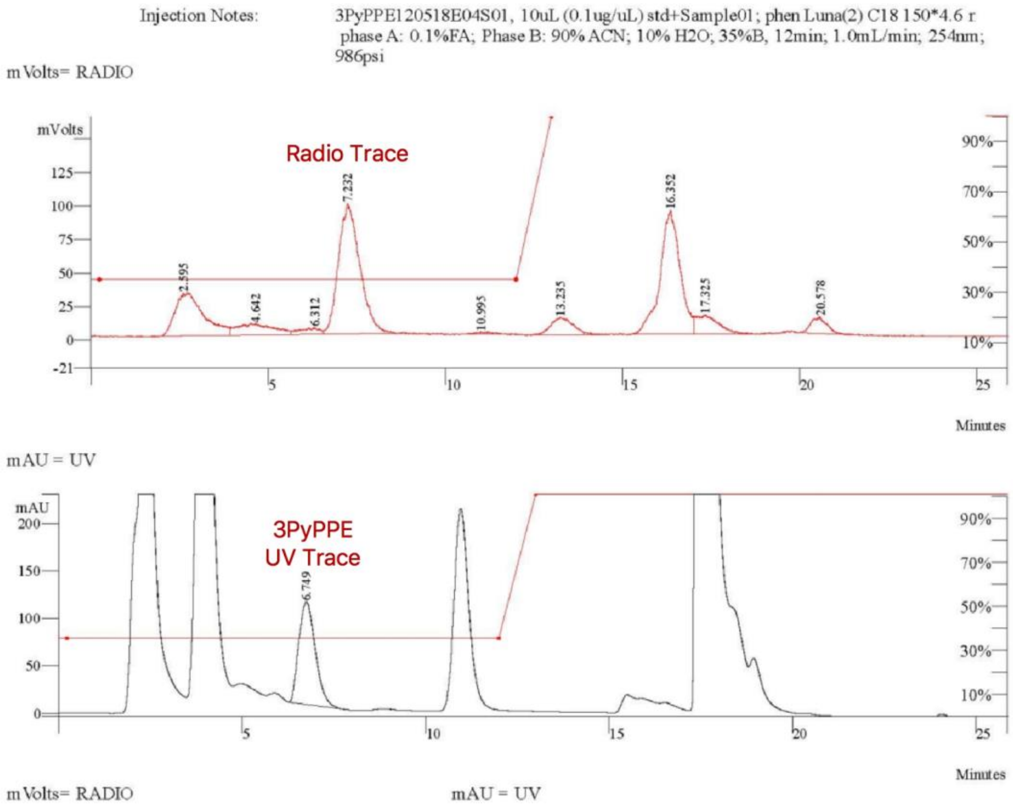

N-Benzyl-3-(3-chlorophenyl)propiolamide-[1-^{11}C], ([^{11}C]9) synthesis

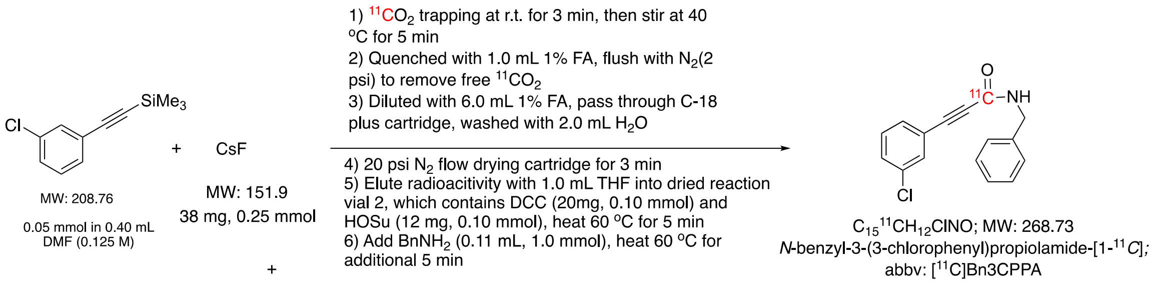

Following the procedures of pre-labeling work and ^{11}C-carboxylation reaction described above, *N*-benzyl-3-(3-chlorophenyl)(1-[^{11}C])propiolamide ([^{11}C]Bn3CPPA), **[^{11}C]9**, was synthesized (see above scheme for details) and the detailed results are listed in the table below:

Supplementary Table 15. Summarization of **[^{11}C]9** synthesis experimental results

No.	RCP and RCY
Exp 01	40.7% & 31.1%
Exp 02	23.8% & 15.9%
Exp 03	22.0% & 14.8%
Avg ± SD of RCP and RCY	28.8 ± 10.3% & 20.6 ± 9.1%

Supplementary Figure 15. A [^{11}C]Bn3CPPA, **[^{11}C]9,** crude product sample analytical HPLC profile

HPLC analysis conditions: stationary phase: Phenomenex Luna(2) C18 150 × 4.6 mm; Mobile phase: phase A (0.1% formic acid), phase B (90% CH_3CN/10% H_2O); UV: 254 nm; Flow rate: 1.0 mL/min; Gradient/isocratic method: 65%B/35%A (0-12 min), rise 65%B to 100%B (12-13 min), 100%B (13-20 min).

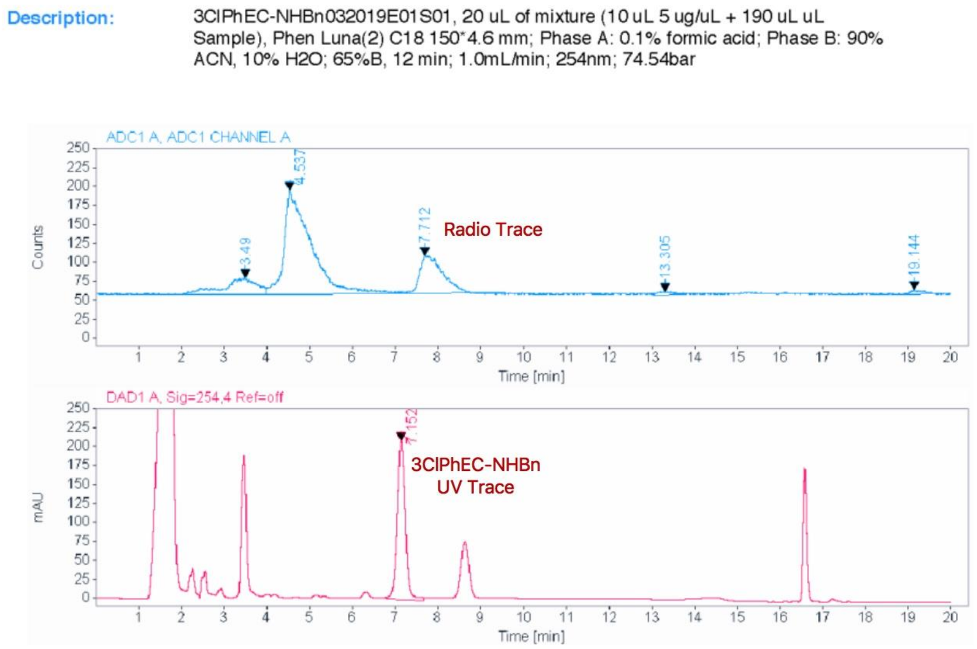

FMDS ^{11}C-carboxylation with sp^2-hybridized carbon attached trimethylsilanes

3-Oxo-3-phenylpropanoic-1-[^{11}C]acid ([^{11}C]OPPA, [^{11}C]11a) synthesis – FMDS approach

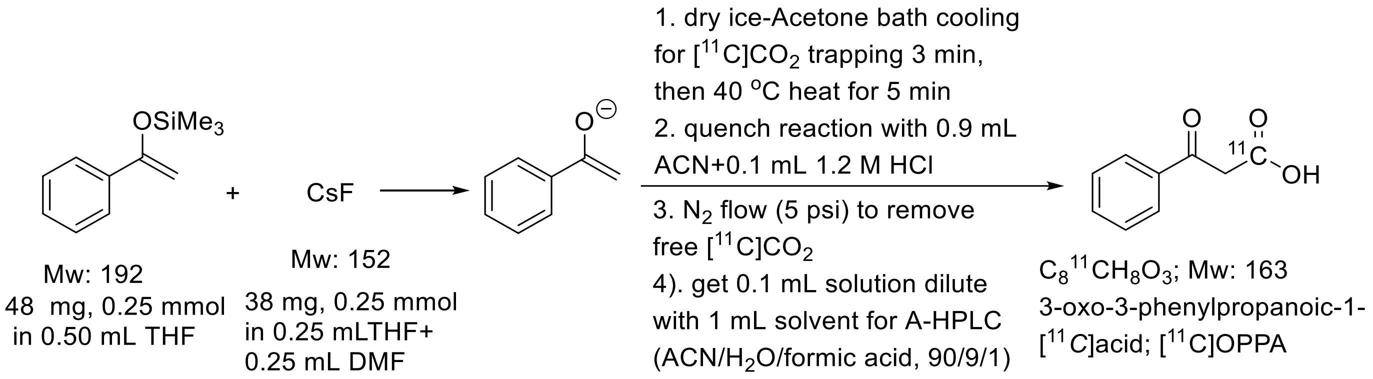

Following the procedures of pre-labeling work and ^{11}C-carboxylation reaction described in the synthesis of [^{11}C]AcAc, 3-Oxo-3-phenylpropanoic-1-[^{11}C]acid ([^{11}C]OPPA), **[^{11}C]11a**, was synthesized (see above scheme for details) and the detailed results are listed in the table below:

Supplementary Table 16. Summarization of **[^{11}C]11a** synthesis experimental results

No.	RCP and RCY
Exp 01	87.9% & 55.6%
Exp 02	96.0% & 72.4%
Exp 03	97.9% & 85.8%
Avg ± SD of RCP & RCY	93.9 ± 5.3% & 71.3 ± 15.1%

Supplementary Figure 16. A [^{11}C]OPPA, **[^{11}C]11a,** crude product sample analytical HPLC profile

HPLC analysis conditions: stationary phase: Phenomenex Luna C18 150 × 4.6 mm; Mobile phase: phase A (0.1% formic acid), phase B (100% CH_3CN); UV: 254 nm; Flow rate: 1.5 mL/min; Gradient/isocratic method: 25%B/75%A (0-10 min), rise 25%B to 90%B (10-11 min), 90%B (11-20 min), drop 90%B to 25%B (20-22 min).

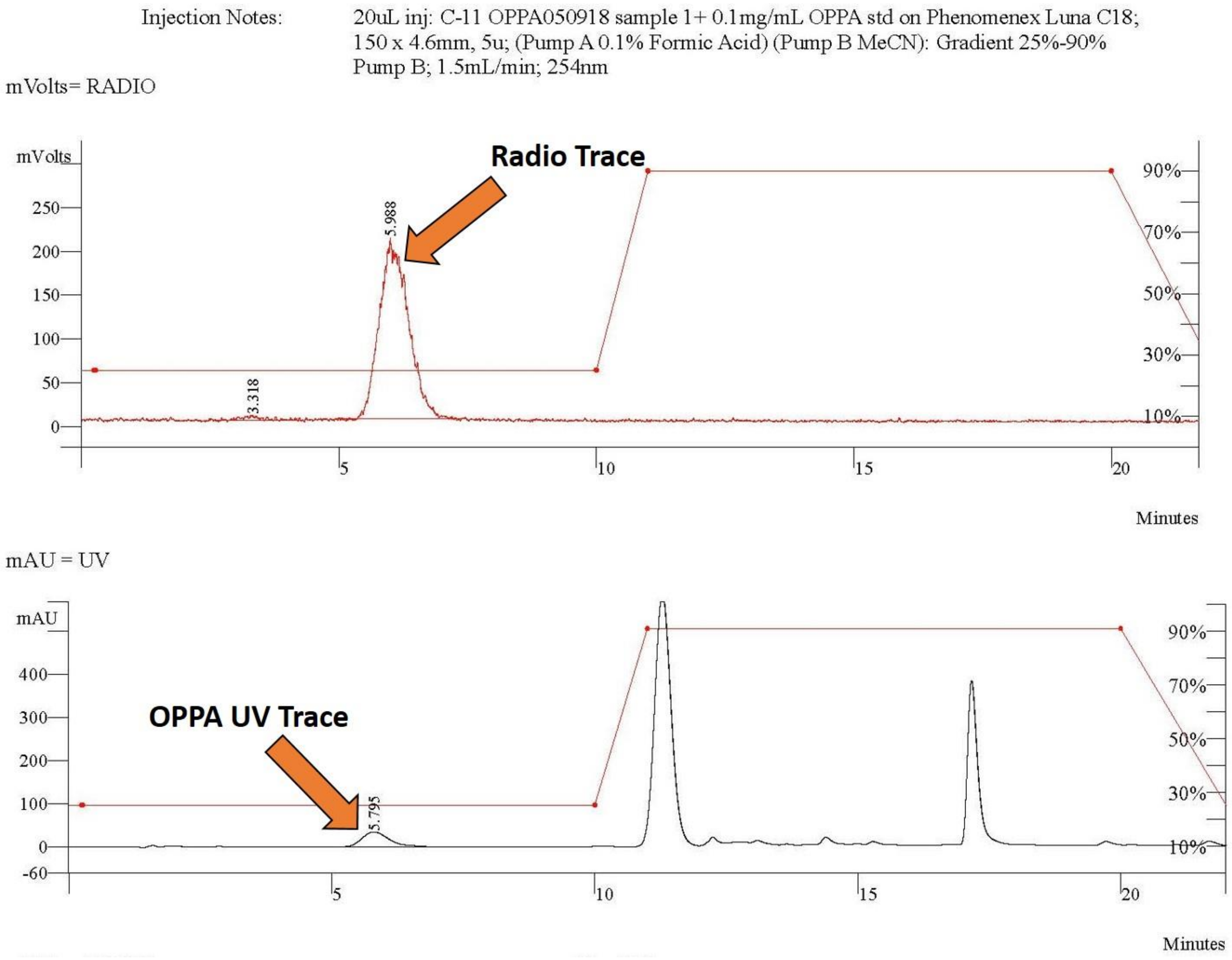

2,3-Difluoro-6-bromobenzoic-1-[11C]acid, ([11C]11b) synthesis

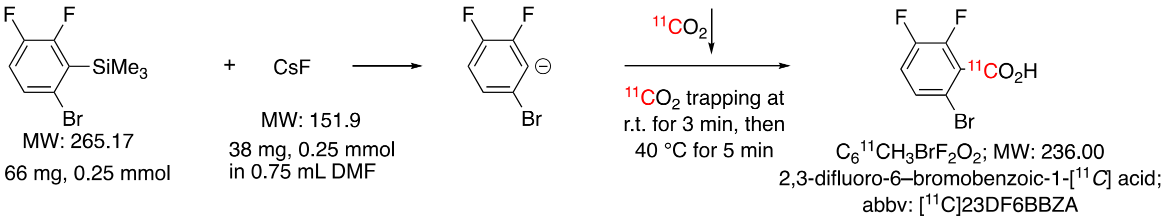

Following the procedures of pre-labeling work and ^{11}C-carboxylation reaction described above, [^{11}C]23DF6BBZA, **[^{11}C]11b**, was synthesized (see above scheme for details) and the detailed results are listed in the table below:

Supplementary Table. 17. Summarization of **[^{11}C]11b** synthesis experimental results

No.	RCP and RCY
Exp 01	>99% & 93.0%
Exp 02	>99% & 80.0%
Exp 03	92.0% & 84.8%
Avg ± SD of RCP & RCY	96.7 ± 4.0% & 85.9 ± 6.6%

Supplementary Figure 17. A [^{11}C]23DF6BBZA, **[^{11}C]11b,** crude product sample analytical HPLC profile

HPLC analysis conditions: stationary phase: Phenomenex Luna(2) C18 150 × 4.6 mm; Mobile phase: phase A (0.1% formic acid), phase B (90% CH_3CN/10% H_2O); UV: 215 nm; Flow rate: 1.0 mL/min; Gradient/isocratic method: 40%B/60%A (0-12 min), rise 40%B to 100%B (12-13 min), 100%B (13-23 min).

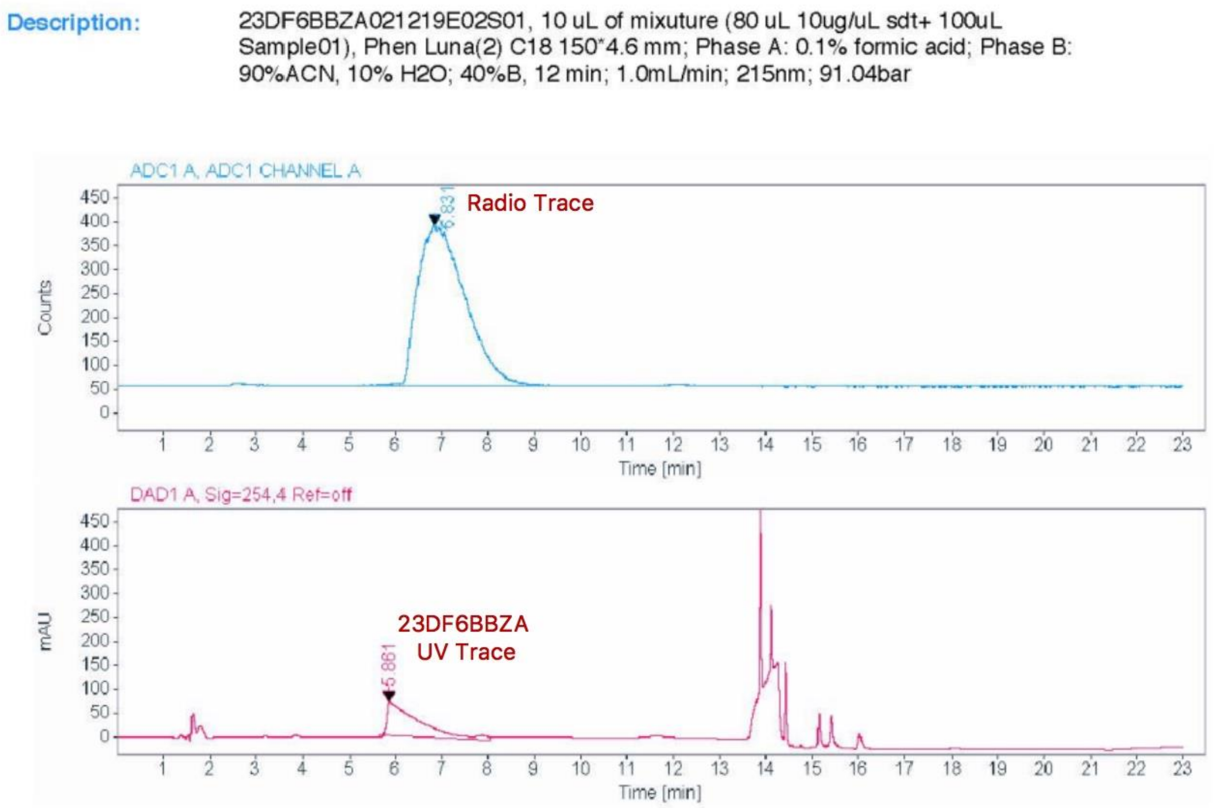

2-Fluoro-6-bromobenzoic-1-[^{11}C]acid, ([^{11}C]11c) synthesis

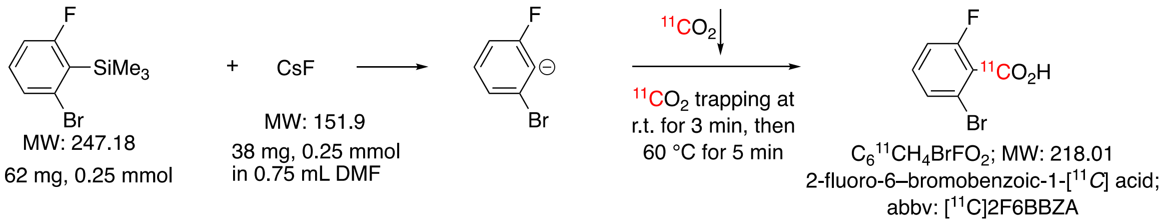

Following the procedures of pre-labeling work and ^{11}C-carboxylation reaction described above, [^{11}C]2F6BBZA, **[^{11}C]11c**, was synthesized (see above scheme for details) and the detailed results are listed in the table below:

Supplementary Table 18. Summarization of **[^{11}C]11c** synthesis experimental results

No.	RCP and RCY
Exp 01	97.8% & 89.1%
Exp 02	97.6% & 80.6%
Exp 03	97.7% & 84.0%
Avg ± SD of RCP & RCY	97.7 ± 0.1% & 84.6 ± 4.3%

Supplementary Figure 18. A [^{11}C]2F6BBZA, **[^{11}C]11c,** crude product sample analytical HPLC profile

HPLC analysis conditions: stationary phase: Phenomenex Luna(2) C18 150 × 4.6 mm; Mobile phase: phase A (0.1% formic acid), phase B (90% CH_3CN/10% H_2O); UV: 254 nm; Flow rate: 1.0 mL/min; Gradient/isocratic method: 40%B/60%A (0-10 min), rise 40%B to 100%B (10-11 min), 100%B (11-18 min), drop 100% to 40%B (18-19 min),40%B (19-22 min).

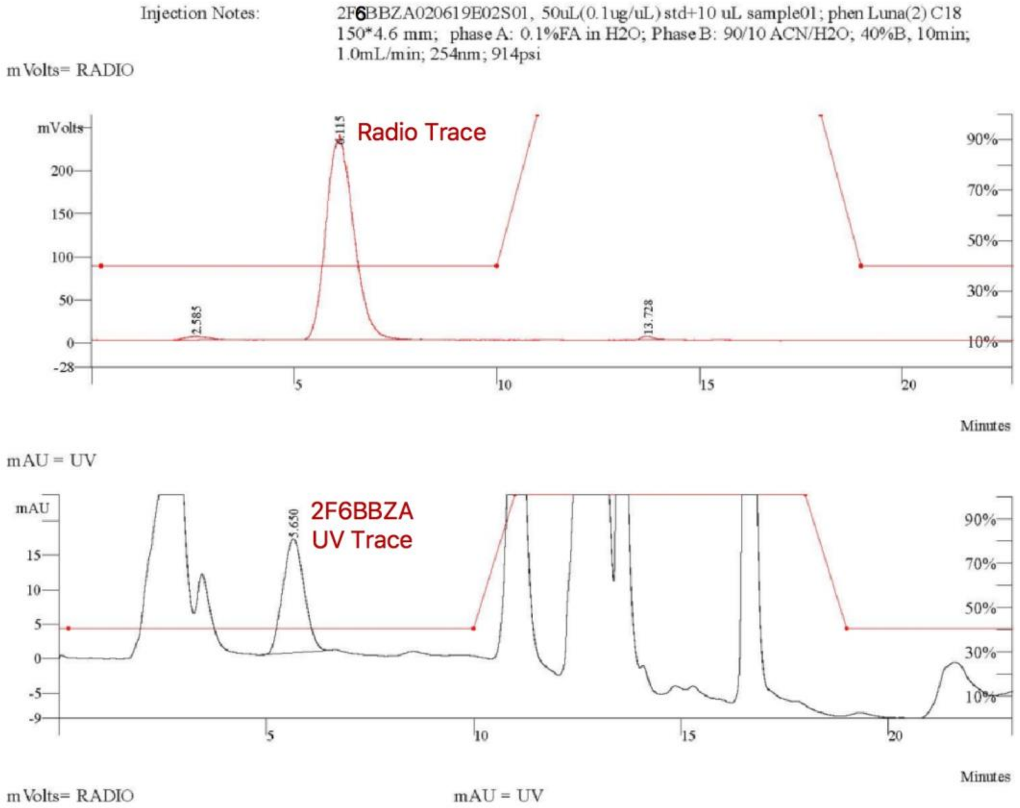

3,5-Dibromobenzoic-1-[^{11}C]acid, ([^{11}C]11d) synthesis

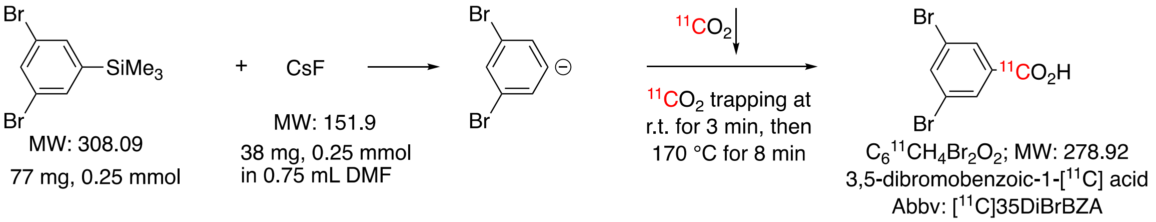

Following the procedures of pre-labeling work and ^{11}C-carboxylation reaction described above, [^{11}C]35DiBrBZA, **[^{11}C]11d**, was synthesized (see above scheme for details) and the detailed results are listed in the table below:

Supplementary Table 19. Summarization of **[^{11}C]11d** synthesis experimental results

No.	RCP and RCY
Exp 01	>99% & 20.5%
Exp 02	91.2% & 19.1%
Exp 03	>99% & 16.9%
Avg ± SD of RCP & RCY	96.4 ± 4.5% & 18.8 ± 1.8%

Supplementary Figure 19. A [^{11}C]35DiBrBZA, **[^{11}C]11d,** crude product sample analytical HPLC profile

HPLC analysis conditions: stationary phase: Phenomenex Luna(2) C18 150 × 4.6 mm; Mobile phase: phase A (0.1% formic acid), phase B (90% CH_3CN/10% H_2O); UV: 254 nm; Flow rate: 1.0 mL/min; Gradient/isocratic method: 50%B/50%A (0-12 min), rise 50%B to 100%B (12-13 min), 100%B (13-25 min).

Description: 35diBrBZA041019E03S01, 20 uL of mixture (20 uL 10.0 ug/uL std+180 uL sample), Phen Luna(2) C18 150*4.6 mm; Phase A: 0.1% formic acid; Phase B: 90%ACN, 10% H2O; 50%B, 12 min; 1.0mL/min; 254nm; 86.04bar

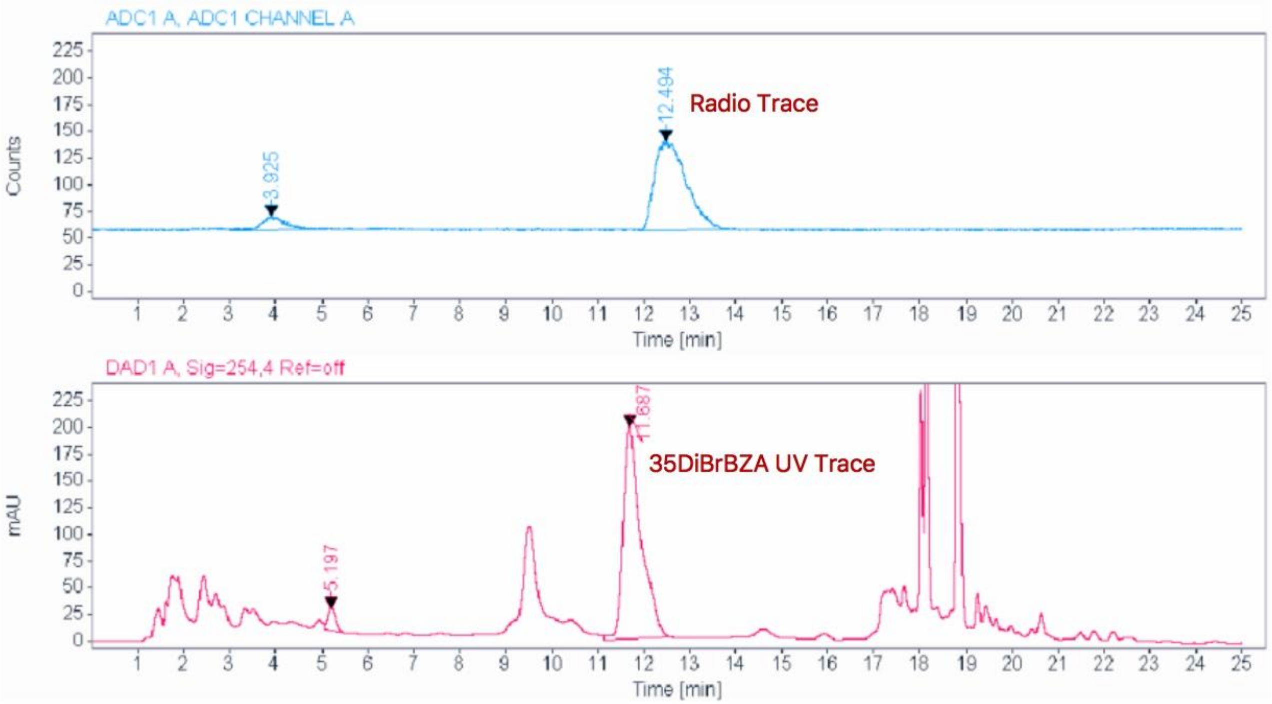

3-Bromobenzoic-1-[^{11}C]acid, ([^{11}C]11e) synthesis

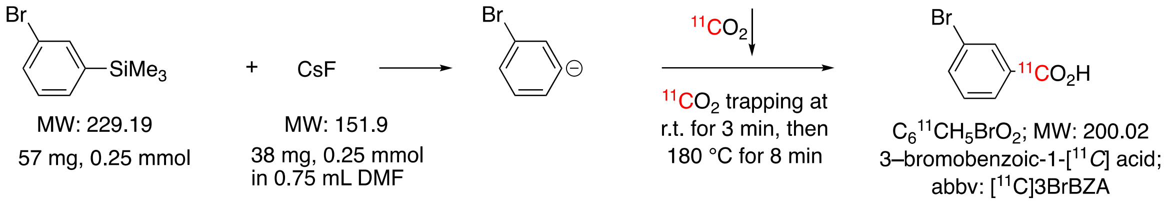

Following the procedures of pre-labeling work and ^{11}C-carboxylation reaction described above, [^{11}C]3BrBZA, **[^{11}C]11e**, was synthesized (see above scheme for details) and the detailed results are listed in the table below:

Supplementary Table 20. Summarization of **[^{11}C]11e** synthesis experimental results

No.	RCP and RCY
Exp 01	77.7% & 26.9%
Exp 02	73.3% & 12.9%
Exp 03	78.8% & 18.4%
Avg ± SD of RCP and RCY	76.5 ± 2.8% & 19.4 ± 7.1%

Supplementary Figure 20. A [^{11}C]3BrBZA, **[^{11}C]11e,** crude product sample analytical HPLC profile

HPLC analysis conditions: stationary phase: Phenomenex Luna(2) C18 150 × 4.6 mm; Mobile phase: phase A (0.1% formic acid), phase B (90% CH_3CN/10% H_2O); UV: 254 nm; Flow rate: 1.0 mL/min; Gradient/isocratic method: 40%B/60%A (0-12 min), rise 40%B to 100%B (12-13 min), 100%B (13-21 min).

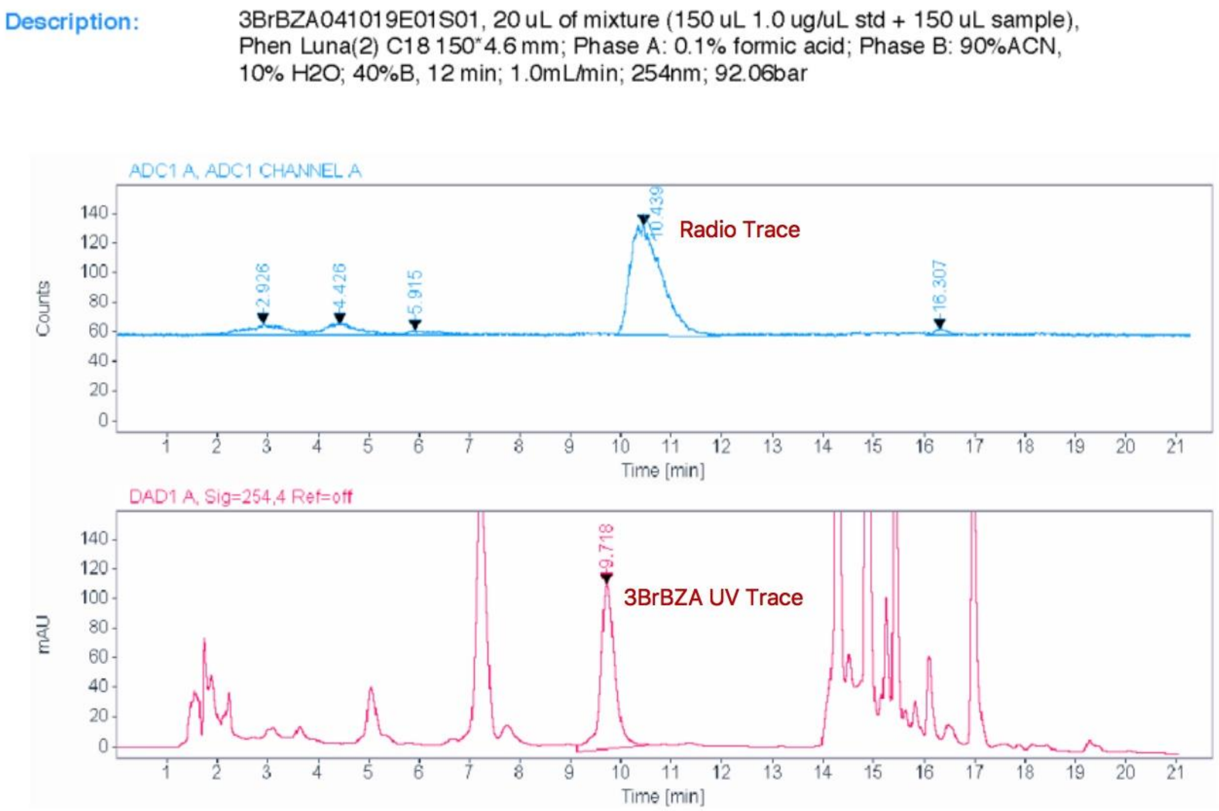

4-Bromobenzoic-1-[^{11}C]acid, ([^{11}C]11f) synthesis

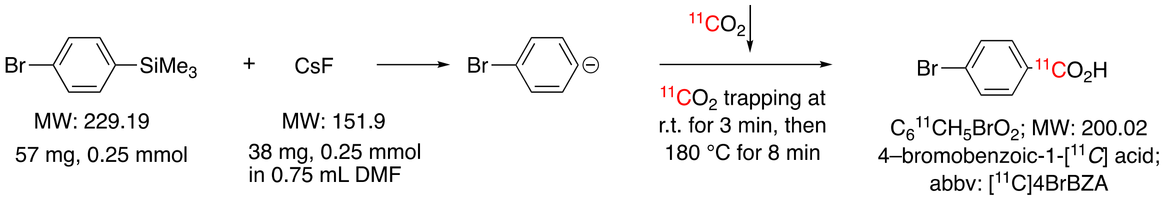

Following the procedures of pre-labeling work and ^{11}C-carboxylation reaction described above, [^{11}C]4BrBZA, **[^{11}C]11f**, was synthesized (see above scheme for details) and the detailed results are listed in the table below:

Supplementary Table 21. Summarization of **[^{11}C]11f** synthesis experimental results

No.	RCP and RCY
Exp 01	78.1% & 13.2%
Exp 02	83.6% & 9.8%
Exp 03	82.6% & 10.3%
Avg ± SD of RCP and RCY	81.4 ± 2.9% & 11.1 ± 1.8%

Supplementary Figure 21. A [^{11}C]4BrBZA, **[^{11}C]11f,** crude product sample analytical HPLC profile

HPLC analysis conditions: stationary phase: Phenomenex Luna(2) C18 150 × 4.6 mm; Mobile phase: phase A (0.1% formic acid), phase B (90% CH_3CN/10% H_2O); UV: 254 nm; Flow rate: 1.0 mL/min; Gradient/isocratic method: 50%B/60%A (0-12 min), rise 50%B to 100%B (12-13 min), 100%B (13-23 min).

Description: 4BrBZA040919E01S01, 20 uL of mixture (4 uL 10 ug/uL std + 196 uL sample), Phen Luna(2) C18 150*4.6 mm; Phase A: 0.1% formic acid; Phase B: 90%ACN, 10% H2O; 50%B, 12 min; 1.0mL/min; 254nm; 88.05bar

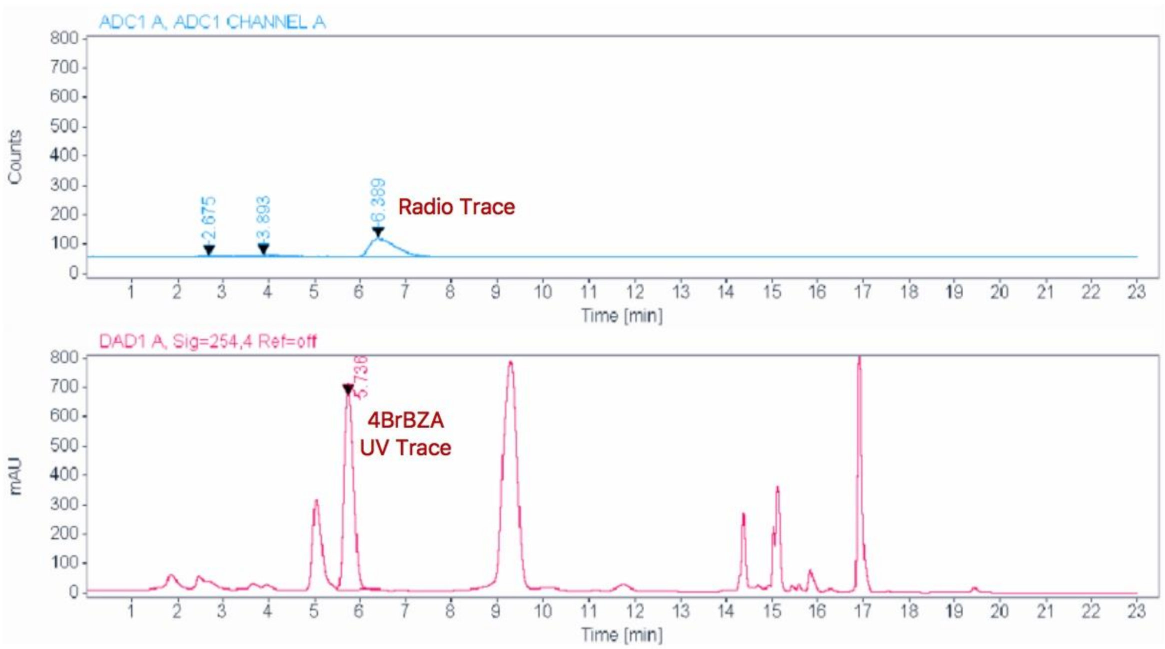

Benzoic-1-[^{11}C]acid, ([^{11}C]11g) synthesis

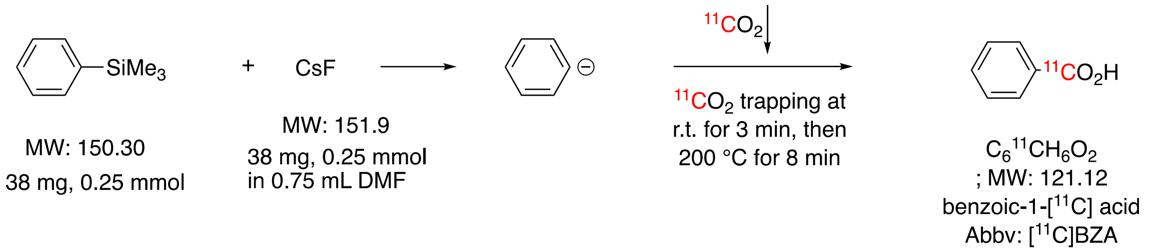

Following the procedures of pre-labeling work and ^{11}C-carboxylation reaction described above, [^{11}C]BZA, **[^{11}C]11g**, was synthesized (see above scheme for details) and the detailed results are listed in the table below:

Supplementary Table 22. Summarization of **[^{11}C]11g** synthesis experimental results

No.	RCP and RCY
Exp 01	30.1% & 1.4%

Supplementary Figure 22. A [^{11}C]BZA, **[^{11}C]11g,** crude product sample analytical HPLC profile

HPLC analysis conditions: stationary phase: Phenomenex Luna(2) C18 150 × 4.6 mm; Mobile phase: phase A (0.1% formic acid), phase B (90% CH_3CN/10% H_2O); UV: 254 nm; Flow rate: 1.0 mL/min; Gradient/isocratic method: 35%B/75%A (0-12 min), rise 35%B to 100%B (12-13 min), 100%B (13-23 min).

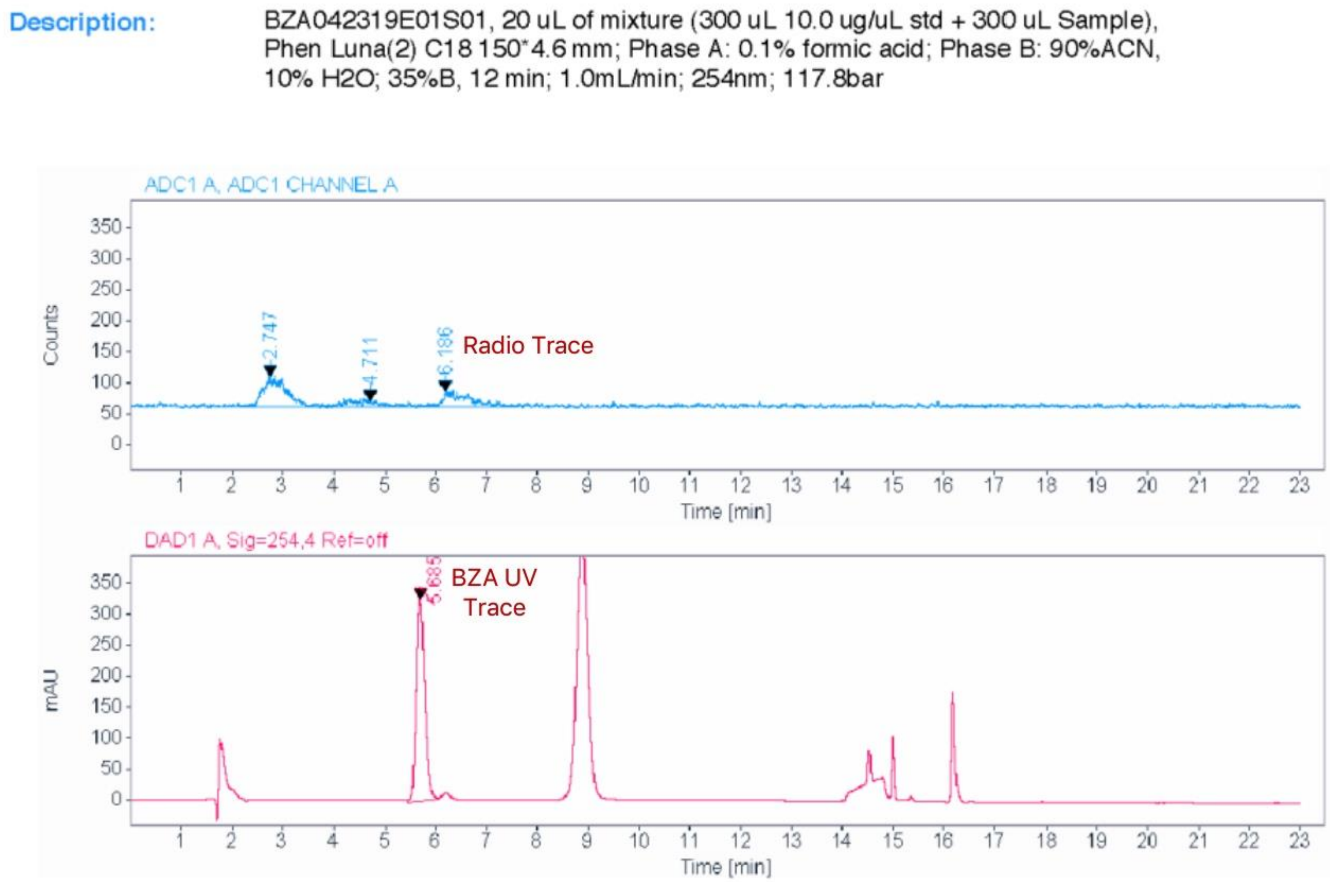

Naphthoic-1-[^{11}C]acid, ([^{11}C]11h) synthesis

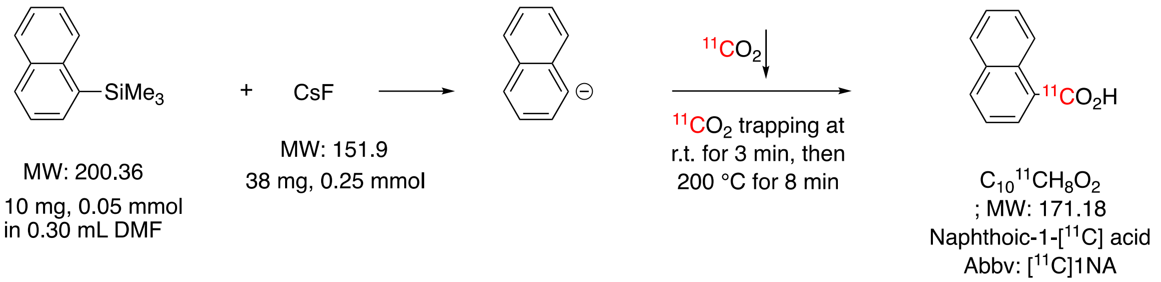

Following the procedures of pre-labeling work and ^{11}C-carboxylation reaction described above, [^{11}C]1NA, **[^{11}C]11h**,was synthesized (see above scheme for details) and the detailed results are listed in the table below:

Supplementary Table 23. Summarization of **[^{11}C]11h** synthesis experimental results

No.	RCP and RCY
Exp 01	71.8% & 9.3%
Exp 02	82.7% & 3.7%
Exp 03	56.8% & 2.7%
Exp 04	75.2% & 4.2%
Avg ± SD of RCP and RCY	71.6 ± 9.4% & 5.0 ± 2.6%

Supplementary Figure 23. A [^{11}C]1NA, **[^{11}C]11h,** crude product sample analytical HPLC profile

HPLC analysis conditions: stationary phase: Phenomenex Luna(2) C18 150 × 4.6 mm; Mobile phase: phase A (0.1% formic acid), phase B (90% CH_3CN/10% H_2O); UV: 254 nm; Flow rate: 1.0 mL/min; Gradient/isocratic method: 40%B/60%A (0-12 min), rise 40%B to 100%B (12-13 min), 100%B (13-23 min).

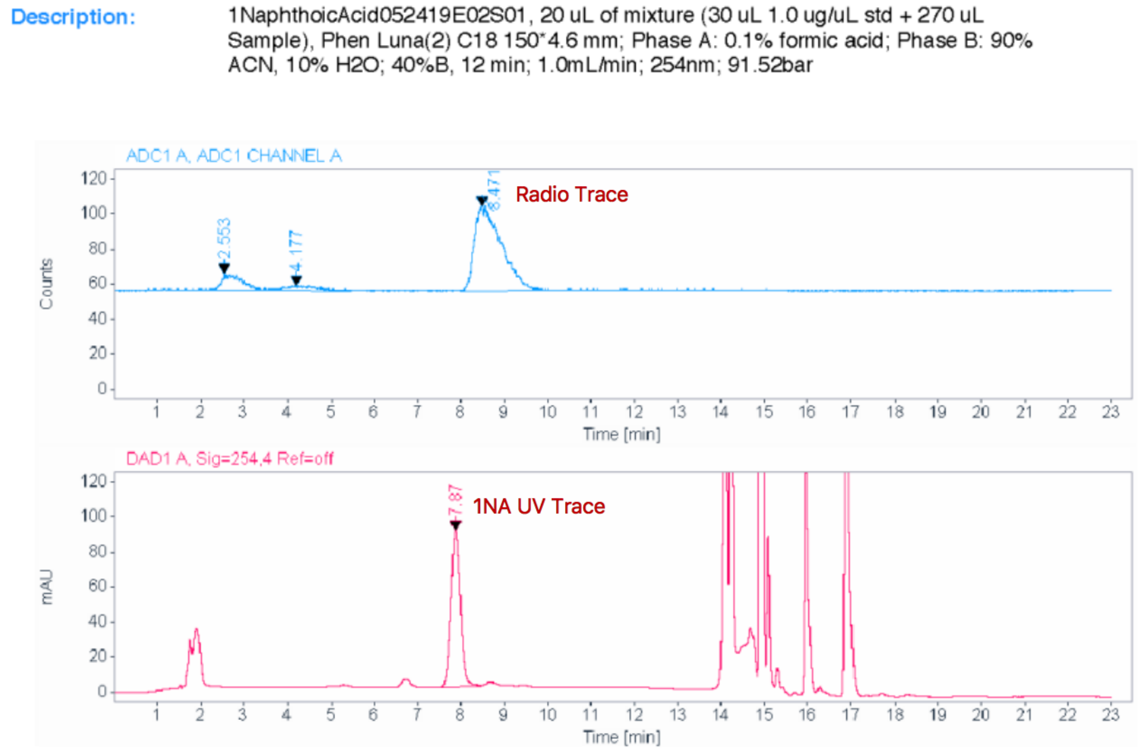

3-Fluoro-4-pyridinecarboxylic-[^{11}C]acid, ([^{11}C]11i) synthesis

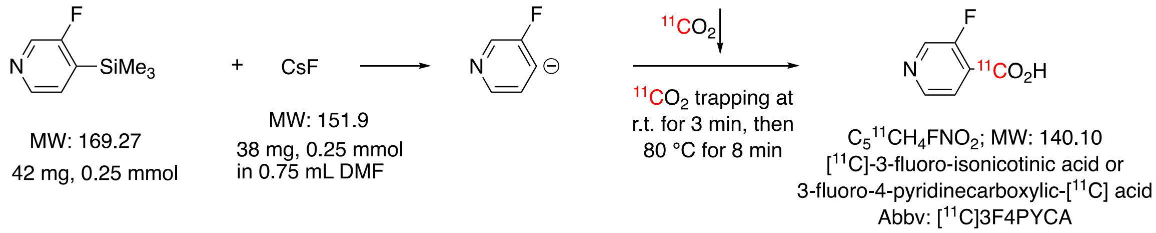

Following the procedures of pre-labeling work and ^{11}C-carboxylation reaction described above, [^{11}C]3F4PYCA, **[^{11}C]11i**, was synthesized (see above scheme for details) and the detailed results are listed in the table below:

Supplementary Table 24. Summarization of **[^{11}C]11i** synthesis experimental results

No.	RCP and RCY
Exp 01	93.9% & 26.3%
Exp 02	79.8% & 20.4%
Exp 03	76.1% & 14.3%
Avg ± SD of RCP and RCY	83.3 ± 9.4% & 20.3 ± 6.0%

Supplementary Figure 24. A [^{11}C]3F4CPYCA, **[^{11}C]11i,** crude product sample analytical HPLC profile

HPLC analysis conditions: stationary phase: Phenomenex Luna NH_2 150 × 4.6 mm; Mobile phase: phase A 100 mM NH_4OH + 50 mM HOAc (PH = 9.0), phase B (90% CH_3CN/10% H_2O); UV: 264 nm; Flow rate: 1.0 mL/min; Gradient/isocratic method: 70%B/30%A (0-12 min), rise 30%A to 100%A (12-13 min), 100%A (13-25 min).

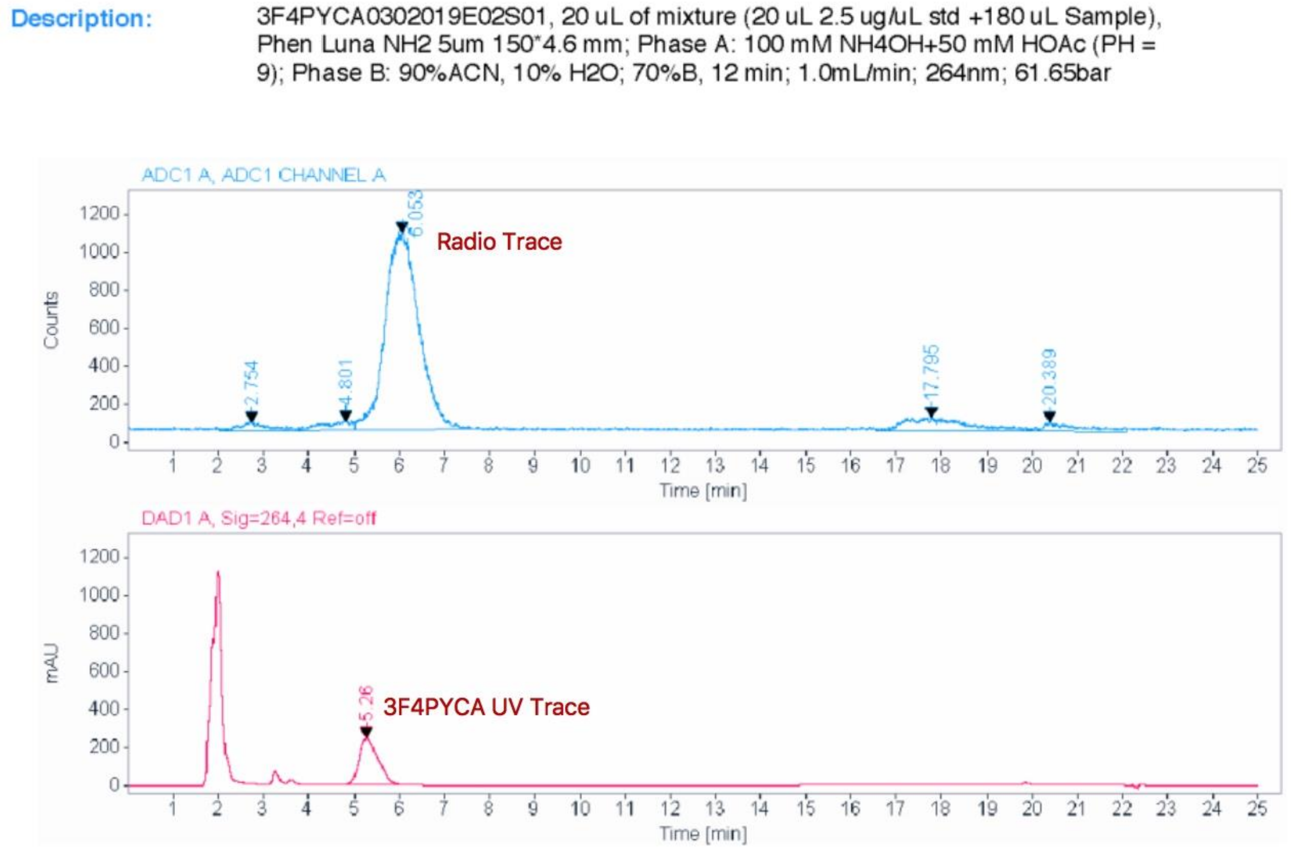

4-Chloro-2-pyridinecarboxylic-[^{11}C]acid, ([^{11}C]11j) synthesis

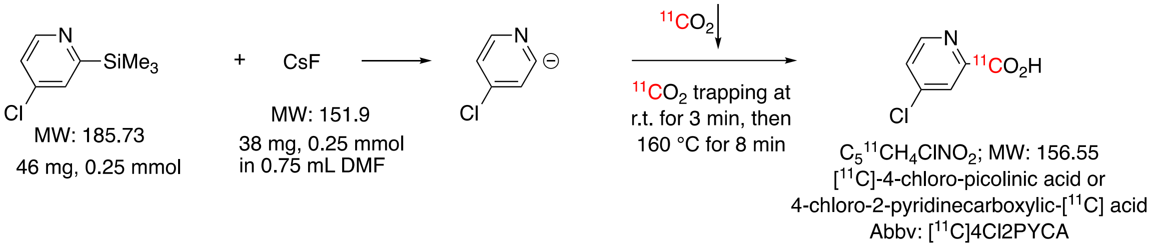

Following the procedures of pre-labeling work & ^{11}C-carboxylation reaction described above, [^{11}C]4Cl2PYCA, **[^{11}C]11j**, was synthesized (see above scheme for details) and the detailed results are listed in the table below:

Supplementary Table 25. Summarization of **[^{11}C]11j** synthesis experimental results

No.	RCP and RCY
Exp 01	83.6% & 25.0%
Exp 02	76.5% & 21.1%
Exp 03	78.7% & 22.3%
Avg ± SD of RCP and RCY	79.6 ± 3.6% & 22.8 ± 2.0%

Supplementary Figure 25. A [^{11}C]4Cl2PYCA, **[^{11}C]11j,** crude product sample analytical HPLC profile

HPLC analysis conditions: stationary phase: Phenomenex Luna(2) C18 150 × 4.6 mm; Mobile phase: phase A (0.1% formic acid), phase B (90% CH_3CN/10% H_2O); UV: 254 nm; Flow rate: 1.2 mL/min; Gradient/isocratic method: 5%B/95%A (0-15 min), rise 5%B to 100%B (15-16 min), 100%B (16-22 min).

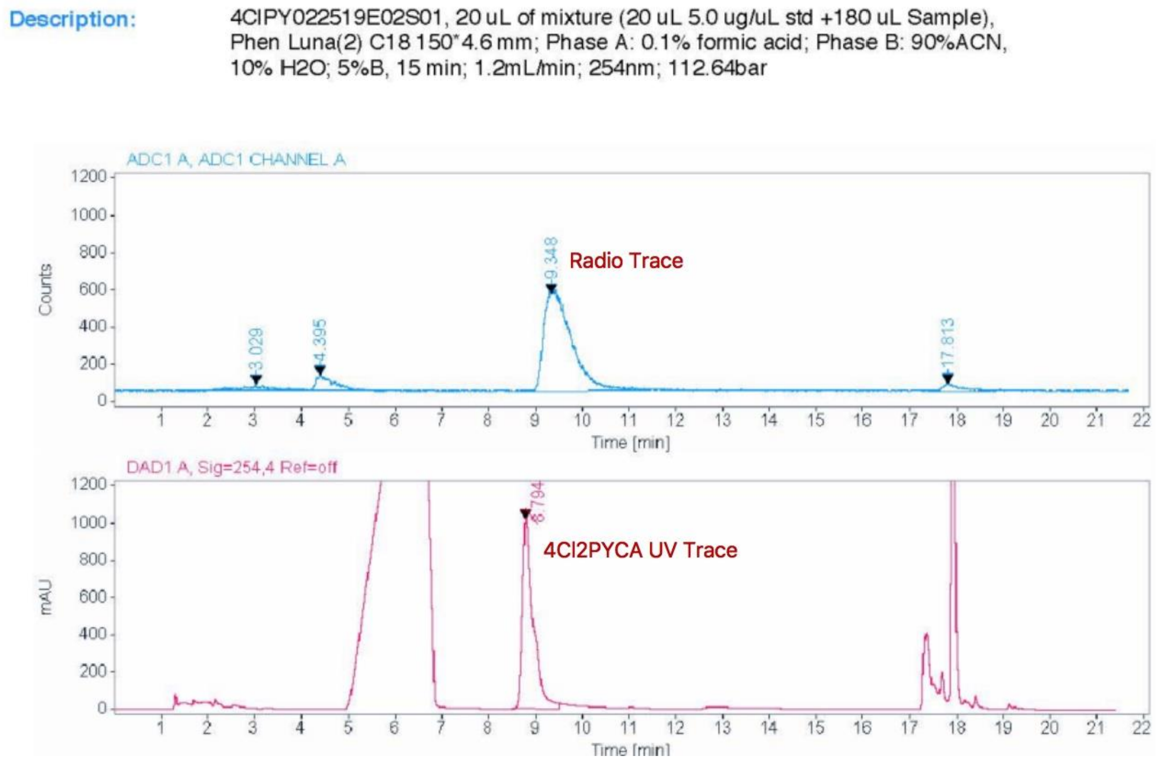

2-Pyridinecarboxylic-[^{11}C]acid, ([^{11}C]11k) synthesis

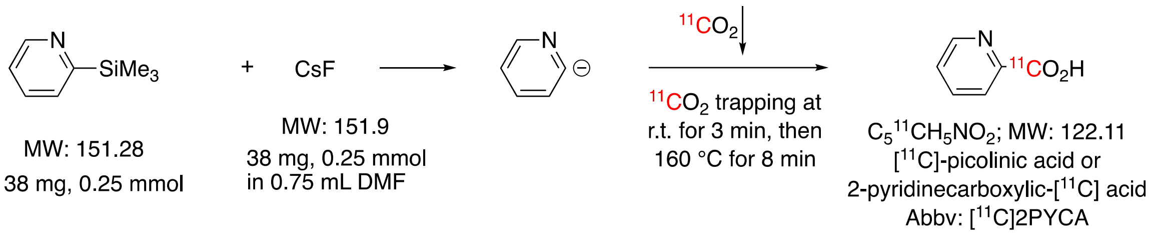

Following the procedures of pre-labeling work and ^{11}C-carboxylation reaction described above, [^{11}C]2PYCA, **[^{11}C]11k**, was synthesized (see above scheme for details) and the detailed results are listed in the table below:

Supplementary Table 26. Summarization of **[^{11}C]11k** synthesis experimental results

No.	RCP and RCY
Exp 01	90.4% & 15.3%
Exp 02	89.9% & 13.5%
Exp 03	87.7% & 10.7%
Avg ± SD of RCP and RCY	89.3 ± 1.4% & 13.2 ± 2.3%

Supplementary Figure 26. A [^{11}C]2PYCA, **[^{11}C]11k,** crude product sample analytical HPLC profile

HPLC analysis conditions: stationary phase: Phenomenex Luna NH_2 150 × 4.6 mm; Mobile phase: phase A 100 mM NH_4OH + 50 mM HOAc (PH = 9.0), phase B (90% CH_3CN/10% H_2O); UV: 264 nm; Flow rate: 1.0 mL/min; Gradient/isocratic method: 65%B/35%A (0-12 min), rise 35%A to 100%A (12-13 min), 100%A (13-25 min)

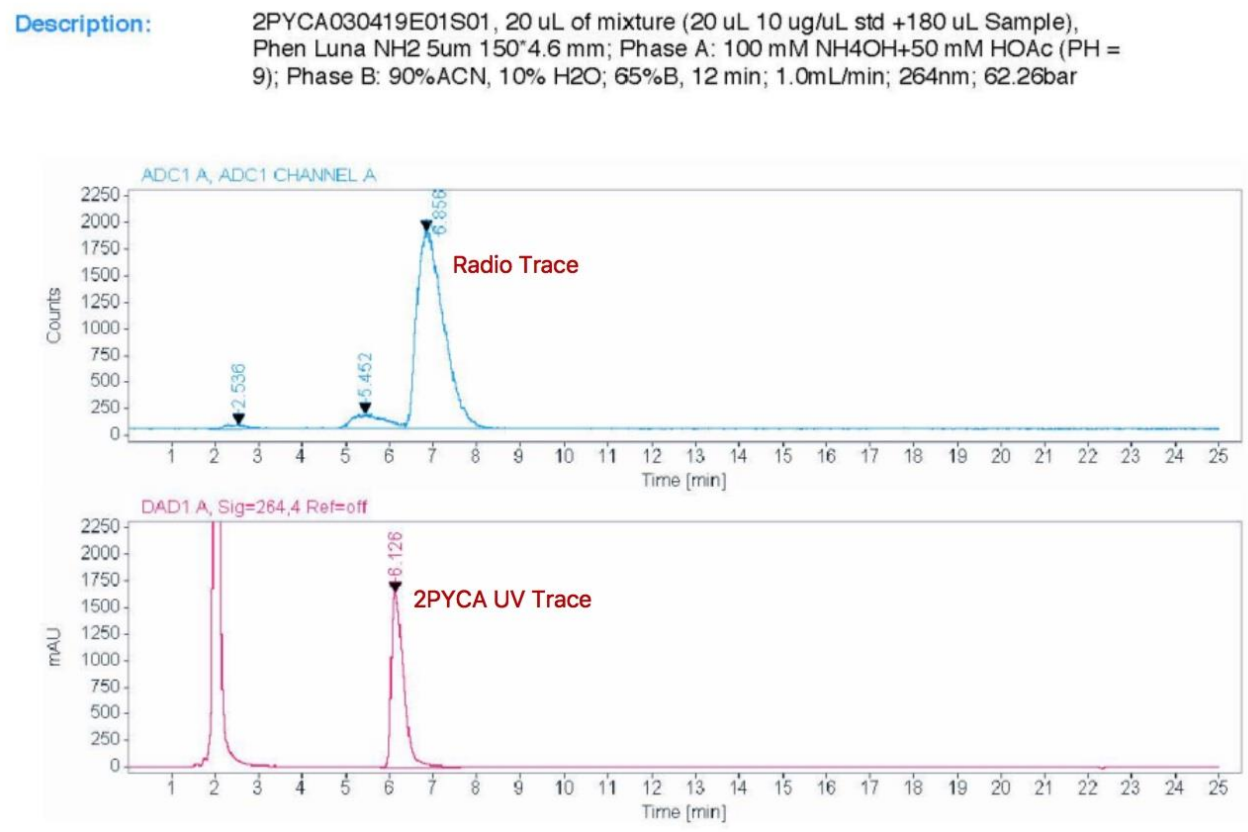

Benzo[*d*]thiazole-2-carboxylic-[11C]acid, ([11C]11l) synthesis

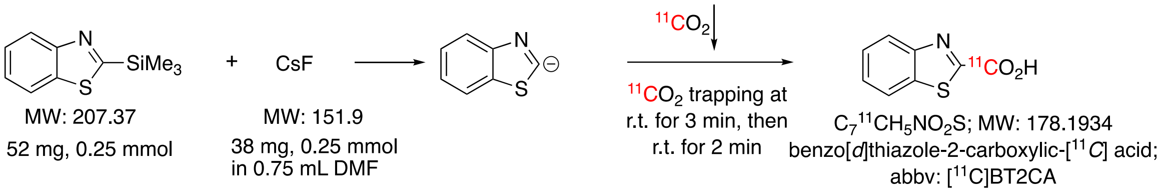

Following the procedures of pre-labeling work and ^{11}C-carboxylation reaction described above, [^{11}C]BT2CA, **[^{11}C]11l**, was synthesized (see above scheme for details) and the detailed results are listed in the table below:

Supplementary Table 27. Summarization of **[^{11}C]11l** synthesis experimental results

No.	RCP and RCY
Exp 01	97.1% & 75.9%
Exp 02	97.8% & 68.3%
Exp 03	97.7% & 80.5%
Avg ± SD of RCP and RCY	97.5 ± 0.4% & 74.9 ± 6.2%

Supplementary Figure 27. A [^{11}C]BT2CA, **[^{11}C]11l,** crude product sample analytical HPLC profile

HPLC analysis conditions: stationary phase: Phenomenex Luna(2) C18 150 × 4.6 mm; Mobile phase: phase A (0.1% formic acid), phase B (90% CH_3CN/10% H_2O); UV: 284 nm; Flow rate: 1.0 mL/min; Gradient/isocratic method: 30%B/70%A (0-10 min), rise 30%B to 100%B (10-11 min), 100%B (11-18 min), drop 100% to 30%B (18-19 min), 30%B (19-23 min).

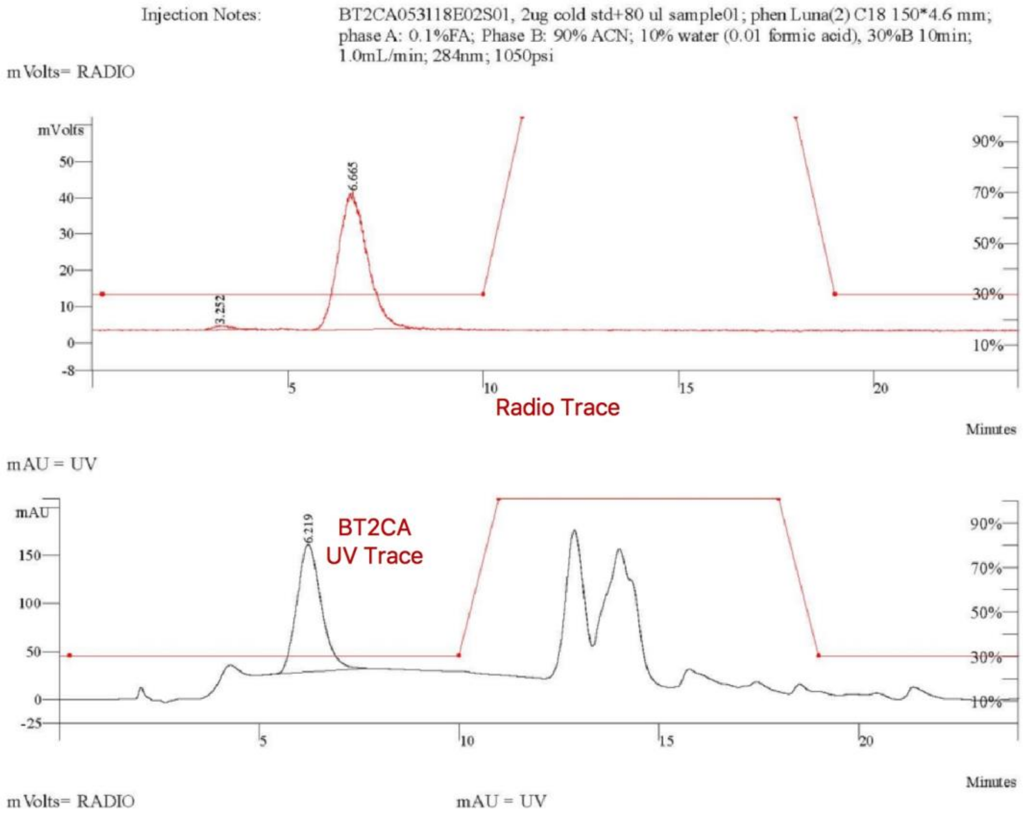

1-Methyl-1H-imidazole-2-carboxylic-[^{11}C]acid, ([^{11}C]11m) synthesis

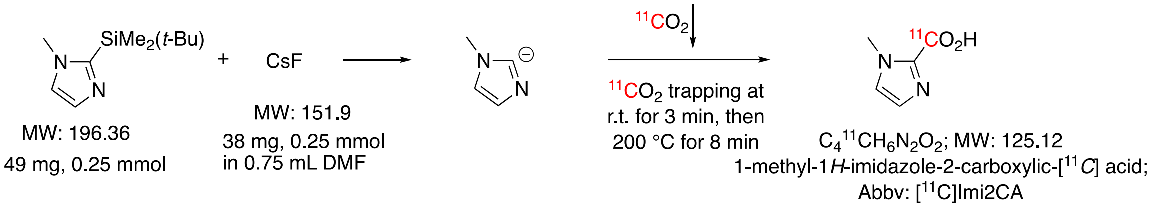

Following the procedures of pre-labeling work and ^{11}C-carboxylation reaction described above, [^{11}C]Imi2CA, **[^{11}C]11m**, was synthesized (see above scheme for details) and the detailed result is listed in the table below:

Supplementary Table 28. Summarization of **[^{11}C]11m** synthesis experimental results

No.	Reaction conditions	RCP and RCY
Exp 01	120 ^{o}C, 8 min	NA (no product peak detected)
Exp 02	160 ^{o}C, 8 min	78.0% & 1.3%
Exp 03	200 ^{o}C, 8 min	94.4% & 6.5%

Supplementary Figure 28. A [^{11}C]Imi2CA, **[^{11}C]11m,** crude product sample analytical HPLC profile

HPLC analysis conditions: stationary phase: Phenomenex Luna NH_2 150 × 4.6 mm; Mobile phase: phase A 200 mM NH_4OH + 100 mM HOAc (PH = 9.5), phase B (90% CH_3CN/10% H_2O); UV: 254 nm; Flow rate: 1.0 mL/min; Gradient/isocratic method: 60%B/70%A (0-12 min), rise 70%A to 100%A (12-13 min), 100%A (13-24 min).

Description: Imi2CA050319E01S01, 20 uL of mixture (5.0 uL,5.0 ug/uL std +295 uL Sample), Phen Luna NH2 5um 150*4.6 mm; Phase A: 200 mM NH4OH+100 mM HOAc (PH = 9.5); Phase B: 90%ACN, 10% H2O; 60%B, 12 min; 1.0mL/min; 254nm; 70.41bar

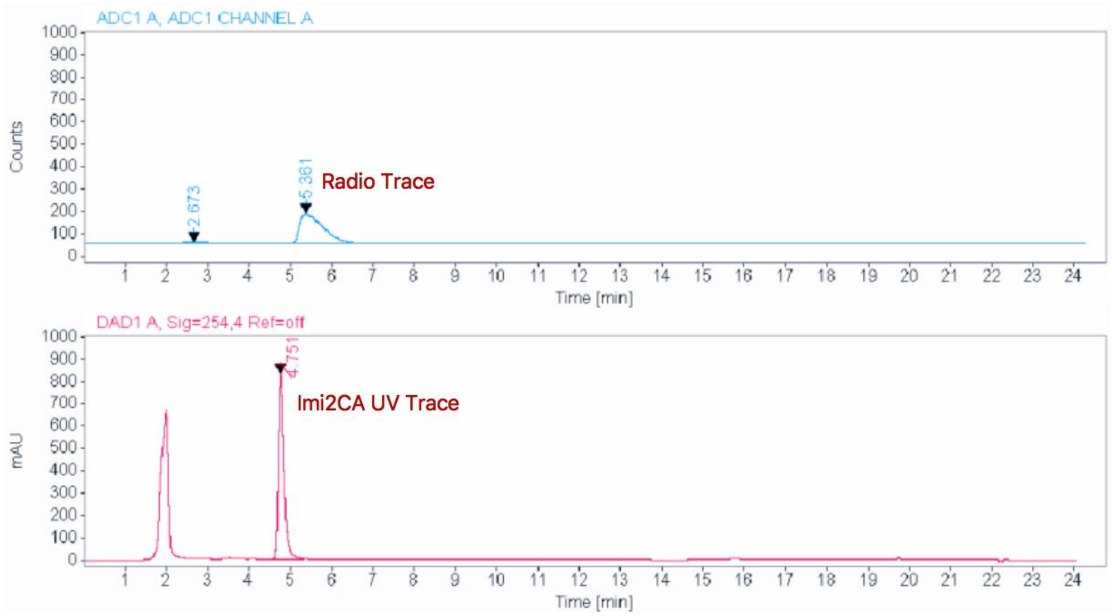

FMDS ^{11}C-carboxylation with sp^3-hybridized carbon attached trimethylsilanes

2-(4-Bromophenyl)acetic-1-[^{11}C]acid, ([^{11}C]13a) synthesis

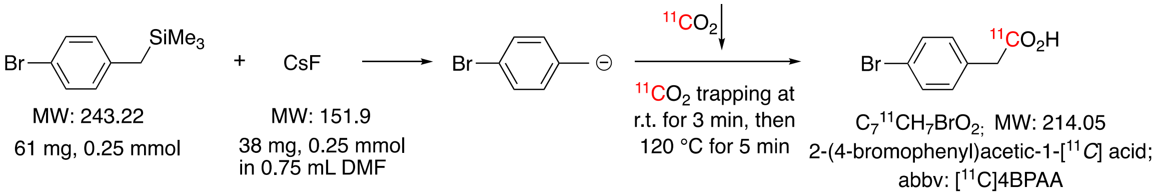

Following the procedures of pre-labeling work and ^{11}C-carboxylation reaction described above, [^{11}C]4BPAA, **[^{11}C]13a**, was synthesized (see above scheme for details) and the detailed results are listed in the table below:

Supplementary Table 29. Summarization of **[^{11}C]13a** synthesis experimental results

No.	RCP and RCY
Exp 01	88.4% & 68.5%
Exp 02	80.5% & 73.5%
Exp 03	78.4% & 75.4%
Avg ± SD of RCP and RCY	82.4 ± 5.3% & 72.5 ± 3.6%

Supplementary Figure 29. A [^{11}C]4BPAA, **[^{11}C]13a,** crude product sample analytical HPLC profile

HPLC analysis conditions: stationary phase: Phenomenex Luna(2) C18 150 × 4.6 mm; Mobile phase: phase A (0.1% formic acid), phase B (90% CH_3CN/10% H_2O); UV: 254 nm; Flow rate: 1.0 mL/min; Gradient/isocratic method: 50%B/50%A (0-10 min), rise 50%B to 100%B (10-11 min), 100%B (11-26 min), drop 100% to 50%B (26-27 min), 30%B (27-30 min).

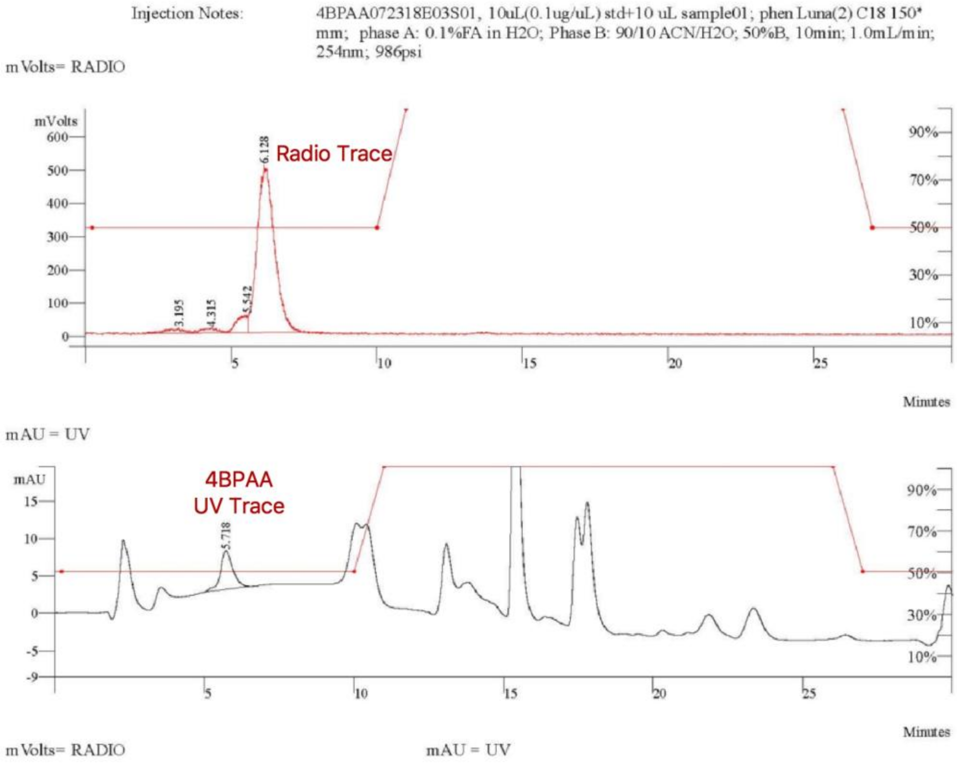

2-(2-Bromophenyl)acetic-1-[^{11}C]acid, ([^{11}C]13b) synthesis

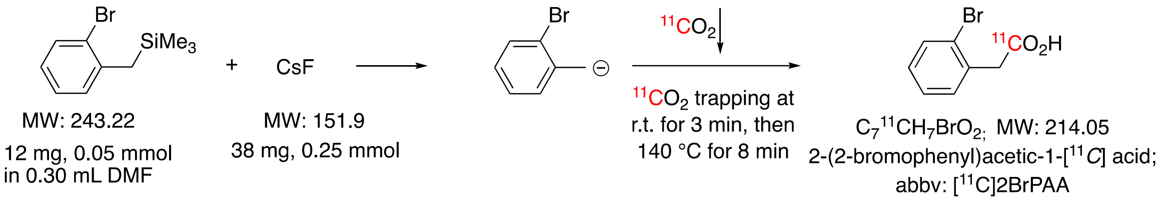

Following the procedures of pre-labeling work and ^{11}C-carboxylation reaction described above, [^{11}C]2BrPAA, **[^{11}C]13b**, was synthesized (see above scheme for details) and the detailed results are listed in the table below:

Supplementary Table 30. Summarization of **[^{11}C]13b** synthesis experimental results

No.	RCP and RCY
Exp 01	97.4% & 74.8%
Exp 02	>99% & 41.1%
Exp 03	94.1% & 48.3%
Avg ± SD of RCP and RCY	96.8 ± 2.5% & 54.7 ± 17.7%

Supplementary Figure 30. A [^{11}C]2BrPAA, **[^{11}C]13b,** crude product sample analytical HPLC profile

HPLC analysis conditions: stationary phase: Phenomenex Luna(2) C18 150 × 4.6 mm; Mobile phase: phase A (0.1% formic acid), phase B (90% CH_3CN/10% H_2O); UV: 254 nm; Flow rate: 1.0 mL/min; Gradient/isocratic method: 40%B/60%A (0-12 min), rise 40%B to 100%B (12-13 min), 100%B (11-23 min).

Description: 2BrPAA030819E04S01, 20 uL of mixture (100 uL 10.0 ug/uL std +100 uL Sample), Phen Luna(2) C18 150*4.6 mm; Phase A: 0.1% formic acid; Phase B: 90%ACN, 10% H2O; 40%B, 12 min; 1.0mL/min; 254nm; 92.32bar

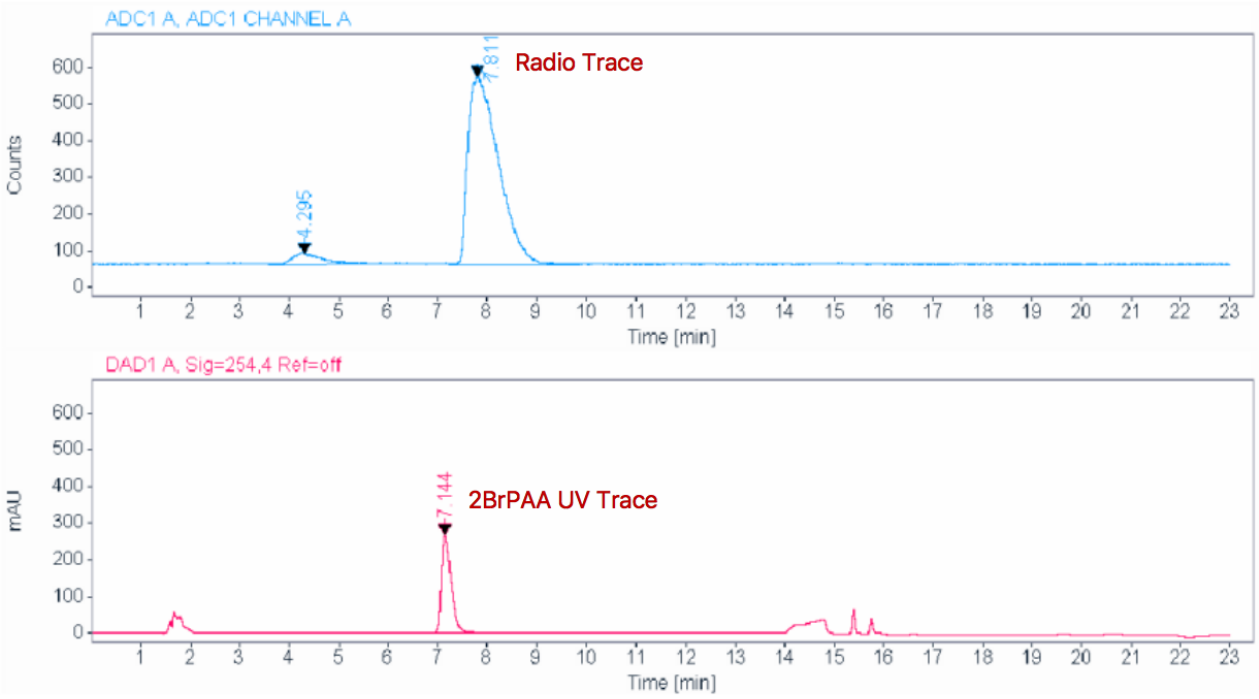

2-Phenylacetic-1-[^{11}C]acid, ([^{11}C]13c) synthesis

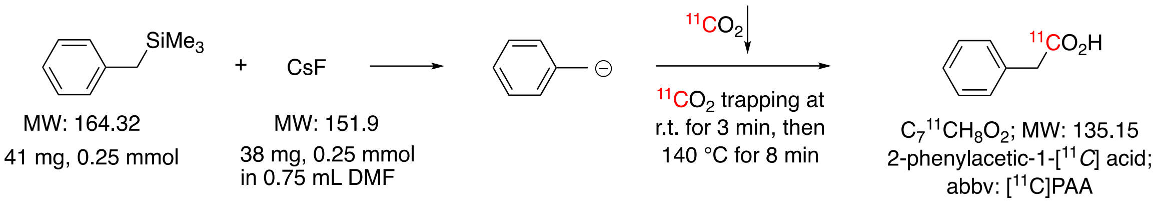

Following the procedures of pre-labeling work and ^{11}C-carboxylation reaction described above, [^{11}C]PAA, **[^{11}C]13c**, was synthesized (see above scheme for details) and the detailed results are listed in the table below:

Supplementary Table 31. Summarization of **[^{11}C]13c** synthesis experimental results

No.	RCP and RCY
Exp 01	96.2% & 68.9%
Exp 02	94.1% & 53.5%
Exp 03	94.7% & 65.2%
Avg ± SD of RCP and RCY	95.0 ± 1.1% & 62.5 ± 3.2%

Supplementary Figure 31. A [^{11}C]PAA, **[^{11}C]13c,** crude product sample analytical HPLC profile

HPLC analysis conditions: stationary phase: Phenomenex Luna(2) C18 150 × 4.6 mm; Mobile phase: phase A (0.1% formic acid), phase B (90% CH_3CN/10% H_2O); UV: 254 nm; Flow rate: 1.0 mL/min; Gradient/isocratic method: 35%B/65%A (0-12 min), rise 35%B to 100%B (12-13 min), 100%B (13-19 min), drop 100% to 35%B (19-20 min), 35%B (20-22 min).

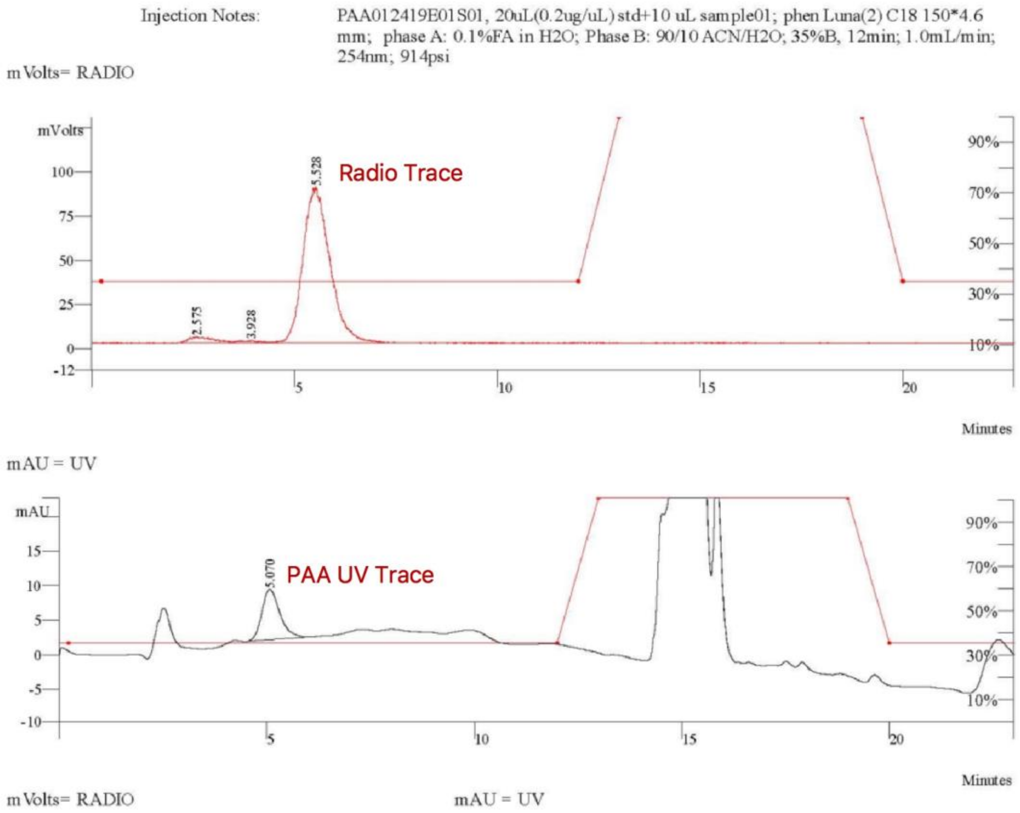

9*H*-fluorene-9-carboxylic-[11C]acid, ([11C]13d) synthesis

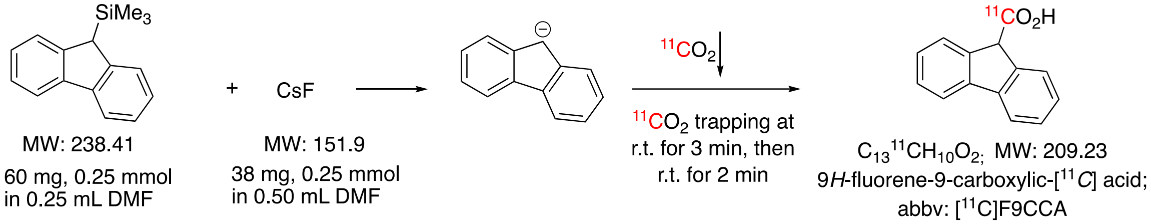

Following the procedures of pre-labeling work and ^{11}C-carboxylation reaction described above, [^{11}C]F9CCA, **[^{11}C]13d**, was synthesized (see above scheme for details) and the detailed results are listed in the table below:

Supplementary Table 32. Summarization of **[^{11}C]13d** synthesis experimental results

No.	RCP and RCY
Exp 01	99.6% & 93.8%
Exp 02	99.5% & 91.2%
Exp 03	>99% & 96.6%
Avg ± SD of RCP and RCY	99.4 ± 0.3% & 93.9 ± 2.7%

Supplementary Figure 32. A [^{11}C]F9CCA, **[^{11}C]13d,** crude product sample analytical HPLC (A-HPLC) profile HPLC analysis conditions: stationary phase: Phenomenex Luna(2) C18 150 × 4.6 mm; Mobile phase: phase A (0.1% formic acid), phase B (90% CH_3CN/10% H_2O); UV: 266 nm; Flow rate: 1.0 mL/min; Gradient/isocratic method: 55%B/45%A (0-12 min), rise 55%B to 100%B (12-13 min), 100%B (11-26 min), drop 100% to 55%B (26-27 min), 55%B (27-30 min).

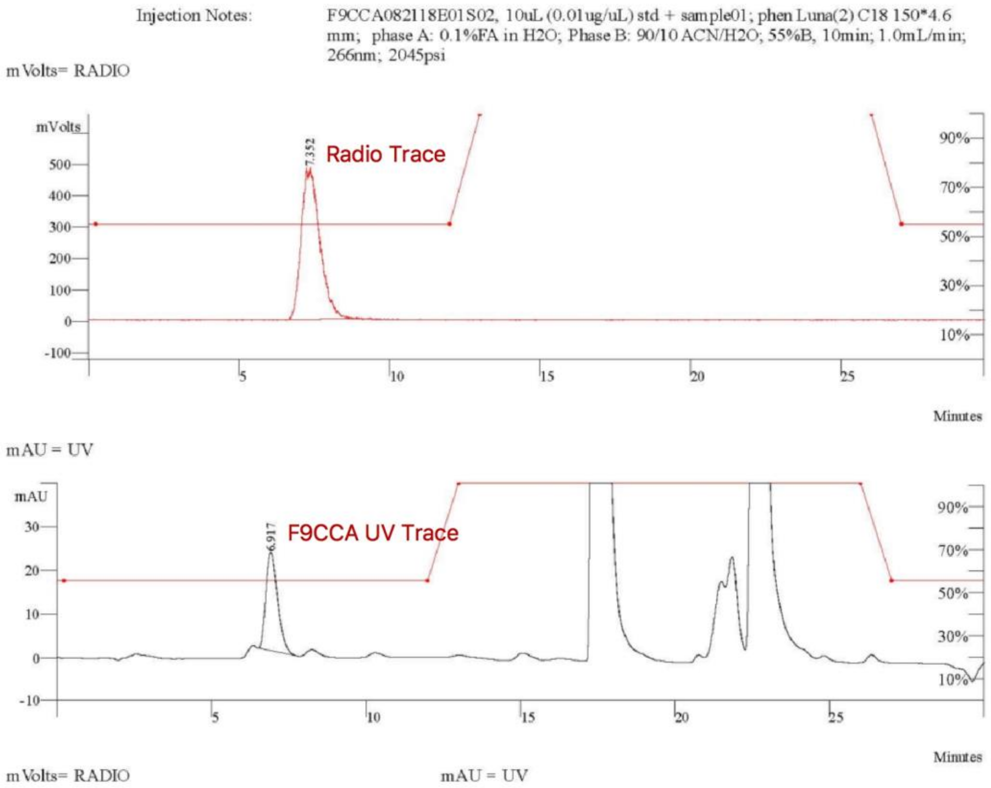

1*H*-Indene-1-Carboxylic-[^{11}C]acid, ([^{11}C]13e) synthesis

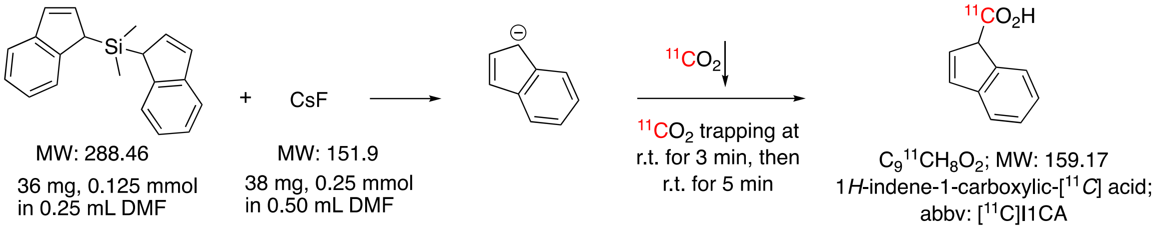

Following the procedures of pre-labeling work and ^{11}C-carboxylation reaction described above, [^{11}C]I1CA, **[^{11}C]13e**, was synthesized (see above scheme for details) and the detailed results are listed in the table below:

Supplementary Table 33. Summarization of **[^{11}C]13e** synthesis experimental results

No.	RCP and RCY
Exp 01	98.1% & 97.4%
Exp 02	98.7% & 97.7%
Exp 03	99.6% & 96.2%
Avg ± SD of RCP and RCY	98.8 ± 0.8% & 97.1 ± 0.8%

Supplementary Figure 33. A [^{11}C]I1CA, **[^{11}C]13e,** crude product sample analytical HPLC profile

HPLC analysis conditions: stationary phase: Phenomenex Luna(2) C18 150 × 4.6 mm; Mobile phase: phase A (0.1% formic acid), phase B (90% CH_3CN/10% H_2O); UV: 254 nm; Flow rate: 1.0 mL/min; Gradient/isocratic method: 40%B/60%A (0-12 min), rise 40%B to 100%B (12-13 min), 100%B (13-25 min).

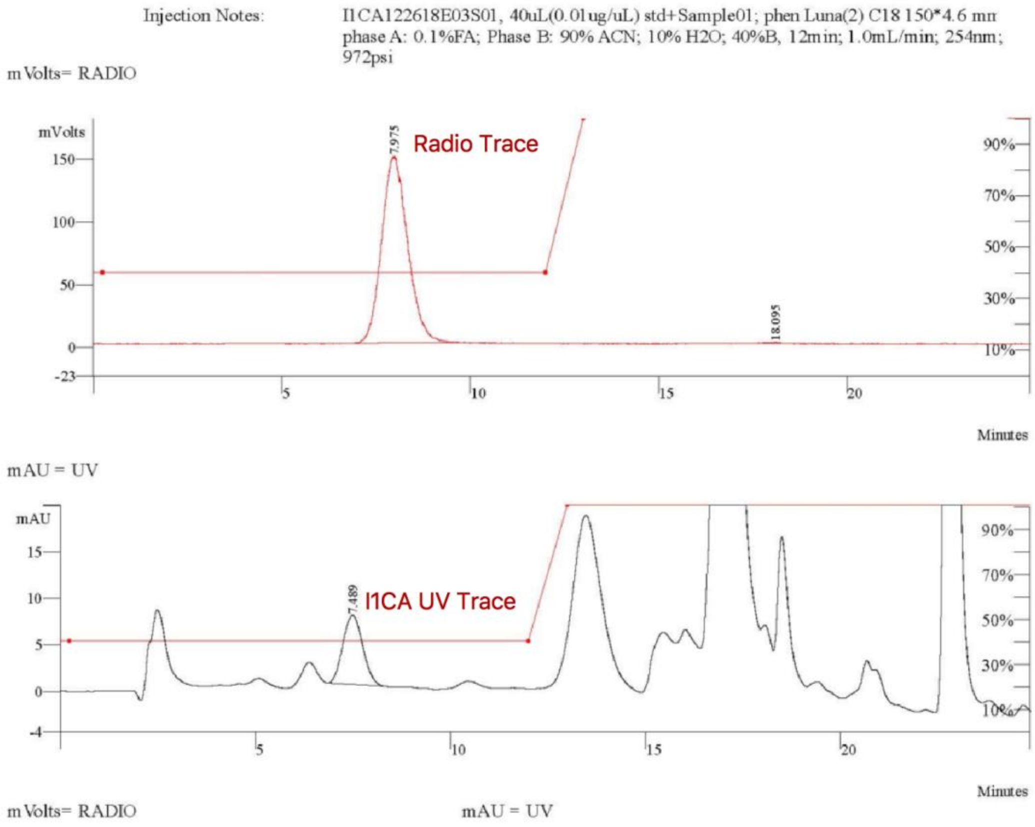

2,2,2-Trifluoroacetic-1-[^{11}C]acid, ([^{11}C]13f) synthesis

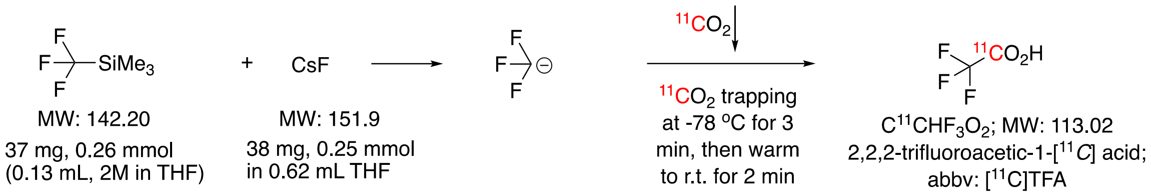

Following the procedures of pre-labeling work and ^{11}C-carboxylation reaction described above, [^{11}C]TFA, **[^{11}C]13f**, was synthesized (see above scheme for details) and the detailed results are listed in the table below:

Supplementary Table 34. Summarization of **[^{11}C]13f** synthesis experimental results

No.	RCP and RCY
Exp 01	>99% & 93.3%
Exp 02	>99% & 93.9%
Exp 03	>99% & 89.8%
Avg ± SD of RCP and RCY	>99 ± 0% & 92.3 ± 2.2%

Supplementary Figure 34. A [^{11}C]TFA, **[^{11}C]13f,** crude product sample analytical HPLC profile

HPLC analysis conditions: stationary phase: Phenomenex PFP(2) C18 250 × 4.6 mm; Mobile phase: phase A 0.1% Et_3N + H_2SO_4 (PH = 2.5), phase B (100% MeOH); UV: 200 nm; Flow rate: 1.0 mL/min; Gradient/isocratic method: 5%B/95%A (0-12 min), rise 5%B to 100%B (12-13 min), 100%B (13-21 min).

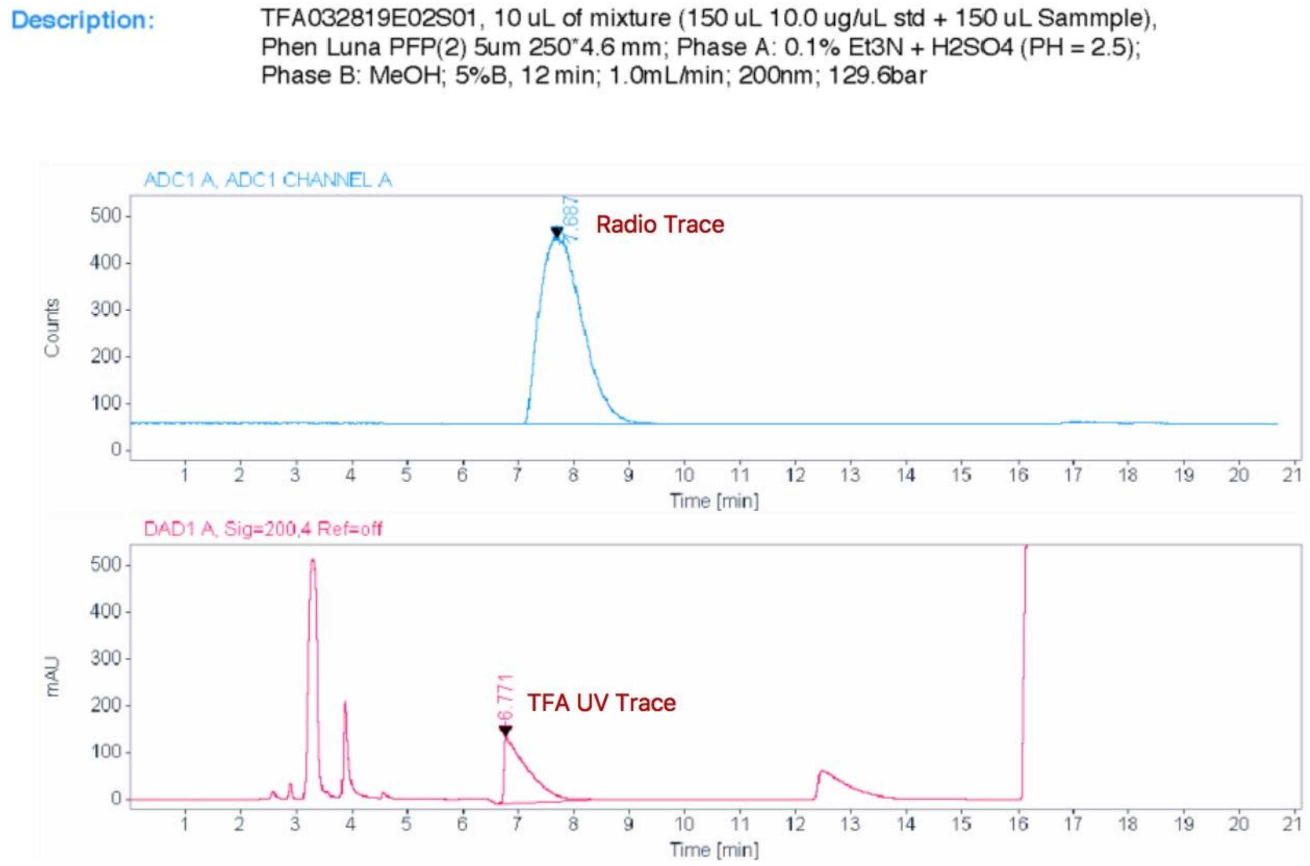

2,2-Dichloroacetic-1-[^{11}C]acid, ([^{11}C]13g) synthesis

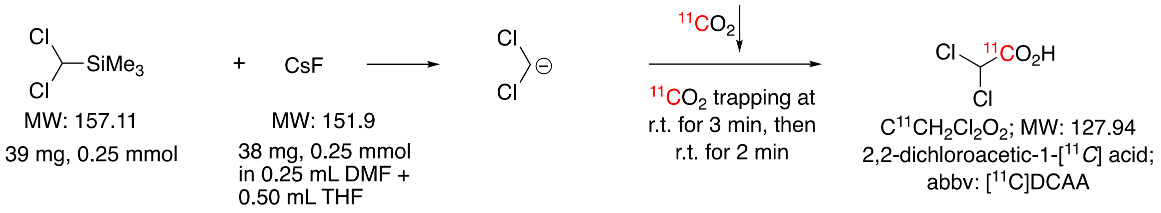

Following the procedures of pre-labeling work and ^{11}C-carboxylation reaction described above, [^{11}C]DCAA, **[^{11}C]13g**, was synthesized (see above scheme for details) and the detailed results are listed in the table below:

Supplementary Table 35. Summarization of **[^{11}C]13g** synthesis experimental results

No.	RCP and RCY
Exp 01	59.7% & 49.3%
Exp 02	45.1% & 28.0%
Exp 03	58.5% & 40.3%
Avg ± SD of RCP and RCY	54.4 ± 8.1% & 39.2 ± 10.7%

Supplementary Figure 35. A [^{11}C]DCAA, **[^{11}C]13g,** crude product sample analytical HPLC profile

HPLC analysis conditions: stationary phase: Phenomenex Luna(2) C18 150 × 4.6 mm; Mobile phase: phase A (0.1% formic acid), phase B (90% CH_3CN/10% H_2O); UV: 210 nm; Flow rate: 1.0 mL/min; Gradient/isocratic method: 1%B/99%A (0-12 min), rise 1%B to 100%B (12-13 min), 100%B (11-26 min), drop 100%B to 1%B (26-27 min), 1%B (27-30 min).

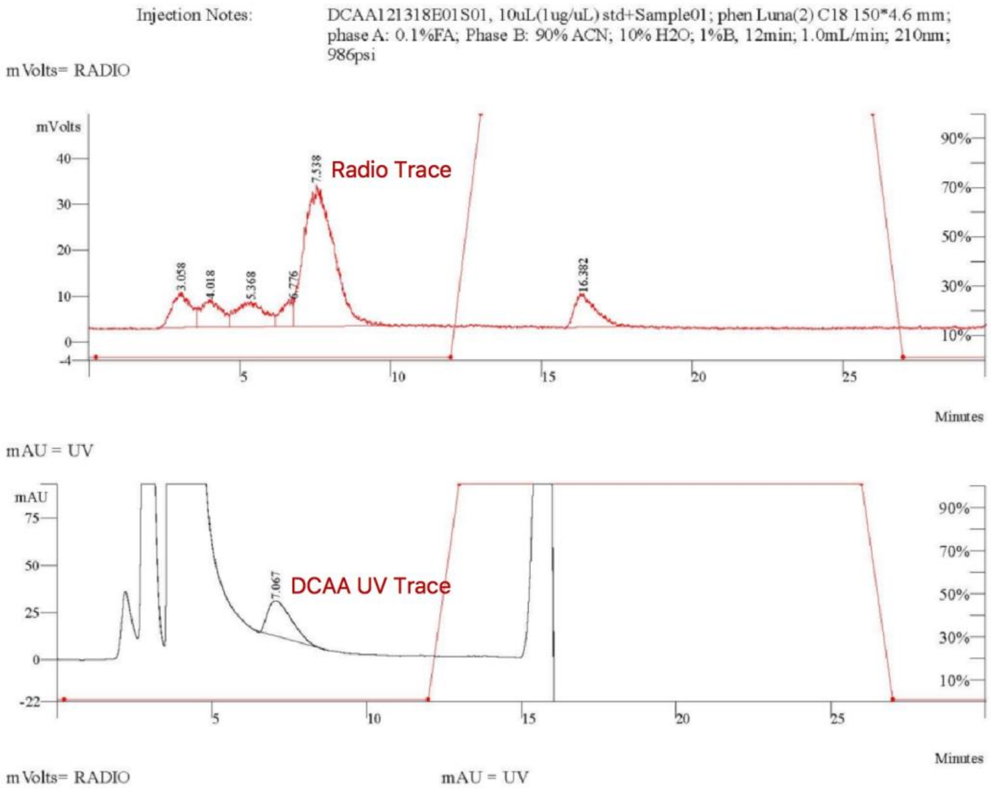

3-Ethoxy-3-oxopropanoic-1-[^{11}C]acid, ([^{11}C]13h) synthesis

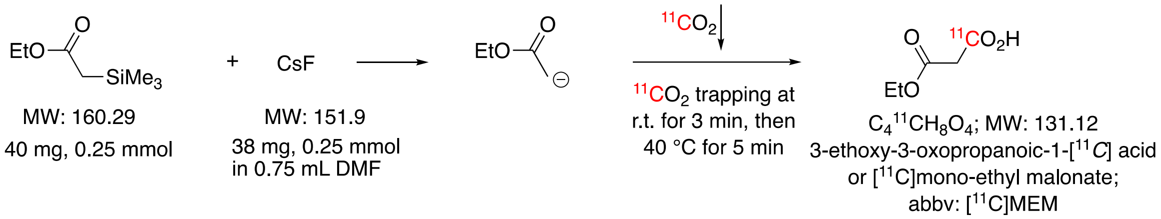

Following the procedures of pre-labeling work and ^{11}C-carboxylation reaction described above, [^{11}C]MEM, **[^{11}C]13h**, was synthesized (see above scheme for details) and the detailed results are listed in the table below:

Supplementary Table 36. Summarization of **[^{11}C]13h** synthesis experimental results

No.	RCP and RCY
Exp 01	>99% & 95.2%
Exp 02	96.5% & 96.3%
Exp 03	95.8% & 95.0%
Avg ± SD of RCP and RCY	97.1 ± 1.7% & 95.5 ± 0.7%

Supplementary Figure 36. A [^{11}C]MEM, **[^{11}C]13h,** crude product sample analytical HPLC profile

HPLC analysis conditions: stationary phase: Phenomenex Luna(2) C18 150 × 4.6 mm; Mobile phase: phase A (0.1% formic acid), phase B (90% CH_3CN/10% H_2O); UV: 210 nm; Flow rate: 1.0 mL/min; Gradient/isocratic method: 10%B/90%A (0-12 min), rise 10%B to 100%B (12-13 min), 100%B (13-26 min), drop 100%B to 10%B (26-27 min), 10%B (27-30 min).

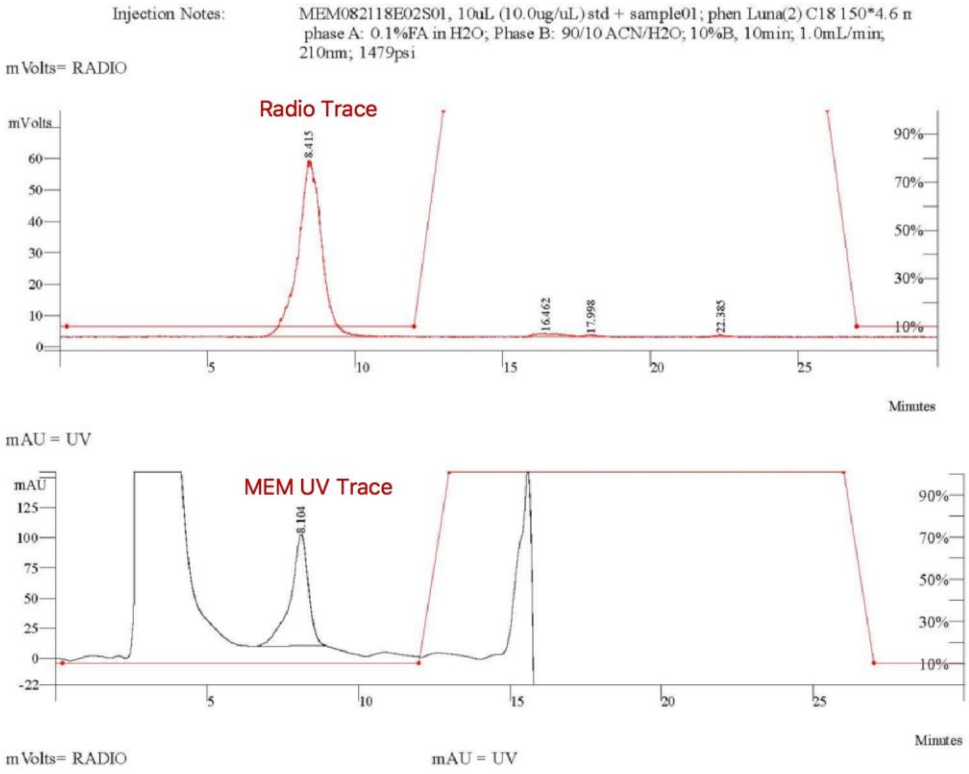

2-(Phenylsulfonyl)acetic-1-[^{11}C]acid, ([^{11}C]13i) synthesis

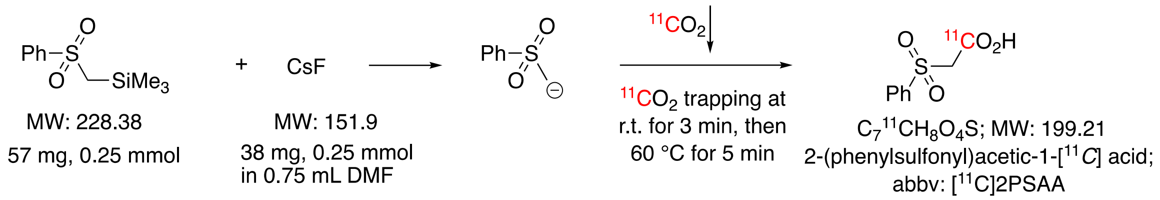

Following the procedures of pre-labeling work and ^{11}C-carboxylation reaction described above, [^{11}C]2PSAA, **[^{11}C]13i**, was synthesized (see above scheme for details) and the detailed results are listed in the table below:

Supplementary Table 37. Summarization of **[^{11}C]13i** synthesis experimental results

No.	RCP and RCY
Exp 01	98.4% & 61.9%
Exp 02	>99% & 99.3%
Exp 03	>99% & 98.7%
Avg ± SD of RCP and RCY	98.8 ± 0.3% & 86.6 ± 21.4%

Supplementary Figure 37. A [^{11}C]2PSAA, **[^{11}C]13i,** crude product sample analytical HPLC profile

HPLC analysis conditions: stationary phase: Phenomenex Luna(2) C18 150 × 4.6 mm; Mobile phase: phase A (0.1% formic acid), phase B (90% CH_3CN/10% H_2O); UV: 254 nm; Flow rate: 1.0 mL/min; Gradient/isocratic method: 20%B/80%A (0-12 min), rise 20%B to 100%B (12-13 min), 100%B (13-23 min).

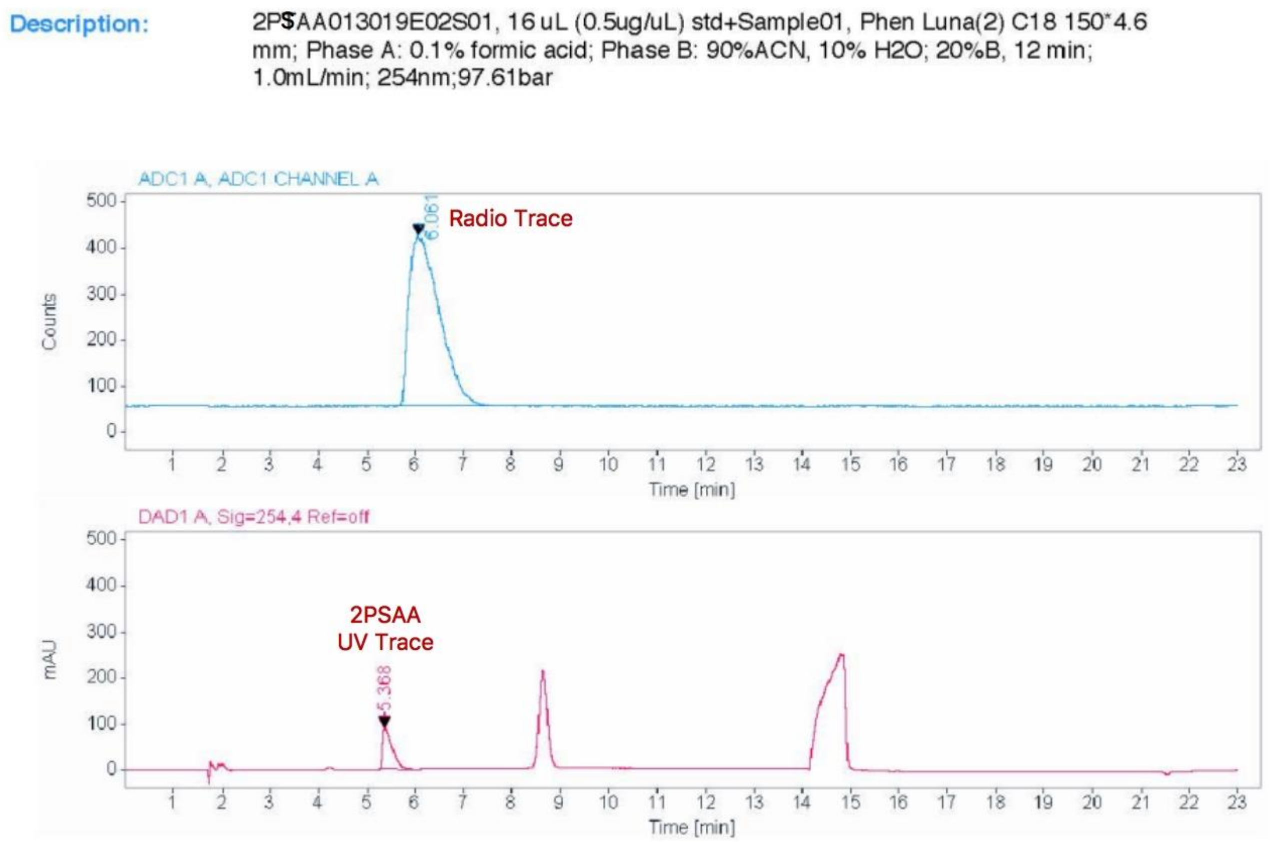

2-(Phenylthio)acetic-1-[11C]acid, ([11C]13j) synthesis

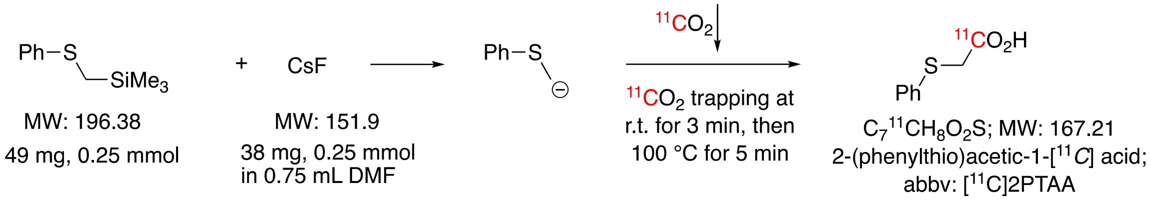

Following the procedures of pre-labeling work and ^{11}C-carboxylation reaction described above, [^{11}C]2PTAA, **[^{11}C]13j**, was synthesized (see above scheme for details) and the detailed results are listed in the table below:

Supplementary Table 38. Summarization of **[^{11}C]13j** synthesis experimental results

No.	RCP and RCY
Exp 01	83.3% & 16.1%
Exp 02	73.5% & 21.9%
Exp 03	94.7% & 21.1%
Exp 04	93.3% & 16.2%
Avg ± SD of RCP and RCY	86.2 ± 8.5% & 18.8 ± 2.7%

Supplementary Figure 38. A [^{11}C]2PTAA, **[^{11}C]13j,** crude product sample analytical HPLC profile

HPLC analysis conditions: stationary phase: Phenomenex Luna(2) C18 150 × 4.6 mm; Mobile phase: phase A (0.1% formic acid), phase B (90% CH_3CN/10% H_2O); UV: 254 nm; Flow rate: 1.0 mL/min; Gradient/isocratic method: 40%B/60%A (0-12 min), rise 40%B to 100%B (12-13 min), 100%B (13-26 min).

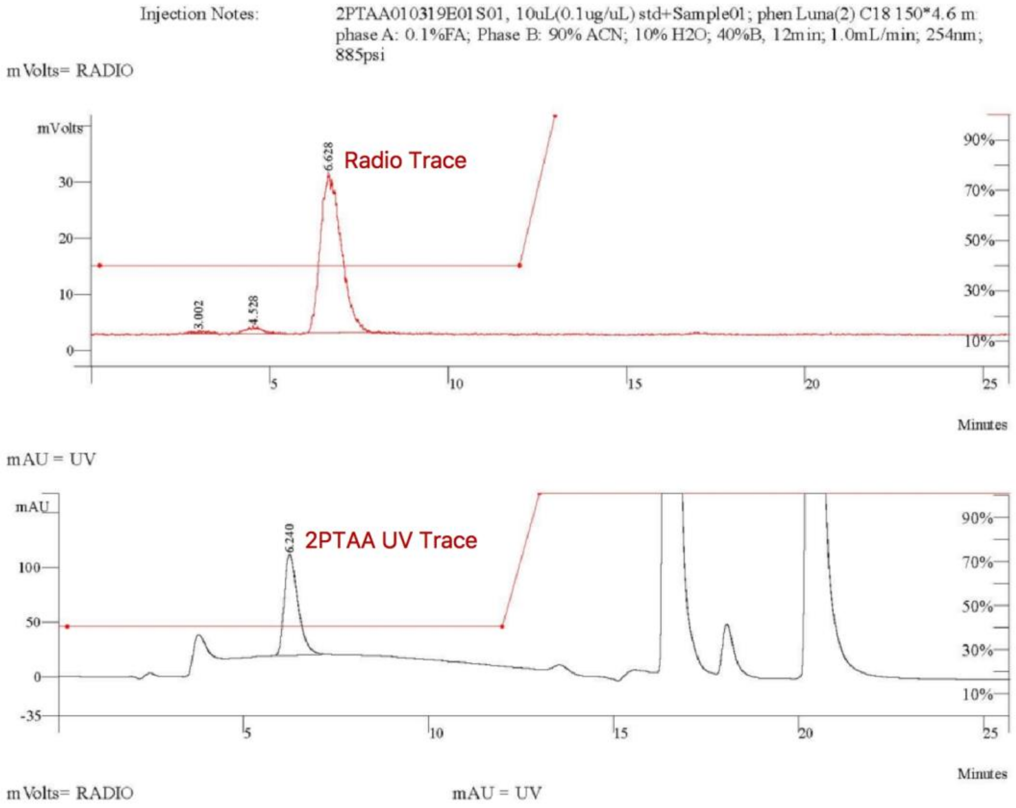

3-(Ethoxycarbonyl)but-3-enoic-1-[^{11}C]acid, ([^{11}C]13k) synthesis

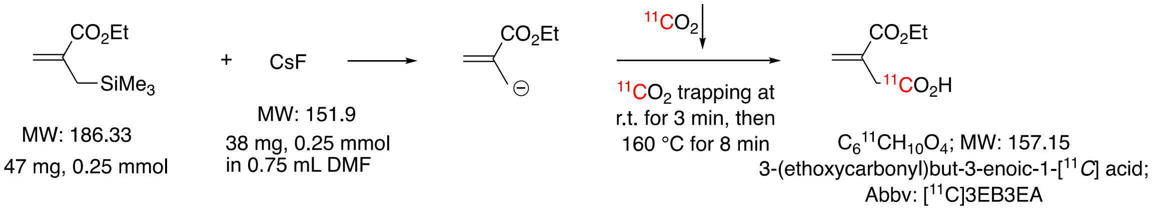

Following the procedures of pre-labeling work and ^{11}C-carboxylation reaction described above, [^{11}C]3EB3EA, **[^{11}C]13k**, was synthesized (see above scheme for details)and the detailed results are listed in the table below:

Supplementary Table 39. Summarization of **[^{11}C]13k** synthesis experimental results

No.	RCP and RCY
Exp 01	56.3% & 17.2%
Exp 02	57.9% & 20.2%
Exp 03	49.4% & 20.4%
Avg ± SD of RCP and RCY	54.5 ± 4.5% & 19.3 ± 1.8%

Supplementary Figure 39. A [^{11}C]3EB3EA, **[^{11}C]13k,** crude product sample analytical HPLC profile

HPLC analysis conditions: stationary phase: Phenomenex Luna(2) C18 150 × 4.6 mm; Mobile phase: phase A (0.1% formic acid), phase B (90% CH_3CN/10% H_2O); UV: 215 nm; Flow rate: 1.0 mL/min; Gradient/isocratic method: 20%B/80%A (0-15 min), rise 20%B to 100%B (15-16 min), 100%B (16-22 min).

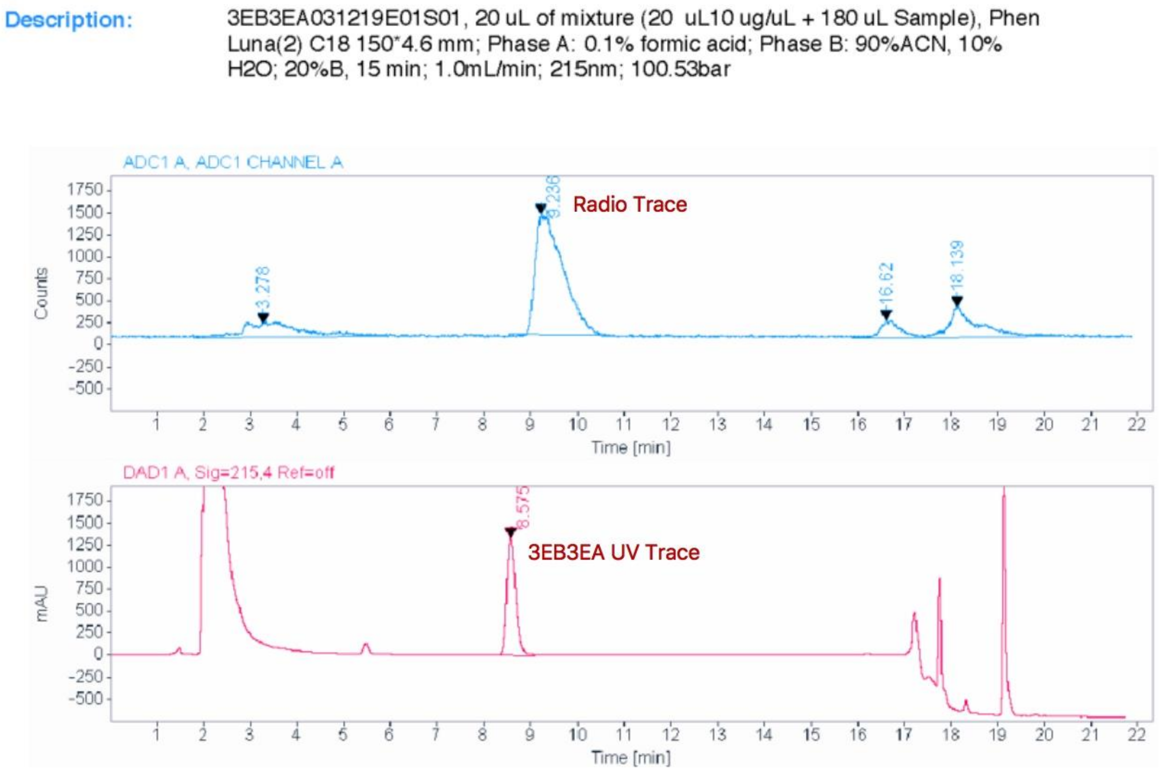

^{11}C-Methylation experimental results

[^{11}C]CH_3I/[^{11}C]CH_3OTf production

[^{11}C]CO_2 was generated by bombarding N_2 gas (360 psi 99.9999% pure N_2 doped with 0.5% O_2) via the ^{14}N(p,α)^{11}C nuclear reaction using a EBCO TR-19/9 cyclotron. General bombardment conditions for [^{11}C]CH_3I/[^{11}C]CH_3OTf production: 5 – 40 min beam time with 25 µA current. After the bombardment, target gas containing radioactivity was released and delivered to a GE TRACERlab FXC automatic synthesizer to convert [^{11}C]CO_2 to [^{11}C]CH_3I or [^{11}C]CH_3OTf. It took 16 – 18 min from end of bombardment (EOB) to finish the collection of [^{11}C]CH_3I or [^{11}C]CH_3OTf radioactivity in the reaction vial.

General procedures of ^{11}C-methylation reaction, determination of radiochemical purities (RCP) and radiochemical yields (RCY)

The FDSM ^{11}C-methylation experimental process was the same as FDSM ^{11}C-carboxylation reactions except for that there was no unreacted [^{11}C]CO_2 exclusion process since unreacted [^{11}C]CH_3I or by-product [^{11}C]CH_3OH dissolved well in the reaction mixture and there was no leakage of radioactivity detected during the sampling process after the ^{11}C-methylation reaction.

When [^{11}C]CH_3I or [^{11}C]CH_3OTf was ready, to a reaction vial equipped with outlet line, the radioactivity delivery line with a 4” needle was inserted into the anhydrous fluoride reagent and solvent reaction mixture. The organosilane precursor was immediately added. The reaction vial was placed in a dose calibrator for measuring the collected radioactivity. Once the increase of radioactivity in the reaction vial plateaued, the [^{11}C]CH_3I or [^{11}C]CH_3OTf delivery line and airflow outlet line were removed. The total activity trapped in the reaction vial was checked again as starting radioactivity $\mathbf{A_0}$. After keeping the reaction mixture stirring under desired temperature for a certain time (generally 2 – 10 minutes), the reaction mixture was measured again to have total radioactivity A_0' (*The value of* $\boldsymbol{A_0'}$ *was supposed to equal as* $\boldsymbol{A_0}$ *after decay correction since unreacted [*^{11}*C]*CH_3*I or by-product [*^{11}*C]*CH_3*OH dissolved well in the reaction mixture. However, it was found that the value of* $\boldsymbol{A_0'}$ *was slightly less than the value of* $\boldsymbol{A_0}$ *after decay correction in some experiments. It is most likely because of the small leakage of [*^{11}*C]*CH_3*I or by-product [*^{11}*C]*CH_3*OH from reaction vial*.) A small portion of reaction mixture (~ 0.1 mL) was removed and diluted with an acidic solution (CH_3CN/1% formic acid, 90/10) in a septa cap sealed glass vial; the radioactivity of this sample was counted and recorded. Next, an analytical sample, which was a mixture of an aliquot of sample solution (usually 10 µL) and a product standard solution (usually 10 µL, 1mg/mL solution), was analyzed by analytical HPLC (A-HPLC). The percentage of radio-peak in radio-chromatogram coincident with product reference UV peak was regarded as radiochemical purity (RCP) and also as radiochemical yield (RCY, decay corrected) if $\boldsymbol{A_0' = A_0}$, or RCY = RCP × [$\boldsymbol{A_0'/A_0}$] at the case of $\boldsymbol{A_0' < A_0}$. If the reaction mixture was submitted for the purification process (solid phase extraction, semi-prep HPLC, anion/cation exchange method, or the combination of two of these methods), the total amount of radioactivity of purified product was recorded as $\mathbf{A_{prod}}$. The radiochemical yield (RCY, decay corrected) was calculated as $\frac{Aprod}{A0} \times 100\%$. **Molar activity values ($\mathbf{A_m}$)**,

decay corrected back to EOB and recorded in GBq/μmol, were determined from the carbon-11 activity in the HPLC product peak and the mass of compound. Total synthesis times were calculated from EOB to the end of radioactive product collection after the purification.

2-[4-^{11}C]Butanone ([^{11}C]MeK, [^{11}C]14) synthesis

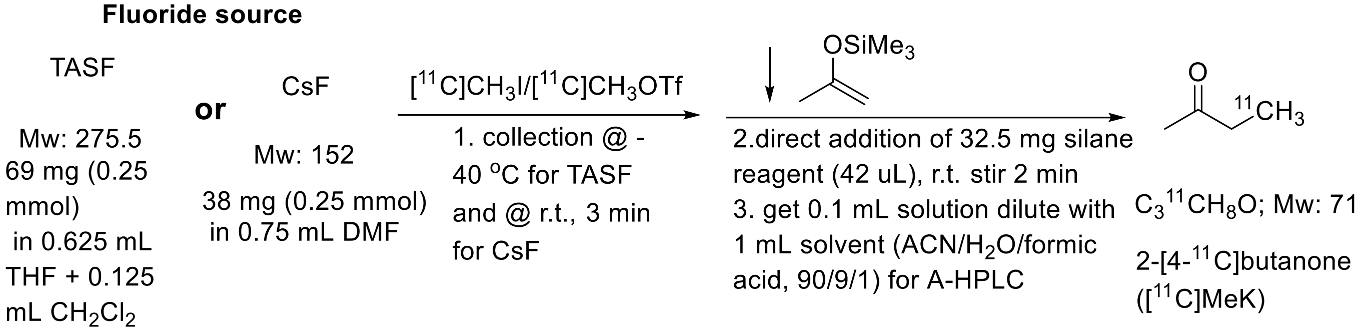

Following the procedures of pre-labeling work and ^{11}C-methylation reaction described above, 2-[4-^{11}C]butanone ([^{11}C]MeK, **[^{11}C]14**) was synthesized (see above scheme for details) and the detailed results are listed in the table below:

Supplementary Table 40. Summarization of **[^{11}C]14** synthesis experimental results

No.	Fluoride Source	Exp 01 Results	Exp 02 Results	Exp 03 Results	Statistic Results
01	TASF	RCY: 40.7%	RCY: 12.7% *$^{11}CH_3OTf$ was used*		
02	CsF	RCY: 59.0%	RCY: 45.7%	RCY: 26.4%	RCY: 43.7 ± 16.4%
03	CsF	RCY: 26.6% *$^{11}CH_3OTf$ was used*			

Supplementary Figure 40. A [^{11}C]MeK, **[^{11}C]14,** crude product sample analytical HPLC profile

HPLC analysis conditions: stationary phase: Phenomenex Luna C18 150 × 4.6 mm; Mobile phase: phase A (0.1% formic acid), phase B (100% CH_3CN); UV: 254 nm; Flow rate: 1.0 mL/min; Gradient/isocratic method: 15%B/85%A (0-9 min), rise 15%B to 100%B (9-10 min), 100%B (10-22 min), drop 100% to 15%B (22-24 min), 15%B (24-27 min).

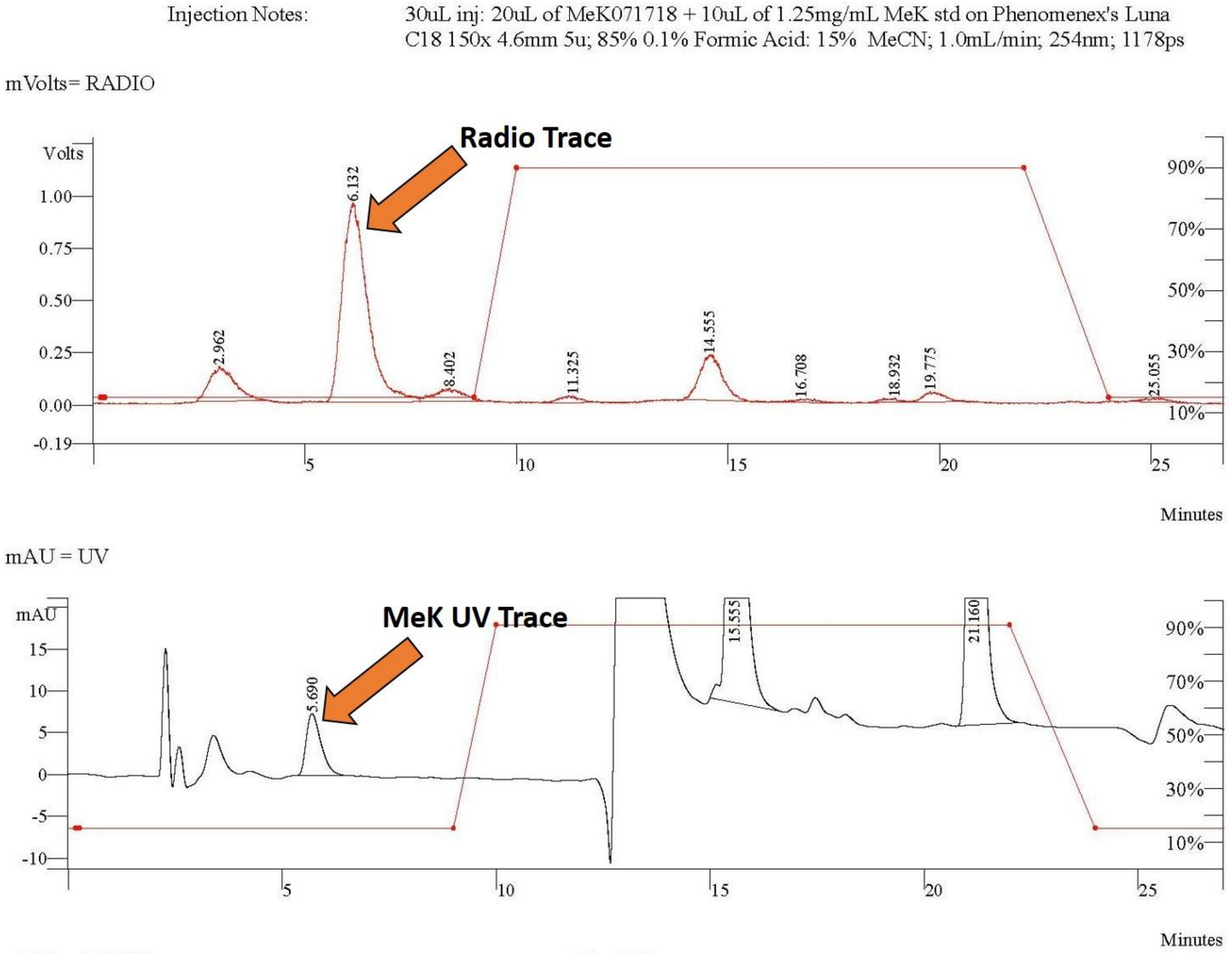

FMDS ^{11}C-methylation with TMS/TBDMS/TIPS/TES attached precursors

[^{11}C]Anisole, ([^{11}C]16a) synthesis

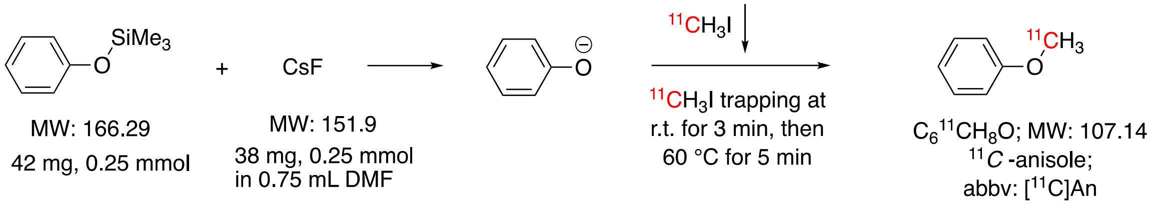

Following the procedures of pre-labeling work and ^{11}C-methylation reaction described above, [^{11}C]Anisole, **[^{11}C]16a**, was synthesized (see above scheme for details) and the detailed results are listed in the table below:

Supplementary Table 41. Summarization of **[^{11}C]16a** synthesis experimental results

No.	RCP and RCY
Exp 01	97.6% & 91.9%
Exp 02	85.2% & 81.8%
Exp 03	81.2% & 80.5%
Avg ± SD of RCP and RCY	88.0 ± 8.6% & 84.7 ± 6.2%

Supplementary Figure 41. An [^{11}C]An, **[^{11}C]16a,** crude product sample analytical HPLC profile

HPLC analysis conditions: stationary phase: Phenomenex Luna(2) C18 150 × 4.6 mm; Mobile phase: phase A (0.1% formic acid), phase B (90% CH_3CN/10% H_2O); UV: 254 nm; Flow rate: 1.0 mL/min; Gradient/isocratic method: 55%B/45%A (0-10 min), rise 55%B to 100%B (10-11 min), 100%B (11-26 min), drop 100%B to 55%B (26-27 min), 55%B (27-30 min).

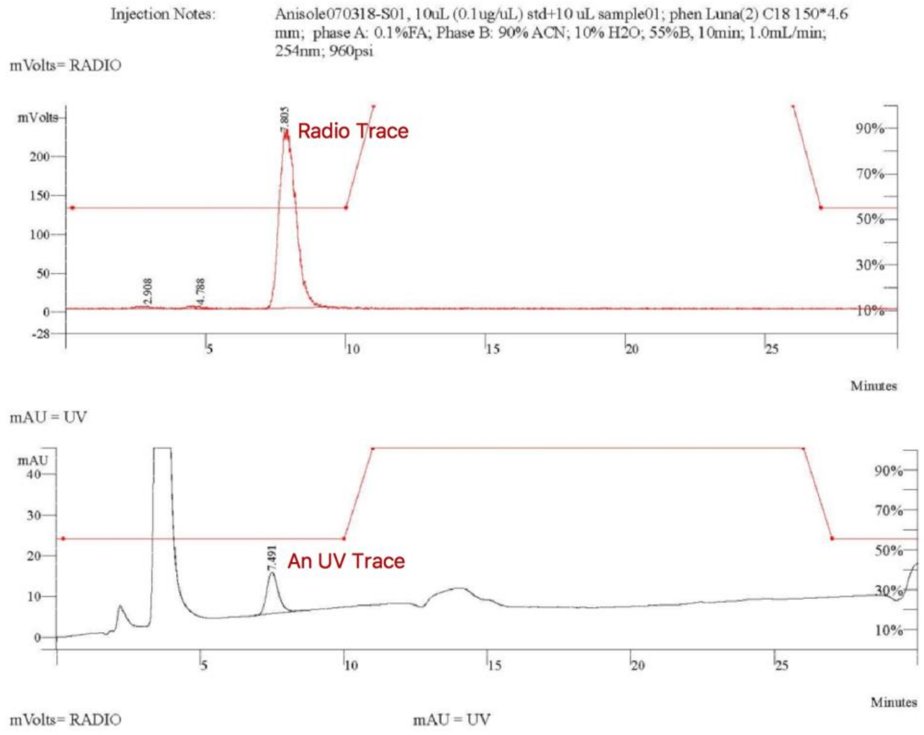

1-Bromo-4-(methoxy-^{11}C)benzene, ([^{11}C]16b) synthesis

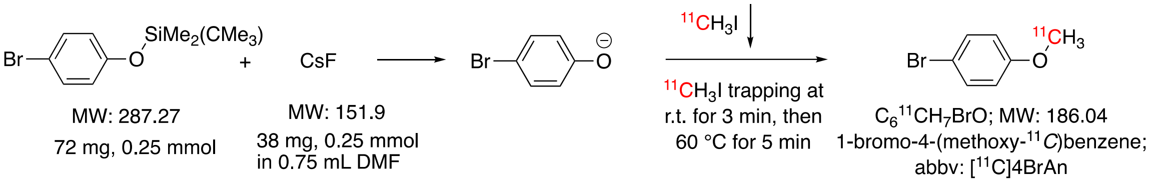

Following the procedures of pre-labeling work and ^{11}C-methylation reaction described above, [^{11}C]4BrAn, **[^{11}C]16b**, was synthesized (see above scheme for details) and the detailed results are listed in the table below:

Supplementary Table 42. Summarization of **[^{11}C]16b** synthesis experimental results

No.	RCP and RCY
Exp 01	82.8% & 82.0%
Exp 02	86.0% & 80.7%
Exp 03	83.9% & 83.1%
Avg ± SD of RCP and RCY	84.2 ± 1.6% & 81.9 ± 1.2%

Supplementary Figure 42. A [^{11}C]4BrAn, **[^{11}C]16b,** crude product sample analytical HPLC profile

HPLC analysis conditions: stationary phase: Phenomenex Luna(2) C18 150 × 4.6 mm; Mobile phase: phase A (0.1% formic acid), phase B (90% CH_3CN/10% H_2O); UV: 254 nm; Flow rate: 1.0 mL/min; Gradient/isocratic method: 70%B/30%A (0-12 min), rise 70%B to 100%B (12-13 min), 100%B (13-21 min), drop 100%B to 70%B (21-22 min), 70%B (22-25 min).

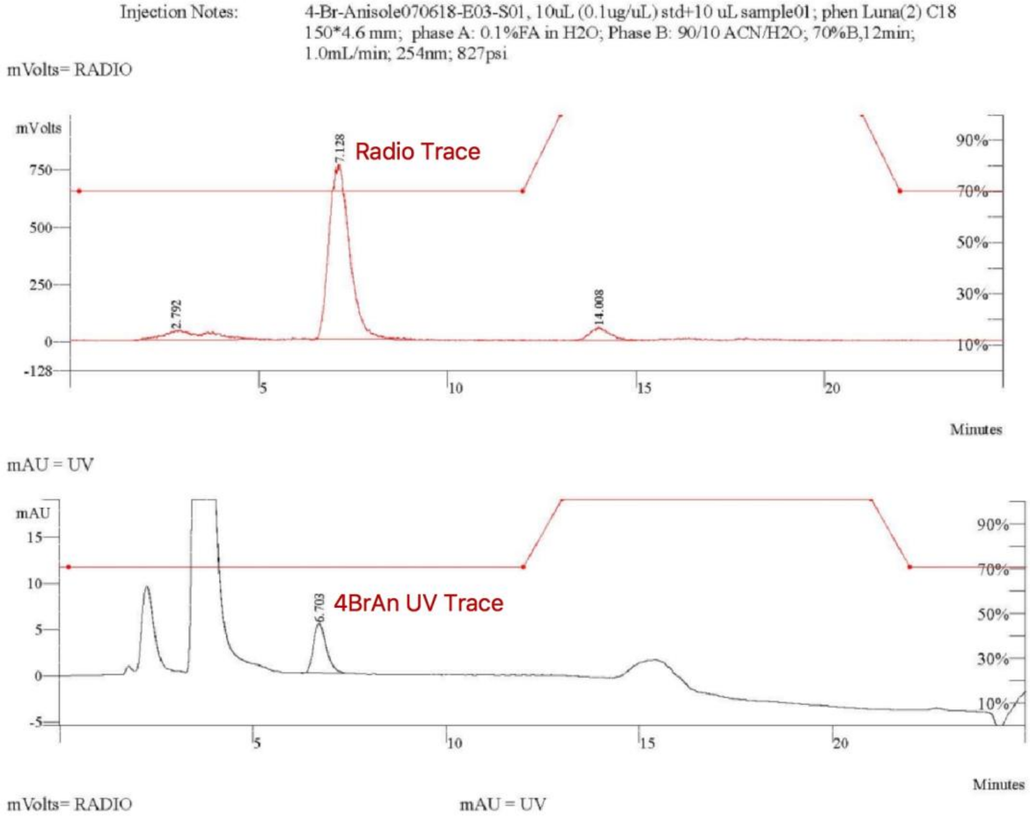

1-Bromo-4-(methoxy-^{11}C)benzene, ([^{11}C]16b) synthesis using triisopropylsilyl precursor

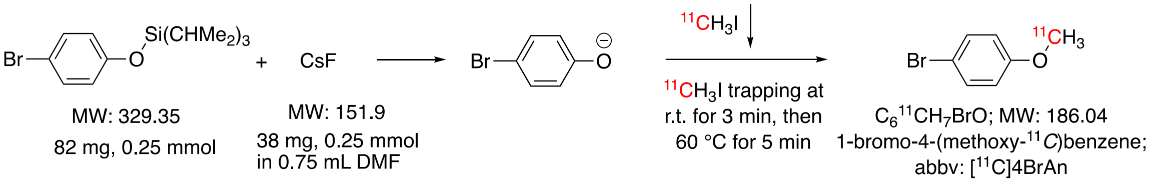

Following the procedures of pre-labeling work and ^{11}C-methylation reaction described above, [^{11}C]4BrAn, **[^{11}C]16b**, was synthesized (see above scheme for details) and the detailed results are listed in the table below:

Supplementary Table 43. Summarization of **[^{11}C]16b** synthesis experimental results

No.	RCP and RCY
Exp 01	93.2% & 92.4%
Exp 02	77.7% & 76.3%
Exp 03	93.2% & 88.3%
Avg ± SD of RCP and RCY	88.0 ± 8.9% & 85.7 ± 8.4%

Supplementary Figure 43. A [^{11}C]4BrAn, **[^{11}C]16b,** crude product sample analytical HPLC profile

HPLC analysis conditions: stationary phase: Phenomenex Luna(2) C18 150 × 4.6 mm; Mobile phase: phase A (0.1% formic acid), phase B (90% CH_3CN/10% H_2O); UV: 254 nm; Flow rate: 1.0 mL/min; Gradient/isocratic method: 70%B/30%A (0-12 min), rise 70%B to 100%B (12-13 min), 100%B (13-21 min), drop 100%B to 70%B (21-22 min), 70%B (22-25 min).

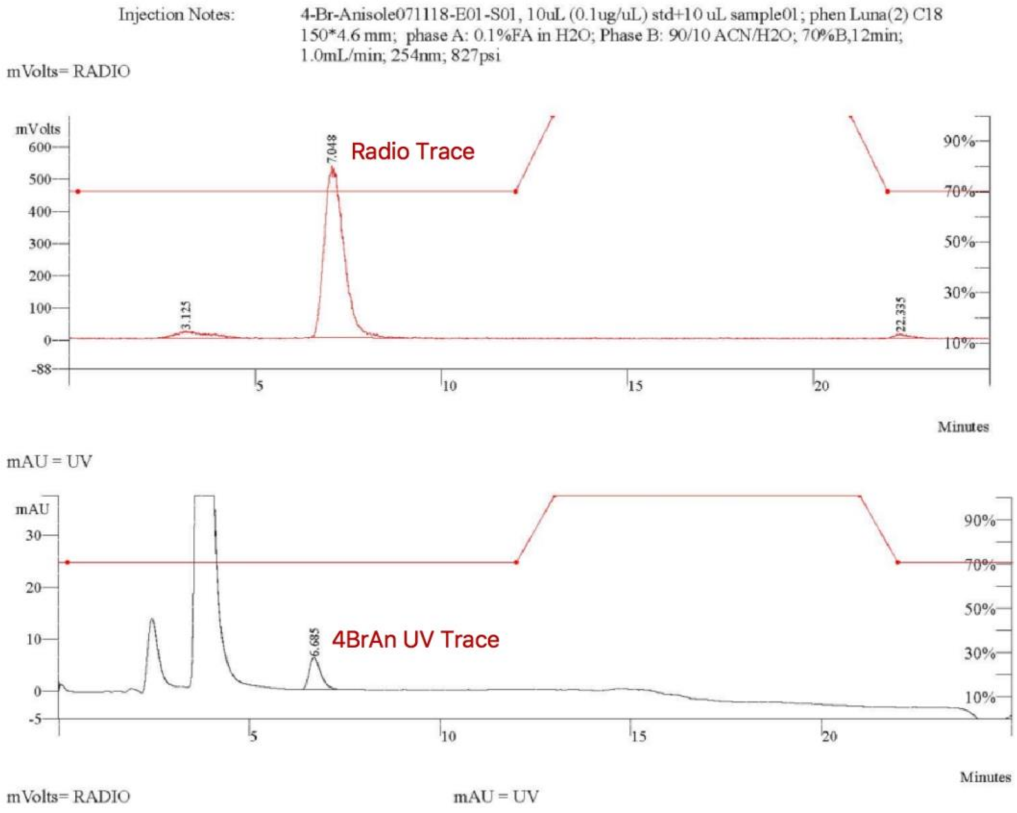

1-Bromo-2-(methoxy-^{11}C)benzene, ([^{11}C]16c) synthesis

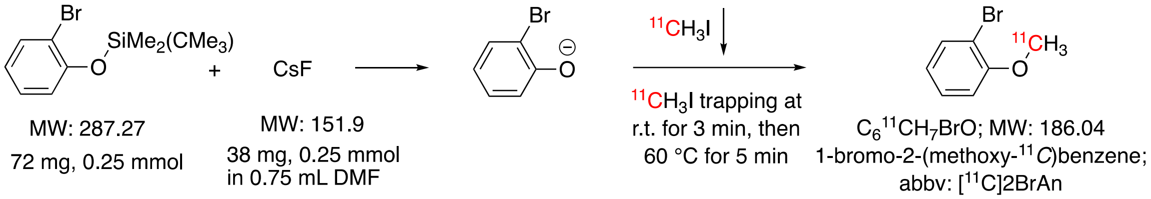

Following the procedures of pre-labeling work and ^{11}C-methylation reaction described above, [^{11}C]2BrAn, **[^{11}C]16c**, was synthesized (see above scheme for details) and the detailed results are listed in the table below:

Supplementary Table 44. Summarization of **[^{11}C]16c** synthesis experimental results

No.	RCP and RCY
Exp 01	85.2% & 83.7%
Exp 02	81.6% & 80.5%
Avg ± SD of RCP and RCY	83.4 ± 2.6% & 82.1 ± 2.3%

Supplementary Figure 44. A [^{11}C]2BrAn, **[^{11}C]16c,** crude product sample analytical HPLC profile

HPLC analysis conditions: stationary phase: Phenomenex Luna(2) C18 150 × 4.6 mm; Mobile phase: phase A (0.1% formic acid), phase B (90% CH_3CN/10% H_2O); UV: 254 nm; Flow rate: 1.0 mL/min; Gradient/isocratic method: 60%B/40%A (0-10 min), rise 60%B to 100%B (10-11 min), 100%B (11-22 min), drop 100%B to 60%B (22-23 min), 60%B (23-25 min).

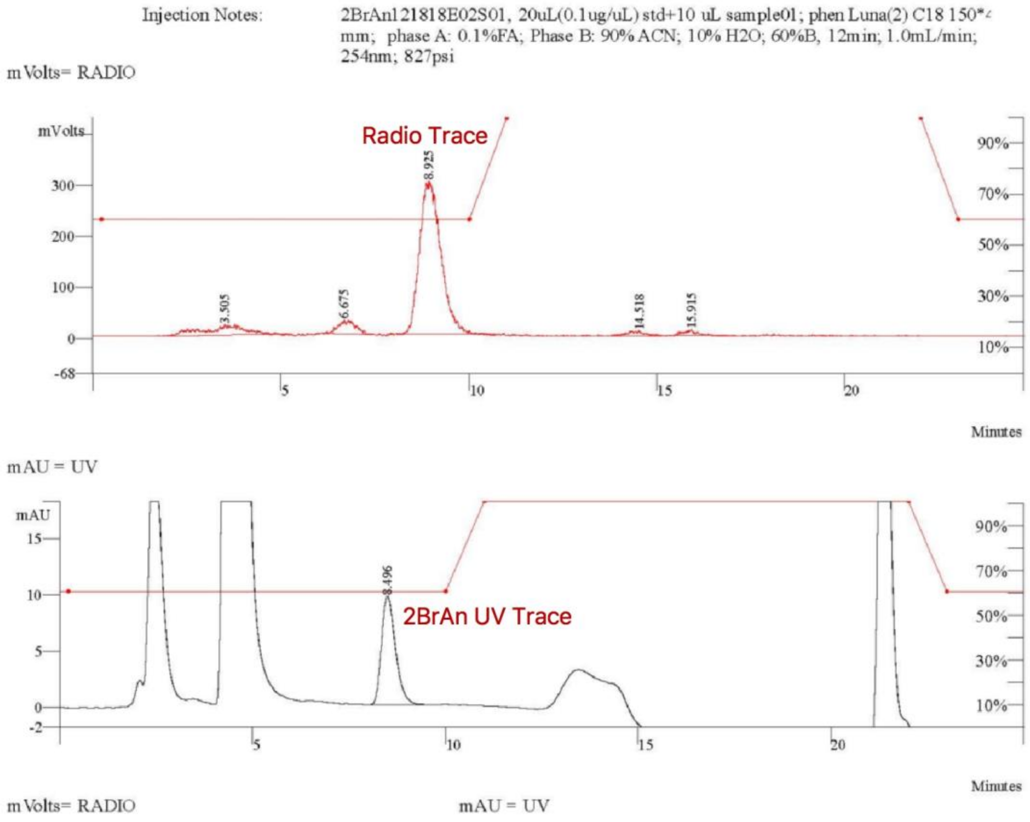

1-Methoxy-4-(methoxy-^{11}C)benzene, ([^{11}C]16d) synthesis

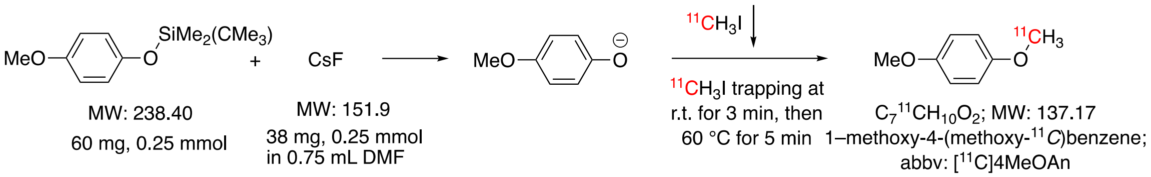

Following the procedures of pre-labeling work and ^{11}C-methylation reaction described above, [^{11}C]4MeOAn, **[^{11}C]16d**, was synthesized (see above scheme for details) and the detailed results are listed in the table below:

Supplementary Table 45. Summarization of **[^{11}C]16d** synthesis experimental results

No.	RCP and RCY
Exp 01	74.5% & 74.5%
Exp 02	75.9% & 75.9%
Exp 03	68.7% & 68.7%
Avg ± SD of RCP and RCY	73.0 ± 3.8% & 73.0 ± 3.8%

Supplementary Figure 45. A [^{11}C]4MeOAn, **[^{11}C]16d,** crude product sample analytical HPLC profile

HPLC analysis conditions: stationary phase: Phenomenex Luna(2) C18 150 × 4.6 mm; Mobile phase: phase A (0.1% formic acid), phase B (90% CH_3CN/10% H_2O); UV: 254 nm; Flow rate: 1.0 mL/min; Gradient/isocratic method: 60%B/40%A (0-10 min), rise 60%B to 100%B (10-11 min), 100%B (11-26 min), drop 100%B to 60%B (26-27 min), 60%B (27-30 min).

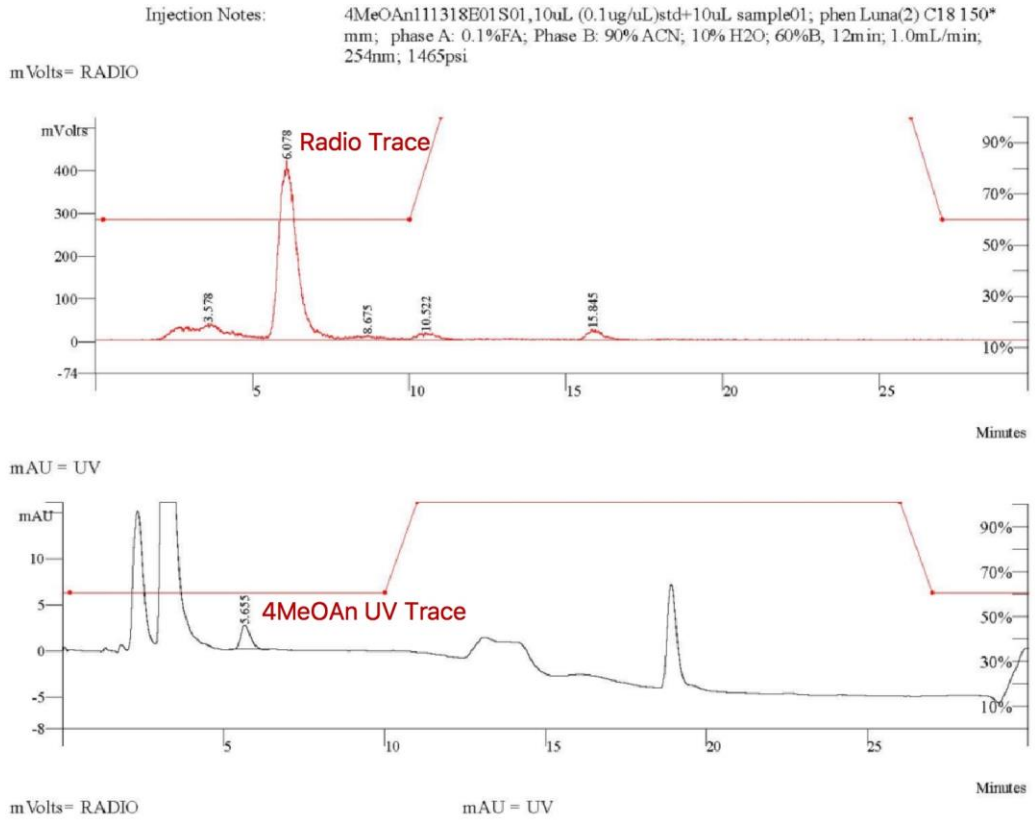

1-Methoxy-3-(methoxy-^{11}*C*)benzene, ([^{11}C]16e) synthesis

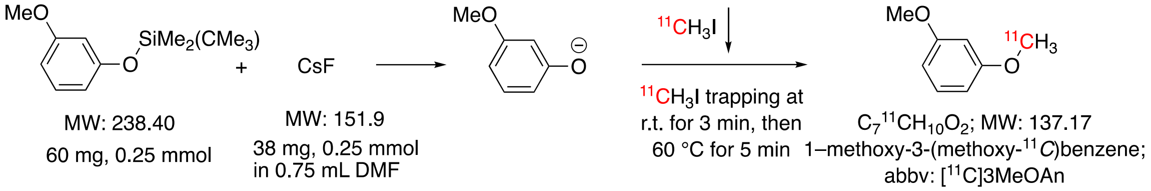

Following the procedures of pre-labeling work and ^{11}C-methylation reaction described above, [^{11}C]3MeOAn, **[^{11}C]16e**, was synthesized (see above scheme for details) and the detailed results are listed in the table below:

Supplementary Table 46. Summarization of **[^{11}C]16e** synthesis experimental results

No.	RCP and RCY
Exp 01	84.5% & 84.0%
Exp 02	66.5% & 64.8%
Exp 03	66.2% & 66.2%
Avg ± SD of RCP and RCY	72.4 ± 10.5% & 71.7 ± 10.7%

Supplementary Figure 46. A [^{11}C]3MeOAn, **[^{11}C]16e,** crude product sample analytical HPLC profile

HPLC analysis conditions: stationary phase: Phenomenex Luna(2) C18 150 × 4.6 mm; Mobile phase: phase A (0.1% formic acid), phase B (90% CH_3CN/10% H_2O); UV: 254 nm; Flow rate: 1.0 mL/min; Gradient/isocratic method: 60%B/40%A (0-10 min), rise 60%B to 100%B (10-11 min), 100%B (11-22 min), drop 100%B to 60%B (22-23 min), 60%B (23-25 min).

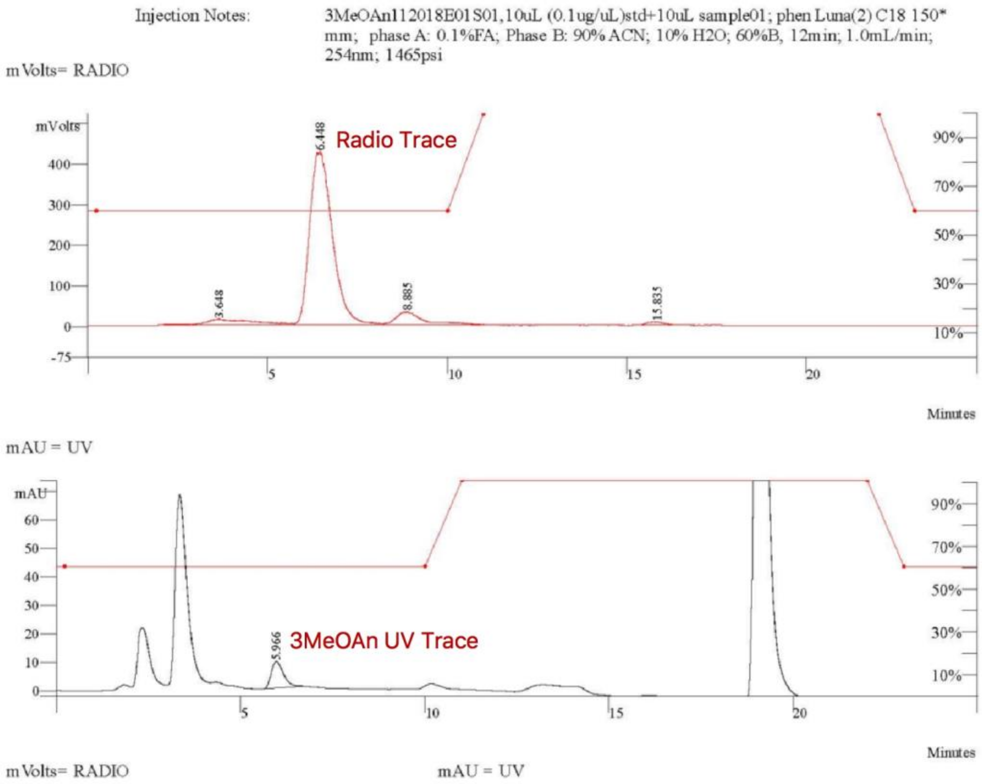

Methyl 4-(methoxy-^{11}*C*)benzoate, ([^{11}C]16f) synthesis

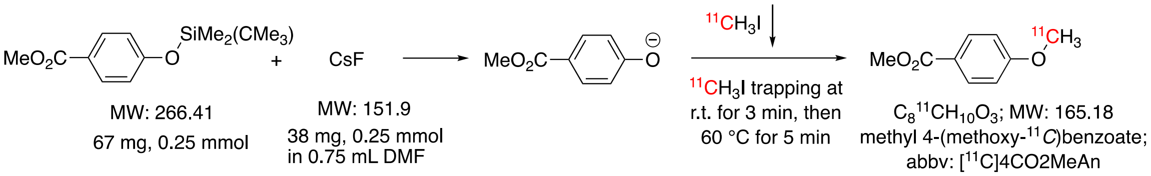

Following the procedures of pre-labeling work and ^{11}C-methylation reaction described above, [^{11}C]4CO2MeAn, **[^{11}C]16f**, was synthesized (see above scheme for details) and the detailed results are listed in the table below:

Supplementary Table 47. Summarization of **[^{11}C]16f** synthesis experimental results

No.	RCP and RCY
Exp 01	88.1% & 85.3%
Exp 02	87.3% & 85.3%
Avg ± SD of RCP and RCY	87.7 ± 0.6% & 85.3 ± 0%

Supplementary Figure 47. A [^{11}C]4CO2MeAn, **[^{11}C]16f,** crude product sample analytical HPLC profile

HPLC analysis conditions: stationary phase: Phenomenex Luna(2) C18 150 × 4.6 mm; Mobile phase: phase A (0.1% formic acid), phase B (90% CH_3CN/10% H_2O); UV: 254 nm; Flow rate: 1.0 mL/min; Gradient/isocratic method: 55%B/45%A (0-12 min), rise 55%B to 100%B (12-13 min), 100%B (13-23 min).

Description: 4CO2MeAn013019E01S01, 10 uL (0.01ug/uL) std+Sample01, Phen Luna(2) C18 150*4.6 mm; Phase A: 0.1% formic acid; Phase B: 90%ACN, 10% H2O; 55%B, 12 min; 1.0mL/min; 254nm; 83.67bar

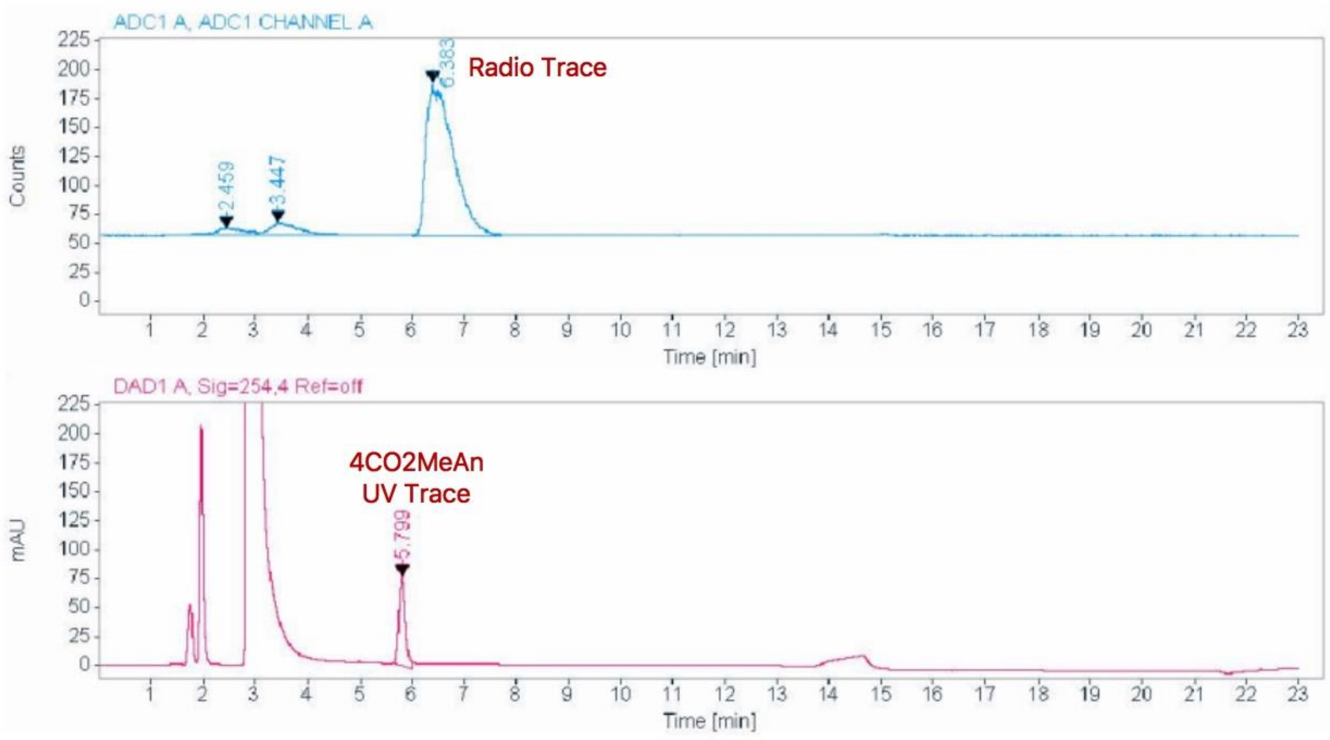

1-Fluoro-4-(methoxy-^{11}C)benzene, ([^{11}C]16g) synthesis

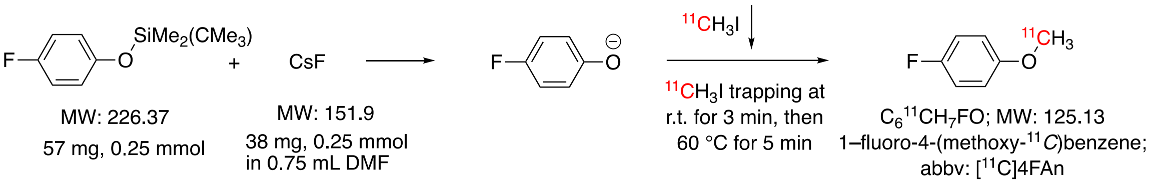

Following the procedures of pre-labeling work and ^{11}C-methylation reaction described above, [^{11}C]4FAn, **[^{11}C]16g**, was synthesized (see above scheme for details) and the detailed results are listed in the table below:

Supplementary Table 48. Summarization of **[^{11}C]16g** synthesis experimental results

No.	RCP and RCY
Exp 01	96.9% & 96.9%
Exp 02	87.0% & 87.0%
Exp 03	87.1% & 87.1%
Avg ± SD of RCP and RCY	90.3 ± 5.7% & 90.3 ± 5.7%

Supplementary Figure 48. A [^{11}C]4FAn, **[^{11}C]16g,** crude product sample analytical HPLC profile

HPLC analysis conditions: stationary phase: Phenomenex Luna(2) C18 150 × 4.6 mm; Mobile phase: phase A (0.1% formic acid), phase B (90% CH_3CN/10% H_2O); UV: 254 nm; Flow rate: 1.0 mL/min; Gradient/isocratic method: 60%B/40%A (0-10 min), rise 60%B to 100%B (10-11 min), 100%B (11-26 min), drop 100%B to 60%B (26-27 min), 60%B (27-30 min).

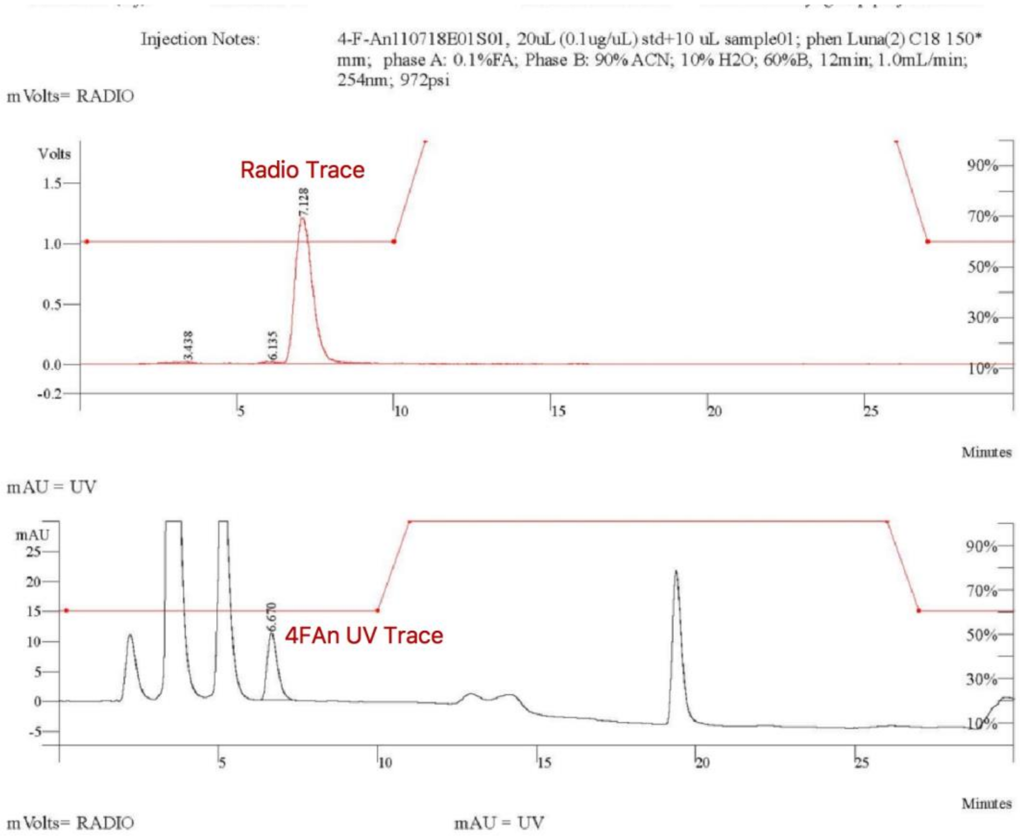

1-Bromo-4,5-dimethoxy-2-(methoxy-^{11}C)benzene, ([^{11}C]16h) synthesis

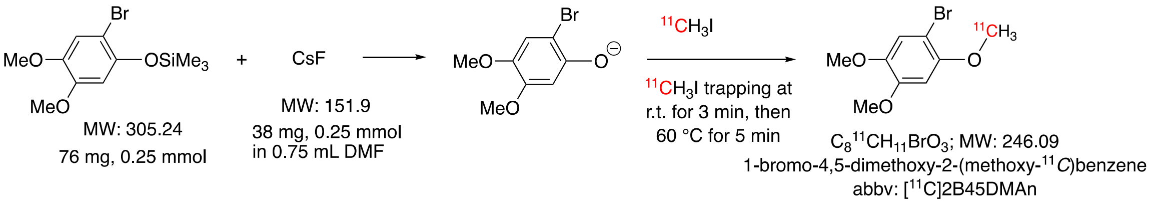

Following the procedures of pre-labeling work and ^{11}C-methylation reaction described above, [^{11}C]2B45DMAn, **[^{11}C]16h**, was synthesized (see above scheme for details) and the detailed results are listed in the table below:

Supplementary Table 49. Summarization of **[^{11}C]16h** synthesis experimental results

No.	RCP and RCY
Exp 01	84.3% & 84.3%
Exp 02	80.0% & 78.9%
Exp 03	83.0% & 82.3%
Avg ± SD of RCP and RCY	82.4 ± 2.2% & 81.8 ± 2.7%

Supplementary Figure 49. A [^{11}C]2B45DMAn, **[^{11}C]16h,** crude product sample analytical HPLC profile

HPLC analysis conditions: stationary phase: Phenomenex Luna(2) C18 150 × 4.6 mm; Mobile phase: phase A (0.1% formic acid), phase B (90% CH_3CN/10% H_2O); UV: 254 nm; Flow rate: 1.0 mL/min; Gradient/isocratic method: 50%B/50%A (0-12 min), rise 50%B to 100%B (12-13 min), 100%B (13-23 min).

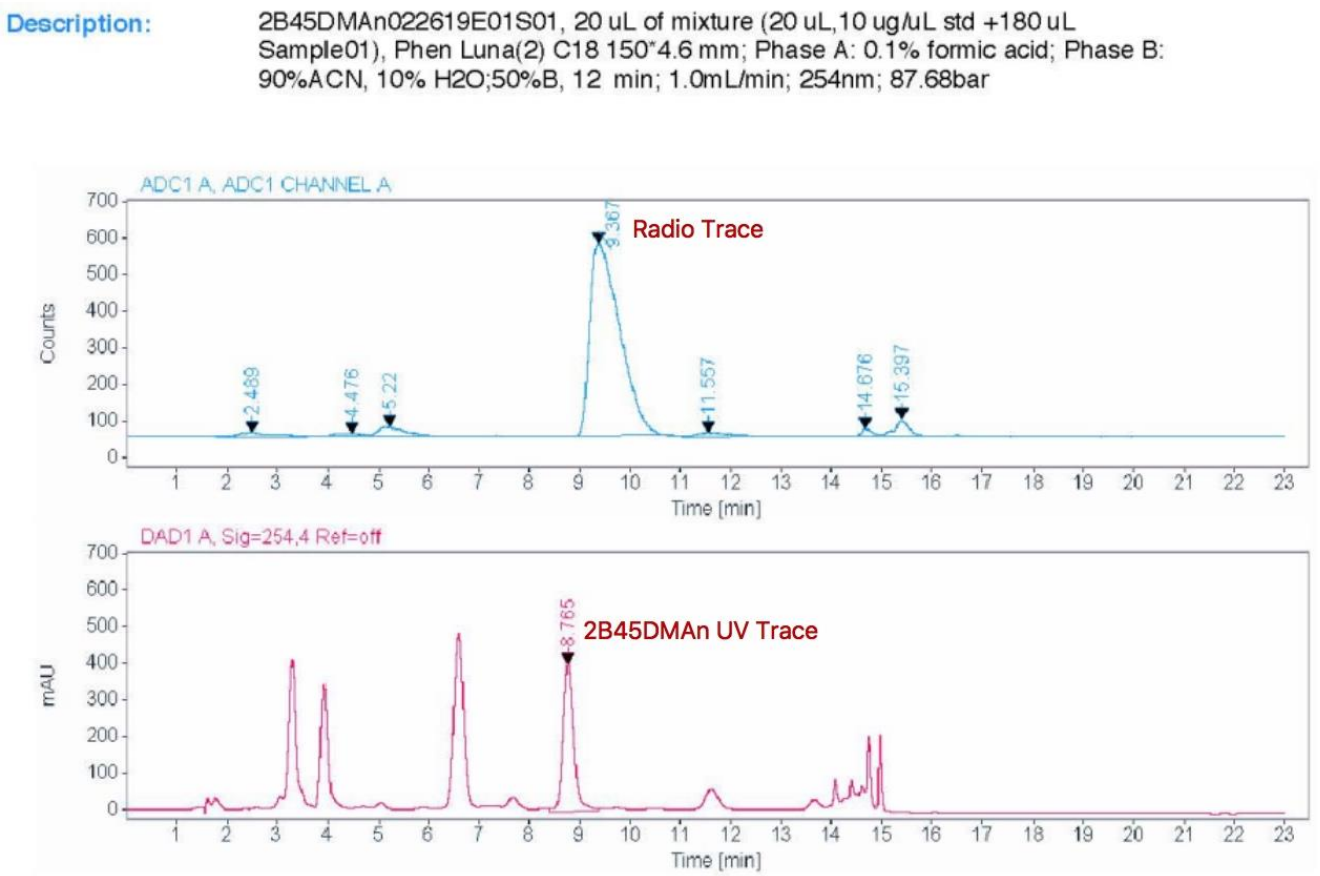

2-(Methoxy-^{11}C)naphthalene, ([^{11}C]16i) synthesis

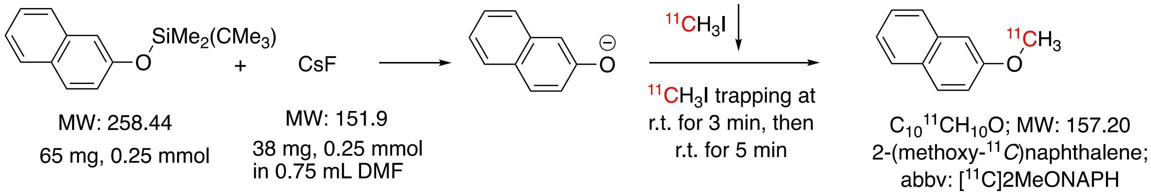

Following the procedures of pre-labeling work and ^{11}C-methylation reaction described above, [^{11}C]2MeONAPH, **[^{11}C]16i**, was synthesized (see above scheme for details) and the detailed results are listed in the table below:

Supplementary Table 50. Summarization of **[^{11}C]16i** synthesis experimental results

No.	RCP and RCY
Exp 01	34.9% & 34.9%
Exp 02	35.0% & 35.0%
Exp 03	58.8% & 58.8%
Avg ± SD of RCP and RCY	42.9 ± 13.8% & 42.9 ± 13.8%

Supplementary Figure 50. A [^{11}C]2MeONAPH, **[^{11}C]16i,** crude product sample analytical HPLC profile

HPLC analysis conditions: stationary phase: Phenomenex Luna(2) C18 150 × 4.6 mm; Mobile phase: phase A (0.1% formic acid), phase B (90% CH_3CN/10% H_2O); UV: 254 nm; Flow rate: 1.0 mL/min; Gradient/isocratic method: 70%B/30%A (0-12 min), rise 70%B to 100%B (12-13 min), 100%B (13-18 min), drop 100%B to 70%B (18-19 min), 70%B (19-22 min).

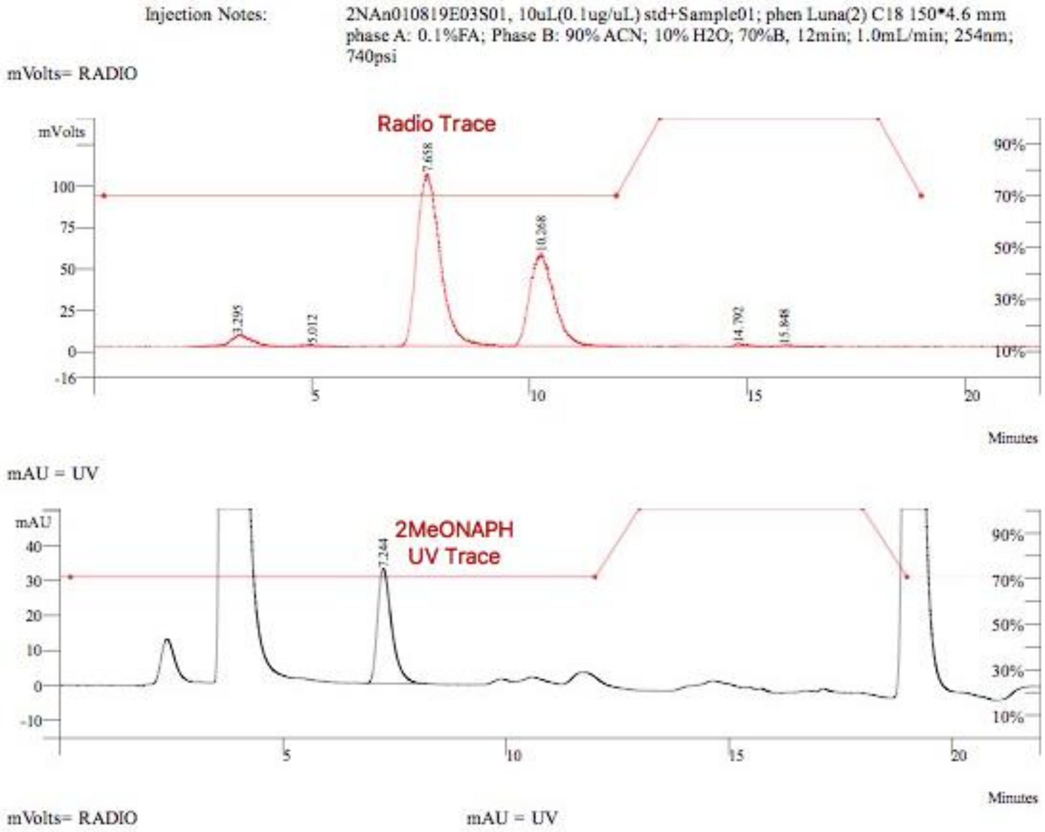

[^{11}C]Thioanisole ([^{11}C]TA, [^{11}C]16j) synthesis

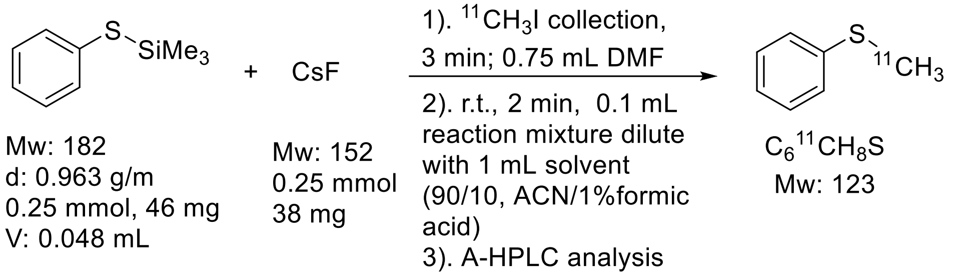

Following the procedures of pre-labeling work and ^{11}C-methylation reaction described above, [^{11}C]thioanisole ([^{11}C]TA, **[^{11}C]16j**) was synthesized and the detailed results are listed in the table below:

Supplementary Table 51. Summarization of **[^{11}C]16j** synthesis experimental results

No.	RCP and RCY
Exp 01	98.4% & 98.4%
Exp 02	87.2% & 87.2%
Exp 03	88.3% & 88.3%
Avg ± SD of RCP and RCY	91.3 ± 6.2% & 91.3 ± 6.2%

Supplementary Figure 51. A [^{11}C]TA, **[^{11}C]16j,** crude product sample analytical HPLC profile

HPLC analysis conditions: stationary phase: Phenomenex Luna C18 150 × 4.6 mm; Mobile phase: phase A (0.1% formic acid), phase B (90% CH_3CN/10% H_2O); UV: 254 nm; Flow rate: 1.0 mL/min; Gradient/isocratic method: 65%B/35%A (0-10 min), rise 65%B to 100%B (10-11 min), 100%B (11-27 min), drop 100%B to 65%B (27-28 min).

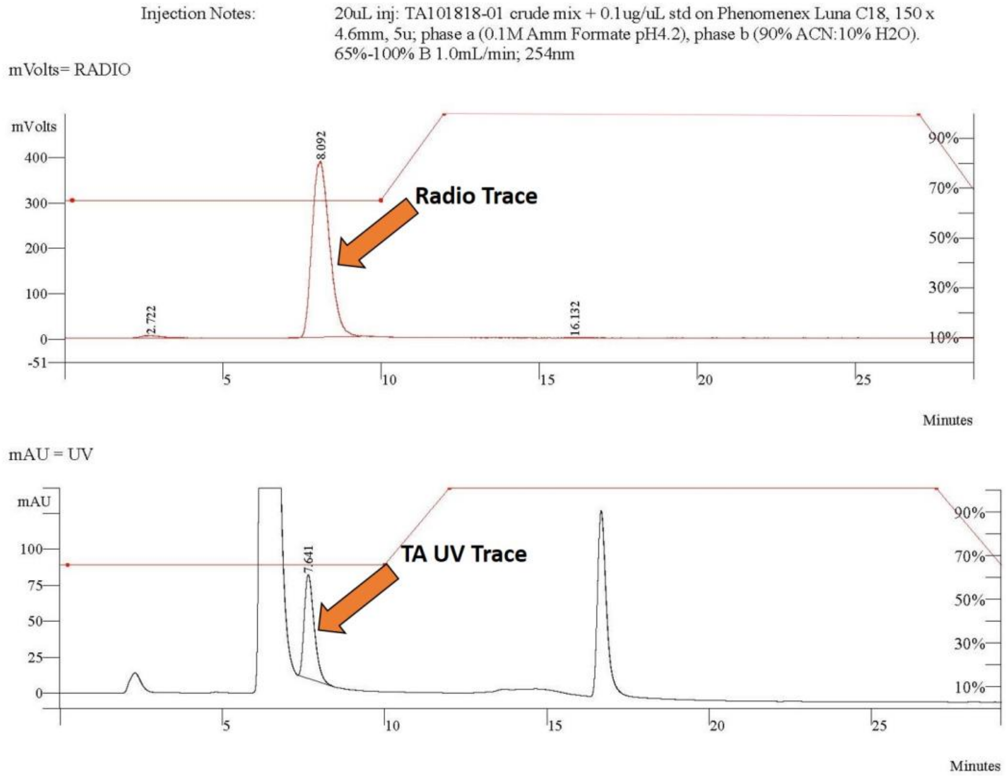

(**(Methoxy-^{11}C)methyl)benzene, ([^{11}C]16k) synthesis**

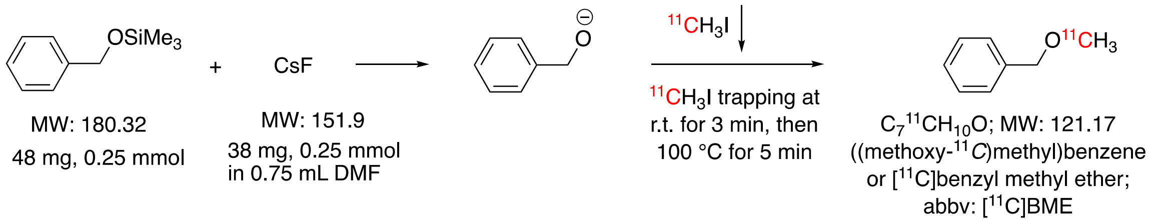

Following the procedures of pre-labeling work and ^{11}C-methylation reaction described above, [^{11}C]BME (**[^{11}C]16k)** was synthesized (see above scheme for details) and the detailed results are listed in the table below:

Supplementary Table 52. Summarization of **[^{11}C]16k** synthesis experimental results

No.	RCP and RCY
Exp 01	38.9% & 37.3%
Exp 02	35.1% & 34.1%
Exp 03	31.2% & 26.6%
Avg ± SD of RCP and RCY	35.1 ± 3.9% & 32.7 ± 5.5%

Supplementary Figure 52. A [^{11}C]BME, **[^{11}C]16k,** crude product sample analytical HPLC profile

HPLC analysis conditions: stationary phase: Phenomenex Luna(2) C18 150 × 4.6 mm; Mobile phase: phase A (0.1% formic acid), phase B (90% CH_3CN/10% H_2O); UV: 215 nm; Flow rate: 1.0 mL/min; Gradient/isocratic method: 50%B/50%A (0-12 min), rise 50%B to 100%B (12-13 min), 100%B (13-20 min).

Description: BME021219E01S01, 20 uL of mixuture (40 uL 10ug/uL sdt+ 200uL Sample01), Phen Luna(2) C18 150*4.6 mm; Phase A: 0.1% formic acid; Phase B: 90%ACN, 10% H2O; 50%B, 12 min; 1.0mL/min; 215nm; 85.80bar

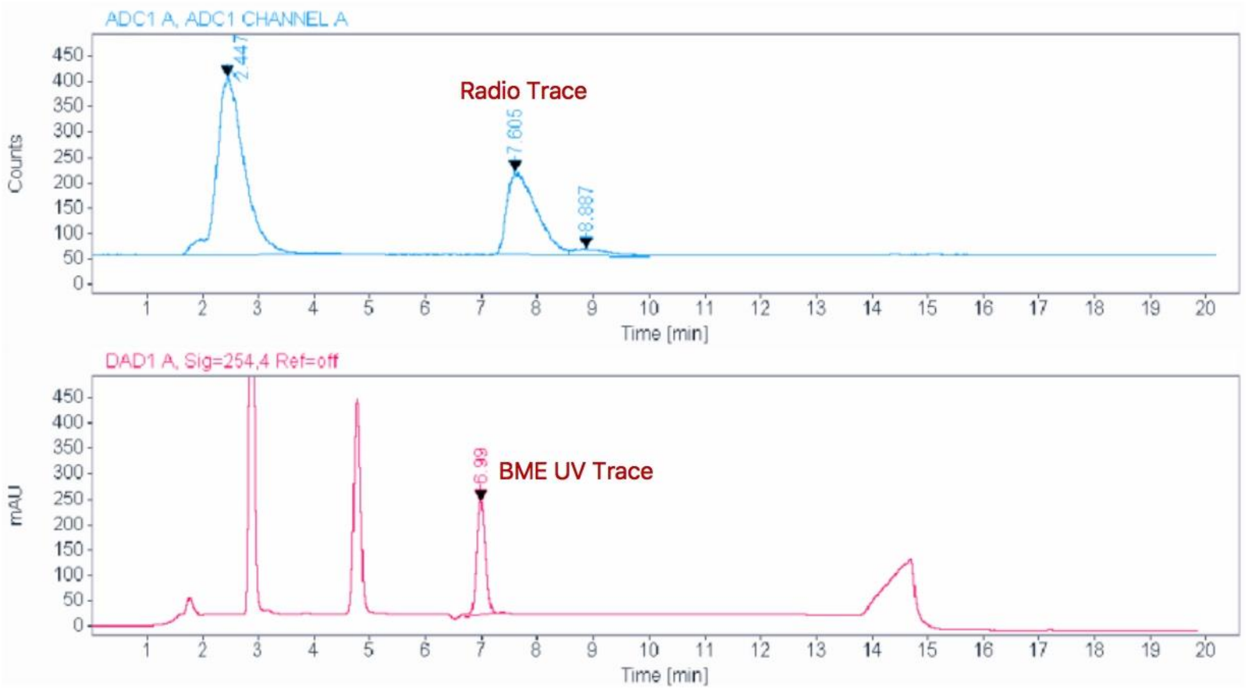

N-methyl-*N*-(methyl-^{11}C)acetamide, ([^{11}C]16l) synthesis

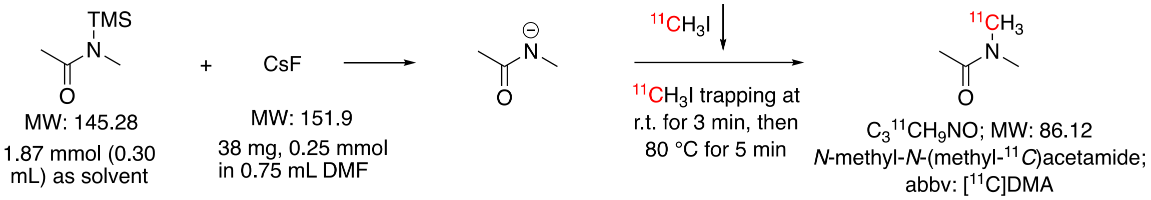

Following the procedures of pre-labeling work and ^{11}C-methylation reaction described above, [^{11}C]DMA **([^{11}C]16l)** was synthesized (see above scheme for details) and the detailed results are listed in the table below:

Supplementary Table 53. Summarization of **[^{11}C]16l** synthesis experimental results

No.	RCP and RCY
Exp 01	92.6% & 91.1%
Exp 02	91.3% & 91.3%
Exp 03	94.7% & 94.7%
Avg ± SD of RCP and RCY	92.9 ± 1.7% & 92.4 ± 2.0%

Supplementary Figure 53. A [^{11}C]DMA, **[^{11}C]16l,** crude product sample analytical HPLC profile

HPLC analysis conditions: stationary phase: Phenomenex PFP(2) C18 250 × 4.6 mm; Mobile phase: phase A (0.1% formic acid), phase B (90% CH_3CN/10% H_2O); UV: 220 nm; Flow rate: 1.0 mL/min; Gradient/isocratic method: 5%B/95%A (0-15 min), rise 5%B to 100%B (15-16 min), 100%B (16-23 min).

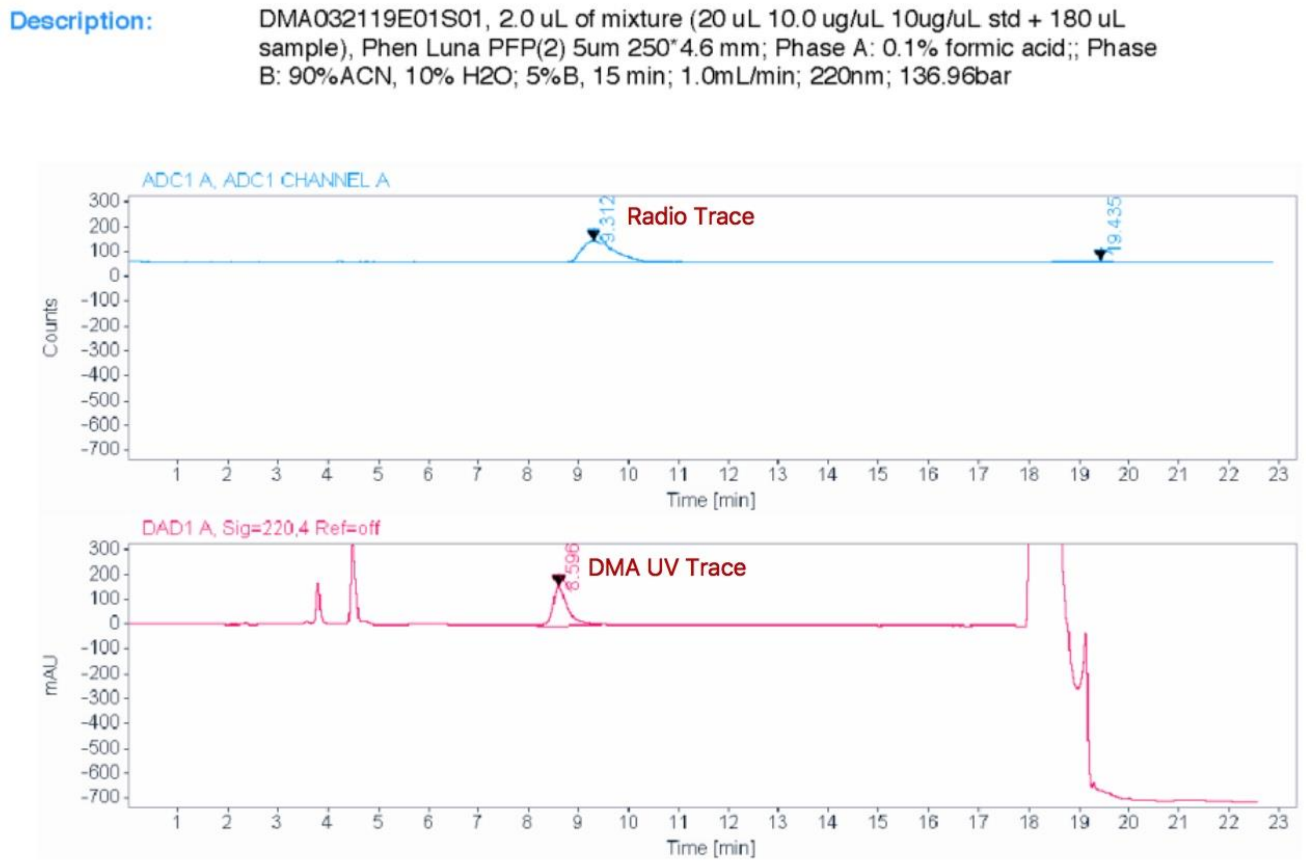

1-(Methyl-^{11}C)-1*H*-imidazole, ([^{11}C]16m) synthesis

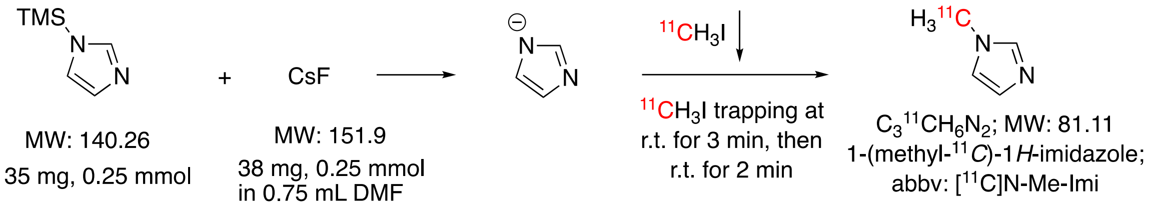

Following the procedures of pre-labeling work and ^{11}C-methylation reaction described above, [^{11}C]N-Me-Imi **([^{11}C]16m)** was synthesized (see above scheme for details) and the detailed results are listed in the table below:

Supplementary Table 54. Summarization of **[^{11}C]16m** synthesis experimental results

No.	RCP and RCY
Exp 01	94.5% & 94.4%
Exp 02	94.5% & 90.0%
Exp 03	97.4% & 95.9%
Avg ± SD of RCP and RCY	95.5 ± 1.7% & 93.4 ± 3.1%

Supplementary Figure 54. A [^{11}C]N-Me-Imi, **[^{11}C]16m,** crude product sample analytical HPLC profile

HPLC analysis conditions: stationary phase: Phenomenex Luna(2) C18 150 × 4.6 mm; Mobile phase: phase A (200 mM NH_4OH + 100 mM HOAc (PH = 9.5)), phase B (90% CH_3CN/10% H_2O); UV: 220 nm; Flow rate: 1.0 mL/min; Gradient/isocratic method: 1%B/99%A (0-12 min), rise 1%B to 100%B (12-13 min), 100%B (13-23 min).

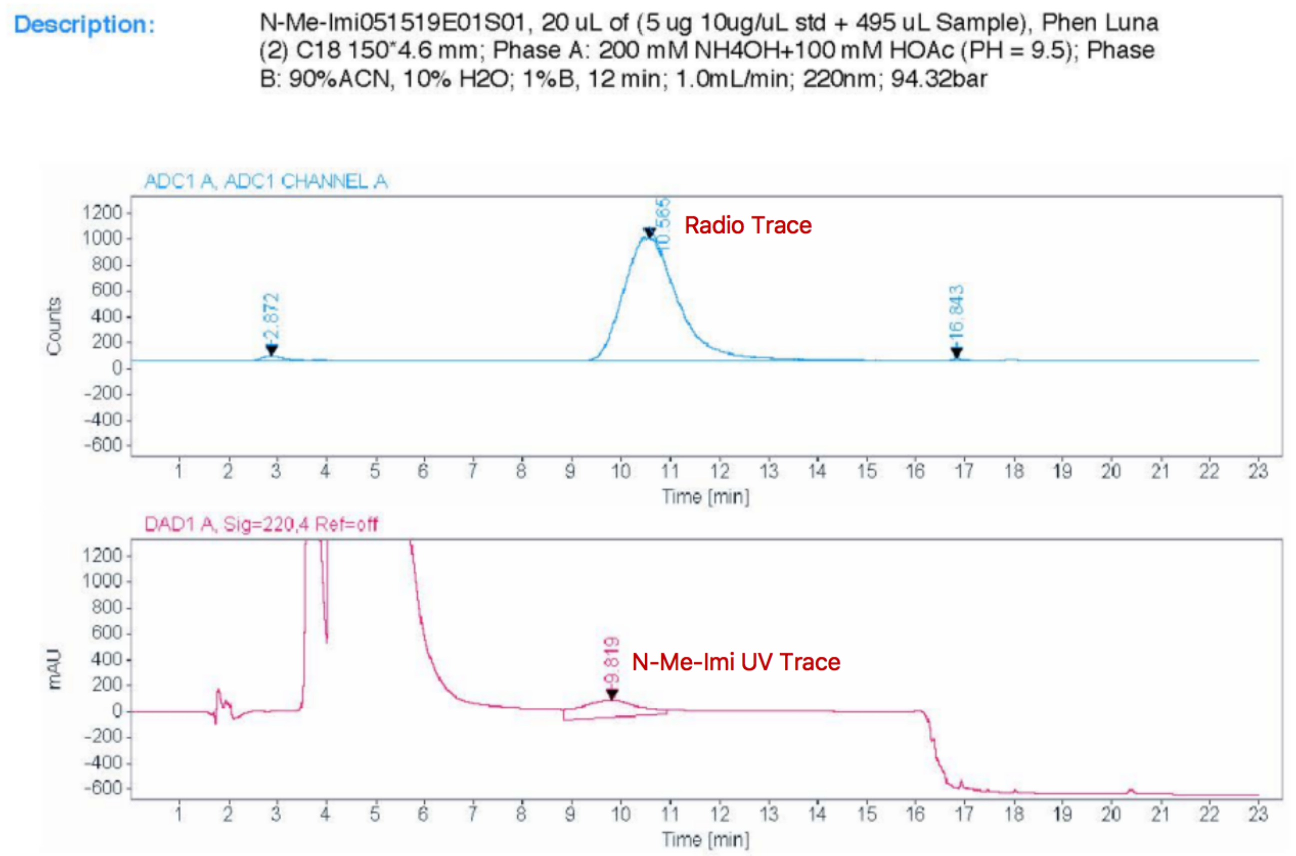

2-(Methyl-^{11}C)benzo[*d*]thiazole, ([^{11}C]16n) synthesis

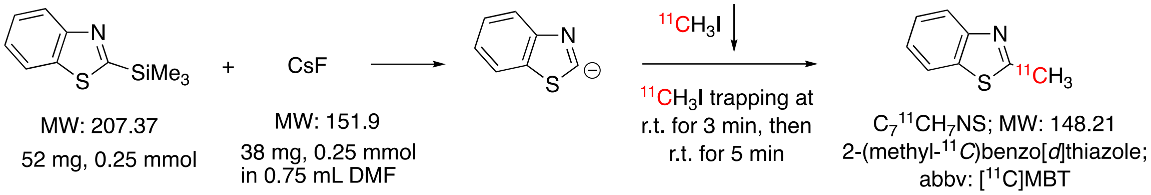

Following the procedures of pre-labeling work and ^{11}C-methylation reaction described above, [^{11}C]MBT (**[^{11}C]16n**) was synthesized (see above scheme for details) and the detailed results are listed in the table below:

Supplementary Table 55. Summarization of **[^{11}C]16n** synthesis experimental results

No.	RCP and RCY
Exp 01	44.6% & 43.3%
Exp 02	61.3% & 60.0%
Exp 03	48.9% & 47.1%
Avg ± SD of RCP and RCY	51.6 ± 8.7% & 50.1 ± 8.8%

Supplementary Figure 55. A [^{11}C]MBT, **[^{11}C]16n,** crude product sample analytical HPLC profile

HPLC analysis conditions: stationary phase: Phenomenex Luna(2) C18 150 × 4.6 mm; Mobile phase: phase A (0.1% formic acid), phase B (90% CH_3CN/10% H_2O); UV: 254 nm; Flow rate: 1.0 mL/min; Gradient/isocratic method: 50%B/50%A (0-10 min), rise 50%B to 100%B (10-11 min), 100%B (11-18 min), drop 100%B to 50%B (18-19 min), 50%B (19-22 min).

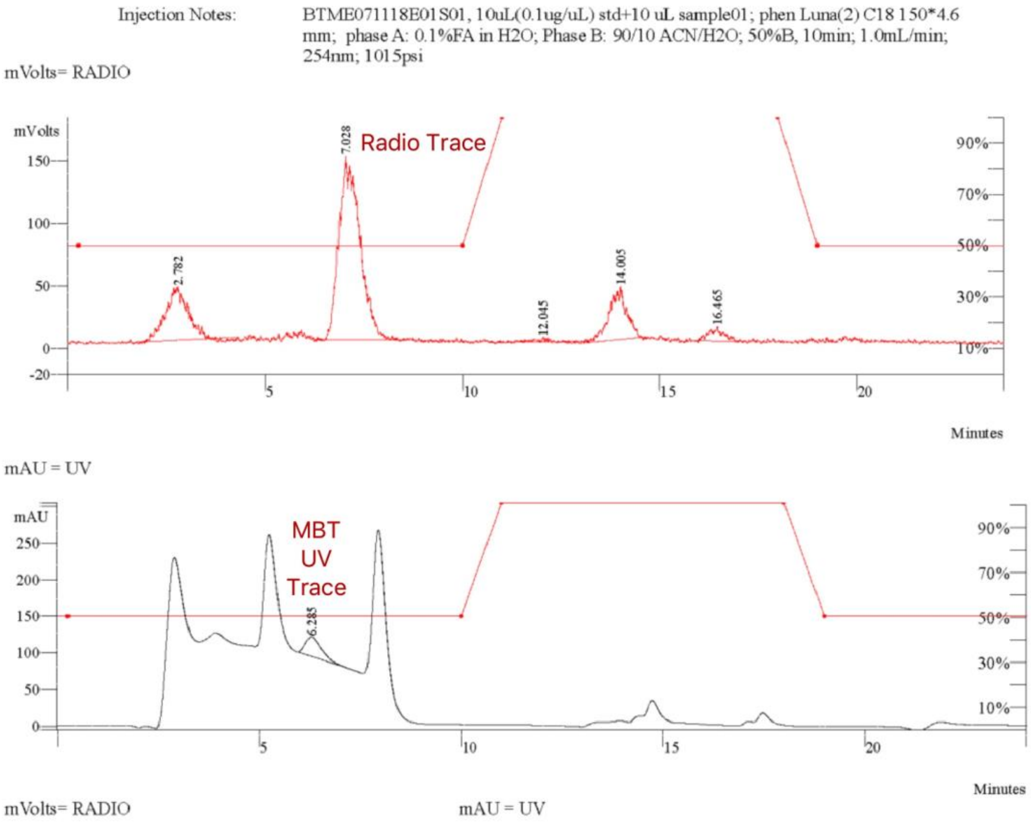

Supplementary Figure 56. A [^{12}C]MBT, **[^{12}C]16n,** standard sample analytical HPLC profile

HPLC analysis conditions: stationary phase: Phenomenex Luna(2) C18 150 × 4.6 mm; Mobile phase: phase A (0.1% formic acid), phase B (90% CH_3CN/10% H_2O); UV: 254 nm; Flow rate: 1.0 mL/min; Isocratic method: 50%B/50%A (0-10 min).

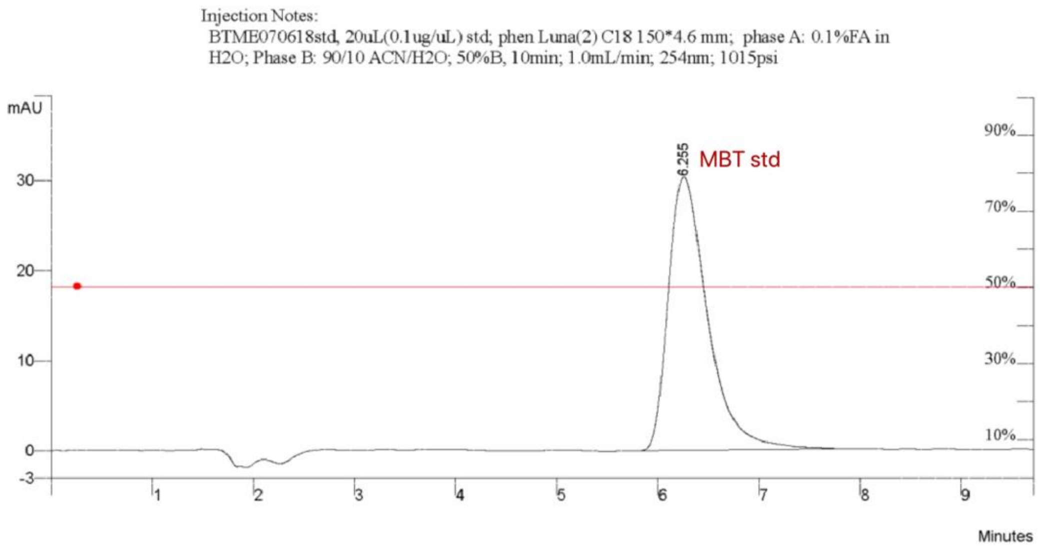

[3-^{11}C]Ibuprofen ([^{11}C]IBP), ([^{11}C]16o) synthesis

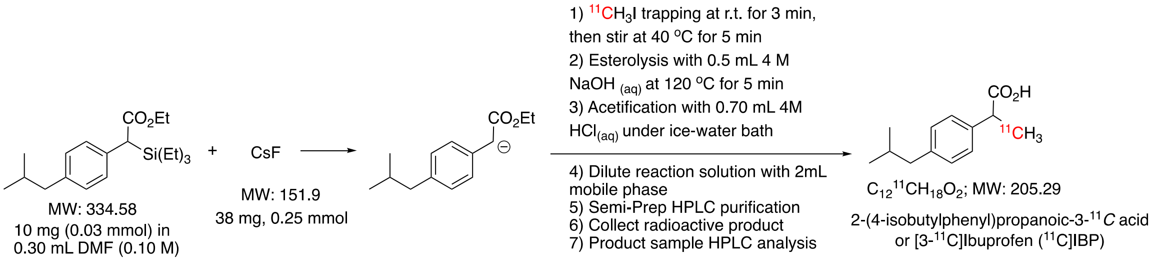

Following the procedures of pre-labeling work and ^{11}C-methylation reaction described above, [3-^{11}C]ibuprofen ([^{11}C]IBP, **[^{11}C]16o**) was synthesized (see above scheme for details) and the detailed results are listed in the table below:

Supplementary Table 56. Summarization of **[^{11}C]16o** synthesis experimental results

Exp 01 Results	Exp 02 Results	Exp 03 Results	Avg ± SD Results
[^{11}C] CH_3I: 6.51 GBq (176 mCi);<br>Final Prod: 0.40 GBq (10.9 mCi) from SP-HPLC;<br>Synthesis time: 43 min;<br>RCP: 99.0%;<br>RCY: 26.5%	[^{11}C]CH_3I: 7.34 GBq (198.3 mCi);<br>Prod: 0.58 GBq (15.8 mCi) from SP-HPLC;<br>Synthesis time: 35 min;<br>RCP: 98.7%;<br>RCY: 26.0%	[^{11}C] CH_3I: 7.73 GBq (209 mCi)<br>Prod: 0.55 GBq (14.9 mCi) from SP-HPLC;<br>Synthesis time: 38 min;<br>RCP: 98.3%;<br>RCY: 25.4%	Starting activity: 7.2 ± 0.6 GBq<br>Product: 0.5 ± 0.1 GBq<br>Synthesis time: 38.7 ± 4.0 min<br>RCP: 98.7 ± 0.4%<br>RCY: 26.0 ± 0.6%

Supplementary Figure 57. A [^{11}C]IBP, **[^{11}C]16o**, semi-prep HPLC profile

HPLC semi-Prep conditions: stationary phase: VP 250/16 Nucleosil 100-7 C18; Mobile phase: 50-0.1% formic acid), 50% CH_3CN; UV: 225 nm; Flow rate: 6.0 mL/min; Isocratic method, 18 min.

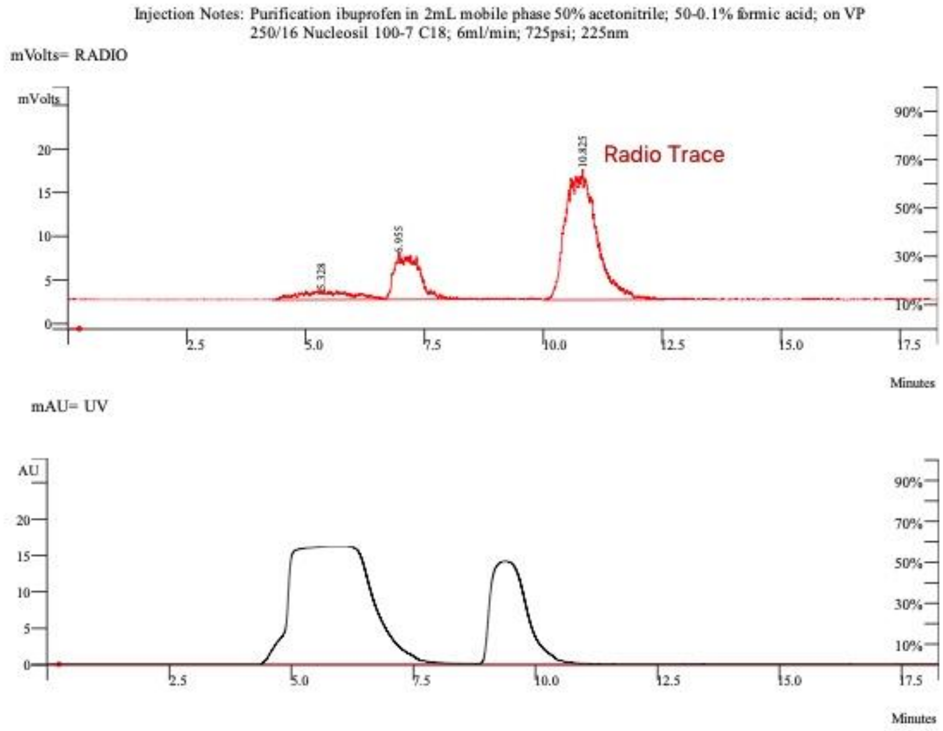

Supplementary Figure 58. An [^{12}C]IBP**, [^{12}C]16o,** standard sample semi-prep HPLC profile

HPLC semi-Prep conditions: stationary phase: VP 250/16 Nucleosil 100-7 C18; Mobile phase: 50-0.1% formic acid), 50% CH_3CN; UV: 225 nm; Flow rate: 6.0 mL/min; Isocratic method, 18 min.

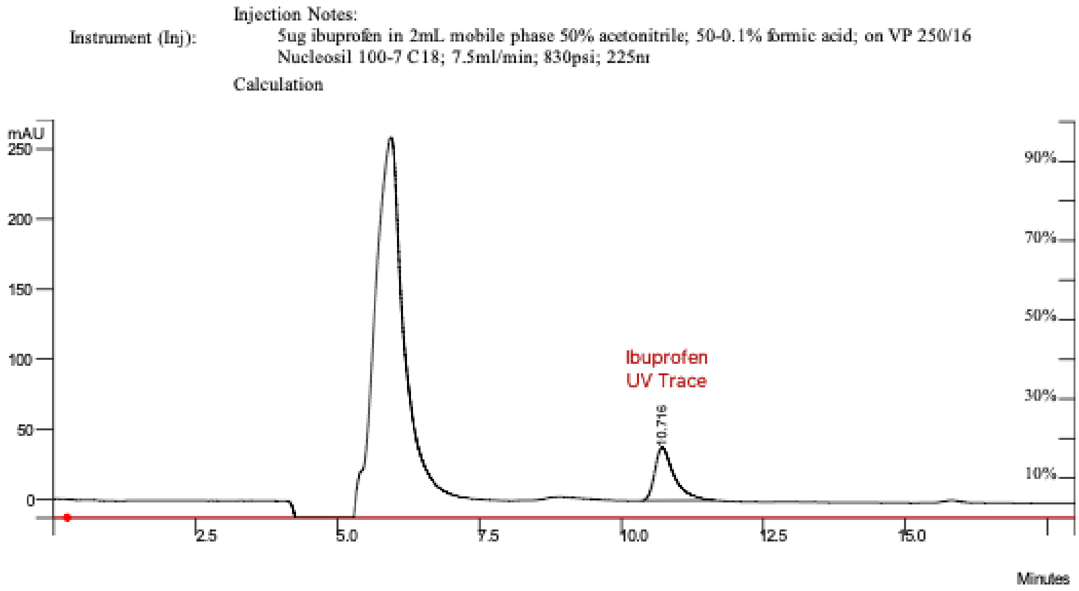

Supplementary Figure 59. An [^{11}C]IBP, **[^{11}C]16o,** final product sample analytical HPLC profile

HPLC analysis conditions: stationary phase: Phenomenex Luna(2) C18 150 × 4.6 mm; Mobile phase: phase A (0.1% formic acid), phase B (90% CH_3CN/10% H_2O); UV: 225 nm; Flow rate: 1.0 mL/min; Isocratic method: 65%B/35%A (0-15 min).

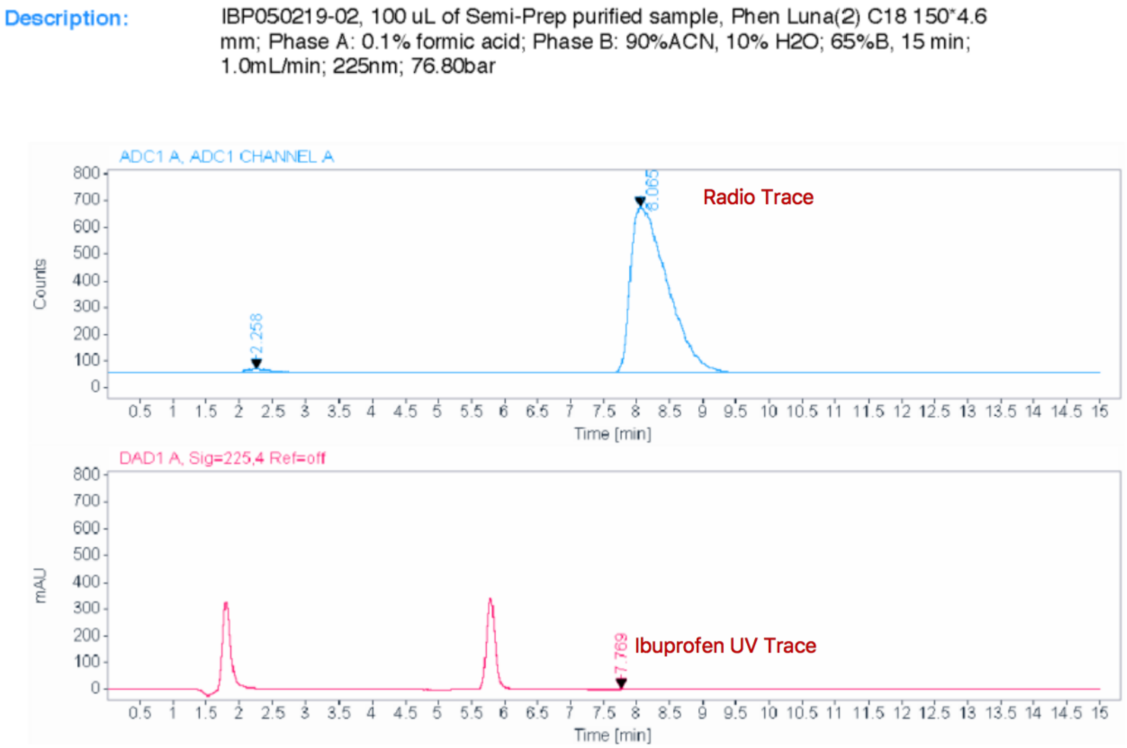

Supplementary Figure 60. An [^{12}C]IBP**, [^{12}C]16o,** standard sample analytical HPLC profile

HPLC analysis conditions: stationary phase: Phenomenex Luna(2) C18 150 × 4.6 mm; Mobile phase: phase A (0.1% formic acid), phase B (90% CH_3CN/10% H_2O); UV: 225 nm; Flow rate: 1.0 mL/min; Isocratic method: 65%B/35%A (0-15 min).

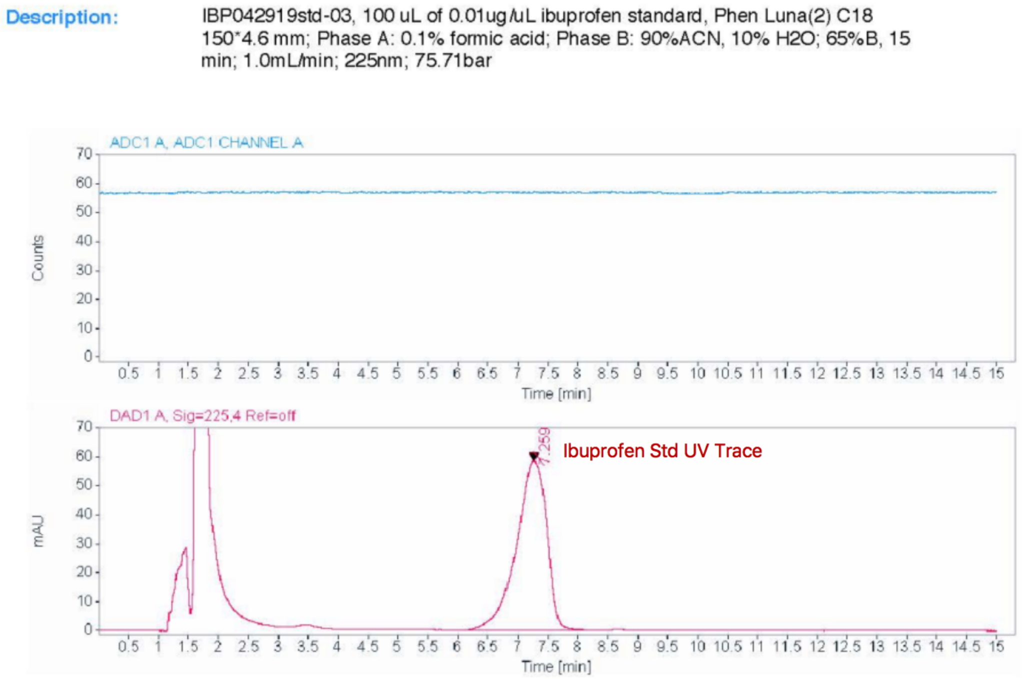

[3-^{11}C]Propiophenone ([^{11}C]PPO, [^{11}C]16p) synthesis

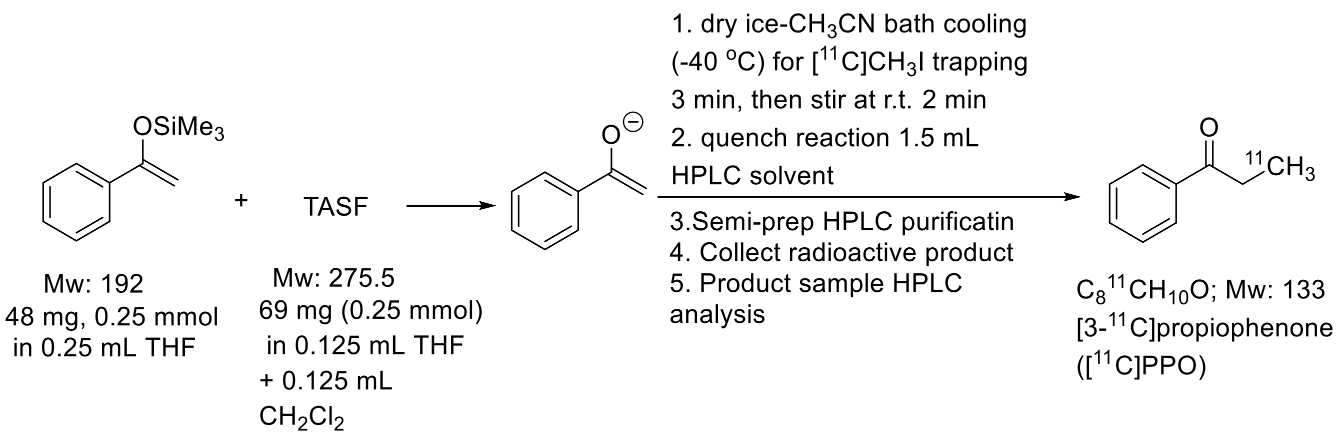

Following the procedures of pre-labeling work and ^{11}C-methylation reaction described above, [^{11}C]PPO (**[^{11}C]16p**) was synthesized (see above scheme for details) and the detailed results are listed in the table below:

Supplementary Table 57. Summarization of **[^{11}C]16p** synthesis experimental results

Exp 01 Results	Exp 02 Results	Exp 03 Results	Avg ± SD Results
[^{11}C]CH_3I: 10.3 GBq (278 mCi)<br>Prod: 3.07 GBq (83 mCi) from SP-HPLC;<br>Synthesis time: 37 min;<br>RCY: 60.9%;<br>RCP: >99%;<br>A_m: 17.1 GBq/µmol or 0.461Ci/µmol (EOB)	[^{11}C]CH_3I: 5.20 GBq (140.5 mCi)<br>Prod: 1.74 GBq (46.9 mCi) from SP-HPLC;<br>Synthesis time: 39 min;<br>RCY: 78.1%;<br>RCP: >99%;<br>A_m: 36.5 GBq/µmol or 0.986Ci/µmol (EOB)	[^{11}C]CH_3I: 6.43 GBq (173.8 mCi)<br>Prod: 2.00 GBq (53.9 mCi) from SP-HPLC;<br>Synthesis time: 37 min;<br>RCY: 65.5%;<br>RCP: >99%;<br>A_m: 35.4 GBq/µmol or 0.956Ci/µmol (EOB)	Starting activity: 7.3 ± 2.67 GBq<br>Product: 2.3 ± 0.7 GBq<br>Synthesis time: 37.7 ± 1.2 min<br>RCY: 68.2 ± 8.9%<br>RCP: 99 ± 0.0%<br>A_m: 29.7 ± 10.9 GBq/µmol

Supplementary Figure 61. A [^{11}C]PPO, **[^{11}C]16p**, semi-prep HPLC profile

HPLC analysis conditions: stationary phase: Phenomenex Luna C18 250 × 10 mm; Mobile phase: 60% 0.1M ammonium formate (PH = 4.2), 40% CH_3CN; UV: 254 nm; Flow rate: 6.0 mL/min; Isocratic method for 27min.

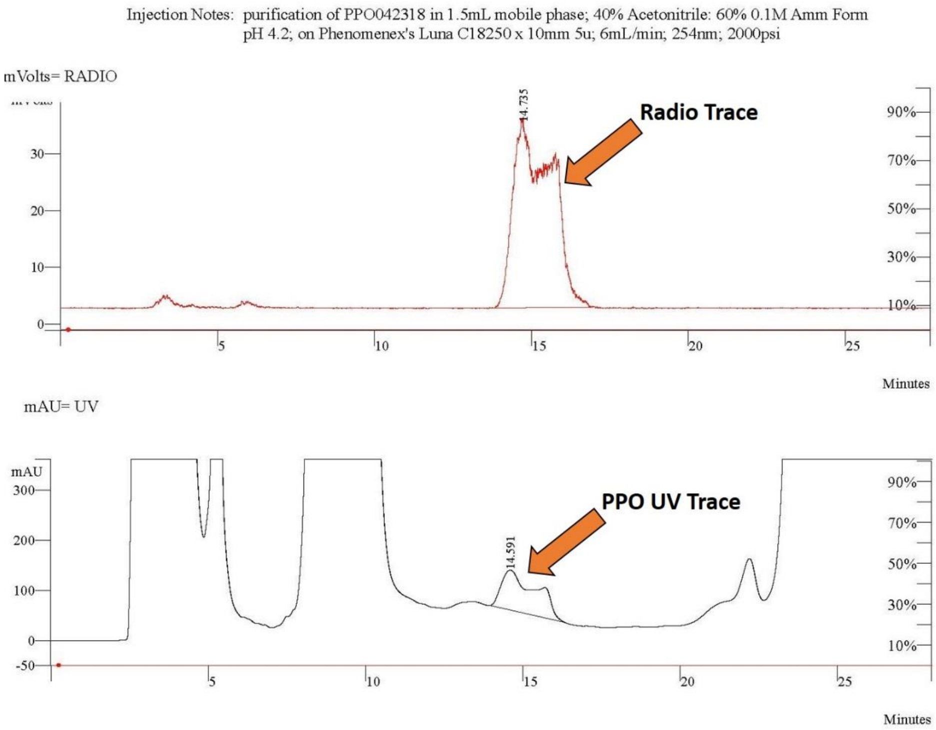

Supplementary Figure 62. A [^{11}C]PPO, **[^{11}C]16p**, final product sample analytical HPLC profile

HPLC analysis conditions: stationary phase: Phenomenex Luna C18 150 × 4.6 mm; Mobile phase: 50% 0.1M ammonium formate (PH = 4.2), 50% CH_3CN; UV: 254 nm; Flow rate: 6.0 mL/min; Isocratic method for 15 min.

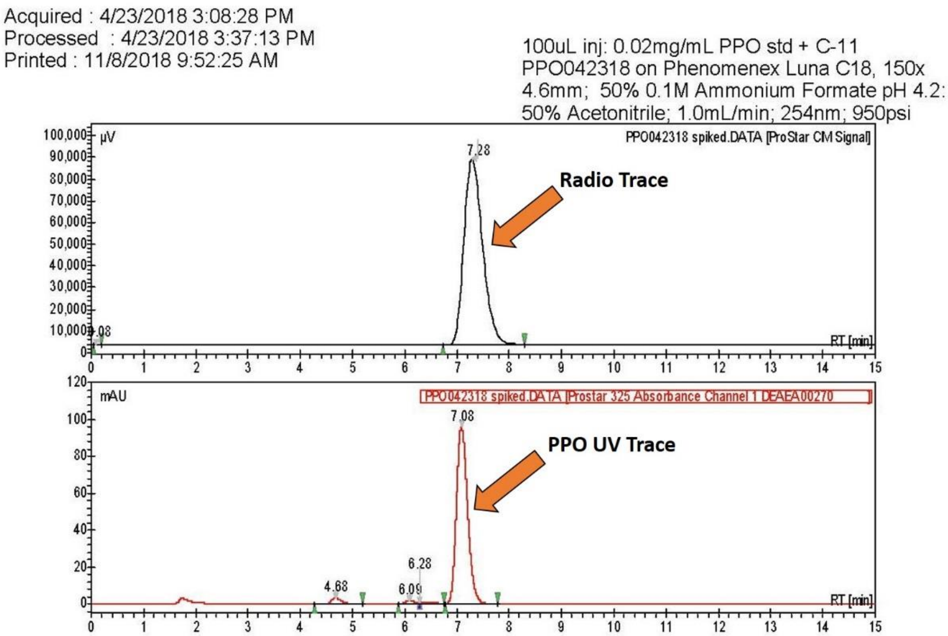

<u>(*S*)-3,5-Dichloro-*N*-((1-ethylpyrrolidin-2-yl)methyl)-2-hydroxy-6-(methoxy-^{11}*C*)benzamide, ([^{11}C]raclopride or [^{11}C]Rac, [^{11}C]18), synthesis</u>

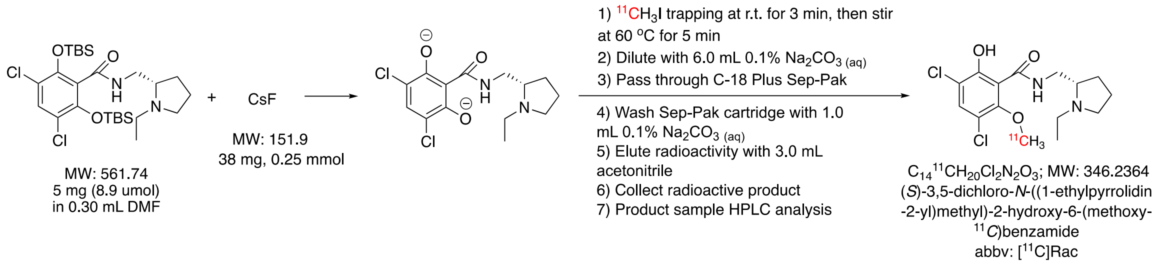

Following <u>the procedures of pre-labeling work</u> and <u>^{11}C-methylation reaction</u> described above, [^{11}C]raclopride ([^{11}C]Rac, **[^{11}C]18**) was synthesized (see above scheme for details) and the detailed results are listed in the table below:

Supplementary Table 58. Summarization of **[^{11}C]18** synthesis experimental results

Exp 01 Results	Exp 02 Results	Exp 03 Results	Avg ± SD Results
[^{11}C] CH_3I: 6.44 GBq (174.2 mCi);<br>Final Prod: 1.59 GBq (42.9 mCi);<br>Synthesis time: 20 min;<br>RCP: 61.9%;<br>RCY: 30.1%	[^{11}C]CH_3I: 4.67 GBq (126.1 mCi);<br>Prod: 1.25 GBq (33.9 mCi);<br>Synthesis time: 14 min;<br>RCP: 59.9%;<br>RCY: 25.8%	[^{11}C] CH_3I: 2.73 GBq (73.8 mCi)<br>Prod: 0.82 GBq (22.2 mCi);<br>Synthesis time: 13 min;<br>RCP: 58.9%;<br>RCY: 27.5%	Starting activity: 4.6 ± 1.9 GBq<br>Product: 1.2 ± 0.4 GBq<br>Synthesis time: 15.7 ± 3.8 min<br>RCP: 60.2 ± 1.5%<br>RCY: 27.8 ± 2.2%

Supplementary Figure 63. A [^{11}C]Rac, **[^{11}C]18,** crude product sample analytical HPLC profile

HPLC analysis conditions: stationary phase: Phenomenex Luna(2) C18 150 × 4.6 mm; Mobile phase: phase A (0.1M ammonium formate (PH = 4.2)), phase B (100% CH_3CN); UV: 235 nm; Flow rate: 1.0 mL/min; Gradient/isocratic method: 35%B/65%A (0-15 min), rise 35%B to 100%B (15-26 min).

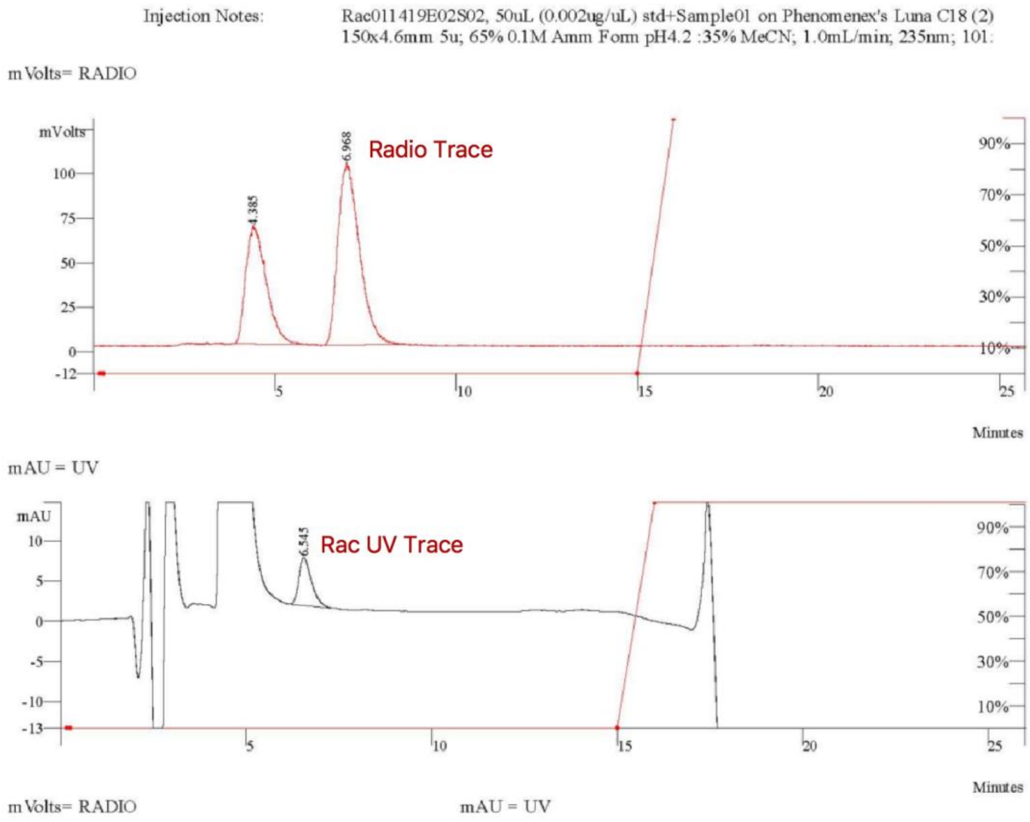

[1-^{11}C]Succinic acid ([^{11}C]SucAcid, [^{11}C]19) synthesis

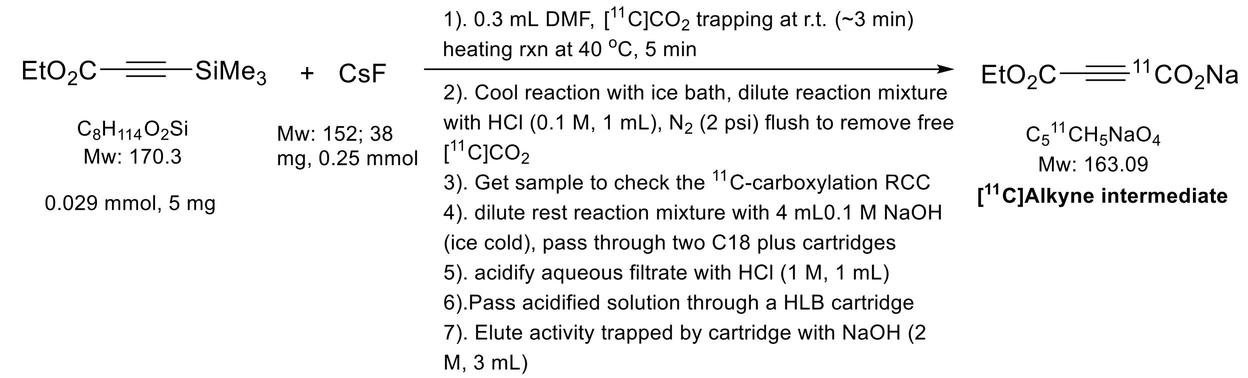

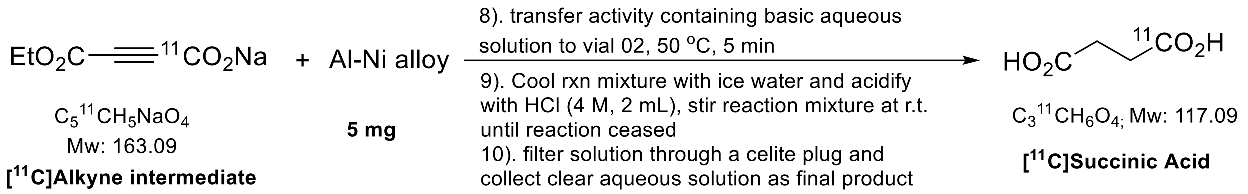

Following the procedures of pre-labeling work and ^{11}C-carboxylation reaction described above, [^{11}C]SucAcid (**[^{11}C]19**) was synthesized (see above scheme for experimental details) and the detailed results are listed in the table below:

Supplementary Table 59. Summarization of **[^{11}C]19** synthesis experimental results

Exp 01 Results	Exp 02 Results	Exp 03 Results	Avg ± SD Results
[^{11}C]CO_2: 4.45 GBq (120.2 mCi); Final Prod: 0.43 GBq (11.6 mCi); Synthesis time: 43 min; RCY: 37.4%; RCP: 90%	[^{11}C]CO_2: 3.81 GBq (103.1 mCi); Prod: 0.588 GBq (15.9 mCi); Synthesis time: 41 min; RCY: 62.0%; RCP: 99%	[^{11}C]CO_2: 5.88 GBq (159 mCi) Prod: 2.00 GBq (39.4 mCi) from SP-HPLC; Synthesis time: 30 min; RCY: 50.8%; RCP: 73.9%	Starting activity: 4.7 ± 1.1 GBq Product: 1.0 ± 0.9 GBq Synthesis time: 38.0 ± 7.0 min RCY: 50.1 ± 12.3% RCP: 87.6 ± 12.7%

Supplementary Figure 64. A [1-^{11}C]succinic acid, **[^{11}C]19**, final product sample analytical HPLC profile

HPLC analysis conditions: stationary phase: Phenomenex Luna(2) C18 150 × 4.6 mm; Mobile phase: phase A (0.1% formic acid), phase B (90% CH_3CN/10% H_2O); UV: 215 nm; Flow rate: 1.0 mL/min; Isocratic method: 1%B/99%A (0-12 min).

Description: SucAcid051519E02S01, 20 uL of mixture (270 uL 1.0 ug/uL std + 30 uL Sample), Phen Luna(2) C18 150*4.6 mm; Phase A: 0.1% formic acid; Phase B: 90%ACN, 10% H2O; 1%B, 12 min; 1.0mL/min; 215nm; 90.73bar

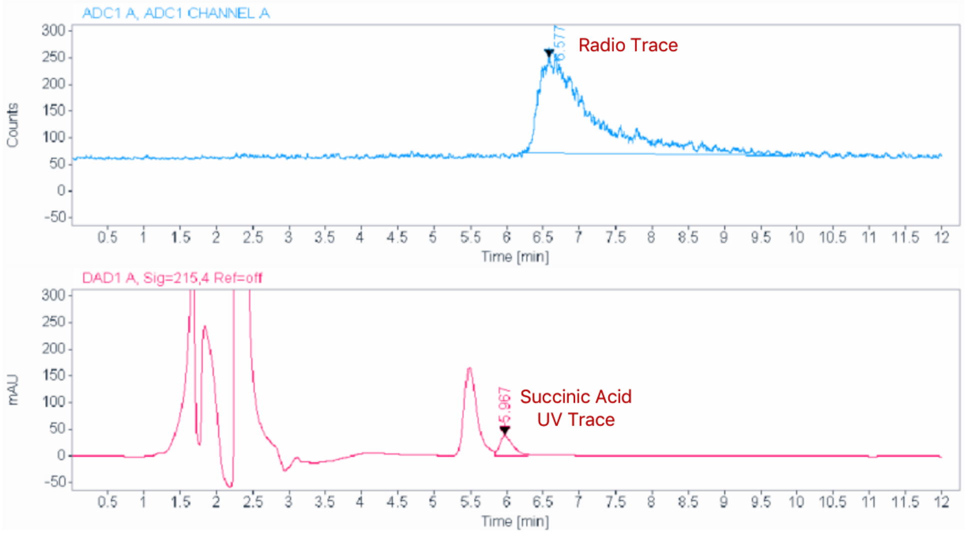

Supplementary Figure 65. A [^{12}C]succinic acid, **[^{12}C]19**, standard sample analytical HPLC profile

HPLC analysis conditions: stationary phase: Phenomenex Luna(2) C18 150 × 4.6 mm; Mobile phase: phase A (0.1% formic acid), phase B (90% CH_3CN/10% H_2O); UV: 215 nm; Flow rate: 1.0 mL/min; Isocratic method: 1%B/99%A (0-7.25 min).

Description: SucAcid051519std, 18 uL of 1.0 ug/uL std, Phen Luna(2) C18 150*4.6 mm; Phase A: 0.1% formic acid; Phase B: 90%ACN, 10% H2O; 1%B, 12 min; 1.0mL/min; 215nm; 90.73bar

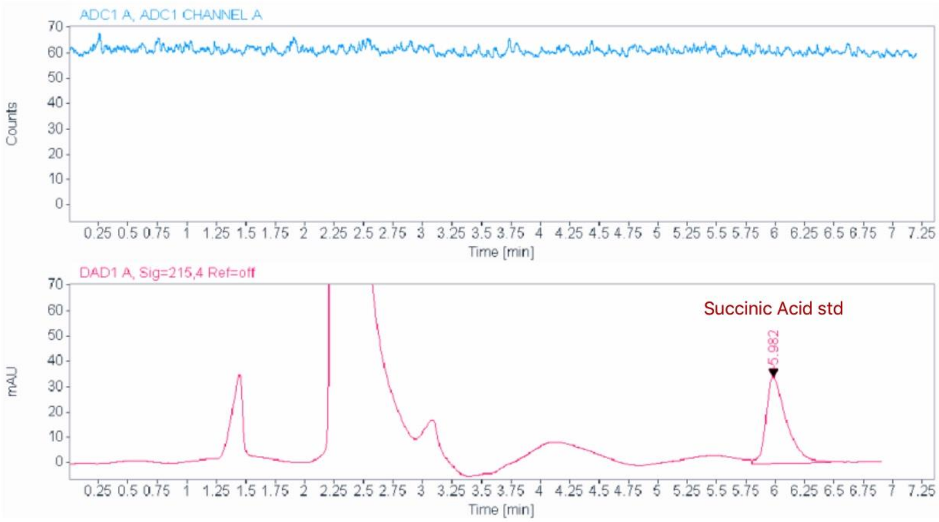

2.5. [1-^{11}C]Dichloroacetic acid ([^{11}C]DCAA, [^{11}C]13g) Synthesis

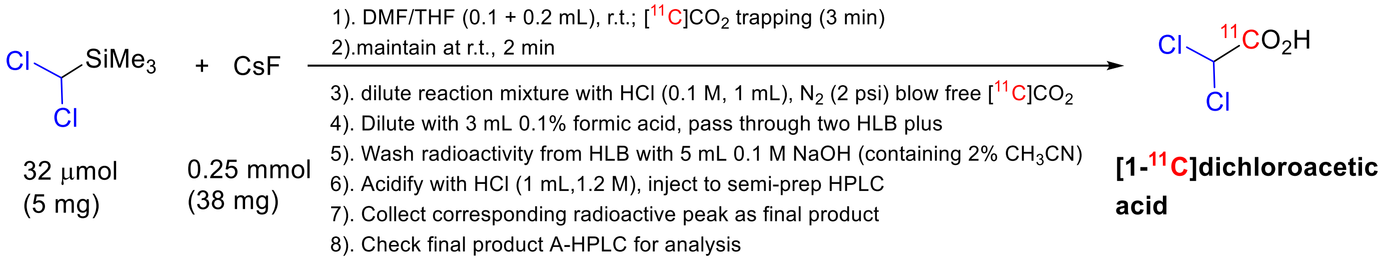

Following the procedures of pre-labeling work and ^{11}C-carboxylation reaction described above, [^{11}C]DCAA (**[^{11}C]13g**) as synthesized (see above scheme for experimental details) and the detailed results are listed in the table below:

Supplementary Table 60. Summarization of **[^{11}C]13g** synthesis experimental results

Exp 01 Results	Exp 02 Results	Exp 03 Results	Avg ± SD Results
[^{11}C]CO_2: 8.36 GBq (226.0 mCi);<br>Prod: 0.73 GBq (19.6 mCi) from SP-HPLC;<br>Synthesis time: 38 min;<br>RCY: 31.8%;<br>RCP: >99%<br>Total mass of ^{12}C-DCAA in final product: 4.73 µg<br>Molar activity (A_m) (GBq/µmol, EOB): 72.52<br>(mCi/µmol, EOB): 1960	[^{11}C]CO_2: 10.47 GBq (283.0 mCi);<br>Prod: 1.22 GBq (32.9 mCi) from SP-HPLC;<br>Synthesis time: 32 min;<br>RCY: 34.8%;<br>RCP: >99%<br>Total mass of ^{12}C-DCAA in final product: 8.82 µg<br>Molar activity (A_m) (GBq/µmol, EOB): 53.28<br>(mCi/µmol, EOB): 1440	[^{11}C]CO_2: 16.47 GBq (445.0 mCi)<br>Prod: 1.35 GBq (36.4 mCi) from SP-HPLC;<br>Synthesis time: 37 min;<br>RCY: 28.7%;<br>RCP: >99%<br>Total mass of ^{12}C-DCAA in final product: 6.82 µg<br>Molar activity (A_m) (GBq/µmol, EOB): 89.39<br>(mCi/µmol, EOB): 2416	Starting activity: 11.8 ± 4.2 GBq<br>Product: 1.1 ± 0.3 GBq<br>Synthesis time: 35.7 ± 3.2 min<br>RCY: 31.8 ± 3.1%<br>RCP: >99 ± 0%<br>Total mass of ^{12}C-DCAA in final product: 6.8 ± 2.0 µg<br>A_m (GBq/µmol, EOB): 71.7 ± 18.1<br>(mCi/µmol, EOB): 1939 ± 488.3

Supplementary Figure 66. A [^{11}C]DCAA, **[^{11}C]13g**, semi-prep HPLC profile

HPLC semi-Prep conditions: stationary phase: Phenomenex Luna C18 PFP(2) 250 × 10 mm; Mobile phase: 0.1% trifluoroacetic acid/CH_3CN (99/1, V/V); UV: 210 nm, flow rate: 4.0 mL/min; Isocratic method, 18 min.

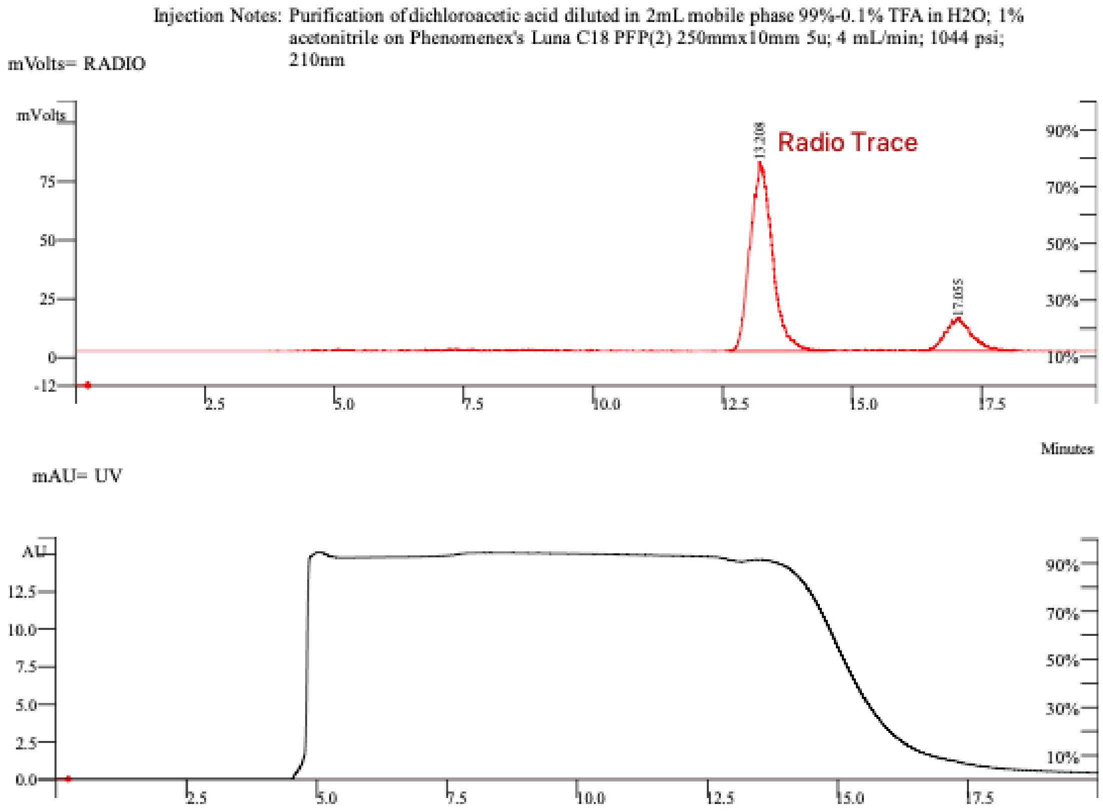

Supplementary Figure 67. A [^{12}C]DCAA, **[^{12}C]13g**, standard sample semi-prep HPLC profile

HPLC semi-Prep conditions: stationary phase: Phenomenex Luna C18 PFP(2) 250 × 10 mm; Mobile phase: 0.1% trifluoroacetic acid/CH_3CN (99/1, V/V); UV: 210 nm, flow rate: 4.0 mL/min; Isocratic method, 18 min.

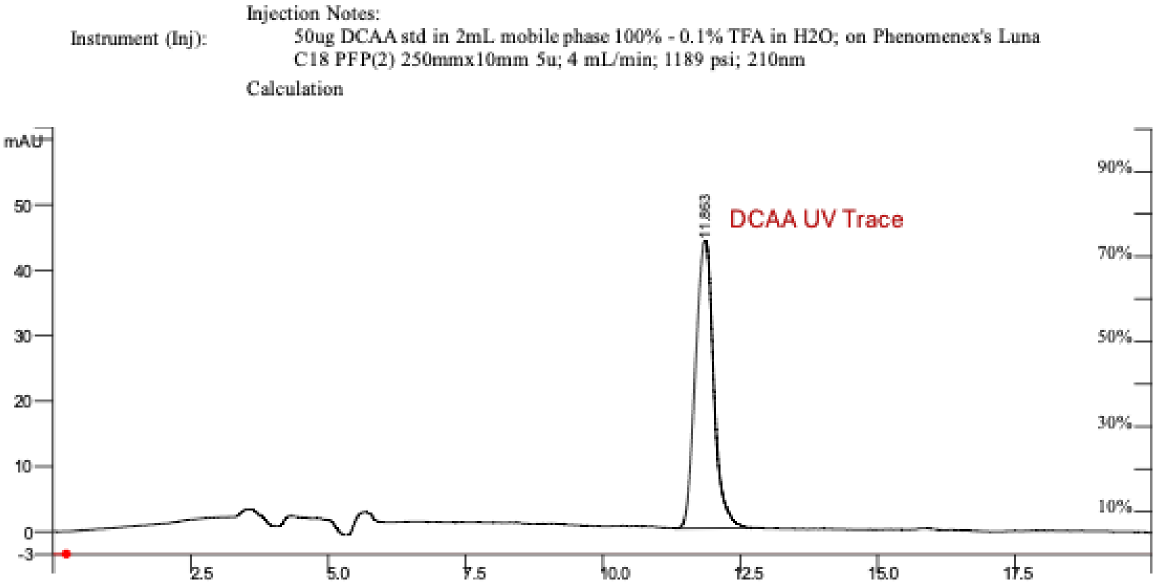

Supplementary Figure 68. A [^{11}C]DCAA, **[^{11}C]13g**, final product sample analytical HPLC profile

HPLC analysis conditions: stationary phase: Phenomenex Luna NH_2 150 × 4.6 mm; Mobile phase: phase A (40 mM NH_4CO_2H), phase B (90% CH_3CN/10% H_2O); UV: 210 nm; Flow rate: 1.5 mL/min; Isocratic method: 55%B/45%A (0-15 min).

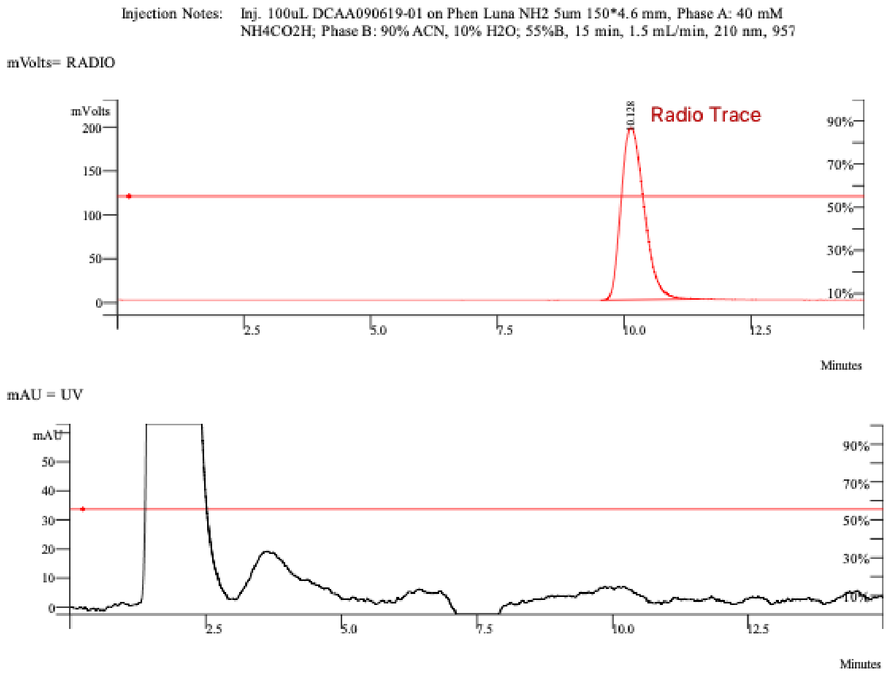

Supplementary Figure 69. A [^{11}C]DCAA, **[^{11}C]13g**, final product sample analytical HPLC profile (co-injection)

HPLC analysis conditions: stationary phase: Phenomenex Luna NH_2 150 × 4.6 mm; Mobile phase: phase A (40 mM NH_4CO_2H), phase B (90% CH_3CN/10% H_2O); UV: 210 nm; Flow rate: 1.5 mL/min; Isocratic method: 55%B/45%A (0-15 min).

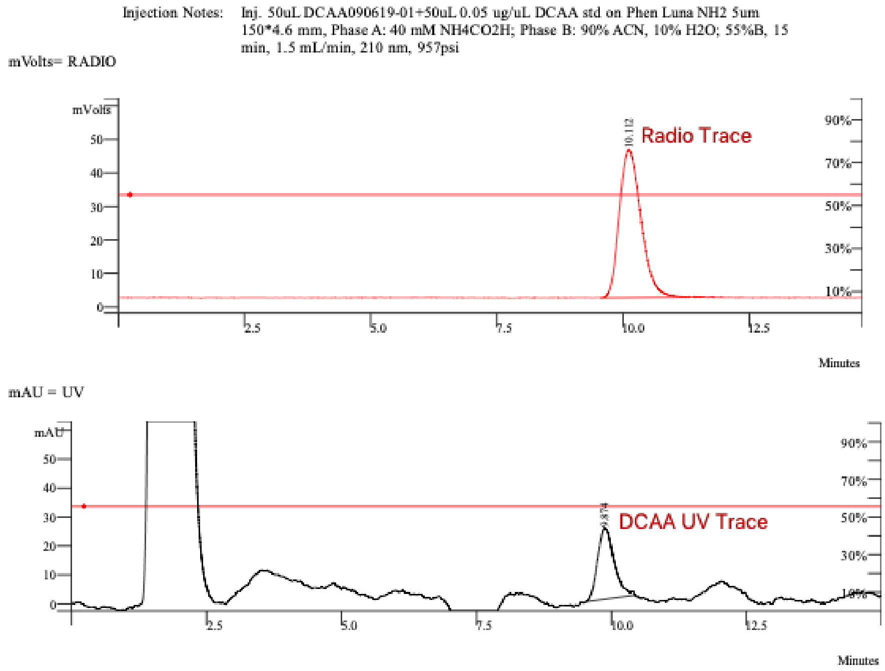

Supplementary Figure 70. A [^{12}C]DCAA, **[^{12}C]13g**, standard sample analytical HPLC profile

HPLC analysis conditions: stationary phase: Phenomenex Luna NH_2 150 × 4.6 mm; Mobile phase: phase A (40 mM NH_4CO_2H), phase B (90% CH_3CN/10% H_2O); UV: 210 nm; Flow rate: 1.5 mL/min; Isocratic method: 55%B/45%A (0-15 min).

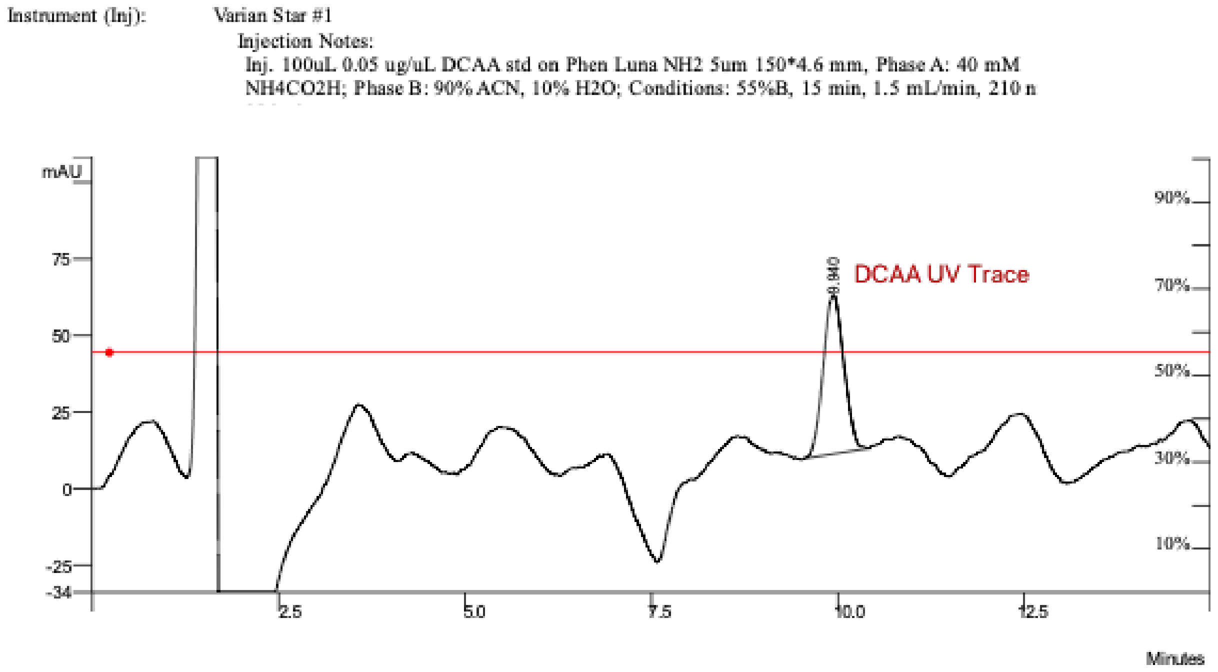

Measurement of molar activity (A_m) of [^{11}C]DCAA, [^{11}C]13g

Molar activity (A_m) was calculated based upon the mass amount of **[^{12}C]13g** in final product. The analytical HPLC analysis was performed with conditions: stationary phase, Phenomenex Luna NH_2 column, 150 x 4.6 mm, 5 µm; mobile phase, acetonitrile and ammonium formate aqueous solution (CH_3CN/NH_4CO_2H, 40 mM, 50/50, V/V); flow rate, 1.5 mL/min; UV @ 210 nm.

Since the amount of **[^{12}C]13g** in final product solution (100 µL injection per sample) couldn't be determined under the above conditions (Figure S67), a higher concentration of the analyte was required. Thus, the decayed product fraction (1.5 mL) was basified with NaOH (0.1 M, 300 µL), and was concentrated by lyophilization. The white solid residue was re-dissolved with HCl (0.6 M, 150 µL). The obtained aqueous solution was analyzed by analytical HPLC (sample injected 100 µL) using the isocratic method described above. The UV absorbance was compared to a standard curve of UV absorbance versus mass of authentic **[^{12}C]13g**. The standard curve was generated by integration of the UV absorbance signal of three different known amounts of authentic **[^{12}C]13g** in duplicate (**Figure S71**).

Supplementary Figure 71: Standard curve of the UV absorbance vs amount of the authentic **[^{12}C]13g**

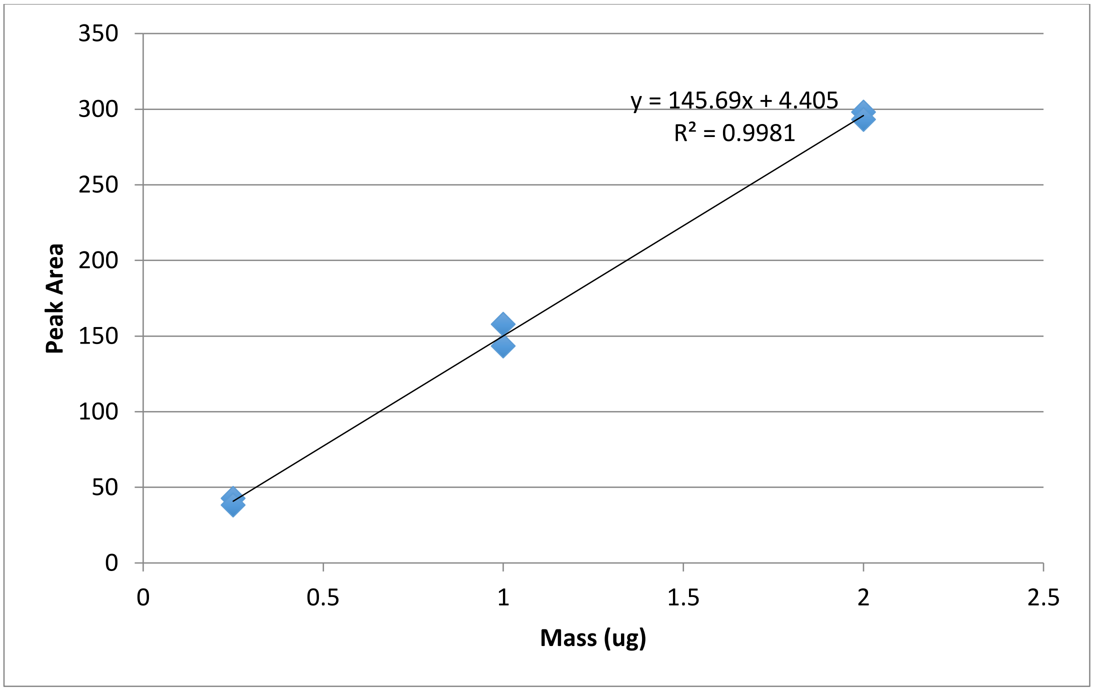

Supplementary Figure 72: ^{1}H NMR of 3-(2-methoxyphenyl)-2-propynoic acid (^{12}C standard **7e**)

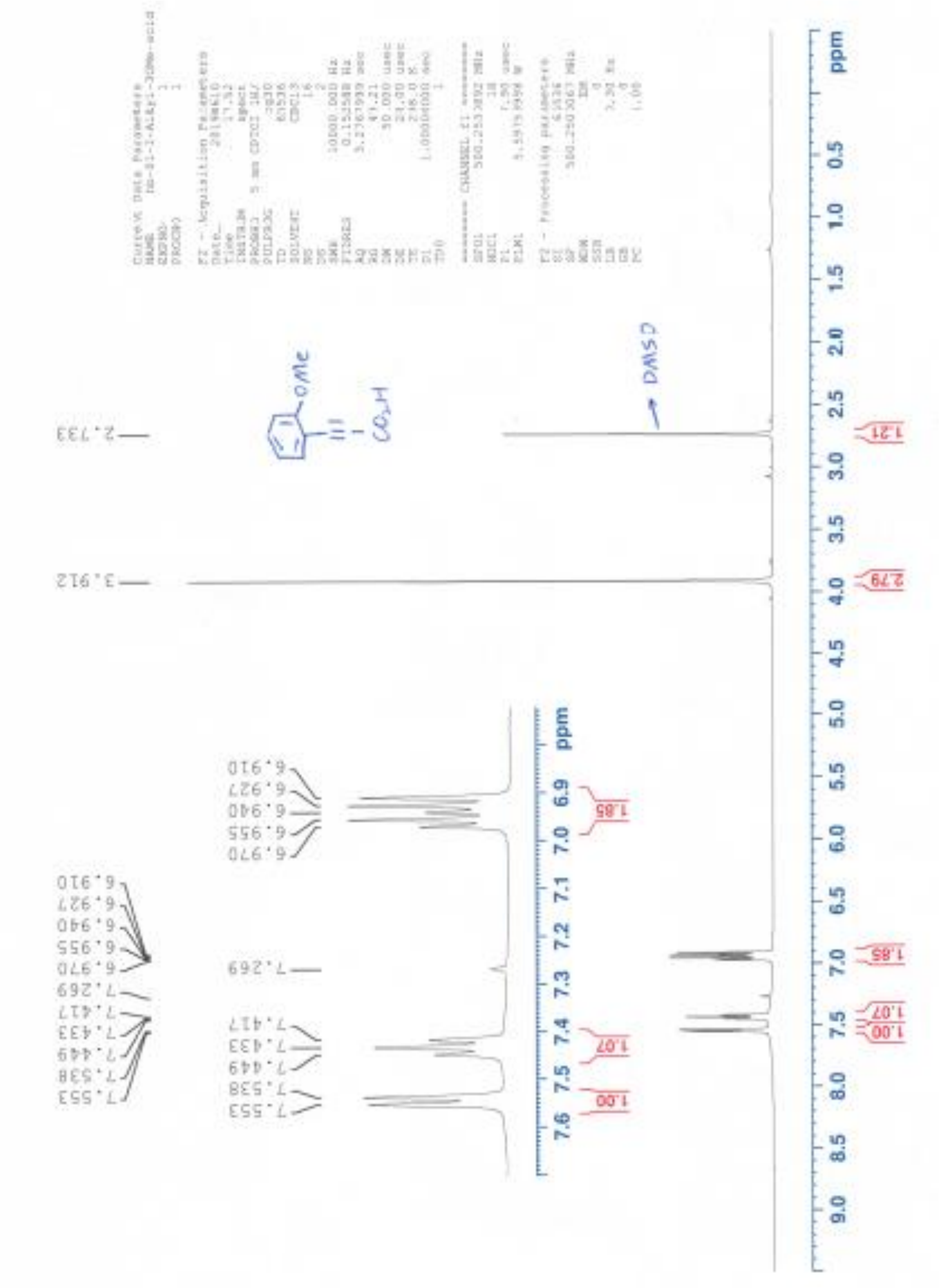

Supplementary Figure 73: ^{13}C NMR of 3-(2-methoxyphenyl)-2-propynoic acid (^{12}C standard **7e**)

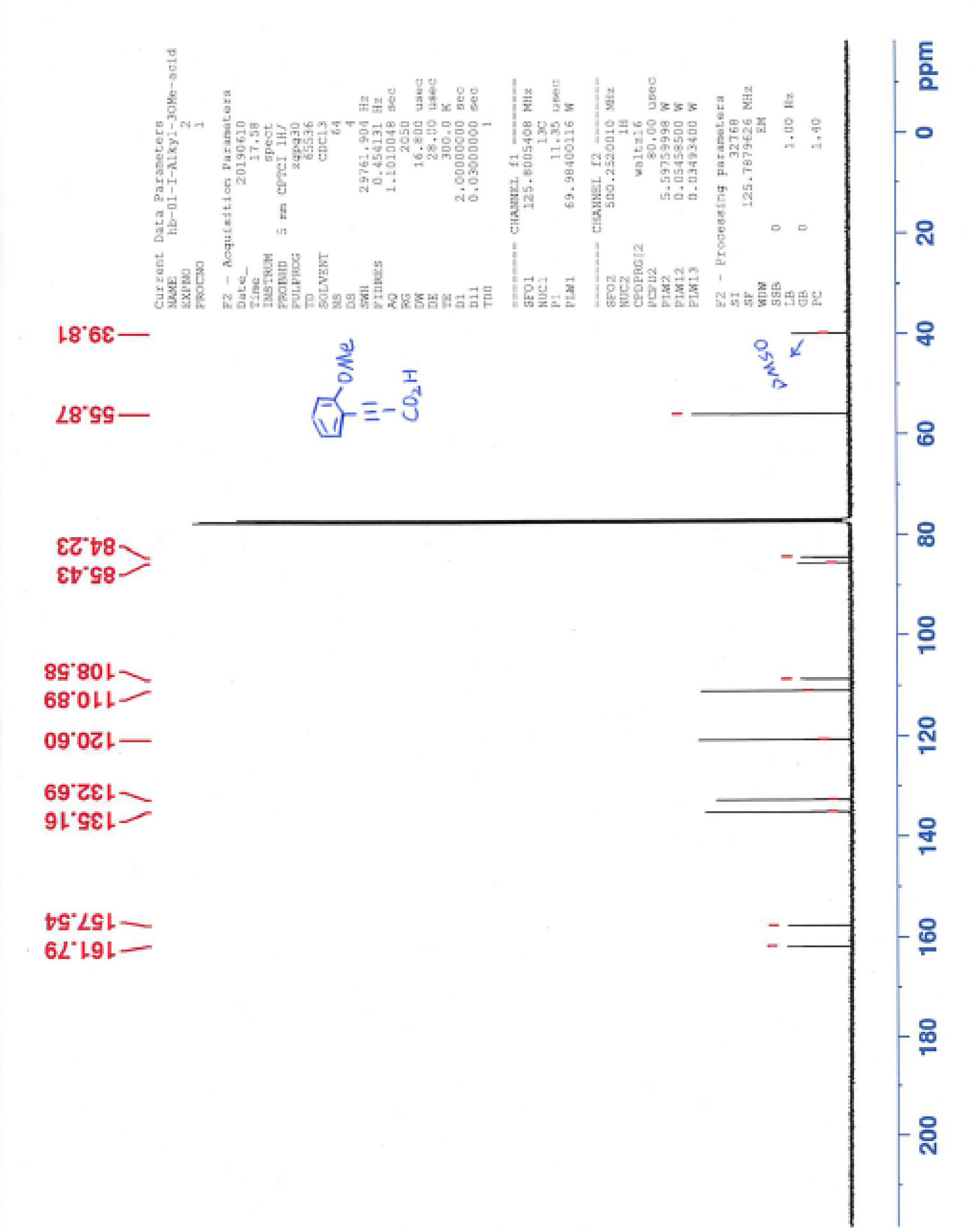

Supplementary Figure 74: ^{1}H NMR NMR of 6-chlorohex-2-ynoic acid (^{12}C standard **7j**)

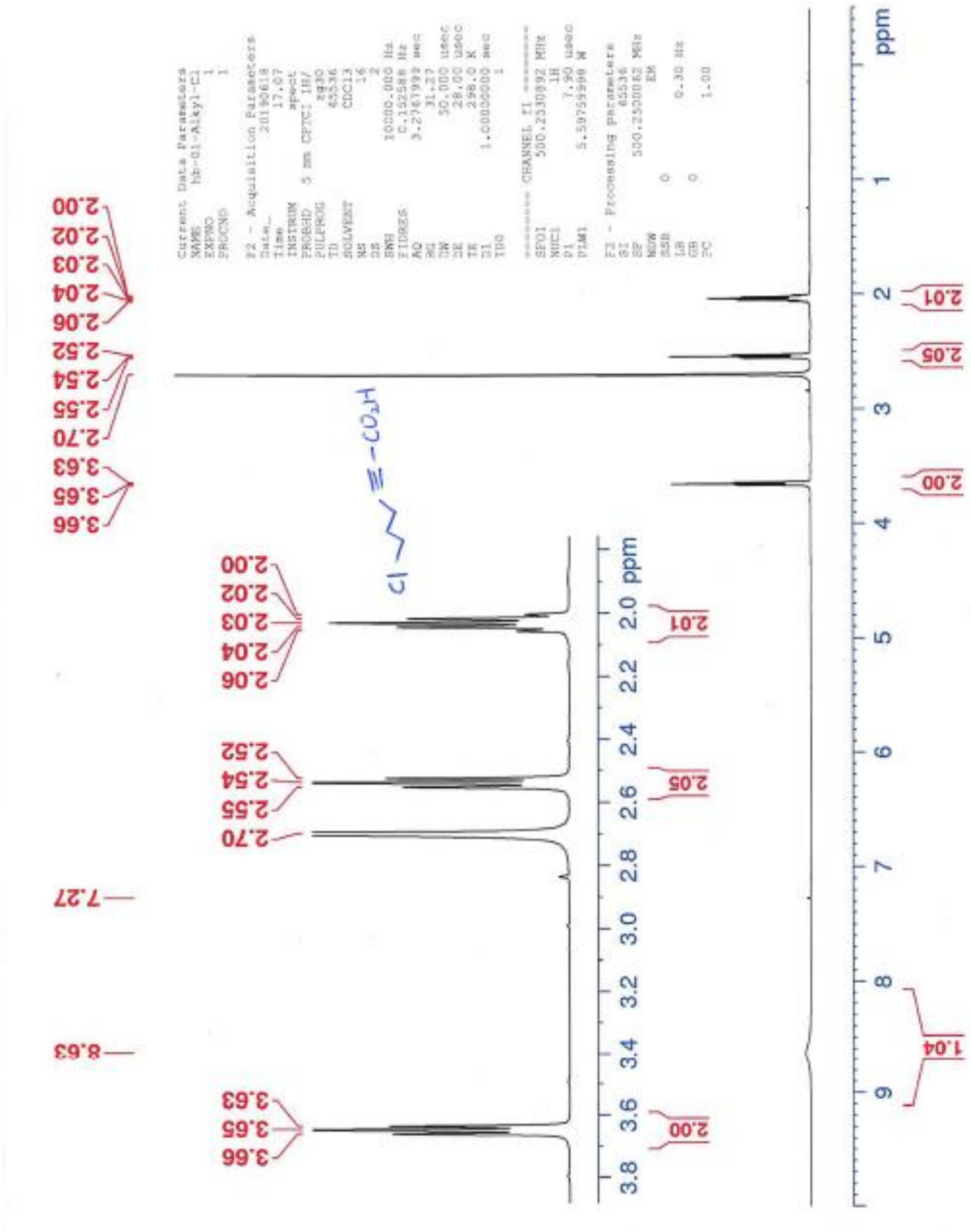

Supplementary Figure 75: ^{13}C NMR of 6-chlorohex-2-ynoic acid (^{12}C standard **7j**)

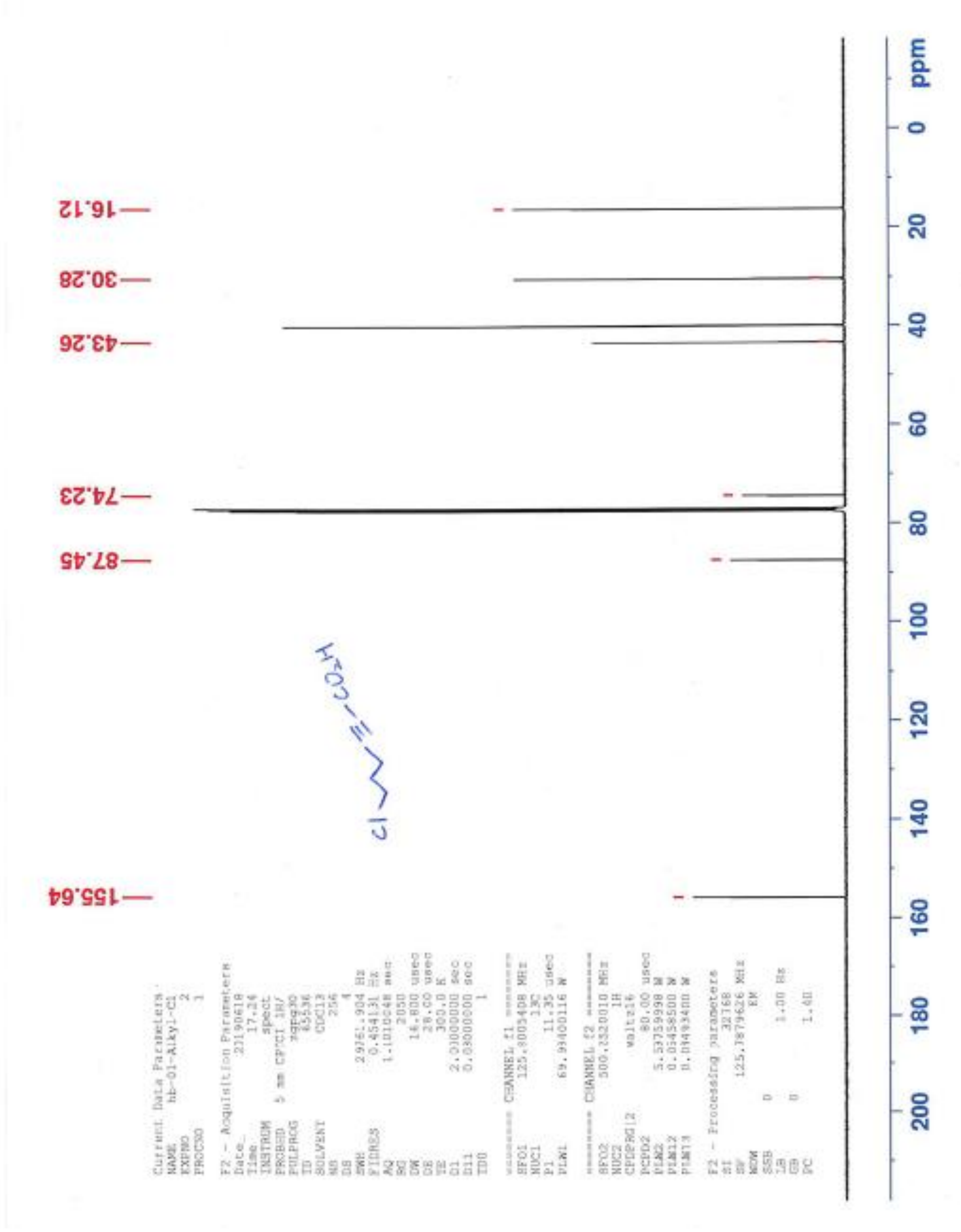

Supplementary Figure 76: ^{1}H NMR of 4-methoxy-4-oxobut-2-ynoic acid (^{12}C standard **7i**)

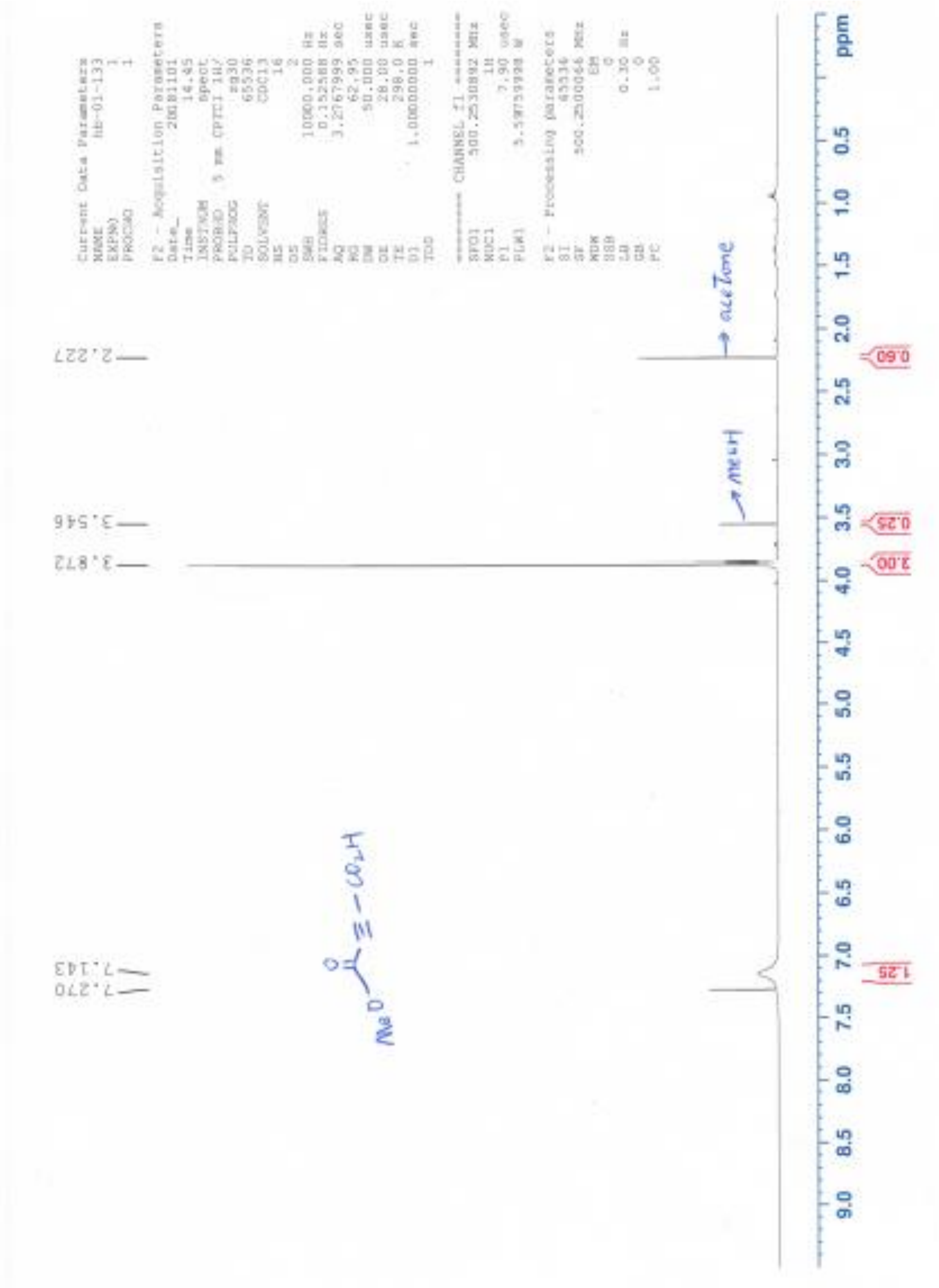

Supplementary Figure 77: ^{13}C NMR of 4-methoxy-4-oxobut-2-ynoic acid (^{12}C standard **7i**)

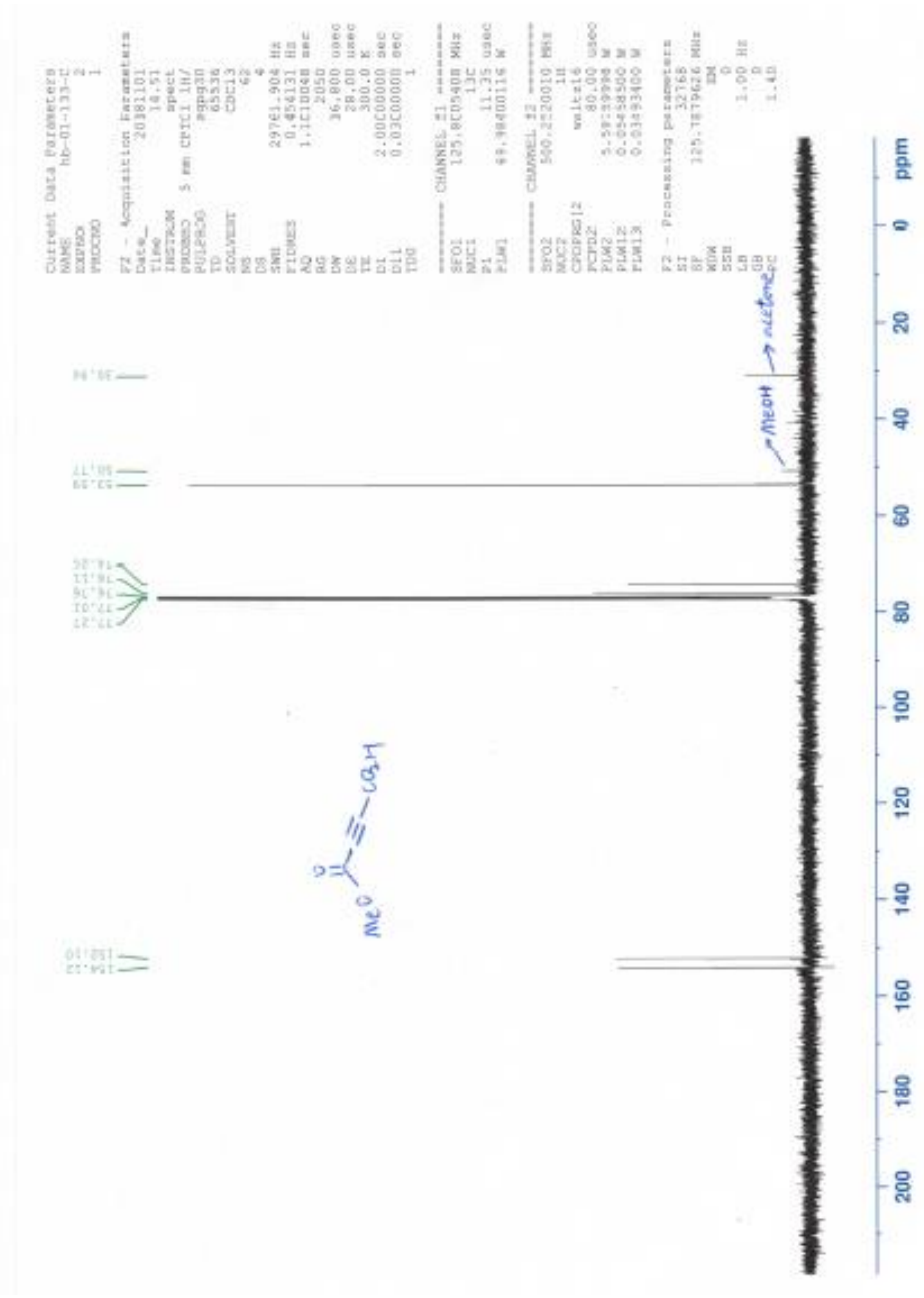

Supplementary Figure 78: ^{1}H NMR of 4-ethoxy-4-oxobut-2-ynoic acid (^{12}C standard **7k**)

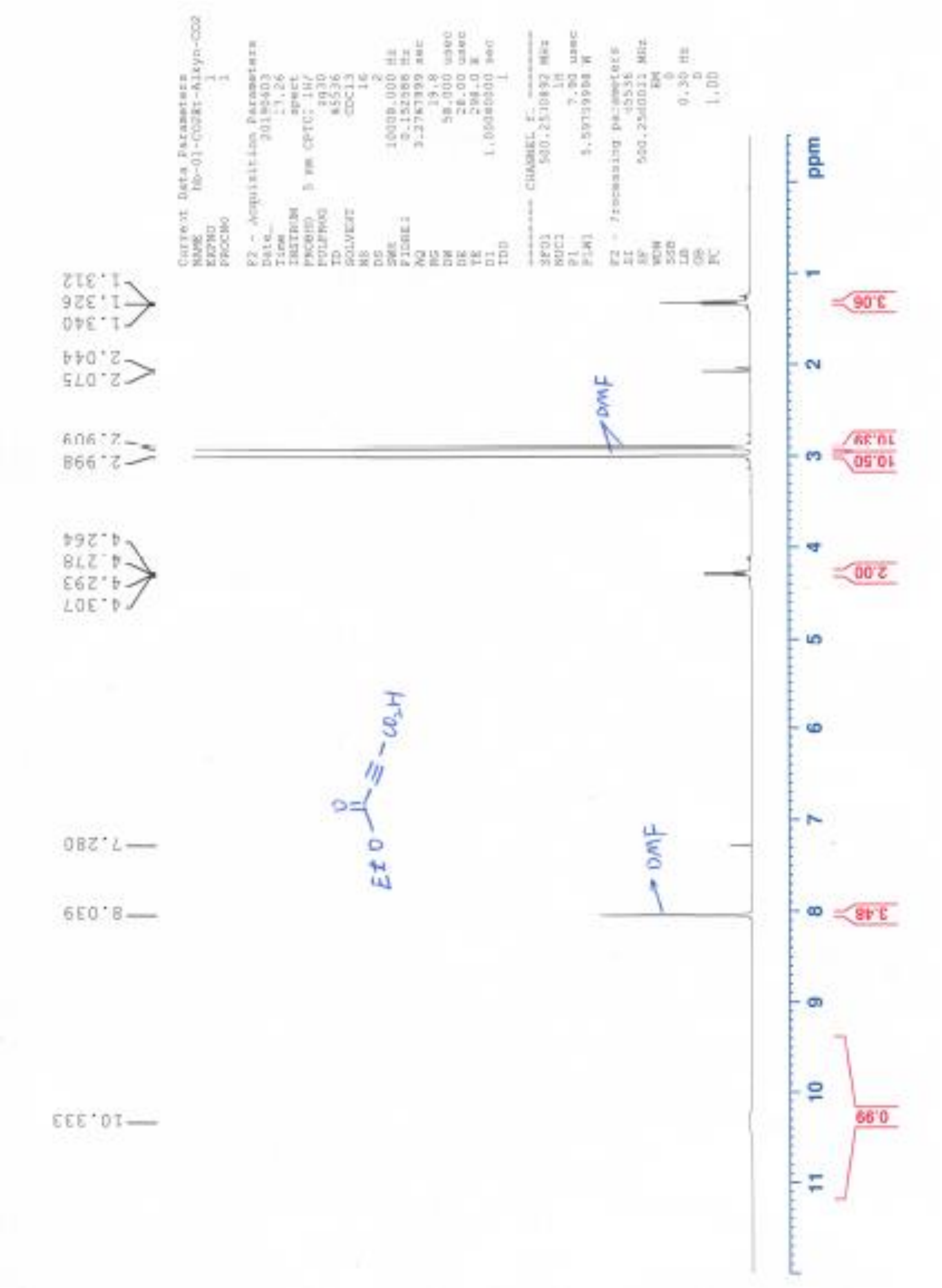

Supplementary Figure 79: ^{13}C NMR of 4-ethoxy-4-oxobut-2-ynoic acid (^{12}C standard **7k**)

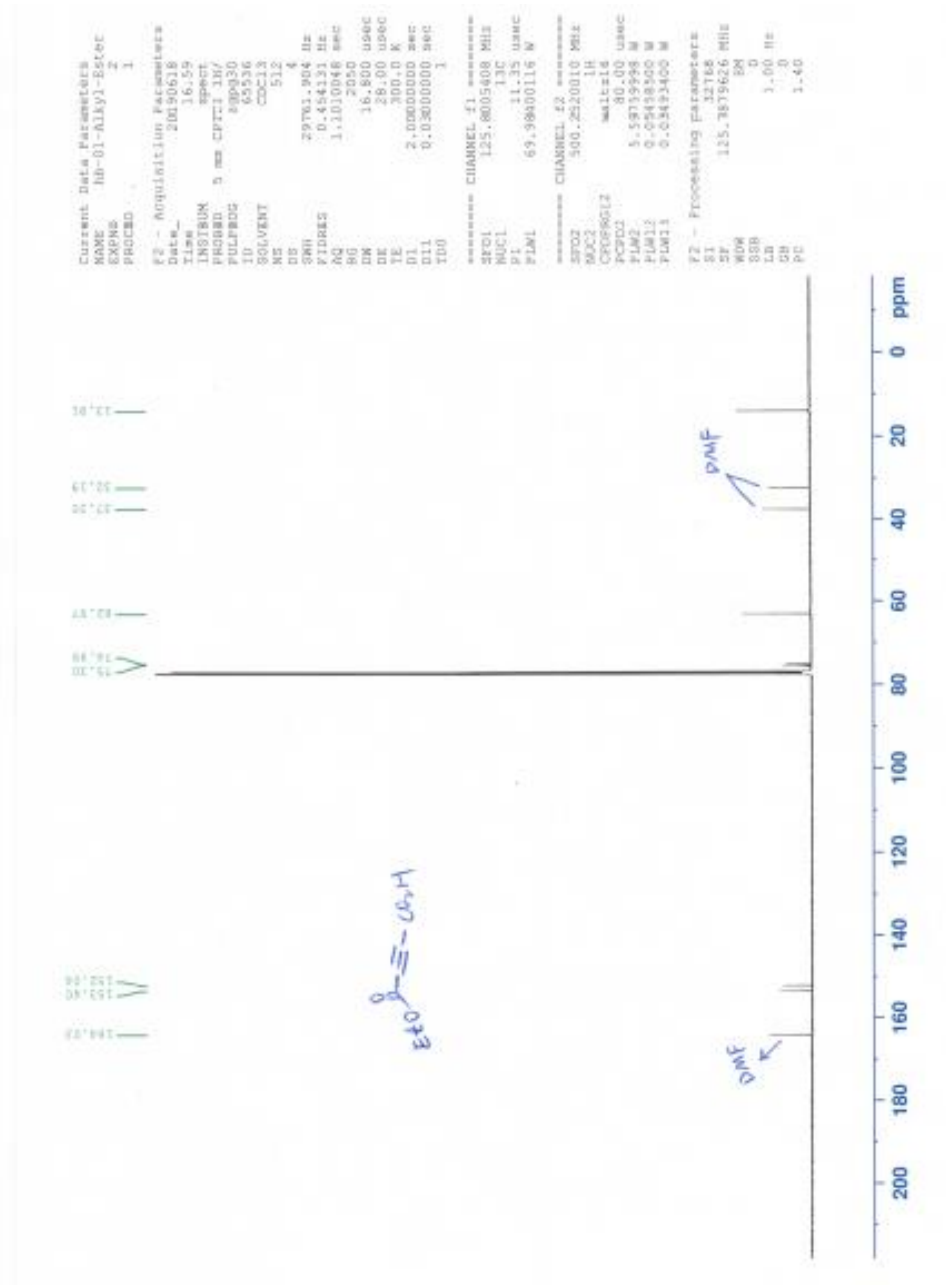

Supplementary Figure 80: ^{1}H NMR of *N*-benzyl-3-(3-chlorophenyl)propiolamide (^{12}C standard **9**)

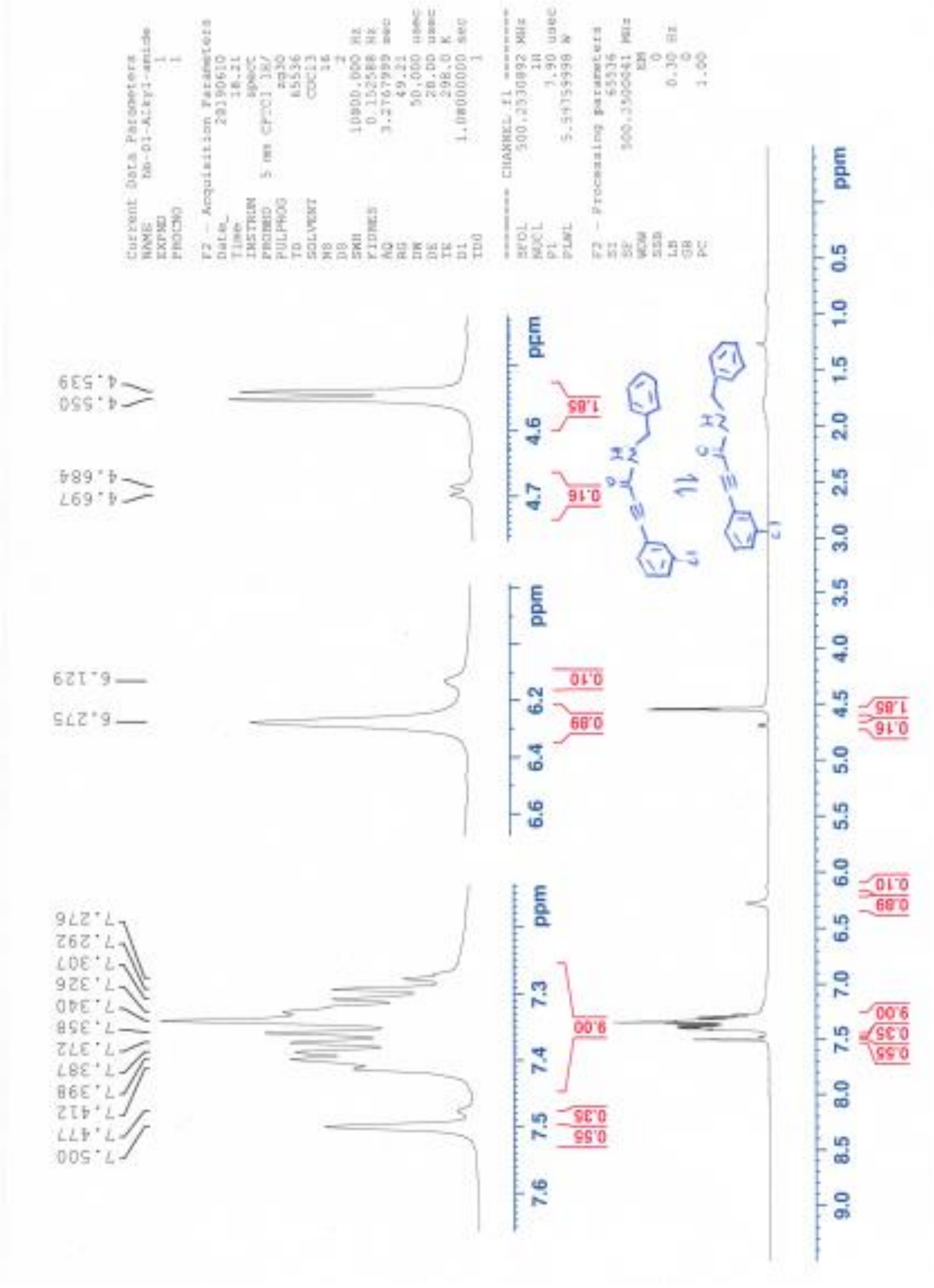

Supplementary Figure 81: ^{13}C NMR of *N*-benzyl-3-(3-chlorophenyl)propiolamide (^{12}C standard **9**)

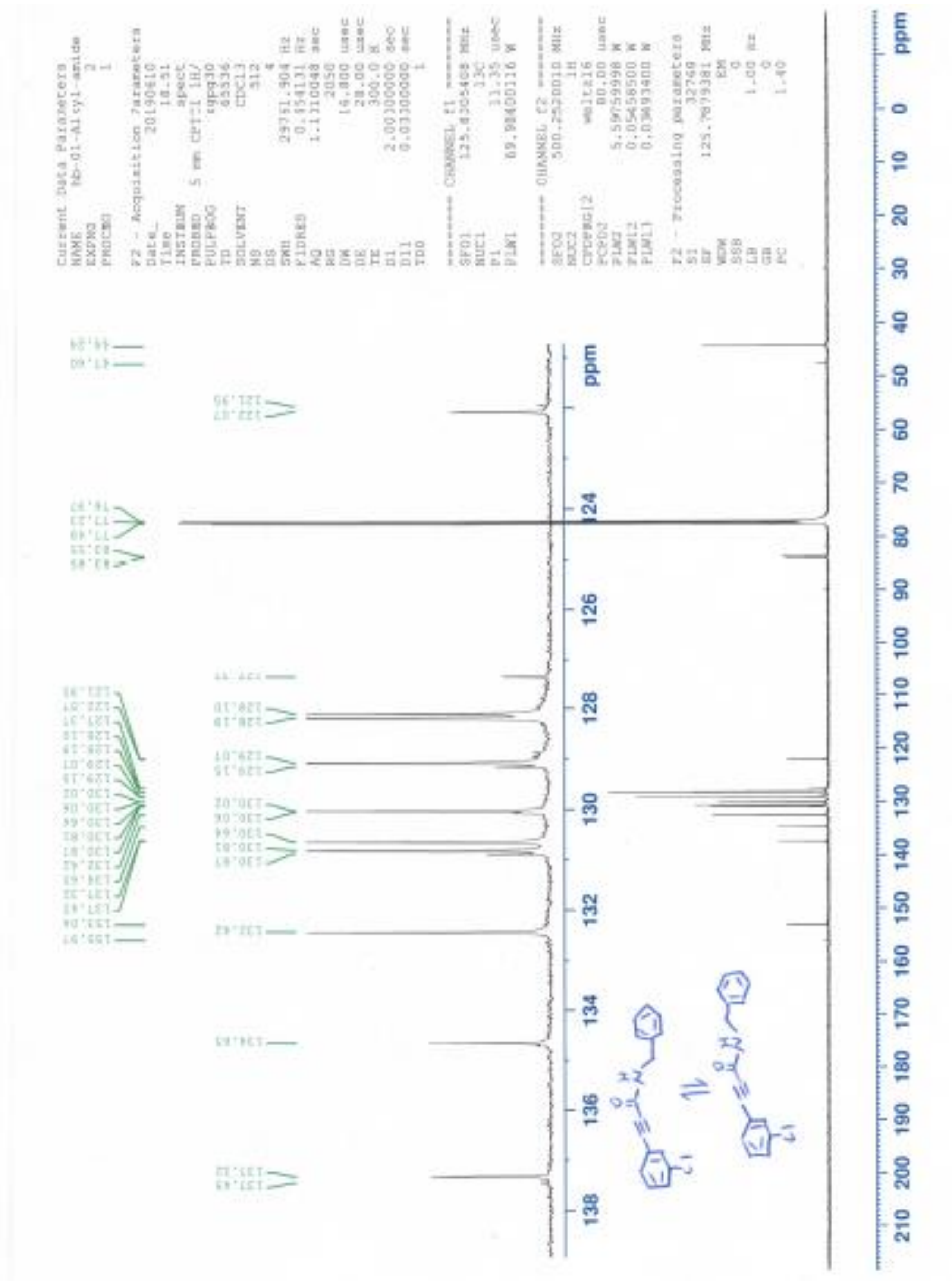

Supplementary Figure 82: ^{1}H NMR of (4-bromophenoxy)(*tert*-butyl)dimethylsilane (**15b**)

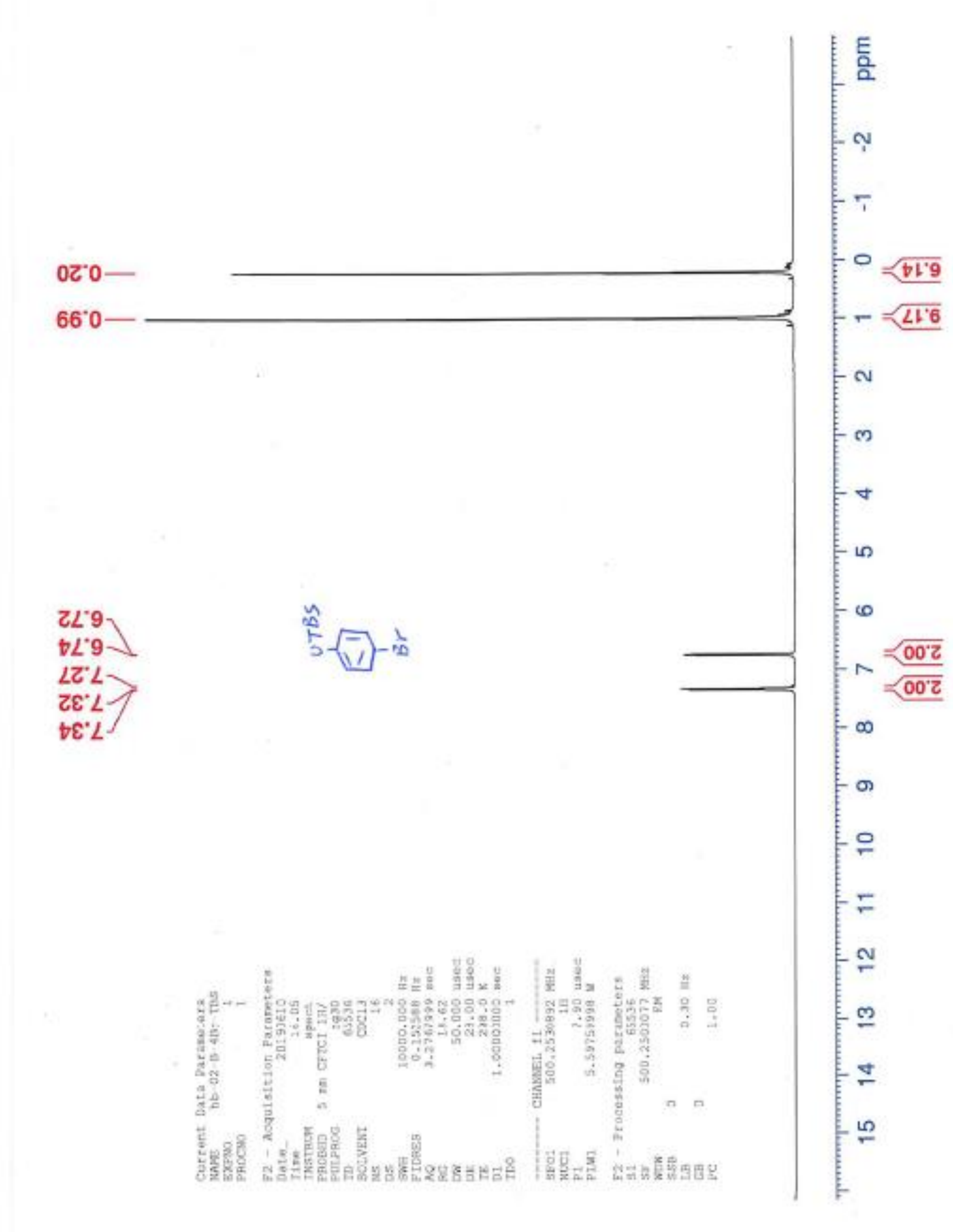

Supplementary Figure 83: ^{13}C NMR of (4-bromophenoxy)(*tert*-butyl)dimethylsilane (**15b**)

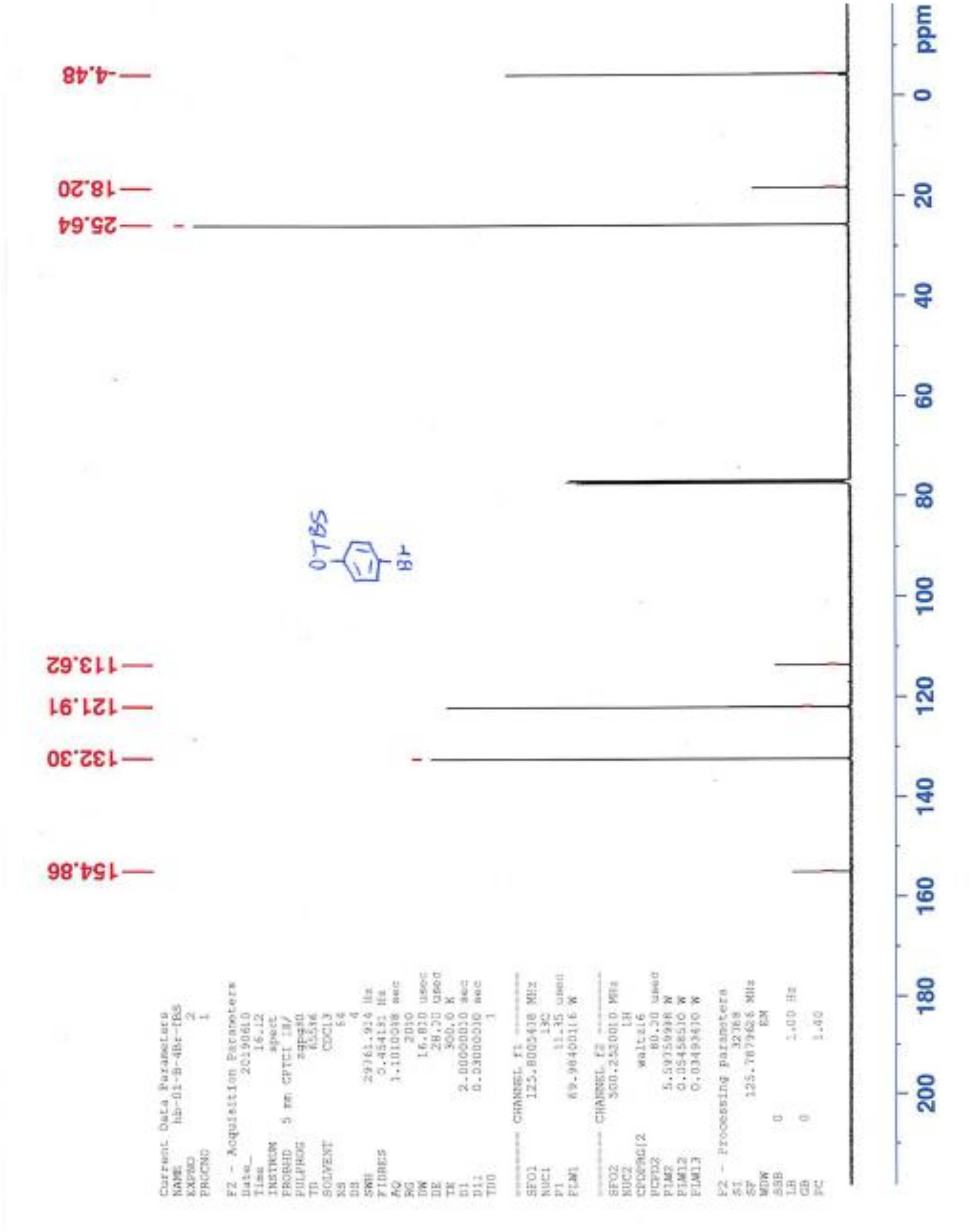

Supplementary Figure 84: ^{1}H NMR of (2-bromophenoxy)(*tert*-butyl)dimethylsilane (**15c**)

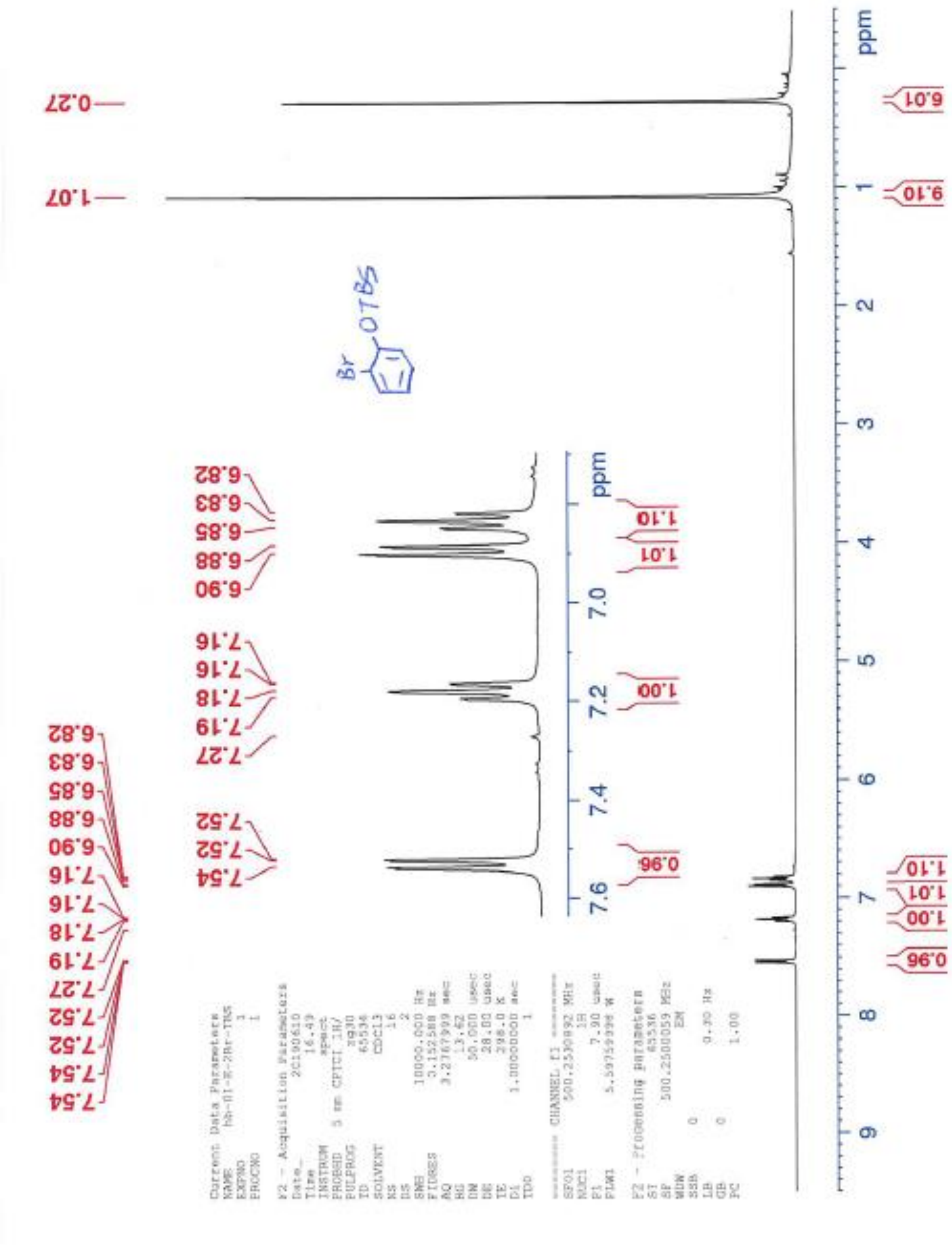

Supplementary Figure 85: ^{13}C NMR of (2-bromophenoxy)(*tert*-butyl)dimethylsilane (**15c**)

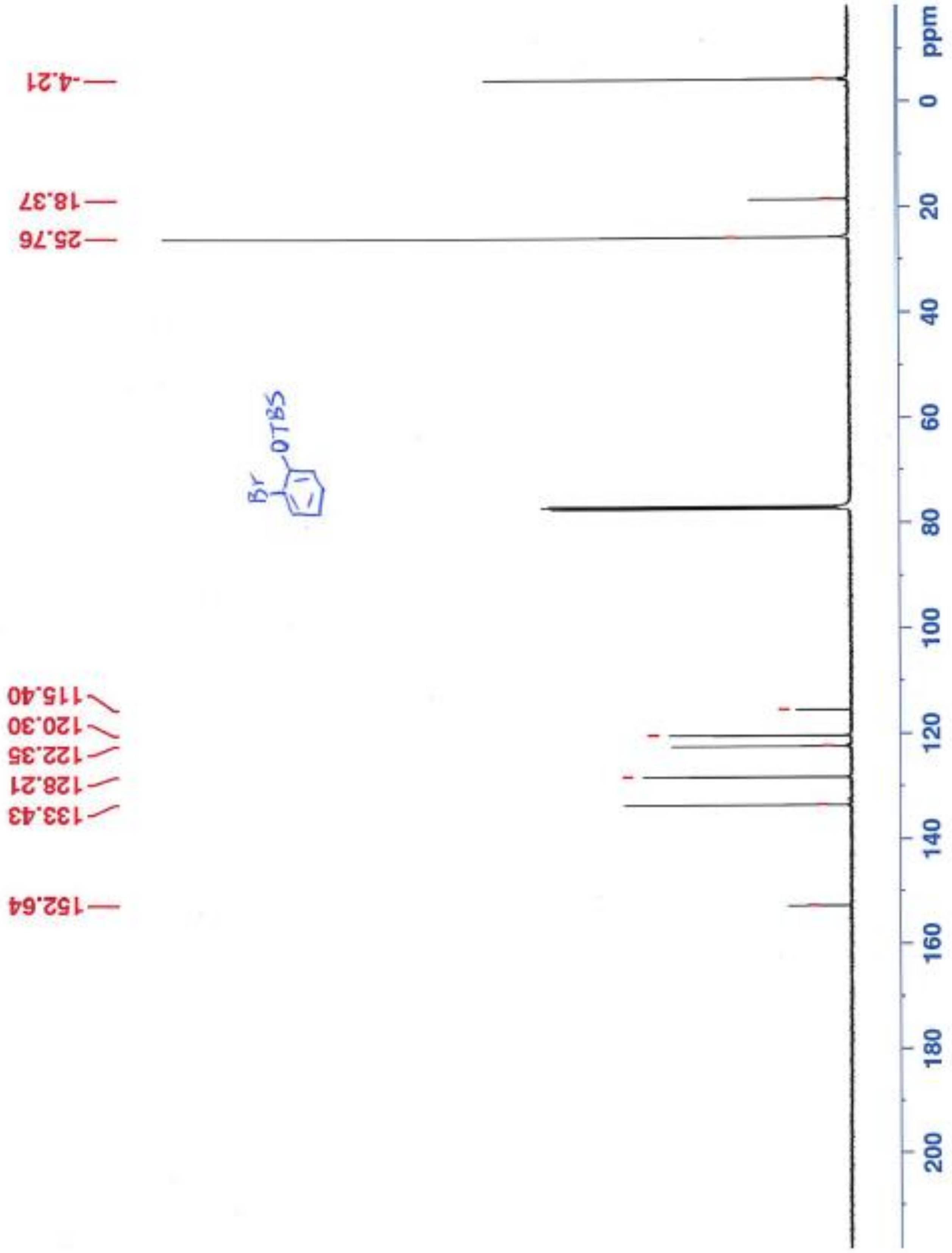

Supplementary Figure 86: ^{1}H NMR of *tert*-butyl(4-methoxyphenoxy)dimethylsilane (**15d**)

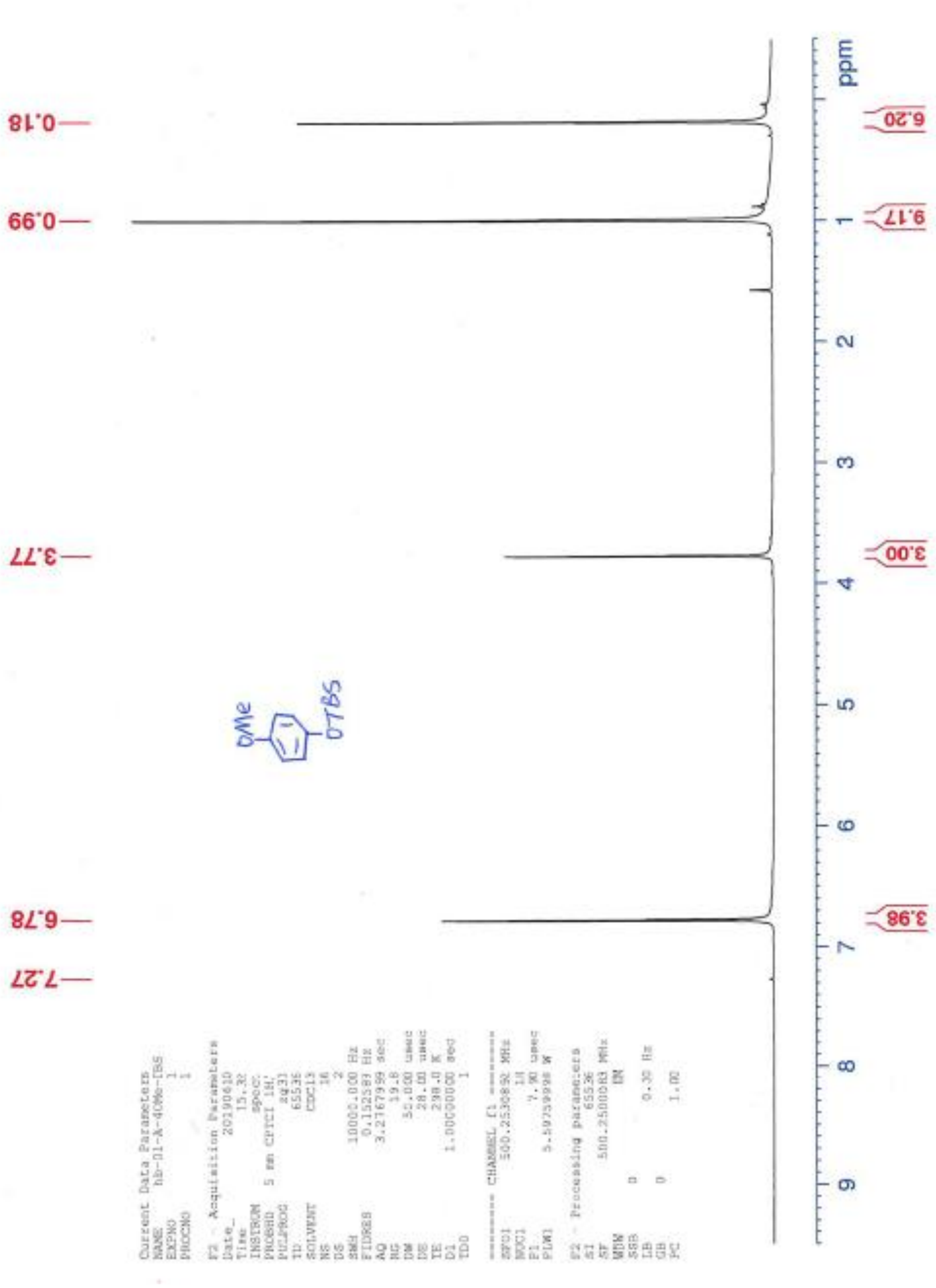

Supplementary Figure 87: ^{13}C NMR of *tert*-butyl(4-methoxyphenoxy)dimethylsilane (**15d**)

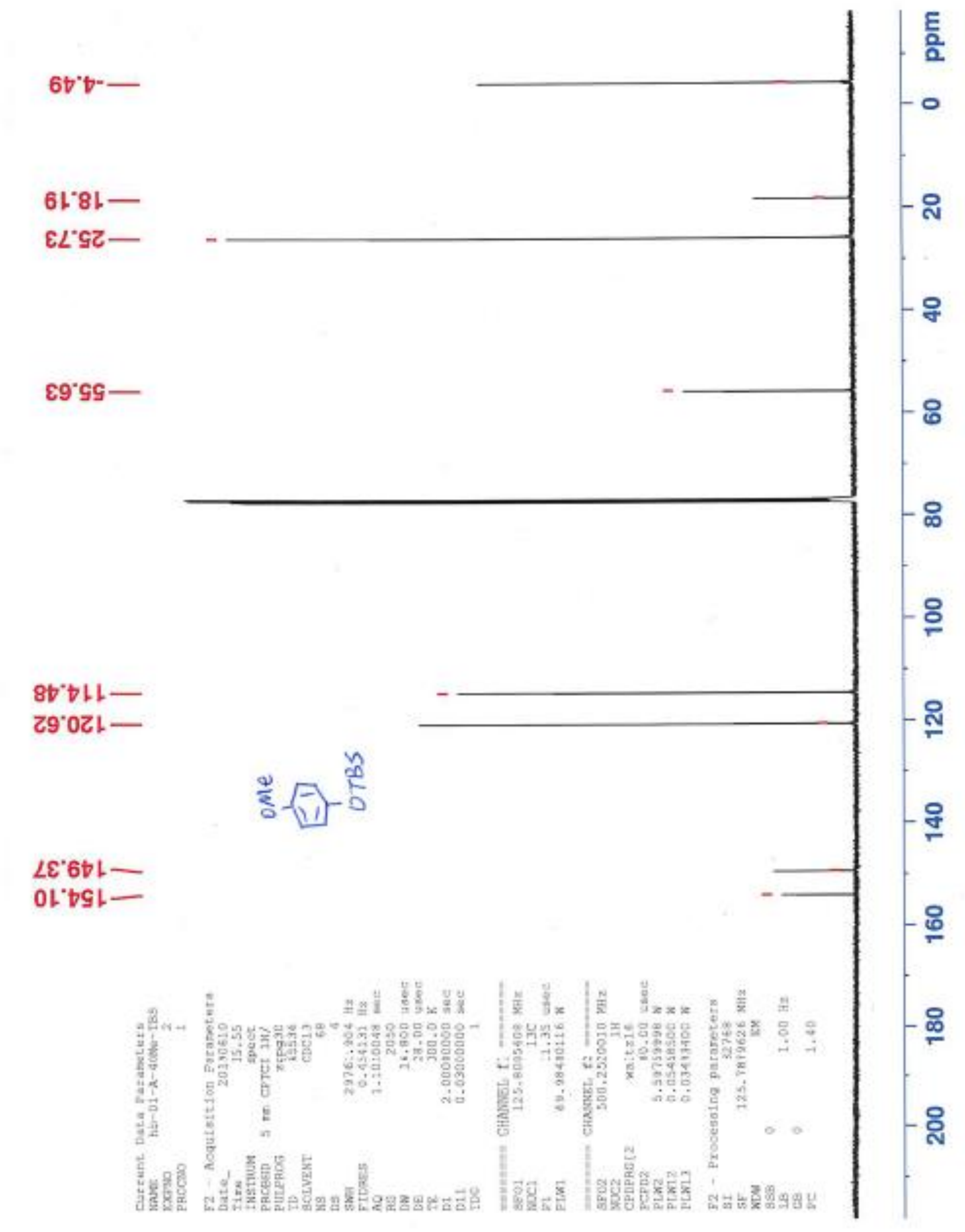

Supplementary Figure 88: ^{1}H NMR of *tert*-Butyl(3-methoxyphenoxy)dimethylsilane (**15e**)

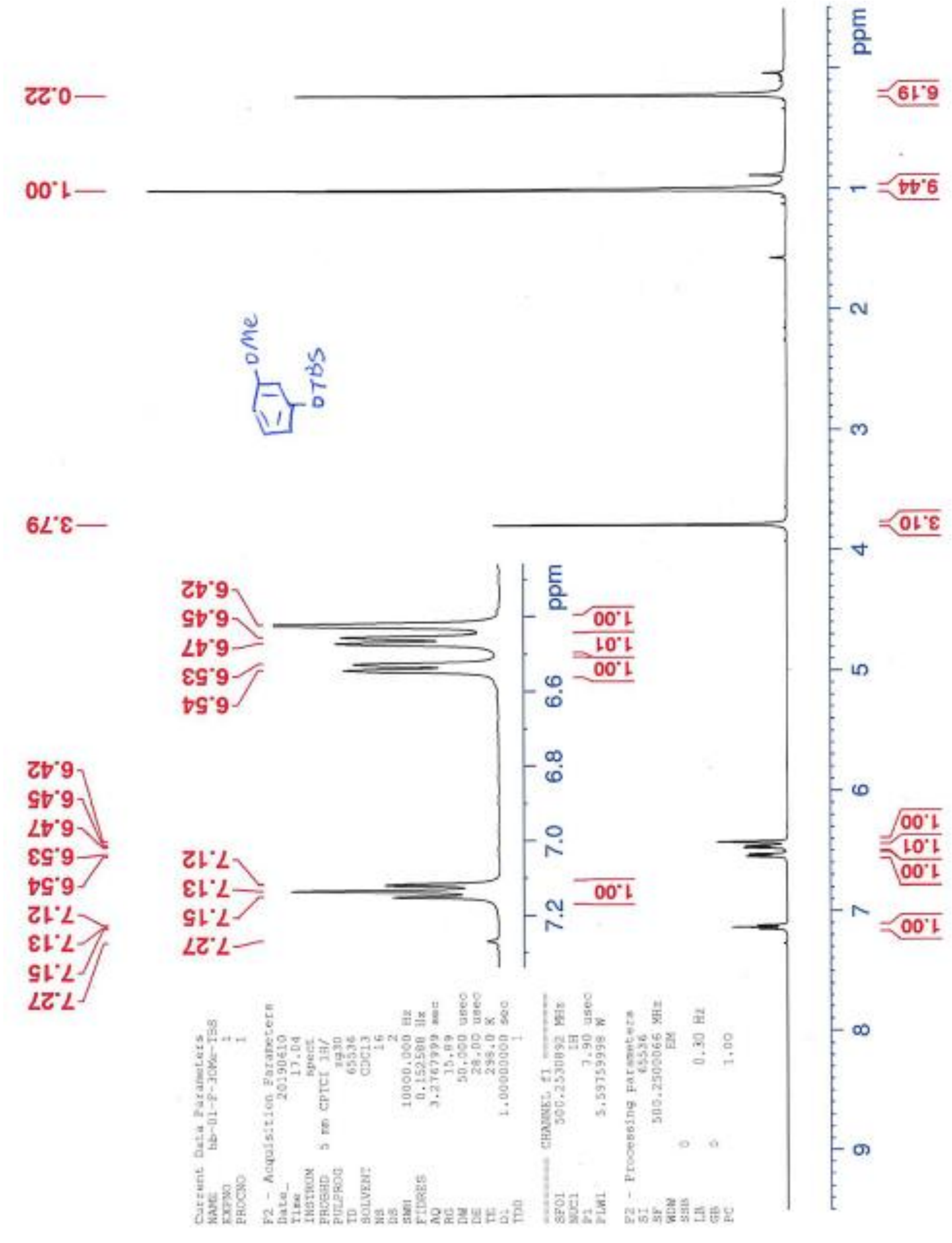

Supplementary Figure 89: ^{13}C NMR of *tert*-butyl(3-methoxyphenoxy)dimethylsilane (**15e**)

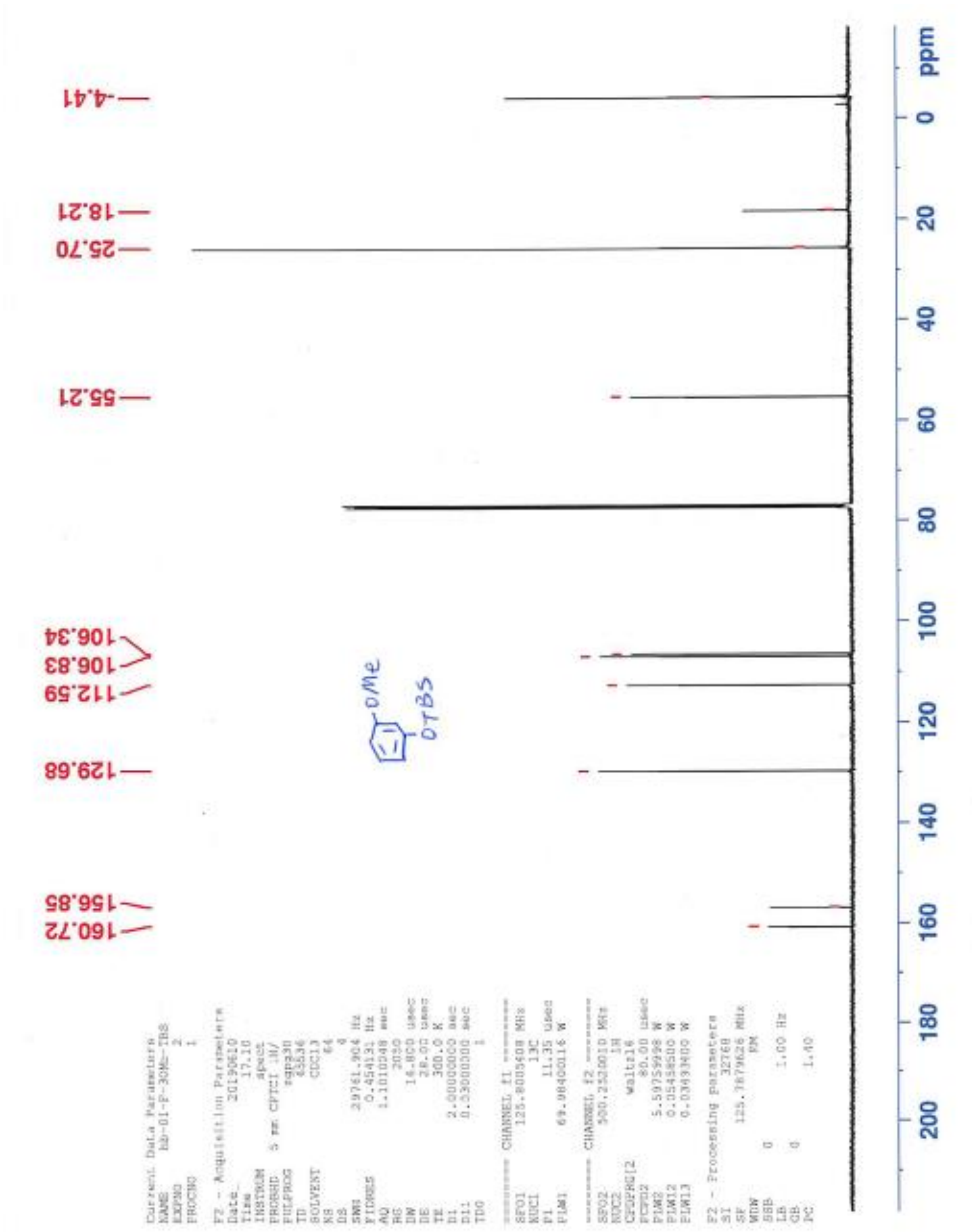

Supplementary Figure 90: ^{1}H NMR of methyl 4-((*tert*-butyldimethylsilyl)oxy)benzoate (**15f**)

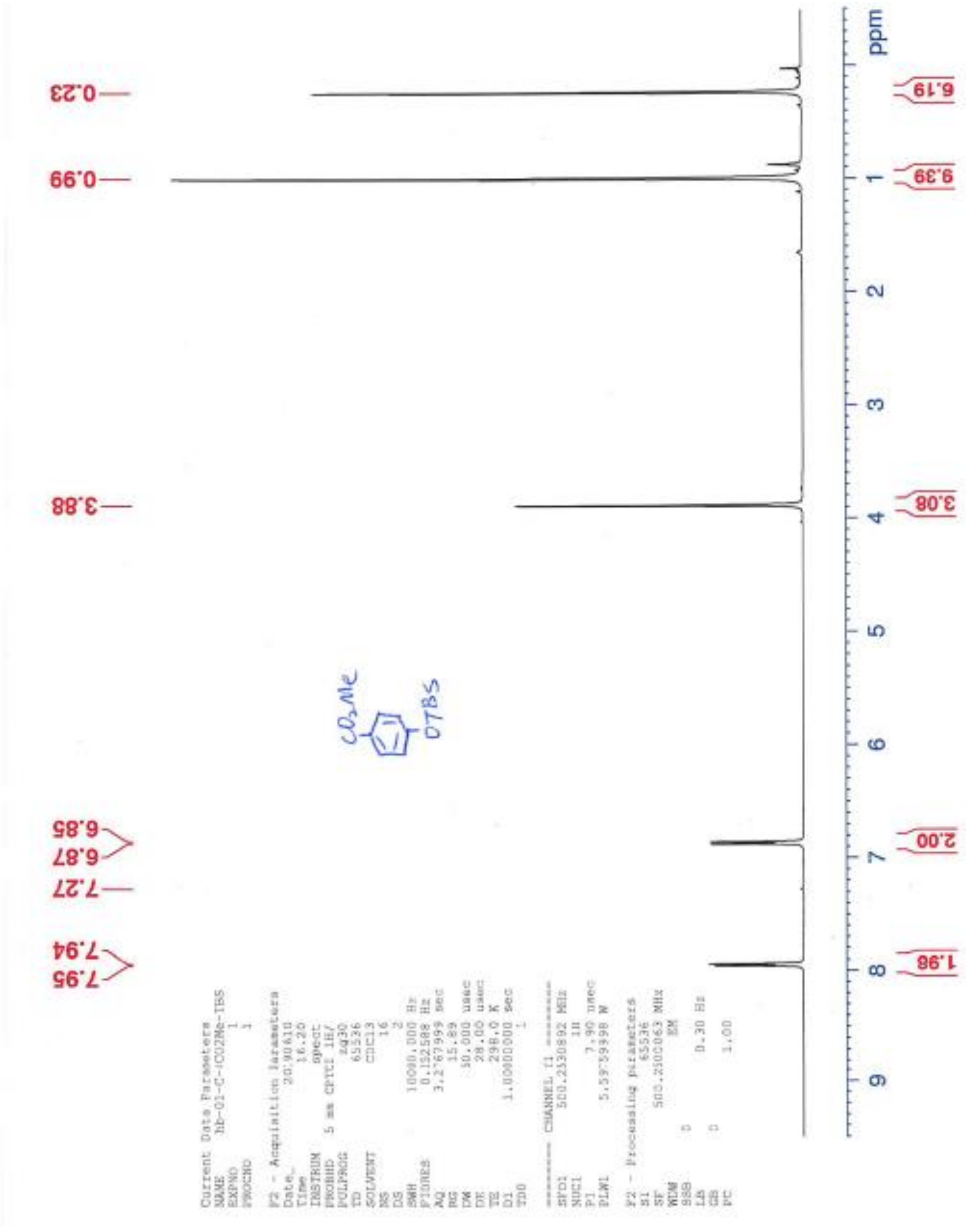

Supplementary Figure 91: ^{13}C NMR of methyl 4-((*tert*-butyldimethylsilyl)oxy)benzoate (**15f**)

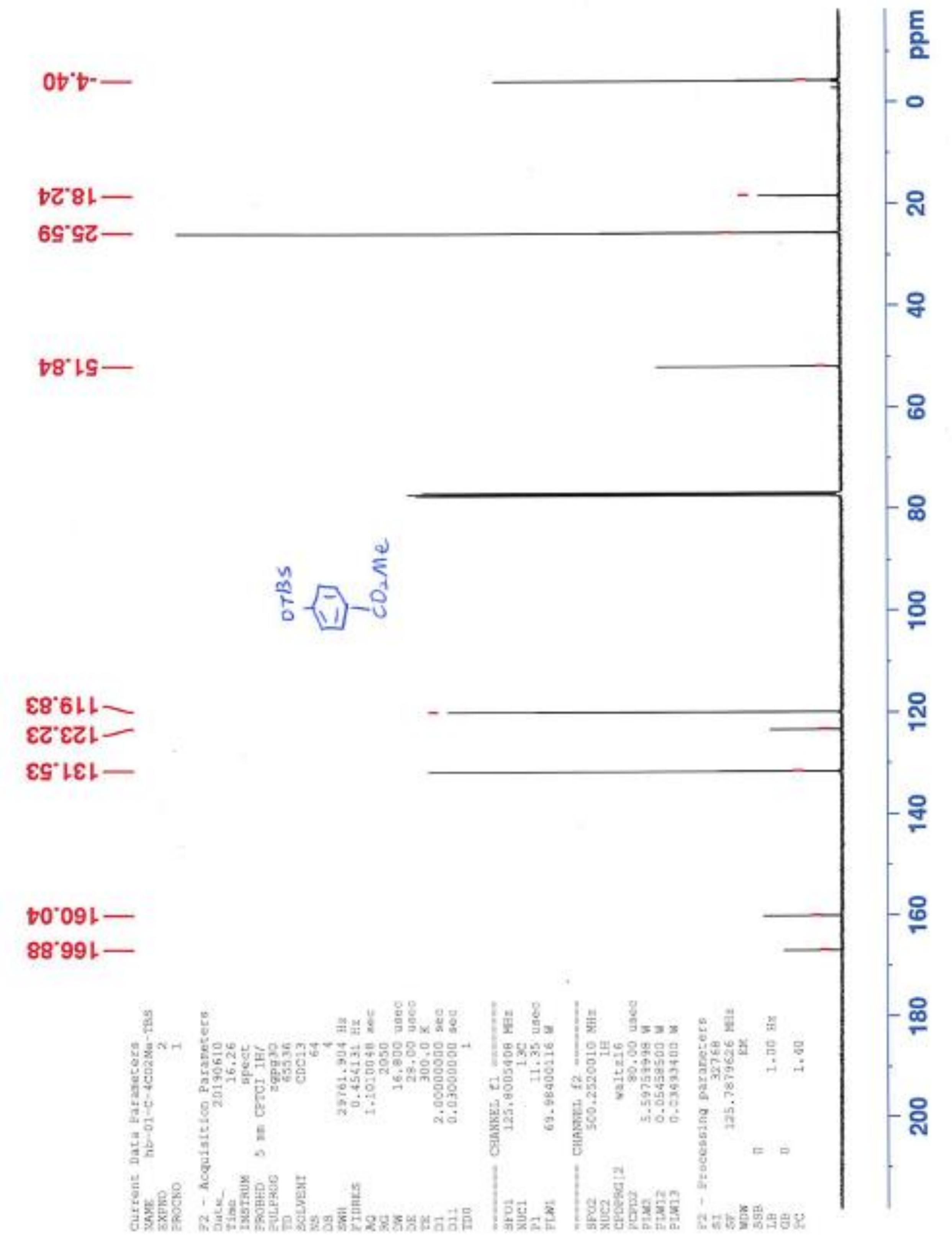

Supplementary Figure 92: ^{1}H NMR of *tert*-butyl(4-fluorophenoxy)dimethylsilane (**15g**)

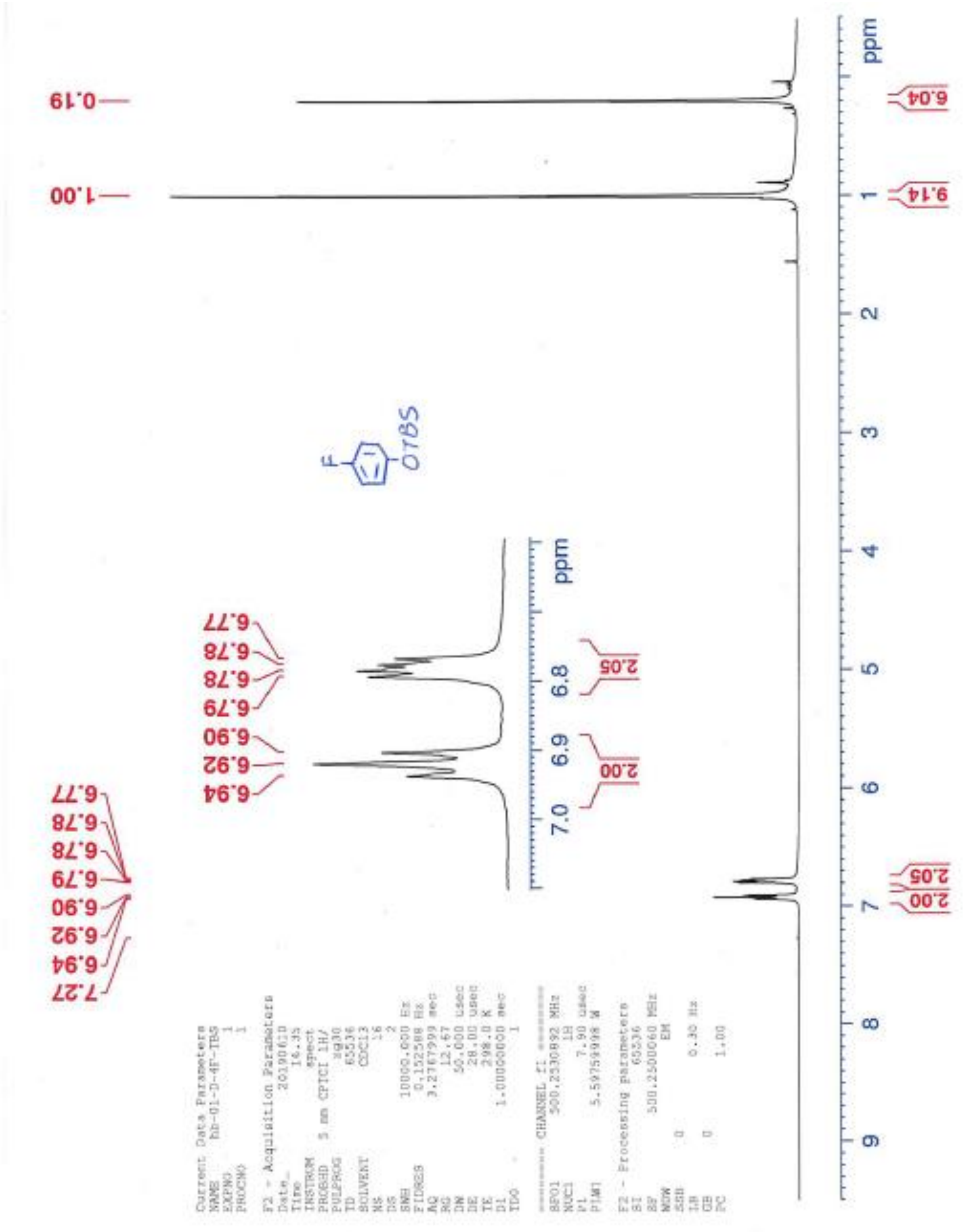

Supplementary Figure 93: ^{13}C NMR of *tert*-butyl(4-fluorophenoxy)dimethylsilane (**15g**)

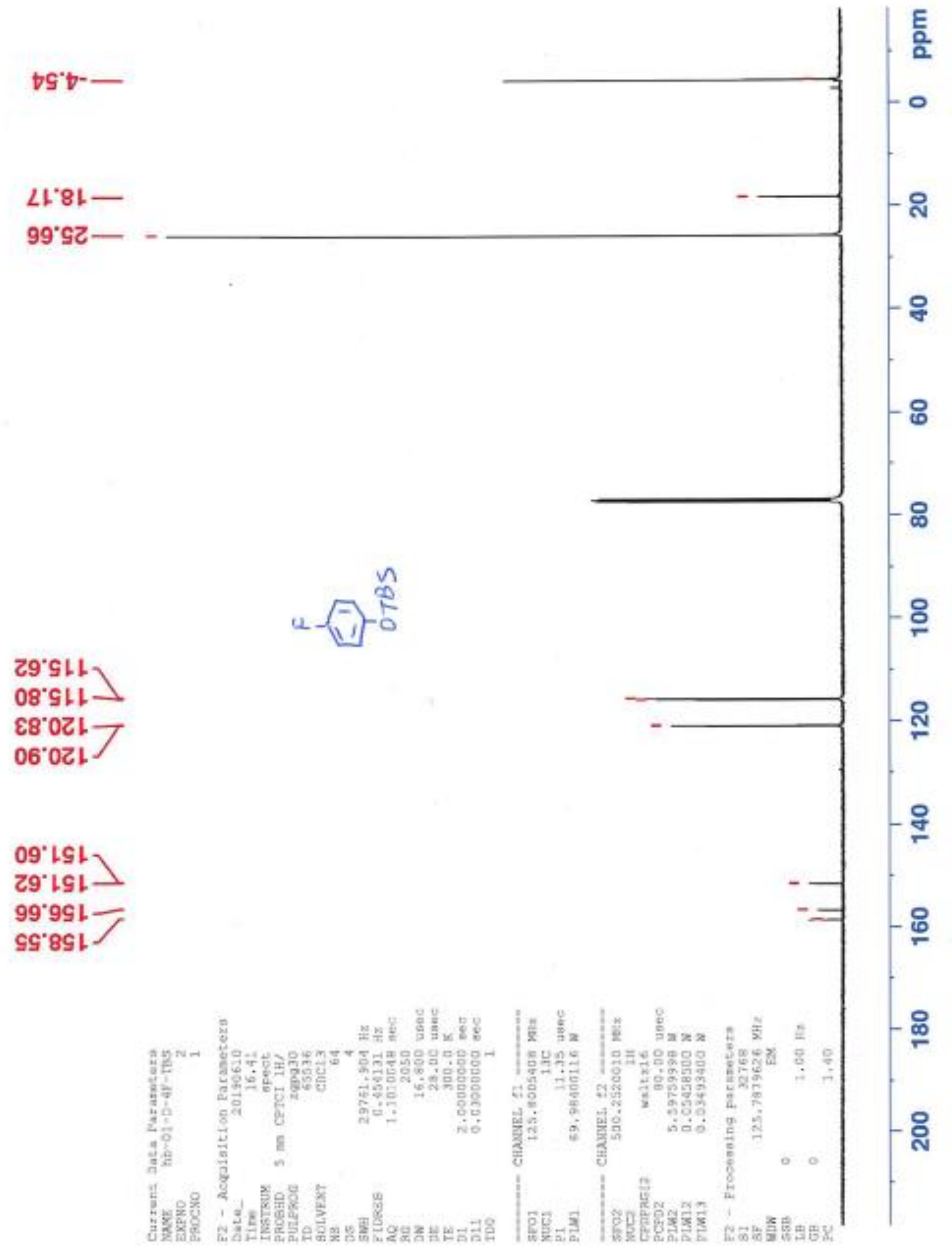

Supplementary Figure 94: ^{1}H NMR of *tert*-butyldimethyl(naphthalen-2-yloxy)silane (**15i**)

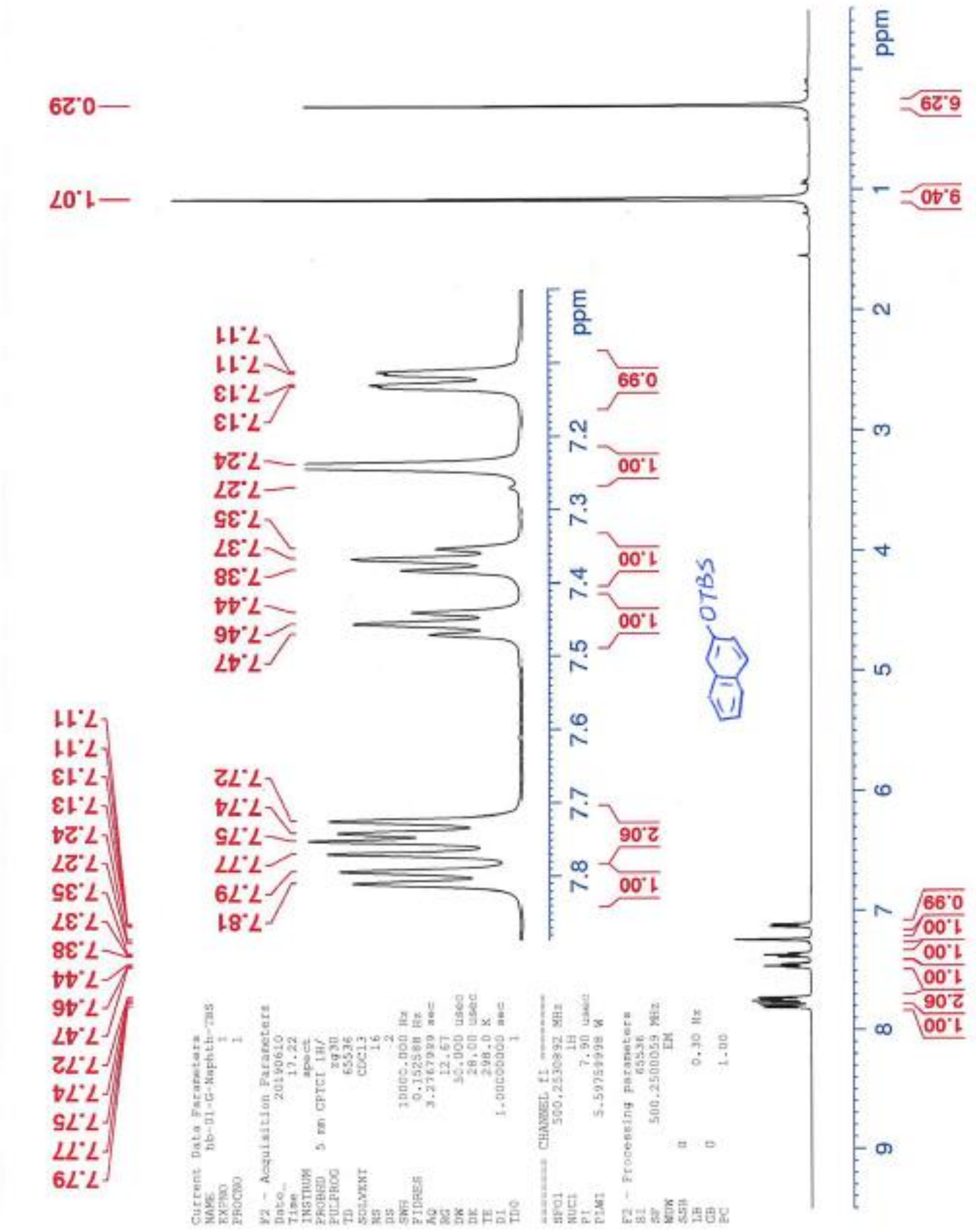

Supplementary Figure 95: ^{13}C NMR of *tert*-butyldimethyl(naphthalen-2-yloxy)silane (**15i**)

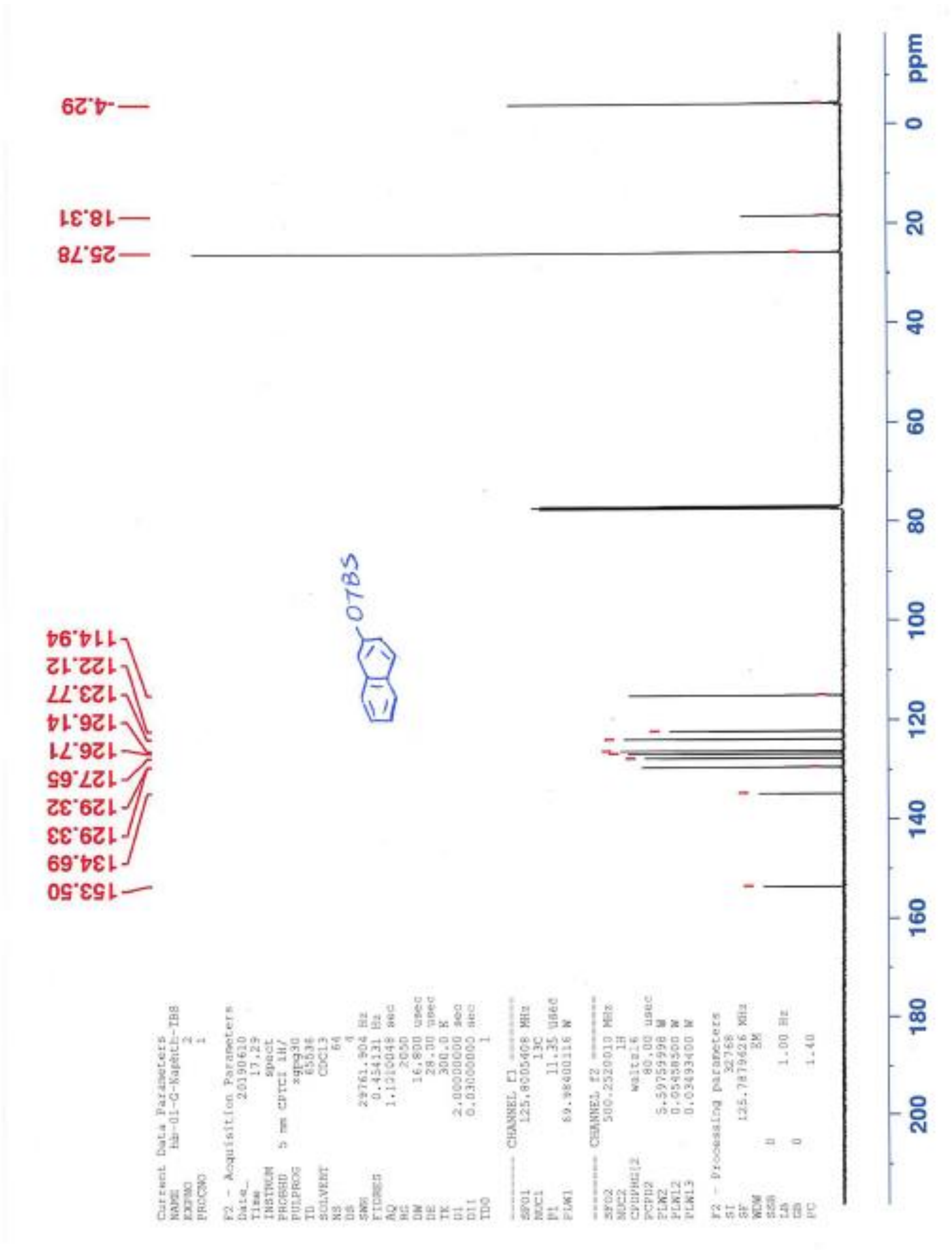

Supplementary Figure 96: ^{1}H NMR of (S)-2,6-bis((*tert*-butyldimethylsilyl)oxy)-3,5-dichloro-N-((1-ethylpyrrolidin-2-yl)methyl)benzamide (**17**)

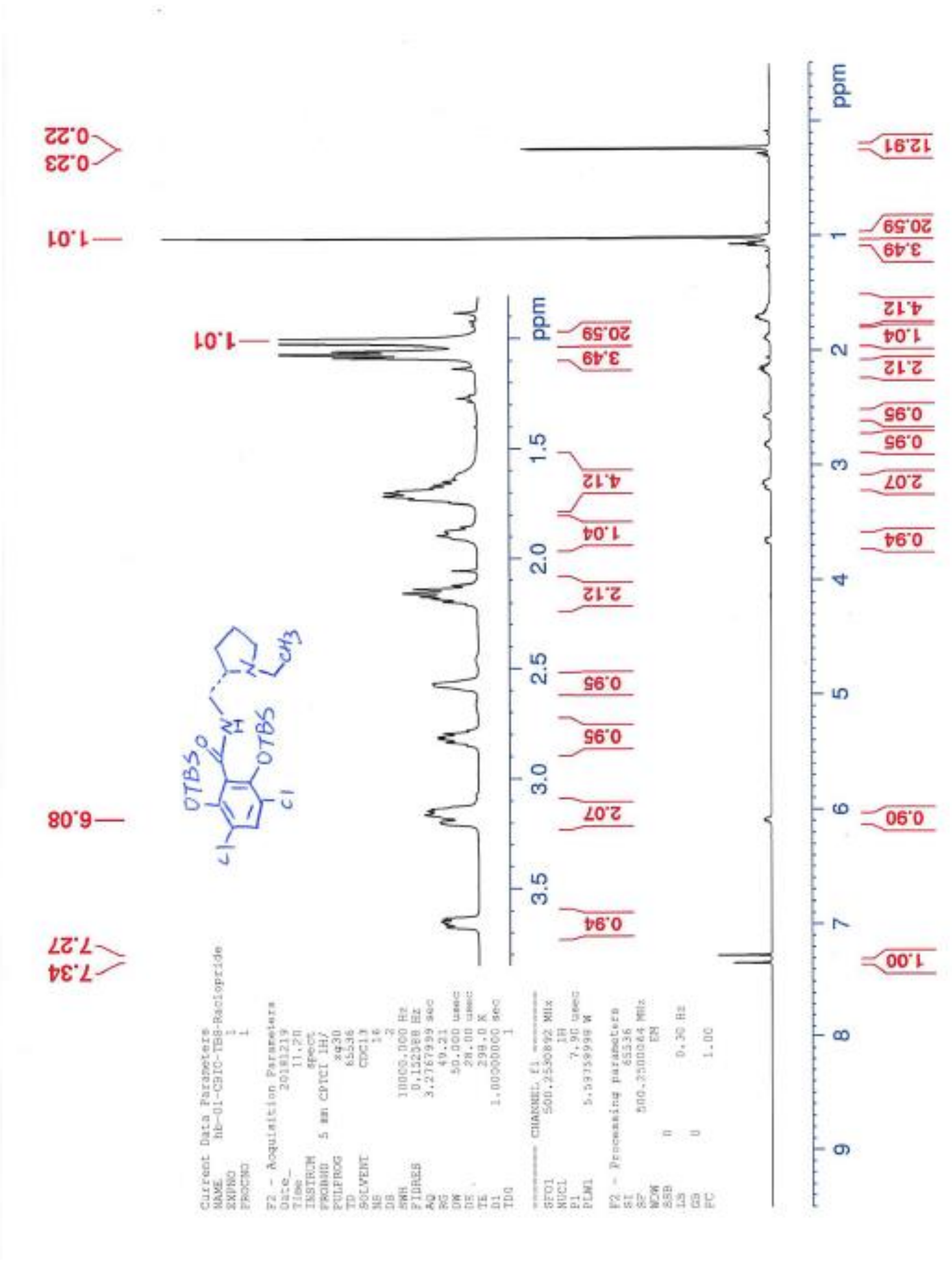

Supplementary Figure 97: ^{13}C NMR of (S)-2,6-bis((*tert*-butyldimethylsilyl)oxy)-3,5-dichloro-N-((1-ethylpyrrolidin-2-yl)methyl)benzamide (**17**)

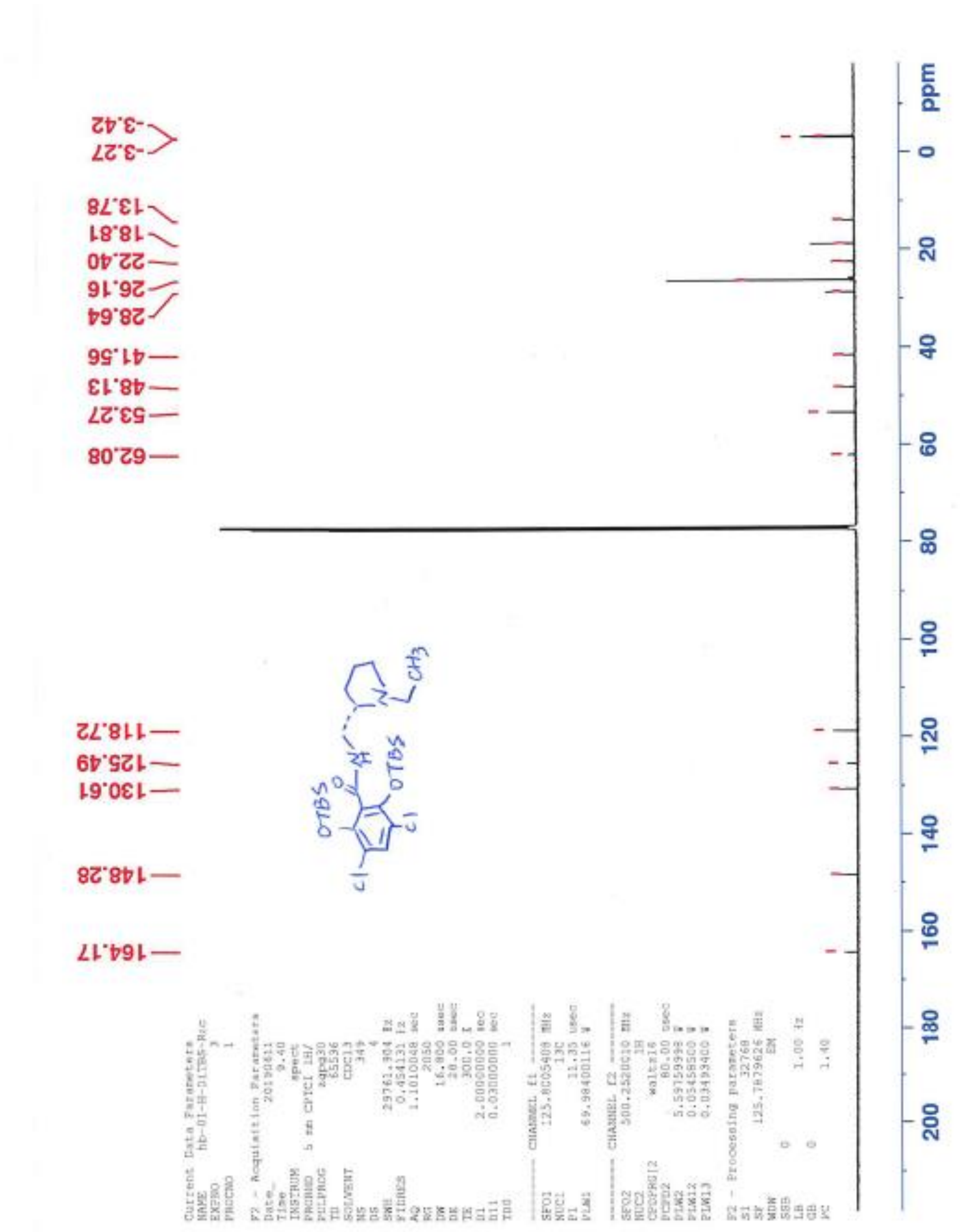

Supplementary Figure 98: ^{1}H NMR of ethyl 2-(4-isobutylphenyl)acetate

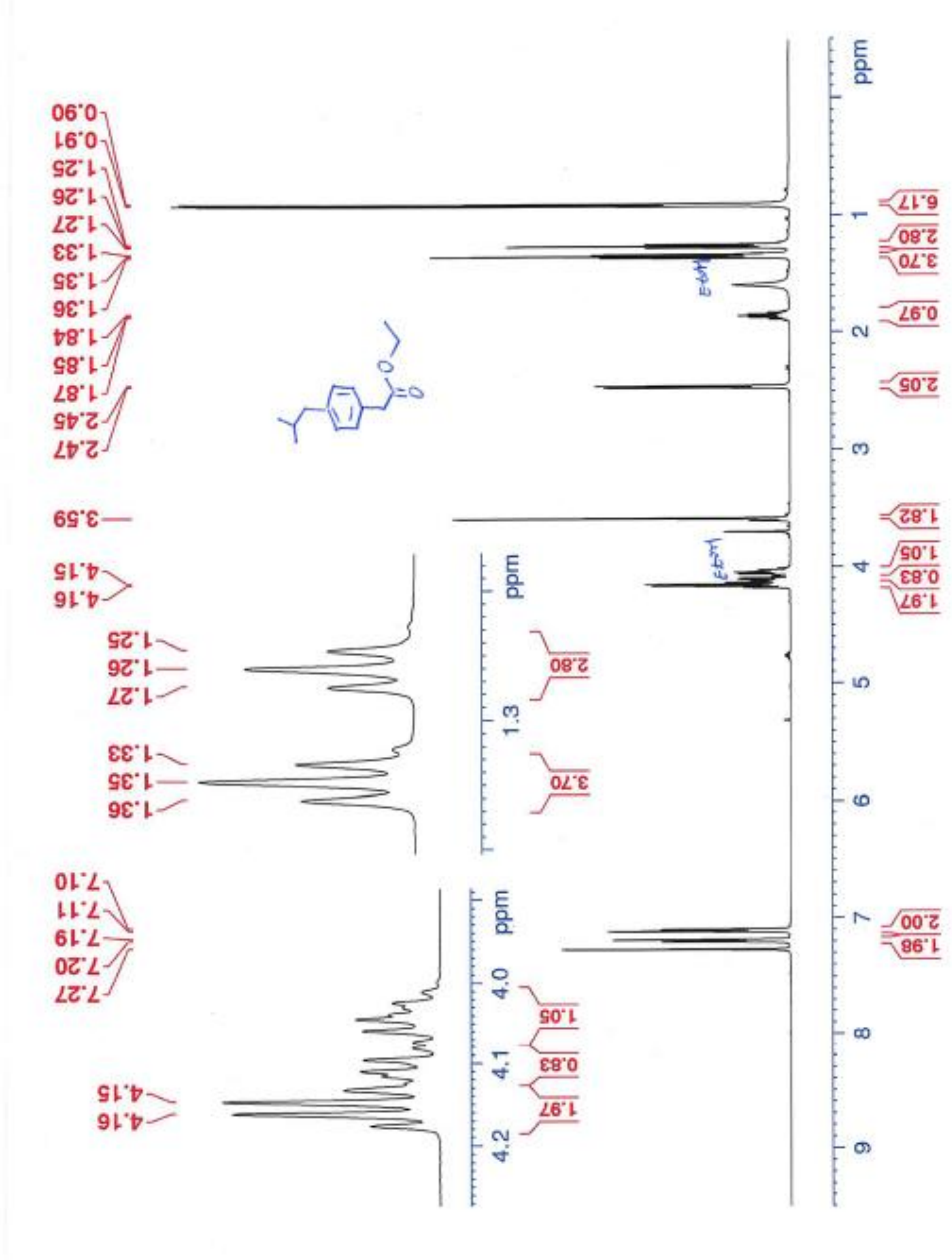

Supplementary Figure 99: ^{13}C NMR of ethyl 2-(4-isobutylphenyl)acetate

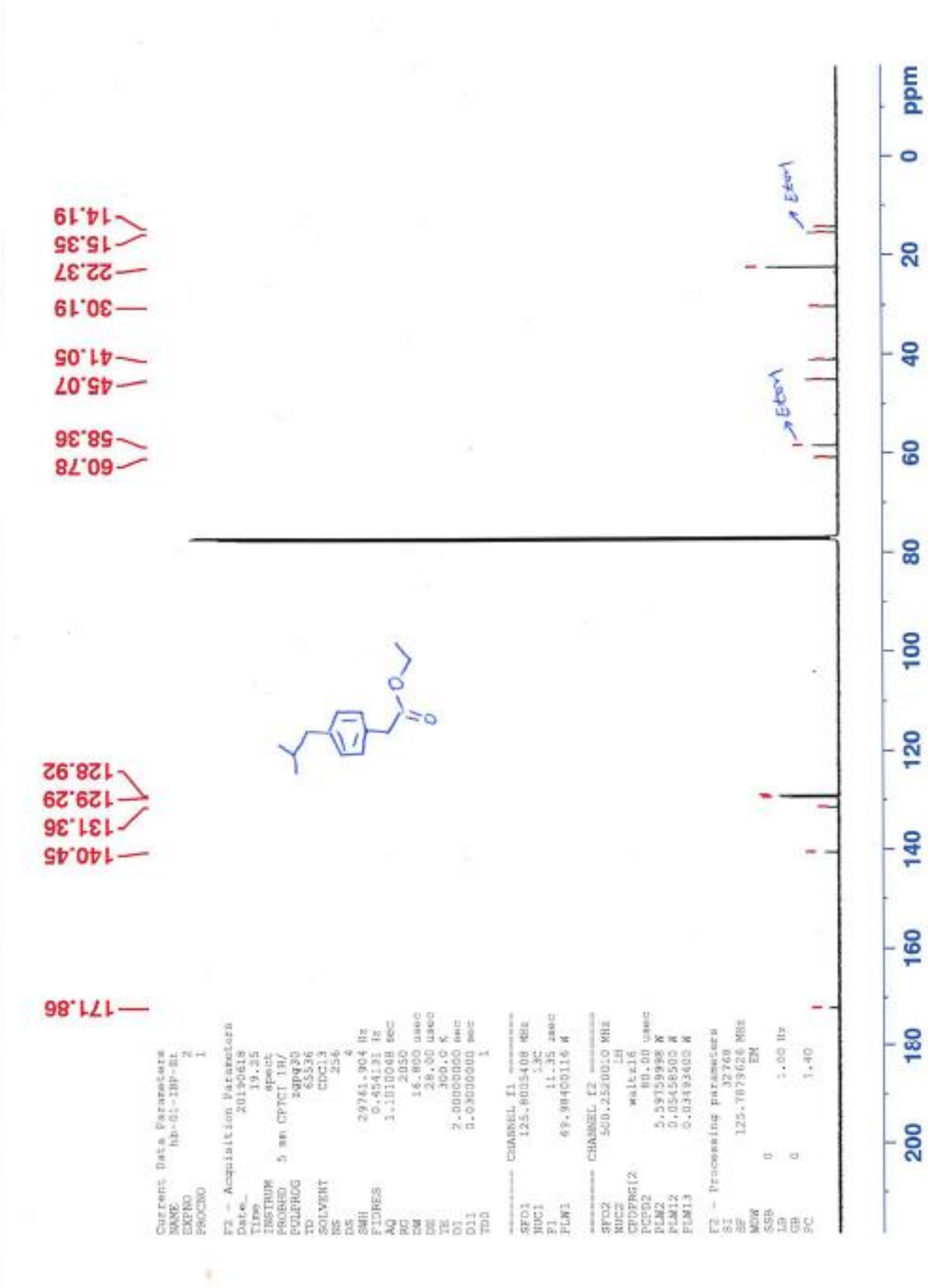

Supplementary Figure 100: ^{1}H NMR of ethyl 2-diazo-2-(4-isobutylphenyl)acetate

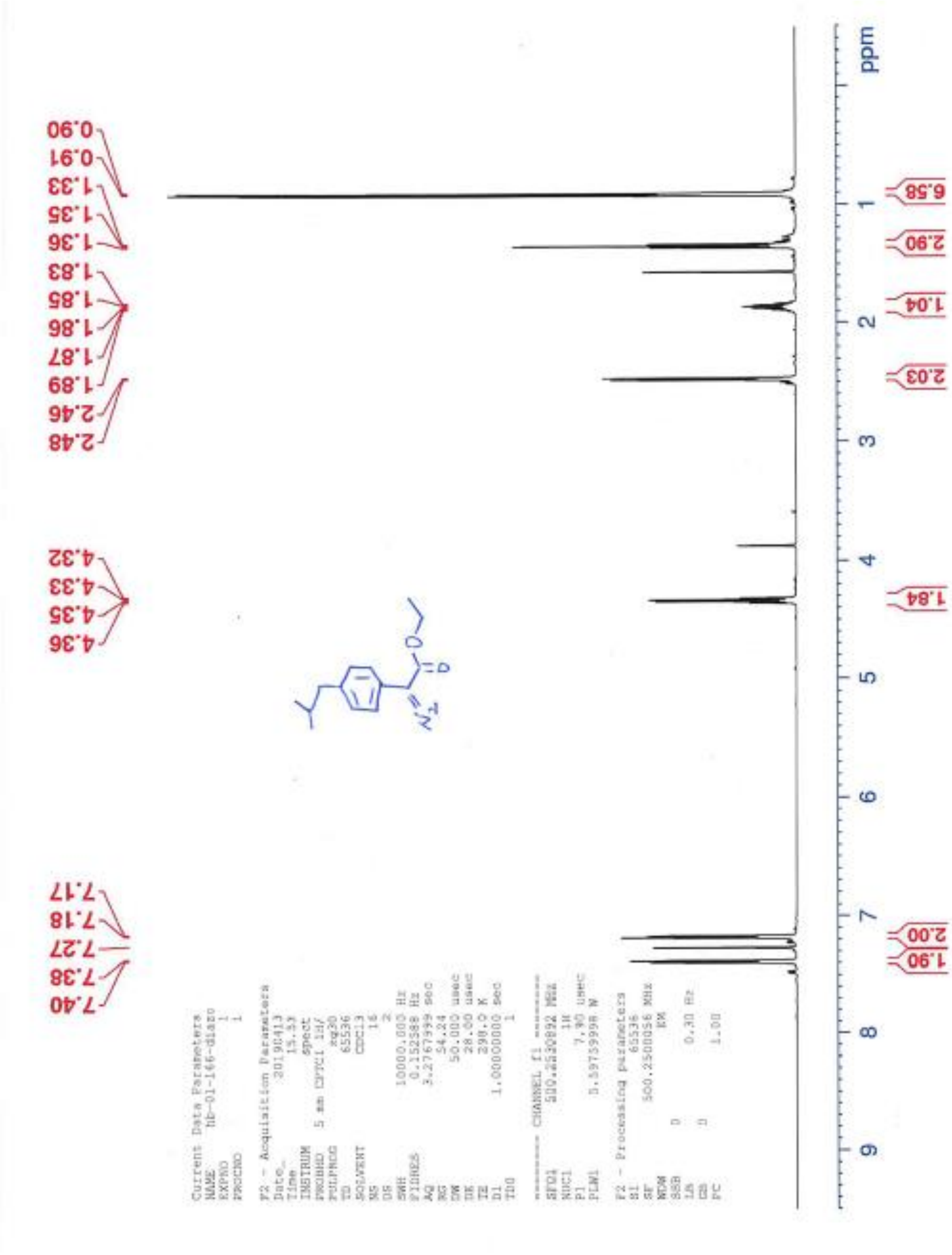

Supplementary Figure 101: ^{13}C NMR of ethyl 2-diazo-2-(4-isobutylphenyl)acetate

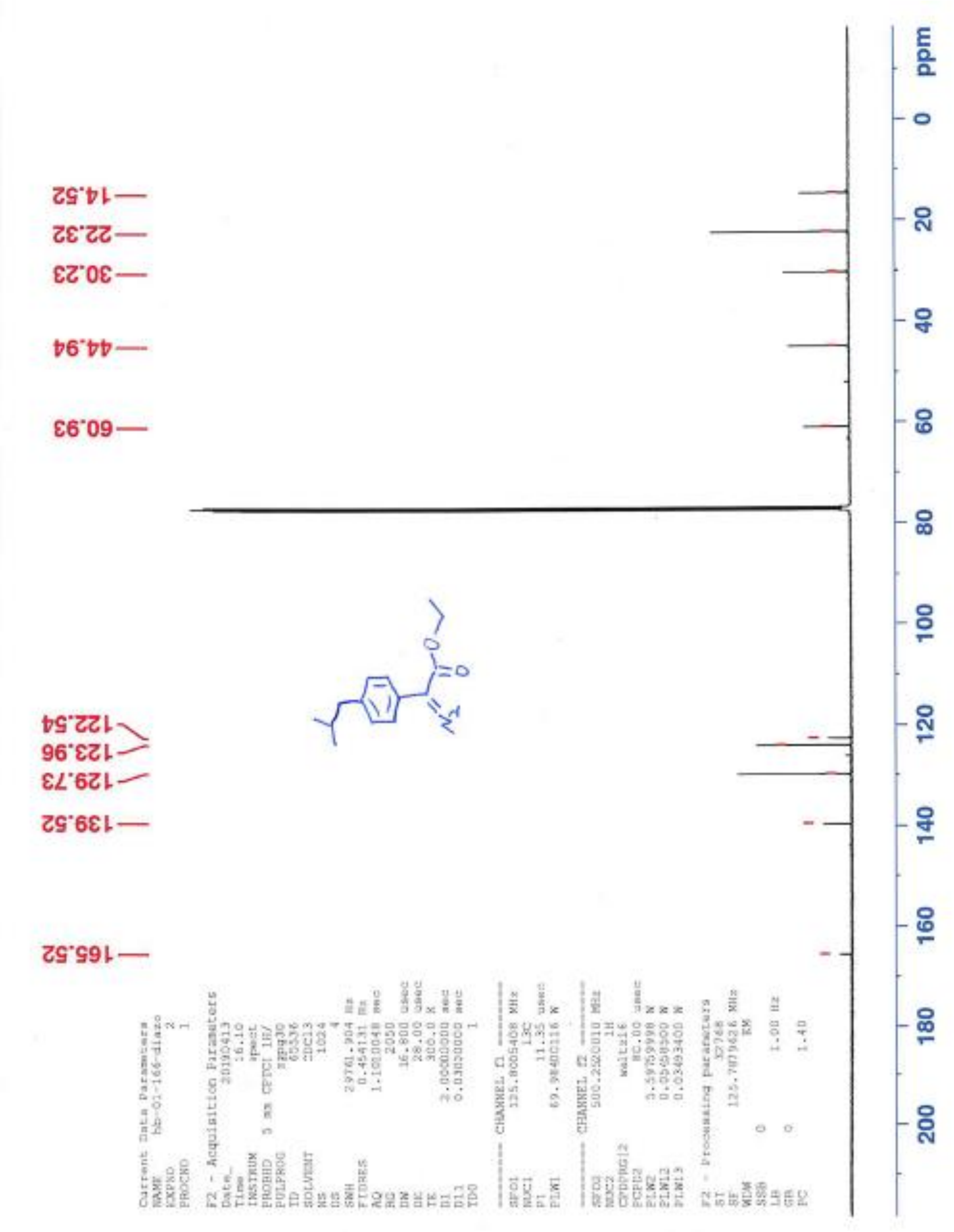

Supplementary Figure 102: ^{1}H NMR of ethyl 2-(4-*iso*butylphenyl)-2-(triethylsilyl)acetate (**15o**)

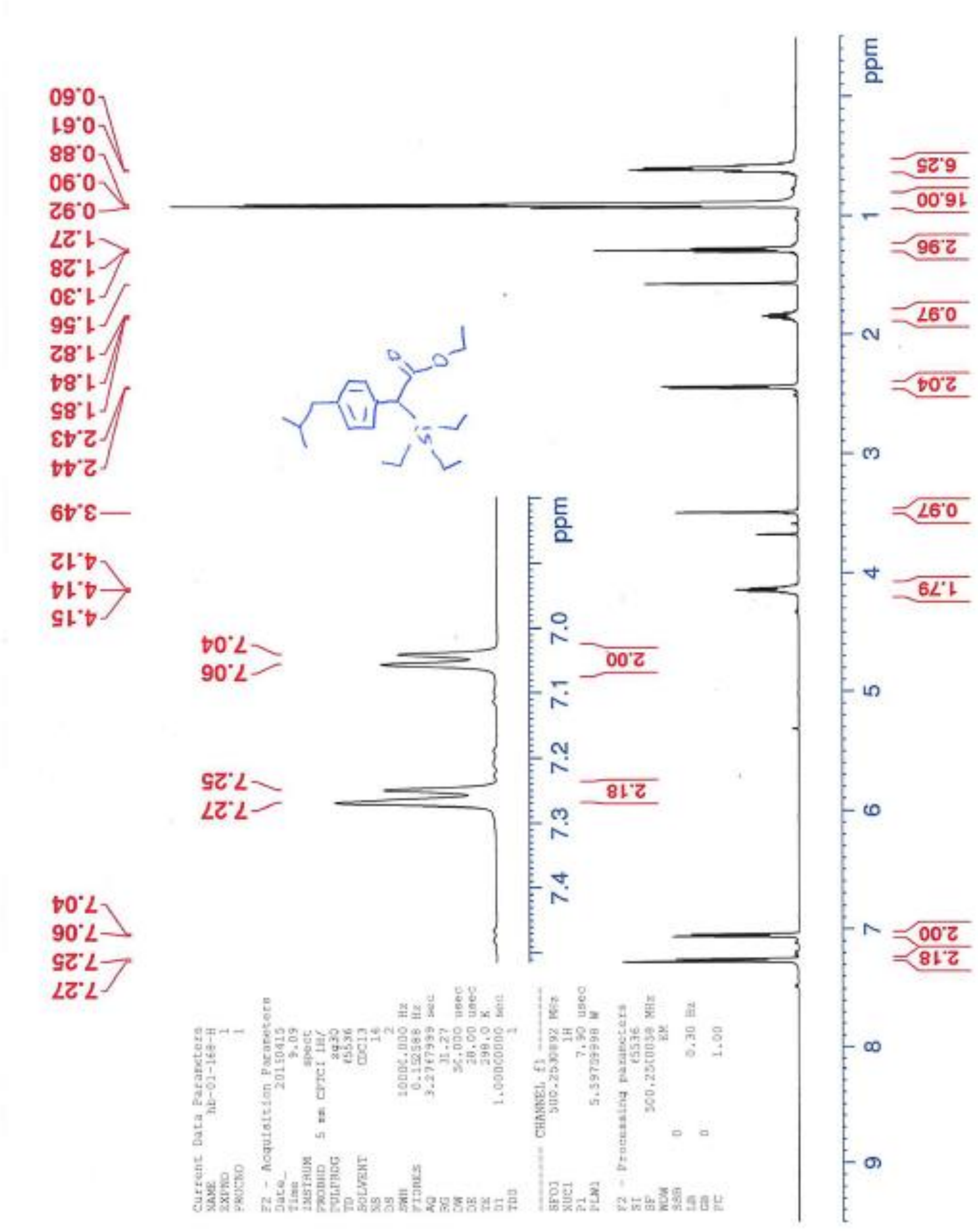

Supplementary Figure 103: ^{13}C NMR of ethyl 2-(4-*iso*butylphenyl)-2-(triethylsilyl)acetate (**15o**)

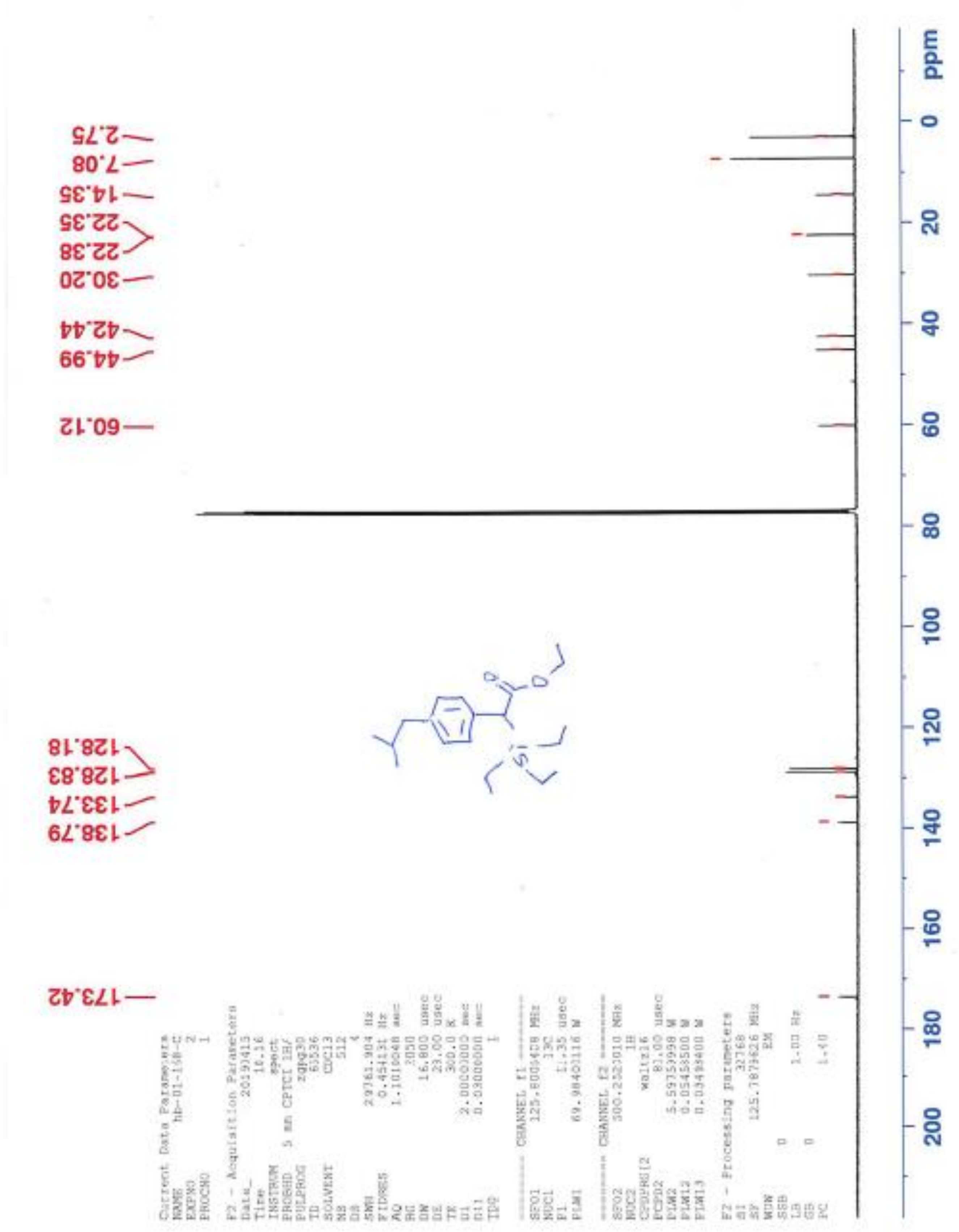

Supplementary References

1. M. Yonemoto-Kobayashi, K. Inamoto, Y. Tanaka, Y. Kondo, Carboxylation of alkynylsilanes with carbon dioxide mediated by cesium fluoride in DMSO. *Org. Biomol. Chem.* **11**, 3773−3775 (2013).

2. M. Arndt, E. Risto, T. Krause, L. J. Gooßen, C-H carboxylation of terminal alkynes catalyzed by low loadings of silver(I)/DMSO at ambient CO_2 pressure. *Chem. Cat. Chem*, **4**, 484–487 (2012).

3. H. Zheng, R. McDonald, D. G. Hall, Boronic acid catalysis for mild and selective [3+2] dipolar cycloadditionsto unsaturated carboxylic acids. *Chem. Eur. J.*, **16**, 5454-5460 (2010).

4. T. P. Luo, M. J. Dai, S. L. Zheng, S. L. Schreiber, Syntheses of α-pyrones using gold-catalyzed coupling reactions. *Org. Lett.*, **13**, 2834-2836 (2011).

5. X. Zhang, W.-Z. Zhang, X. Ren, L.-L. Zhang, X.-B. Lu, Ligand-free Ag(I)-catalyzed carboxylation of terminal alkynes with CO_2. *Org. Lett.* **13**, 2402–2405 (2011).

6. Z. Zhang, W. Wu, J. Liao, J. Li and H. Jiang, Intermolecular asymmetric carboesterification of alkenes by using chiral amine auxiliaries under O_2: synthesis of enantioenriched α-methylene-γ-lactones through chloropalladation of alkynes. *Chem. Eur. J.*, **21**, 6708–6712 (2015).

7. L. Biancalana, L. K. Batchelor, A. De Palo, S. Zacchini, G. Pampaloni, P. J. Dyson, F. Marchetti, A general strategy to add diversity to ruthenium arene complexes with bioactive organic compounds via a coordinated (4-hydroxyphenyl) diphenylphosphine ligand. *Dalton Trans.*, **46**, 12001–12004 (2017).

8. D. Peña, A. Cobas, D. Pérez, E. Guitián, An efficient procedure for the synthesis of ortho-trialkylsilylaryl triflates: easy access to precursors of functionalized arynes. *Synthesis* **10**, 1454–1458 (2002).

9. H. J. Shirley, C. D. Bray, Spiroketal formation by cascade oxidative dearomatization: an approach to the phorbaketal skeleton. *Eur. J. Org. Chem.* 1504–1507 (2016).

10. Q. Shelby, N. Kataoka, G. Mann, J. Hartwig, Unusual in situ ligand modification to generate a catalyst for room temperature aromatic C−O bond formation. *J. Am. Chem. Soc.* **122**, 10718-10719 (2000).

11. C.-E. Yeom, Y. J. Kim, S. Y. Lee, Y. J. Shin, B. M. Kim, Efficient chemoselective deprotection of silyl ethers using catalytic1-chloroethyl chloroformate in methanol. *Tetrahedron* **61**, 12227–12237 (2005).

12. K. G. Zbinden, D. W. Banner, K. Hilpert, J. Himber, T. Lavé, M. A. Riederer, M. Stahl, T. B. Tschopp, U. Obst-Sander, Dose-dependent antithrombotic activity of an orally active tissuefactor/factor VIIa inhibitor without concomitantenhancement of bleeding propensity. *Bioorg. Med. Chem.* **14**, 5357–5369 (2006).

13. A. Wiebe, D. Schollmeyer, K. M. Dyballa, R. Franke, S. R. Waldvogel, Selective synthesis of partially protected nonsymmetric biphenols by reagent- and metal-free anodic cross-coupling reaction. *Angew. Chem. Int. Ed.* **55**, 11801-11805 (2016).

14. B. S. Bodnar and P. F. Vogt, An improved Bouveault−Blanc ester reduction with stabilized alkali metals. *J. Org. Chem.* **74**, 2598–2600 (2009).

15. Tamura, Y.; Choi, H. D.; Shindo, H.; Ishibashi, H. Synthesis of ethyl arylacetates by means of Friedel-Crafts reaction of aromatic compounds with ethyl α-chloro-α-(methylthio) acetate. *Chem. Pharm. Bull.* **30**, 915-921 (1982).

16. H. Keipour and T. Ollevier, Iron-catalyzed carbene insertion reactions of α-diazoesters into Si–H bonds. *Org. Lett.* **19**, 5736−5739 (2017).

17. H. Keipour, A. Jalba, L. Delage-Laurin, T. Ollevier, Ligand-free Ag(I)-catalyzed carboxylation of terminal alkynes with CO_2. *J. Org. Chem.* **82**, 3000−3010 (2017).

18. Tremblay, S.; Ouellet, R.; Rodrigue, S.; Langlois, R.; Benard, F.; Cunnane, S. C., Automated synthesis of ^{11}C-acetoacetic acid, a key alternate brain fuel to glucose. *Applied Radiation and Isotopes* **65**, 934-940 (2007).

19. Prenen, G. H. M.; Go, K. G.; Zuiderveen, F.; Paans, A. M. J.; Vaalburg, W., An improved synthesis of carbon-11 labeled acetoacetic acid and an evaluation of its potential for the investigation of cerebral pathology by positron emission tomography. *Applied Radiation and Isotopes* **1990, 41**, 1209-16 (1990).
